# Evaluating the Incorporation of Picolinamide Pendants into the Macropa Scaffold for Pb(II)- and Bi(III)-Based Radiopharmaceuticals

**DOI:** 10.1021/acs.inorgchem.6c02096

**Published:** 2026-06-22

**Authors:** Charlene Harriswangler, Parmissa Randhawa, Nicolás Sommariva-Ucha, Luke Wharton, Laura Valencia, Brooke L. McNeil, Hua Yang, David Esteban-Gómez, Carlos Platas-Iglesias, Caterina F. Ramogida

**Affiliations:** † CICA—Centro Interdisciplinar de Química E Bioloxía and Departamento de Química, Facultade de Ciencias, 16737 Universidade da Coruña, A Coruña, Galicia 15071, Spain; ‡ Department of Chemistry, 1763 Simon Fraser University, 8888 University Drive, Burnaby, British Columbia V5A 1S6, Canada; § Life Sciences Division, 10084 TRIUMF, 4004 Wesbrook Mall, Vancouver, British Columbia V6T 2A3, Canada; ∥ Departamento de Química Inorgánica, Facultad de Ciencias, 16784 Universidade de Vigo, As Lagoas, Marcosende, Pontevedra 36310, Spain; ⊥ Department of Chemistry, University of British Columbia, 2036 Main Mall, Vancouver, British Columbia V6T 1Z1, Canada

## Abstract

We report a detailed study on how the substitution of carboxylate groups of macropa for amides affects the coordination of Pb­(II) and Bi­(III). We describe a new synthesis of macropam, improving on previously reported yields. Pb­(II) and Bi­(III) complexes of macropa, macropapam, and macropam were prepared *in situ* and characterized. Crystal structures were obtained using XRD which were analyzed using eccentricity measurements and NBO analysis, showing that the lone pair is more active in Bi­(III) complexes than in Pb­(II) analogues and this activity decreases upon incorporation of amides. NMR and X-ray crystallography of [Bi­(macropa)]^+^ evidenced a certain degree of fluxionality. This led to a detailed computational study in which the energies of the transition states for the switching of the X-CH_2_–CH_2_–X units of the macrocycle between λ and δ conformations were calculated. The relatively low energies of the transition states suggest that this process takes place in solution on the μs time scale. Radiolabeling with [^203^Pb]­Pb­(II) and [^213^Bi]­Bi­(III) and stability assays of the [^203^Pb]­Pb­(II)-labeled complexes showed that the incorporation of amides worsens radiochemical performance. Overall, the incorporation of amides is detrimental for radiopharmaceutical applications, highlighting that as borderline acids, Pb­(II) and Bi­(III) at times prefer harder (negatively charged) donor atoms.

## Graphical abstract

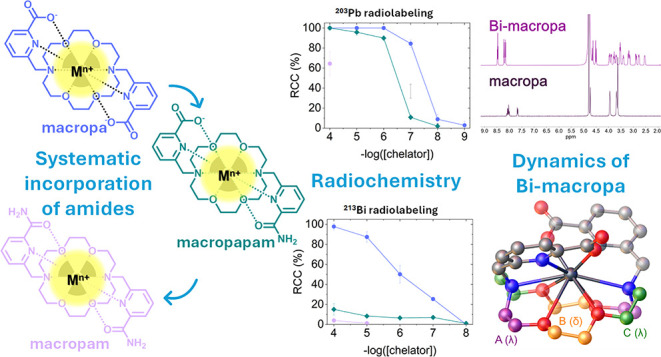

## Introduction

The expanding catalog of medically relevant radionuclides has strengthened the role of radiometal-based agents in modern diagnostic and therapeutic nuclear medicine. Diagnostic applications typically employ γ- or β^+^-emitting radionuclides, for Single Photon Emission Computed Tomography (SPECT) and Positron Emission Tomography (PET), respectively, whereas therapeutic isotopes utilize α particles, β^–^ particles, or Auger electrons to achieve targeted cytotoxicity. The concept of *theranostics* unites these capabilities by pairing isotopes of the same element so that a single molecular platform can first be used to visualize disease and subsequently to treat it, while maintaining closely matched pharmacokinetic and biodistribution profiles.^1,2^

As interest in radiometal theranostics increases, so does the need for chelators capable of binding specific metal ions with high stability, rapid complexation kinetics, and compatibility with biological targeting vectors. Chelator design is fundamentally governed by the Hard–Soft Acid–Base (HSAB) character of the radiometal and the selected donor atoms.^3,4^ Lead­(II) and Bismuth­(III) are isoelectronic cations considered to be borderline or “intermediate” Lewis acids and are of particular importance due to the emerging lead-212 (^212^Pb, *t*_1/2_ = 10.6 h)/lead-203 (^203^Pb, *t*_1/2_ = 51.9 h) theranostic pair and α-emitting radioisotopes bismuth-212 (^212^Bi, *t*_1/2_ = 60.55 min) and bismuth-213 (^213^Bi, *t*_1/2_ = 45.59 min), the former resulting from the decay of ^212^Pb.^5,6^ The SPECT isotope ^203^Pb undergoes electron-capture decay with emission of a 279 keV γ-photon, making it useful for imaging, while ^212^Pb provides therapeutic benefit through β^–^ and α radiation delivered via its decay chain while also allowing for SPECT imaging through the 238.6 keV and 75 to 91 keV γ-photons.^7,8^

Cyclen-based ligands such as DOTA and DOTAM (TCMC) remain widely used in radiopharmaceuticals. A variety of alternate chelators have been explored for their ability to form stable complexes with both Pb­(II) and Bi­(III). These chelators offer different donor atom environments that modulate their affinity for intermediate Lewis acids. More recently, ligand systems including alternative cyclen-based chelators,^9–12^ [2.2.2] cryptands,^13^ 18-membered macrocycles,^14–20^ bispidines,^21^ as well as several acyclic systems,^22–24^ have broadened the landscape of Pb­(II)-chelation strategies. Notably, macropa (Chart [Fig chart1]), an 18-membered macrocycle, has emerged as a leading platform due to its ability to coordinate the relatively large ionic radii of these ions while forming kinetically inert complexes and outperforming traditional macrocycles such as DOTA with respect to complexation efficiency and stability.^18,19^ The Pb­(II) complex of macropa (then referred to as H_2_bp18c6) was first studied nearly 15 years ago when it was shown that the ligand shows high selectivity for large metal ions, with the authors proposing applications in chelation treatment for metal intoxication.^25^

**Scheme 1:**
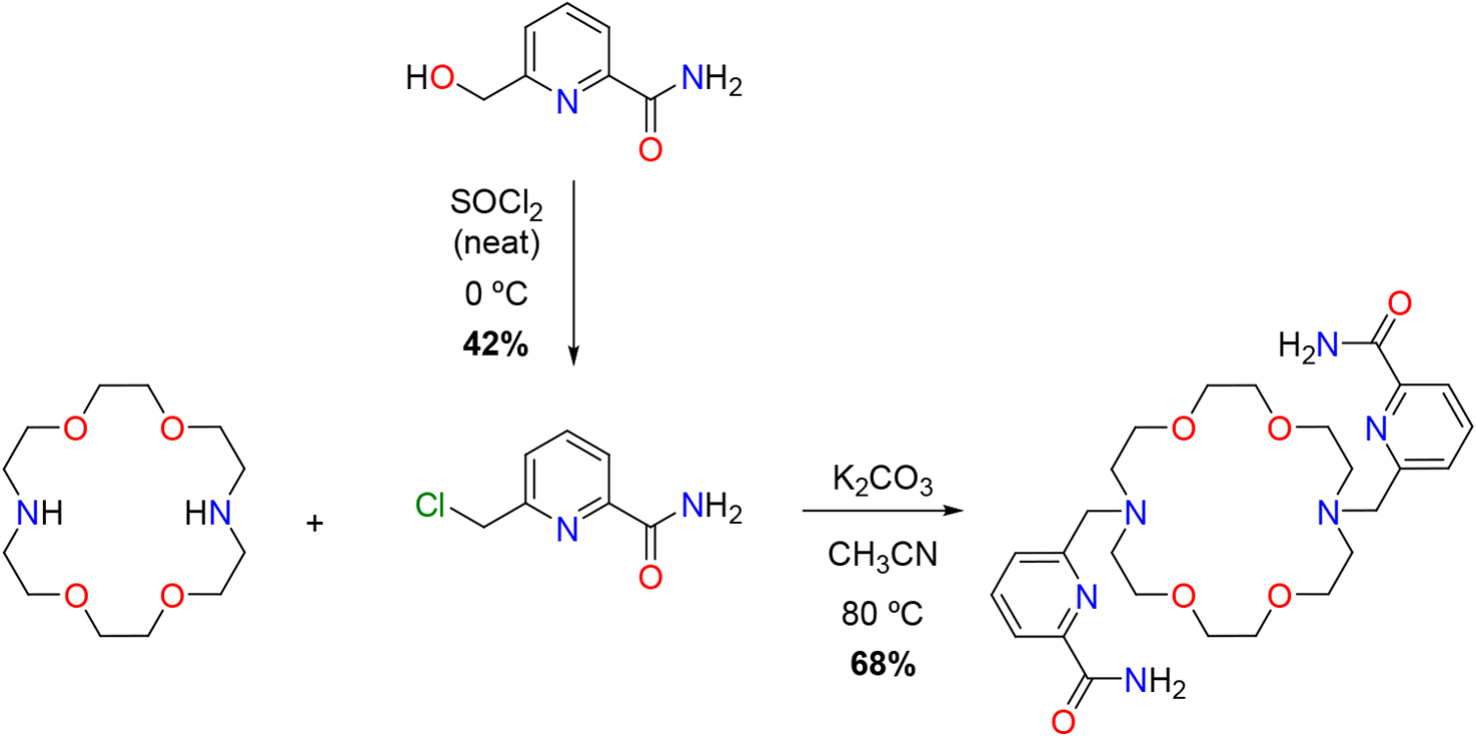
New Synthetic Procedure for the Preparation of the Chelator Macropam

A distinctive feature of Pb­(II) and Bi­(III) coordination chemistry is the stereochemical influence of the 6s^2^ lone pair, which can give rise to hemidirected or holodirected geometries depending on factors such as ligand environment, coordination number, and donor atom type, among others.^26,27^ Hard, anionic donors tend to favor the stereochemical activity of the lone pair and therefore, hemidirected structures, whereas softer, neutral donors more often promote holodirected arrangements. These considerations suggest that tuning donor softness within the chelator framework will influence the activity of this lone pair, possibly affecting other parameters such as radiochemical yields, complex stability, or kinetic inertness. In one of the earliest publications on the development of bifunctional chelators for lead-203 and lead-212, it was shown that DOTAM provided more inert radiocomplexes than DOTA.^28^ Recent studies have further investigated the substitution of carboxylate donor groups for amides, with varying results. In the case of ligands from the pyta family (Chart [Fig chart1]), the presence of amides, which favors a more holodirected environment, results in increased kinetic inertness;^14,15^ for the pada and padam chelators (Chart [Fig chart1]), the carboxylate pendants are favorable for providing a more inert complex;^16^ while in the final example, the inertness of the complexes formed with DO2A-2Py and DCMC-2Py (Chart [Fig chart1]) was not affected by the different pendant arms, though the radiolabeling efficiency was better with the carboxylate pendants.^9^ Motivated by these principles, we sought to study a series of macropa-derived ligands in which the picolinic acid carboxylates are systematically replaced by amide groups, producing two derivatives, macropapam and macropam, with increased donor softness (Chart [Fig chart1]). These chelators were previously reported for the stabilization of Eu­(II).^29^ Using this ligand series, we investigated how donor-atom modification impacts Pb­(II) and Bi­(III) coordination behavior through NMR spectroscopy, X-ray crystallography, and DFT calculations. Radiochemical studies with [^203^Pb]­Pb­(II) and [^213^Bi]­Bi­(III) were employed to further evaluate the labeling efficiency and kinetic inertness of the complexes. Together, these results provide new insight into chelator design for intermediate radiometals and support the development of next-generation Pb­(II)-based radiotheranostic agents.

**1 chart1:**
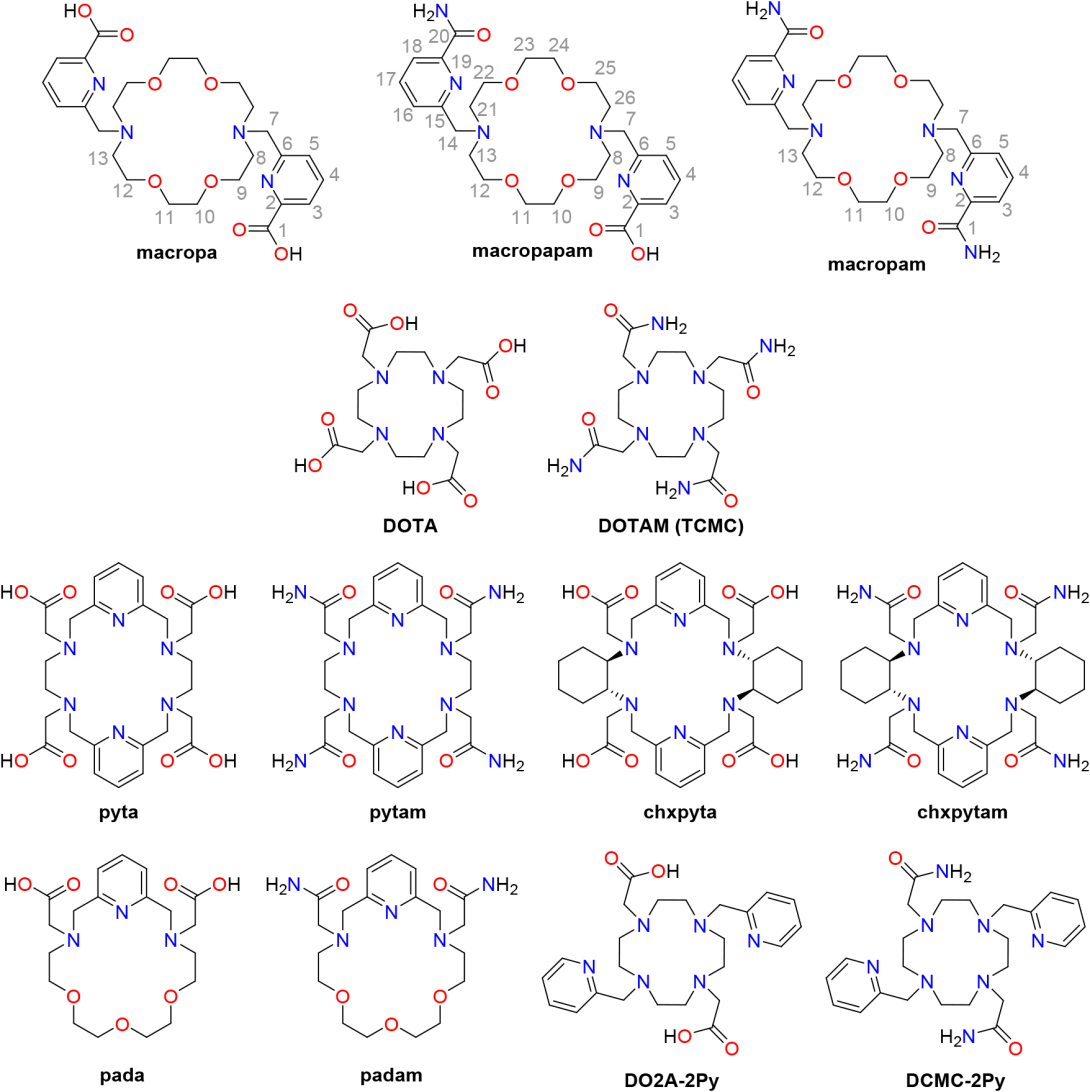
Chelators Discussed in This Work and Numbering Scheme Used for NMR Spectral Assignment[Fn chart1-fn1]

a The protons are excluded from the names for simplification.

## Results and Discussion

### Synthesis and Characterization of the Chelators

All three chelators (macropa, macropapam, and macropam) were previously reported and prepared according to the published procedures.^29,30^ We also carried out an alternative synthesis of the ligand macropam (Scheme 1). In this strategy, the hydroxyl group of 6-(hydroxymethyl)­picolinamide, which is commercially available, is converted into a chloride, which is then used to alkylate the macrocyclic backbone. This procedure is a more direct synthesis of the ligand than the use of ammonia to convert the methyl ester of macropa into macropam, while also avoiding mixtures with the macropapam ligand. The alkylation proceeded with a reasonable yield of 68%, while the reported synthesis in aqueous ammonia afforded a lower 43% yield.

Although the crystal structure of macropam was described previously,^29^ a new crystal structure with a different conformation of the ligand was obtained. Slow evaporation of an acidic aqueous solution containing macropam to which KPF_6_ was added resulted in the crystallization of the protonated ligand. The resulting colorless needles were shown to crystallize in the monoclinic *C2/c* space group, with the asymmetric unit containing half of the chelator, one PF_6_^–^ anion, and a water molecule with 0.5 occupation (one water molecule per macrocycle). In this new structure, the picolinamide pendant arms are in a *syn* conformation, with a water molecule interacting through hydrogen bonds with two ether oxygen atoms of the macrocycle (Figure 1). As was described for the previous crystal structure, the primary amide groups form hydrogen bonds with two neighboring macrocycles, in what is known as an R_2_^2^(8) motif.^31^ In this motif, the hydrogen atom that is *cis* to the carbonyl group of an −NH_2_ group interacts with the oxygen atom of a carbonyl group of an adjacent macrocycle, while the carbonylic oxygen of the first ligand interacts with the *cis* hydrogen atom of the −NH_2_ group of the second ligand (Figure S1, Supporting Information).

**Figure 1:**
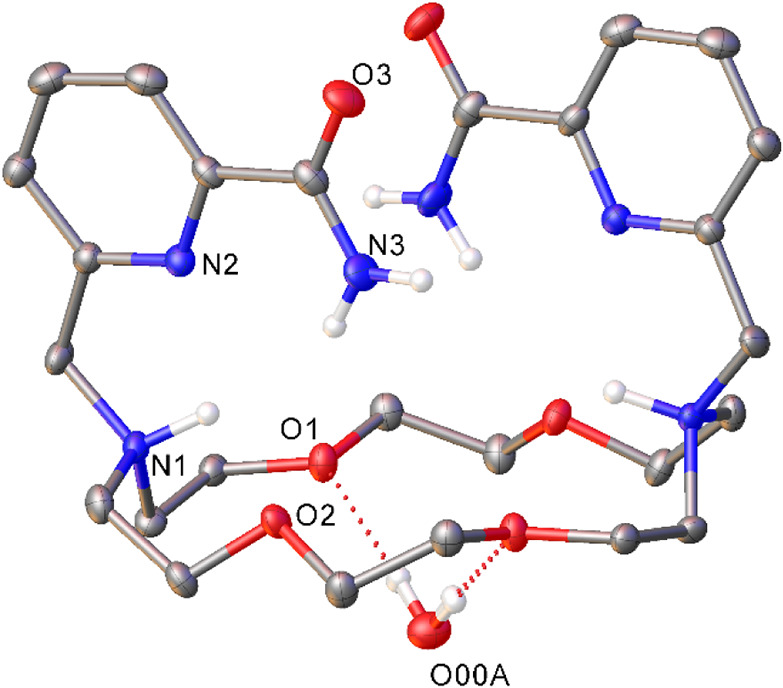
X-ray crystal structure of [H_2_macropam]­(PF_6_)_2_·H_2_O with atom numbering. Anions and hydrogen atoms bonded to carbon atoms are omitted for simplicity. ORTEP plot is at 50% probability.

### X-ray Crystal Structures of [Bi­(macropa)]^+^, [Bi­(macropapam)]^2+^, and [Pb­(macropam)]^2+^

Single crystals appropriate for X-ray diffraction were obtained for three of the studied complexes, [Bi­(macropa)]^+^, [Bi­(macropapam)]^2+^, and [Pb­(macropam)]^2+^, through addition of KPF_6_ to aqueous solutions of the complexes at approximately neutral pH (see Experimental Section for more details). Different views of the crystal structures can be seen in Figure 2, while bond lengths, selected angles, and additional information are reported in Table 1. There are two sources of helicity in this type of complexes where the picolinate arms adopt a *syn* conformation: One which arises from the conformation of the five-membered chelate rings formed between the X–CH_2_–CH_2_–X (X = N or O) units of the macrocycle and the metal center (λ or δ); and that which originates through the wrapping of the pendant arms (Λ or Δ, see Supporting Information, Figure S2).

**Table 1:** Interatomic Distances (Å), Angles (°), Conformation, NBO Analysis and Eccentricity (ε) of the Metal Coordination Environment in Crystals of [Bi­(macropa)]^+^, [Bi­(macropapam)]^2+^, and [Pb­(macropam)]^2+^, along with the Same Information for Previously Reported Crystal Structures of [Bi­(macropa)]^+^ and [Pb­(macropa)]

Metal Ligand	Pb(II) macropam		Pb(II) macropa^25^		Bi(III) macropa		Bi(III) macropapam		Bi(III) macropa^18^	
Pyridine N	N3	2.608(6)	N1	2.585	N2	2.4343(15)	N3	2.416(4)	N2	2.4419
	N5	2.637(5)	N4	2.601			N4	2.479(5)	N4	2.413
Carboxylate O			O1	2.550	O3	2.2429(12)	O6	2.267(3)	O5	2.2577
			O7	2.558					O6	2.2316
Amide O	O5	2.517(5)					O5	2.342(3)		
	O6	2.647(5)								
Ether O	O1	2.966(5)	O3	3.002	O1	2.9046(14)	O1	2.706(4)	O1	2.987
	O2	2.906(5)	O4	2.935	O2	2.8741(13)	O2	3.017(4)	O2	2.895
	O3	3.071(5)	O5	3.041			O3	2.881(3)	O3	2.754
	O4	2.892(5)	O6	2.921			O4	2.824(3)	O4	3.048
Amine N	N1	3.007(6)	N3	2.952	N1	2.8968(15)	N1	2.664(4)	N1	2.930
	N2	2.913(6)	N2	2.962			N2	2.932(5)	N3	2.688
N_pyr_–M–N_pyr_		80.98(17)		82.60		127.22(7)		127.23(14)		131.02
C–N–N–C^a^		32.70		33.26		-44.98		-69.73		-76.68
Conformation^b^	Δ(δλδ)(δλδ)		Δ(δλδ)(δλδ)		Δ(δδδ)(δδδ)		Δ(δλλ)(λδδ)		Δ(δλλ)(λδδ)	
NBO	s(99.46%)p(0.54%)		s(98.87%)p(1.12%)		s(97.47%)p(2.53%)		s(97.87%)p(2.13%)		s(97.10%)p(2.90%)	
ε_corr_ ^c^	0.139		0.149		0.181		0.167		0.189	
ε	0.118		0.127		0.160		0.163		0.185	

a Dihedral angle involving the methylenic carbon atoms of the pendant arms and the amine N atoms for a Δ configuration.

b The corresponding enantiomers are also present in the crystal lattice.

c Value of eccentricity corrected for the different radii of donor atoms.

**Figure 2:**
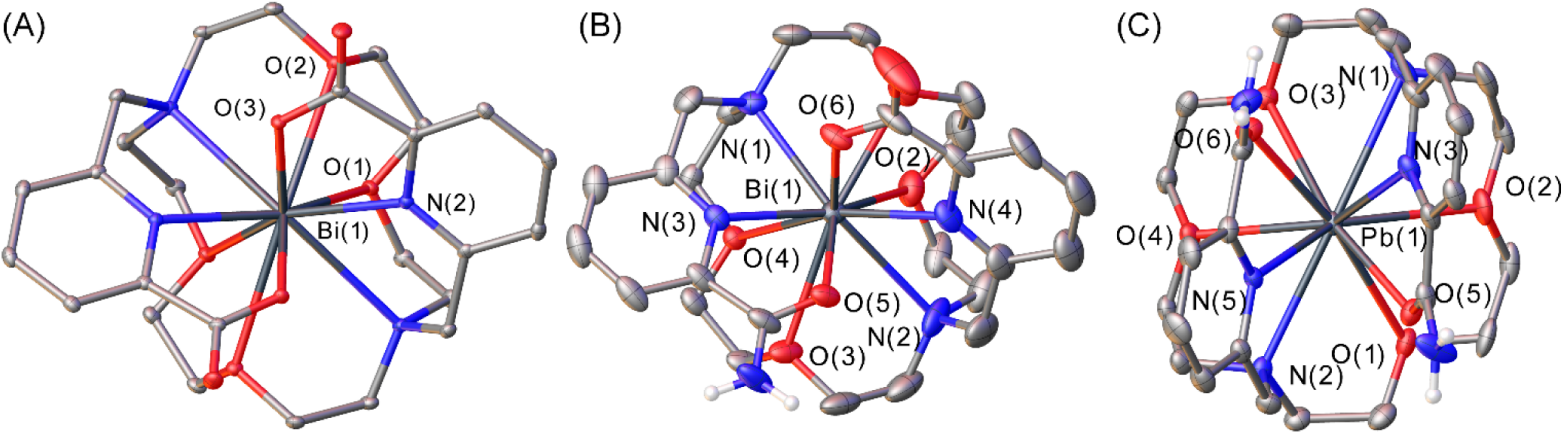
Top views of the X-ray crystal structures of [Bi­(macropa)]­PF_6_ (A), [Bi­(macropapam)]­(PF_6_)_2_·2H_2_O (B), and [Pb­(macropam)]­(PF_6_)_2_·H_2_O (C) with atom numbering. Hydrogen atoms bonded to carbon atoms, non-bonded water molecules and anions are omitted for simplicity. ORTEP plots are at 30% probability. The structure of [Bi­(macropa)]­PF_6_ is grown through symmetry.

The Pb­(II) complex of macropam crystallizes in the monoclinic *C*2*/c* space group, with the formula [Pb­(macropam)]·2PF_6_·H_2_O. As was described for the crystal structure of [Pb­(macropa)],^25^ the complex cation presents a distorted *C*_2_ symmetry with the Δ­(δλδ)­(δλδ) and Λ­(λδλ)­(λδλ) enantiomers present throughout the crystal structure in equal amounts. With respect to the bond lengths from the metal center to the ligand donor atoms, they are very similar to those reported for the macropa complex, with the bond distances to the picolinamide arms (2.517–2.647 Å) being shorter than those to the donor atoms of the macrocyclic cavity (2.892–3.071 Å). This is characteristic of a hemidirected structure, where the 6s^2^ lone pair of the Pb­(II) ion is pointing toward the crown ether, a fact that is additionally supported by the eccentricity value of 0.118, calculated using our previously reported methodology,^32^ and significant p-contribution to the lone pair in the NBO analysis, performed on the crystal structures with DFT-optimized positions of the H atoms (Table 1). Exchanging carboxylate donor atoms with primary amides does not seem to have a large effect on lone pair activity (though p character increases in the macropa complex), conformation of the complex, or bond lengths and angles in the crystallized Pb­(II) complexes.

The case presented by the Bi­(III) complexes is somewhat more complicated. In the crystal structure reported previously by Fiszbein et al., the Bi­(III) macropa complex presents a conformation that does not possess *C*_2_ symmetry, [Δ­(δλλ)­(λδδ)/Λ­(λλδ)­(δδλ)], which differs from the crystal structures of the Pb­(II) complexes, and is not consistent with the reported solution NMR spectra, showing effective *C*_2_ symmetry of the complex. However, the authors do allude to this disparity being due to fluxional behavior. This dynamic behavior in solution is further supported by the Bi­(III) complex crystal structures reported here. The Bi­(III) macropa complex reported here crystallizes in the monoclinic *C*2*/c* space group with the formula [Bi­(macropa)]·PF_6_·2H_2_O, although the asymmetric unit contains only one-half of this formula, giving the complex crystallographically imposed *C*_2_ symmetry with a Δ­(δδδ)­(δδδ)/Λ­(λλλ)­(λλλ) conformation. The bond distances are once again shorter for the donor atoms of the pendant arms (2.2429 Å for Bi–O_carboxylate_ and 2.4343 Å for Bi–N_pyridine_), than those of the donor atoms of the macrocycle (2.8741–2.9046 Å).

The macropapam Bi­(III) complex crystallizes in the triclinic P1̅ space group with the formula [Bi­(macropapam)]·2PF_6_·2H_2_O, though the water molecules are disordered throughout the crystal lattice. The conformation in this case is Δ­(δλλ)­(λδδ)/Λ­(λδδ)­(δλλ). The bond distances for the macrocyclic donor atoms closest to the carboxylate moiety are shorter (N1 and O1), while the opposite donor atoms have the longest bond-lengths (N2, O2, O3, and O4), likely due to the anionic charge of the carboxylate, favoring the lone pair to be directed in the opposite direction of the charged donor group. For all three Bi­(III) complexes, the pendant arms lay much flatter than in the Pb­(II) complexes, with N_pyr_–M–N_pyr_ bond angles of around 130° for the former and angles around 80° for the latter. The dihedral C–N–N–C angle corresponding to the C atoms of the methylene group of the pendant arms and the N atoms of the amines also characterizes this difference in arm configuration, with the Pb­(II) complexes adopting positive dihedral angles and the Bi­(III) complexes possessing negative values for the same angle (with a Δ configuration of the pendant arms). Although the use of eccentricity to determine lone pair activity was described exclusively for Pb­(II) complexes, we determined that due to the presence of an equivalent lone pair in Bi­(III) complexes, this analysis could be applied to the latter as well. Interestingly, these eccentricity values along with the p-character of the lone pair in each case are significantly larger for the Bi­(III) complexes than the Pb­(II) complexes, indicating larger lone pair activity for the Bi­(III) ion than the Pb­(II) ion.

The C–N and C–O distances of the amide group in [Pb­(macropam)]^2+^ [C(6)–N(6) = 1.324(9) Å, C(6)–O(6) = 1.252(8) Å; C(19)–N(4) = 1.300(10) Å, C(19)–O(5) = 1.242(8)] are very similar to those observed for the free ligand [H_2_macropam]^2+^ [C(13)–N(3) = 1.328(3) Å; C(13)–O(3) = 1.238(3) Å], though a slight elongation of the C–O bond is observed upon coordination, with concomitant shortening of the C–N bond. This effect is more pronounced in [Bi­(macropapam)]^2+^ [C(19)–N(5) = 1.262(7) Å; C(19)–O(5) = 1.272(7) Å], indicating that the C–O bond gains single character, while the C–N amide bond gains double character. This can be attributed to an increased contribution of the charge-separated resonance form for the amide group as the polarizing ability of the cation increases.^33^

### Solution Structure of the Pb­(II) and Bi­(III) Complexes

The Bi­(III) and Pb­(II) complexes were all prepared *in situ* in D_2_O, by adding 1.1 mol equiv of the corresponding metal nitrate to an acidic solution of the chelator and then adjusting the pH to near-neutral values to be the closest possible to physiological conditions. This was successful for the synthesis of most of the complexes with the exception of [Bi­(macropam)]^3+^, which was not completely formed at pH 5 and then further dissociated when the pH was increased to around 7, likely due to the competition with the formation of Bi­(III) hydroxide (Figure S3, Supporting Information). Overall, these experiments indicate the low stability of the complex formed with macropam, and though this could be confirmed through either potentiometric or spectrophotometric titrations, the low solubility of the ligand at higher pH values makes the determination of these constants quite difficult. The rest of the complexes were completely characterized by NMR (1D and 2D), MS, and UV–vis (see Supporting Information).

The UV–vis spectra of the complexes show maxima corresponding to the absorption of the pyridyl moieties of the picolinate or picolinamide groups at 272 nm for the Pb­(II) complexes (Figure S4, Supporting Information) and 274 nm for the Bi­(III) complexes (Figure S5, Supporting Information). The Bi­(III) complexes show an additional shoulder around 300 nm, that can be attributed to the 6sp ← 6s absorption band (ε = 2000 M^–1^·cm^–1^). The position of this band is shifted toward slightly lower energies for [Bi­(macropapam)]^2+^ in comparison with [Bi­(macropa)]^+^, as expected due to the higher degree of covalency coming from a bond formed with a softer amide oxygen atom.^34^

The NMR spectra were recorded to obtain additional structural information. ^1^H and ^13^C spectra were assigned when possible, using COSY, HMQC, and HMBC. The spectra of [Pb­(macropa)] and [Bi­(macropa]^+^ were previously reported and completely assigned in the case of the Pb­(II) complex (Tables S1 and S2). The new spectra match those which were reported in prior publications, though according our 2D NMR, the assignment of the H3 and H5 signals of [Pb­(macropa)] is reversed.^18,19,25^

For the Pb­(II) complexes, coordination of the metal ion results in the diastereotopic splitting of the ^1^H signals corresponding to the different −CH_2_– groups of the chelator (Figure 3). The splitting pattern of these signals, due to coordination of the ligand to the Pb­(II) ion, observable in all three cases, was extensively discussed by Ferreirós-Martínez et al. in the first publication on the [Pb­(macropa)] complex.^25^

**Figure 3:**
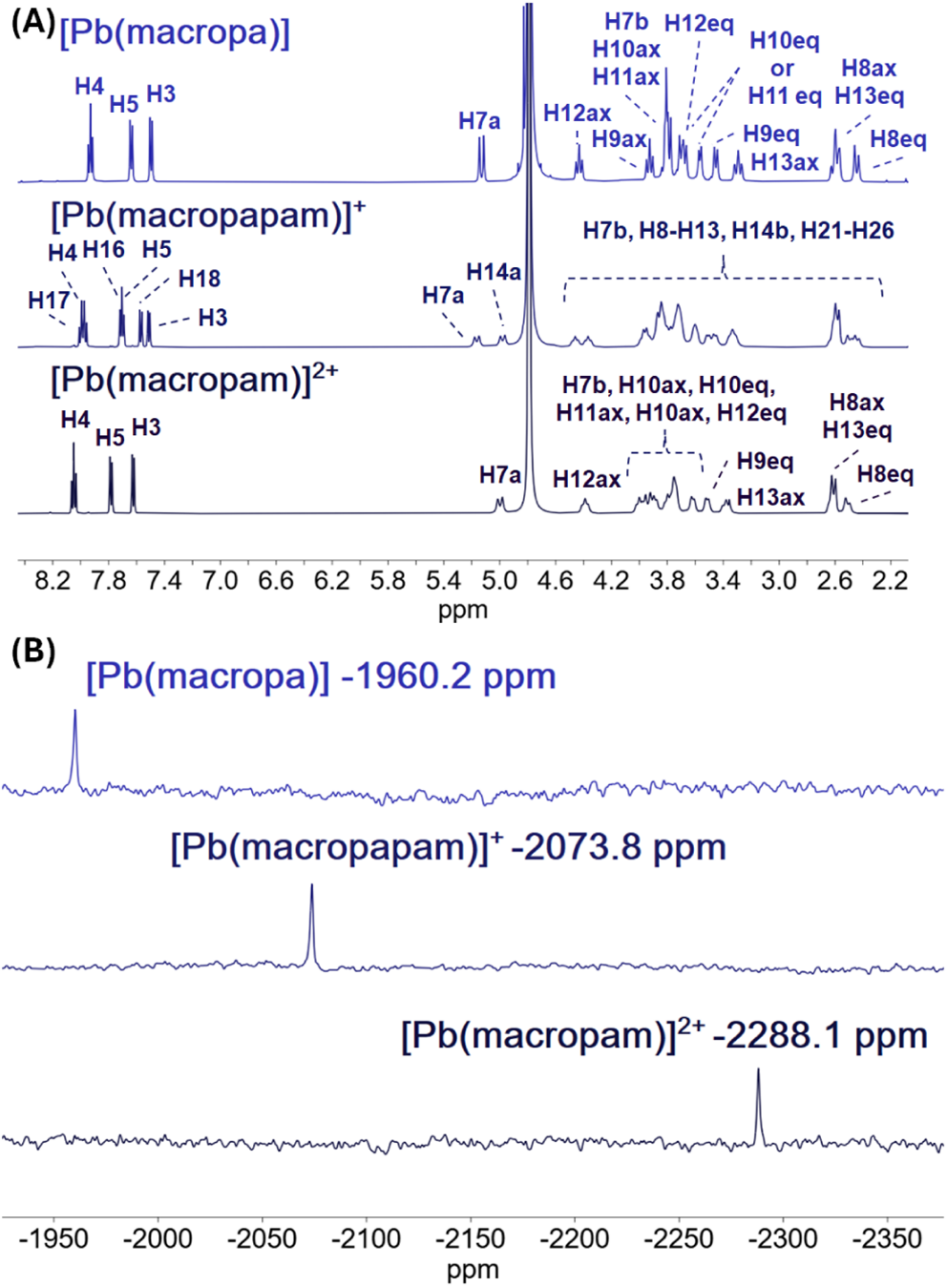
^1^H NMR (500 MHz, 298 K) (A) and ^207^Pb-NMR (84 MHz, 298 K) (B) of the Pb­(II) complexes recorded in D_2_O solution at pD near 7. The chemical shift values are referenced to Pb­(CH_3_)_4_ having a chemical shift of 0 ppm. See Chart [Fig chart1] for numbering scheme.

The Pb­(II) complexes of both macropa and macropam display *C*_2_ symmetry while the macropapam complex exhibits a loss of symmetry and additional splitting of the signals due to the pendant arms no longer being equivalent. Additionally, the proton signals of the amide-containing chelators are somewhat broader than those of [Pb­(macropa)]. We were also able to record the ^207^Pb NMR signals of the three complexes. These signals take on values ranging between −1960 and −2288 ppm. The values agree well with the signals reported for other macrocyclic chelators with high denticity, both structurally related crown ethers and thioethers,^19,35^ and other nonstructurally related decadentate ligands.^14,15^ As the number of amides in the structure increases, the ^207^Pb signal shifts to more negative values, an effect that was observed previously for EDTA derivatives,^36^ and PYTAM (2,2′,2″,2‴-(3,6,10,13-tetraaza-1,8­(2,6)-dipyridinacyclotetradecaphane-3,6,10,13-tetrayl)­tetraacetamide) and its carboxylate analogue PYTA (2,2′,2″,2‴-(3,6,10,13-tetraaza-1,8­(2,6)-dipyridinacyclotetradecaphane-3,6,10,13-tetrayl)­tetraacetic acid).^14,15^

The NMR spectra of [Bi­(macropa)]^+^ and [Bi­(macropapam)]^2+^ (Figure 4) share similar features to those of the Pb­(II) complexes. The main characteristics such as diastereotopic splitting and either *C*_2_ or *C*_1_ symmetry depending on the pendant arms are maintained, although the signals are somewhat broader for the protons of the macrocycle in the spectrum of [Bi­(macropa)]^+^. This broadening is indicative of a certain fluxionality of the complex, which was described previously,^18^ and is in agreement with having obtained crystal structures with different macrocycle conformations. The ^1^H NMR spectra of [Bi­(macropa)]^+^ recorded in MeOD at −70 °C exhibit extensive broadening of the aliphatic proton signals (Figure S6, Supporting Information). However, the slow exchange regime is not reached even at this temperature, and therefore it is not possible to calculate the activation parameters for the process.

### Computational Study of the Pb­(II) and Bi­(III) Complexes

To understand the dynamics in solution of this system, as well as the differences when compared to the Pb­(II) complexes, a series of computational studies were performed. In first place, the eight conformations of the complexes with a Δ helicity of the pendant arms and varying δ or λ conformations of the 5-membered chelates formed through coordination of the macrocycle with *C*_2_ symmetry (*C*_1_ in the case of the macropapam complexes) were optimized in aqueous solution. Another eight conformations with *C*_2_ symmetry and Λ helicity of the pendant arms exist. However, these eight conformations form enantiomeric pairs with the Δ conformations, giving them the same properties in a nonchiral environment. In Table 2 we report the free energies relative to the Δ­(δλδ)­(δλδ) isomer. For the Pb­(II) complexes, the most stable conformation is the Δ­(δλδ)­(δλδ) isomer, followed by the Δ­(λδλ)­(λδλ) isomer with a difference of +11.5 kJ·mol^–1^ for the macropa complex which grows as the carboxylates are substituted for amides, reaching a difference of +16.9 kJ·mol^–1^ for the macropam complex. This is consistent both with the crystal structures of the Pb­(II) complexes which display a single enantiomeric pair [Δ­(δλδ)­(δλδ)/Λ­(λδλ)­(λδλ)] enantiomeric pair in the solid state, as well as with the NMR spectra and the conformational study of macropa that was previously reported.^25^

**Table 2:** Relative Free Energies (kJ·mol^–1^) in Aqueous Solution (PCM) of the Conformations of the Complexes with *C*_2_ Symmetry (in the Case of the Macropapam Complexes There Is No *C*_2_ Symmetry, Although the Conformation of the Chelates Is Maintained)

	[Pb(macropa)]	[Pb(macropapam)]^+^	[Pb(macropam)]^2+^	[Bi(macropa)]^+^	[Bi(macropapam)]^2+^
Δ(λλλ)(λλλ)	+36.30	+35.20	+31.53	+13.26	+10.67
Δ(δδδ)(δδδ)	+29.71	+24.46	+19.24	+5.10	+5.93
Δ(δλλ)(δλλ)	+32.83	+28.71	+26.03	+17.45	+14.79
Δ(λδλ)(λδλ)	+11.50	+12.07	+16.92	–0.65	+7.19
Δ(λλδ)(λλδ)	+39.29	+39.96	+45.26	+42.24	+43.45
Δ(δδλ)(δδλ)	+28.34	+25.25	+25.38	+18.79	+15.20
Δ(λδδ)(λδδ)	+44.91	+46.09	+50.14	+40.57	+40.57
Δ(δλδ)(δλδ)	0.00	0.00	0.00	0.00	0.00

The Bi­(III) complexes, however, present a slightly more intricate situation. The same conformations of the Bi­(III) macropa and macropapam complexes were optimized, as the macropam complex did not form completely. The optimizations of the [Bi­(macropa)]^+^ complexes interestingly showed that the Δ­(λδλ)­(λδλ) isomer was more stable than the Δ­(δλδ)­(δλδ) isomer by a very small difference of −0.65 kJ·mol^–1^. The Δ­(λδλ)­(λδλ) isomer is also more stable for the lanthanides from Nd to Lu, though for these lanthanides other isomers such as Δ­(δδδ)­(δδδ) or Δ­(δδλ)­(δδλ) are closer in energy than the Δ­(δλδ)­(δλδ) isomer, which is favored in the case of La­(III) and Pb­(II) as well as the [Bi­(macropapam)]^2+^ complex.^30^ For both of the Bi­(III) complexes, the Δ­(δδδ)­(δδδ) conformation presents a relatively small difference in energy between 5 and 6 kJ·mol^–1^, and is in fact the conformation of [Bi­(macropa)]^+^ in the newly reported crystal structure. This led us to believe that there is a dynamic process occurring in solution which results in the broadening of NMR signals of the protons of the macrocycle in [Bi­(macropa)]^+^, as well as the crystallization of different isomers in the solid state. For [Bi­(macropapam)]^2+^ the Δ­(δλδ)­(δλδ) isomer is considerably more stable with respect to other conformations, which results in a somewhat more well-resolved NMR spectrum (Figure 4).

**Figure 4:**
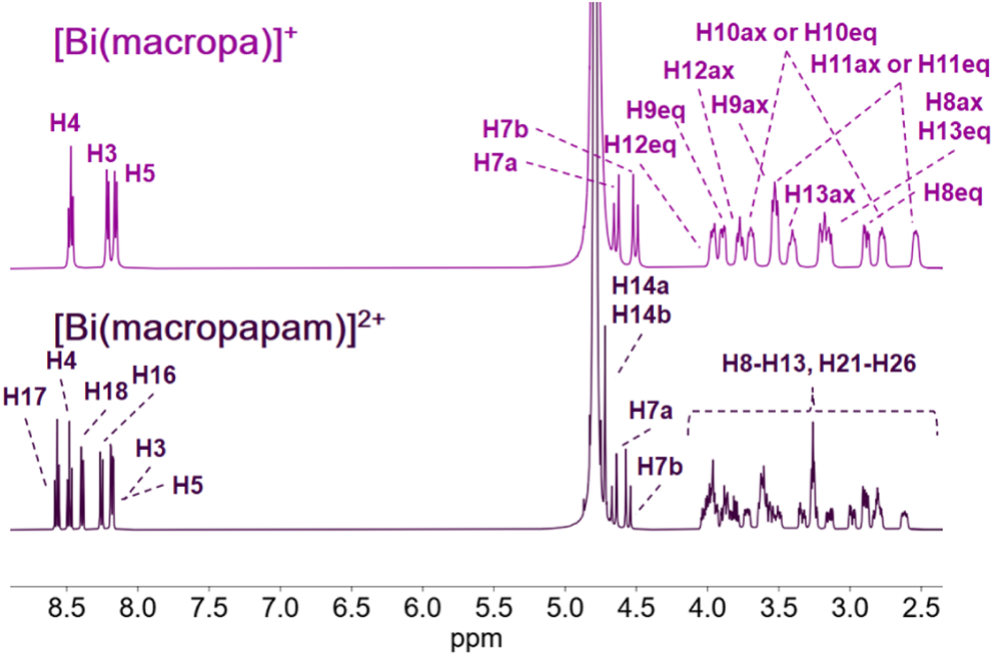
^1^H NMR (500 MHz, 298 K) of the Bi­(III) complexes recorded in D_2_O solution at pD near 7. See Chart [Fig chart1] for numbering scheme.

To further comprehend the fluxionality in [Bi­(macropa)]^+^, pathways between the most stable conformation with *C*_2_ symmetry as well as the conformations of the crystal structures were studied to determine if there were any possible pathways with relatively low energy. Transitioning from one conformation to another implies inverting the λ/δ conformation of one of the five-membered chelates of the macrocyclic backbone. To carry out this inversion, a series of potential energy surface (PES) scans were performed, and the corresponding transition states were optimized. Of note, some pathways led to further structural changes involving the conformation of the pendant arms besides the λ/δ conformational change and were not studied further. Moreover, there are 3 different types of chelates formed with the macrocycle, the −N–CH_2_–CH_2_–O–(A) groups that fall under the picolinate groups, the central −O–CH_2_–CH_2_–O–(B) groups, and the remaining-O–CH_2_–CH_2_–N–(C) groups (Figure 5A). To transition between the most stable Δ­(λδλ)­(λδλ) conformation and the crystal structures, inverting either 3 or 4 chelates is necessary and, in fact, two inversions are common to both pathways. An energy diagram representing the relative energies of the different conformations on the pathways as well as the transition states (TS) is presented in Figure 5B (see also Table S3). It should be mentioned that all of the intermediates and transition states studied mainly affect the chelate ring that is being inverted, with minimal effects on the rest of the macrocyclic structure, including bond lengths and bond angles, as can be observed in superimposed images of the different structures (Figure S7, Supporting Information). The first step involves the inversion of a C chelate, resulting in a structure in which the *C*_2_ symmetry is broken to give a Δ­(λδλ)­(λδδ) conformation. This step is followed by the inversion of the A chelate that is contiguous to the previously inverted C chelate giving a Δ­(δδλ)­(λδδ) conformation. These first two inversions possess transition states with reasonably low energies (37.2 kJ·mol^–1^ and 44.9 kJ·mol^–1^, respectively), that indicate that these processes could take place in solution, as dynamic processes in Bi­(III) complexes have been observed experimentally in solution with energies of around 60 kJ·mol^–1^.^37,38^ The Δ­(δδλ)­(λδδ) conformer is a common intermediate for the interconversion of the Δ­(λδλ)­(λδλ) isomer to both crystal structures. The pathway to crystal structure 1 (CS1, Δ­(δλλ)­(λδδ)) involves one last inversion of the B chelate contiguous to the A chelate that was inverted in the previous step. The transition state for this process has a low energy of just 27.7 kJ·mol^–1^. In relation to crystal structure 2 (CS2), two possible pathways were determined: in the first (dark blue in Figure 5B), the remaining A chelate was inverted followed by the remaining C chelate; and in the second (light blue in Figure 5B), the C chelate was initially inverted followed by the A chelate. The first pathway results in an intermediate (Δ­(δδλ)­(δδδ)) with much higher energy than any of the other conformations, but the transition state of the second inversion that leads to the final Δ­(δδδ)­(δδδ) conformation has a much lower energy barrier. The second pathway reverses the order of the inversions, with a Δ­(δδδ)­(λδδ) intermediate. While this conformer has a lower energy than the Δ­(δδλ)­(δδδ) conformation of the first pathway, the TS has a relative energy of over 50 kJ·mol^–1^, making it so the first pathway may be more likely.

**Figure 5:**
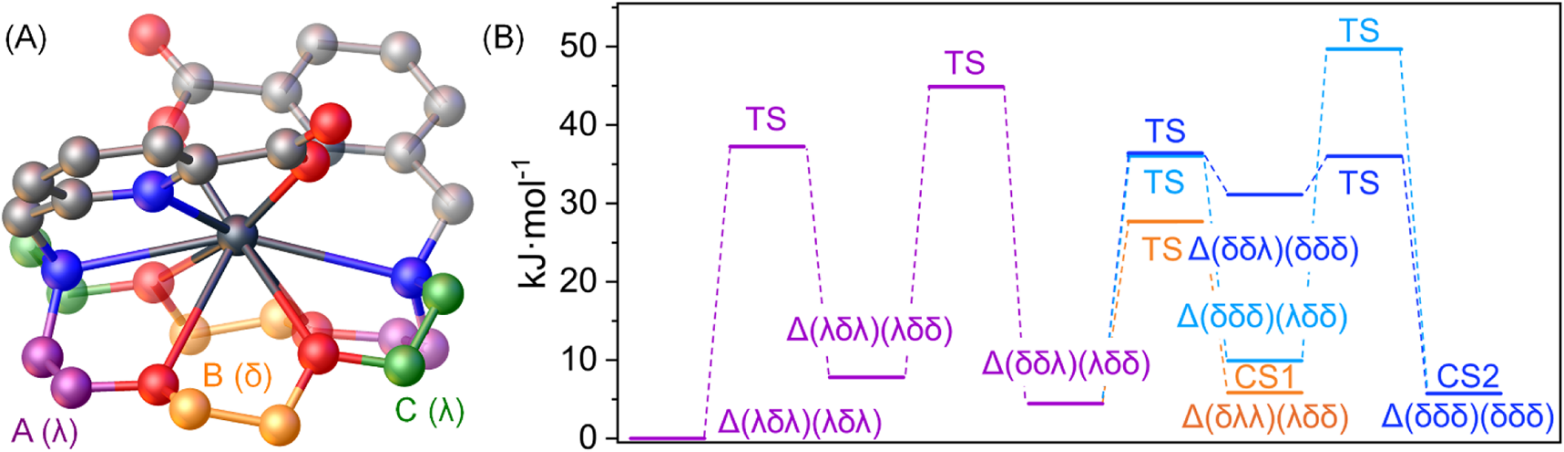
Optimized Δ­(λδλ)­(λδλ) geometry highlighting the −CH_2_– groups characterizing the A, B, and C chelates (A). Relative energies in kJ·mol^–1^ of the conformations of [Bi­(macropa)]^+^ found along the pathways from the most stable conformation to crystal structure 1 (CS1) and crystal structure 2 (CS2) as well as the relative energies of the transition states characterizing each chelate inversion (B).

The energies of the transition states responsible for the interconversion between the most stable isomer (Δ­(λδλ)­(λδλ)) and those observed in the X-ray structures (CS1 and CS2) are <50 kJ·mol^–1^. This rather low activation free energy explains the fluxional behavior of [Bi­(macropa)]^+^, which appears to involve changes in the conformation of the macrocycle leading to several isomers with relatively low energy differences. As mentioned previously, considerably higher activation free energies (>60 kJ·mol^–1^) were determined for the inversion of the macrocyclic unit in DOTA derivatives of Bi­(III) and the lanthanide ions.^37–39^ Once the energies of the transition states were determined, the Eyring equation was used to estimate the rate of the process.^40^ Considering an activation energy of 50 kJ·mol^–1^, which is higher than all our calculated transition energies shown in Figure 5, at 298 K, the process can be estimated to occur on the μs time scale, with smaller activation barriers having even shorter lifetimes (see Supporting Information for additional details). This is consistent with the NMR data, as we could not observe distinct peaks that could be assigned to each conformation. Furthermore, the four energy minima are expected to have significant populations in solution, and therefore, it is not surprising that the crystal structures of two diastereoisomers (CS1 and CS2) were obtained.

### Radiolabeling Studies

Radiolabeling experiments with [^203^Pb]­Pb­(II) were performed at room temperature in 0.1 M ammonium acetate buffer (pH 7), using macropa, macropapam, macropam, and the commercial standard DOTAM as a reference. Radiochemical conversions (%RCC)^41^ were determined using iTLC after 1 h across ligand concentrations ranging from 10^–4^ to 10^–9^ M.

Concentration-dependent radiolabeling trends are shown in Figure 6 and Table S4, Supporting Information. Both macropa and macropapam achieved quantitative or >90% incorporation of [^203^Pb]­Pb­(II) at the higher ligand concentrations. For macropa, radiolabeling remained quantitative from 10^–4^ to 10^–6^ M (%RCC = > 99% at all three concentrations), followed by a gradual decline at lower chelator concentrations: 84 ± 4% at 10^–7^ M, 9 ± 5% at 10^–8^ M, and 3 ± 4% at 10^–9^ M. Macropapam similarly produced full incorporation at 10^–4^ M, with high yields at 10^–5^ M (96 ± 3%) and 10^–6^ M (90 ± 2%). A sharper decrease was observed at 10^–7^ M (11 ± 1%) and 10^–8^ M (2 ± 0%). Macropam exhibited weaker performance under identical conditions. Although it reached 65 ± 12% RCC at 10^–4^ M, measurable radiolabeling was not detected at lower concentrations in this study, indicating a substantially reduced affinity for Pb­(II) compared to the other macropa-derived ligands. This trend that is observed with the macropa derivatives is consistent with the incorporation of amides provoking a decrease in thermodynamic stability.^42^ DOTAM, used as a benchmark chelator, showed high complexation efficiency at between concentrations 10^–4^ and 10^–6^ M (> 99% RCC at all three concentrations). However, its performance decreased more rapidly than that of macropa at lower chelator levels: 37 ± 7% at 10^–7^ M and 5 ± 0% at 10^–8^ M. No labeling was observed at 10^–9^ M.

**Figure 6:**
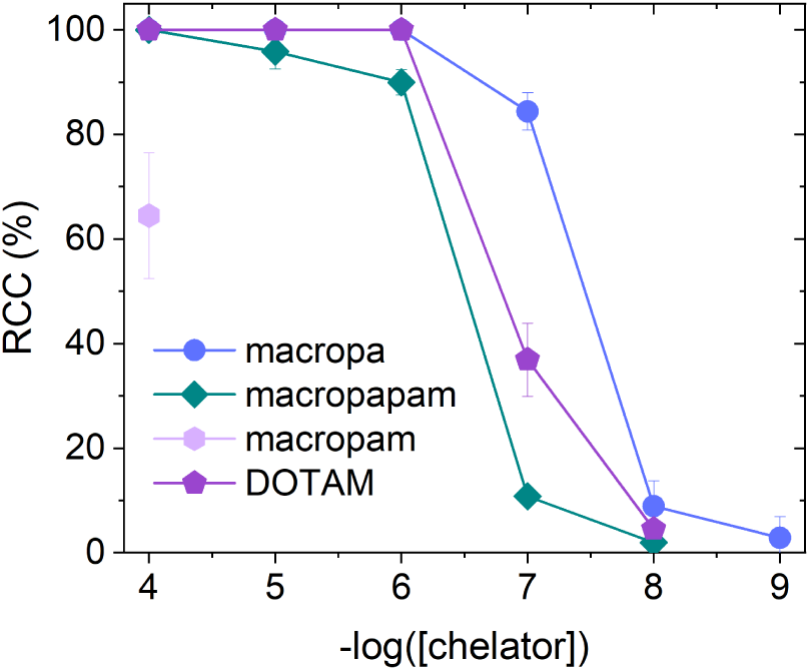
[^203^Pb]­Pb­(II) concentration-dependent radiolabeling at room temperature after 60 min (1.35 MBq, 0.1 M NH_4_OAc buffer, pH 7, 100 μL total reaction volume, *n* = 3 for each data point).

Radiolabeling with [^213^Bi]­Bi­(III) was performed at room temperature in 0.5 M MES (2-(*N*-morpholino)­ethanesulfonic acid) buffer (pH 5.5), using the same chelators along with DOTA as a benchmark chelator and DOTAM since it is the amide-containing analogue of DOTA. In this case, the radiochemical conversions (%RCC) were determined after 5 min for ligand concentrations ranging from 10^–4^ to 10^–8^ M. The only chelator that was labeled above >95% under these conditions at a concentration of 10^–4^ M was macropa, with all remaining chelators displaying RCCs under 20% (Figure 7 and Table S5, Supporting Information). As discussed previously, the [Bi­(macropam)]^3+^ complex was not completely formed at pH 5 according to the NMR spectra, making the poor radiolabeling consistent with this reduced performance. Additionally, it seems that even the incorporation of one amide group, in the case of macropapam, is sufficient to drastically impact radiochemical yields at the studied pH, time, and temperature conditions.

**Figure 7:**
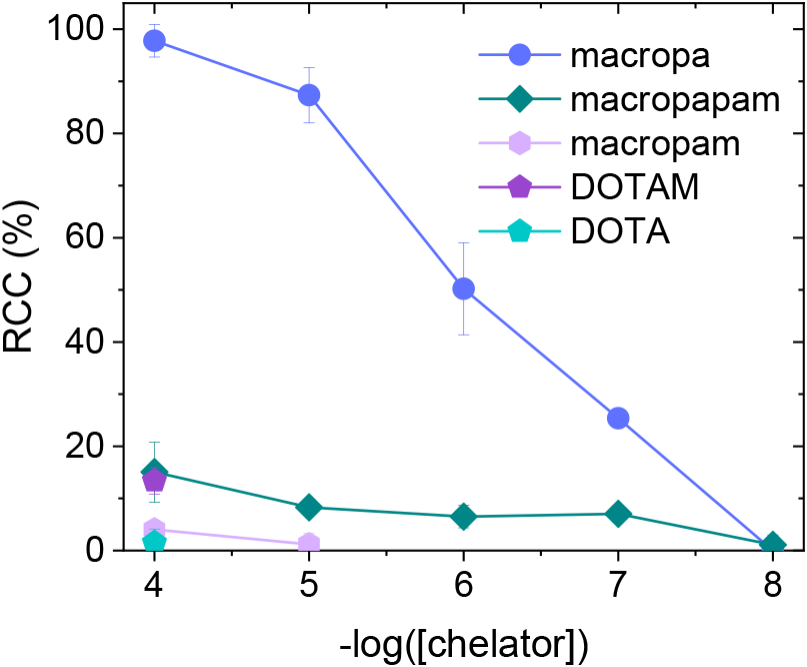
[^213^Bi]­Bi­(III) concentration-dependent radiolabeling at room temperature after 5 min (∼50–60 kBq, 0.5 M MES buffer, pH 5.5, 50 μL total reaction volume, *n* = 2 for each data point).

Overall, the data demonstrate that macropa maintains the highest labeling efficiency at trace concentrations of [^203^Pb]­Pb­(II) and [^213^Bi]­Bi­(III), outperforming both the commercial standard DOTAM (as well as DOTA in the case of [^213^Bi]­Bi­(III)) and the two modified derivatives. Macropapam shows strong labeling behavior with [^203^Pb]­Pb­(II) at moderate concentrations but loses efficiency more sharply at the submicromolar level and is not labeled by [^213^Bi]­Bi­(III). Macropam, despite containing softer donor functionalities, exhibits markedly diminished complexation capacity under these conditions. These results align with the expected influence of ligand donor environment on complex stability for intermediate (borderline) metal ions such as Pb­(II).

### Kinetic Inertness Studies of the Radiocomplexes with EDTA and Stable Metal Ions

The kinetic inertness of the new [^203^Pb]­Pb­(II)-labeled complexes was investigated by challenging the radiocomplexes with different competing agents at physiological conditions (pH 7, 37 °C): 20 equiv of the competitive chelator EDTA, 20 equiv of stable Pb­(II) ions, or a cocktail containing 10 equiv each of competing stable metal ions (ZnCl_2_, FeCl_3_, CuCl_2_, MgCl_2_, and CoCl_2_), with respect to chelator concentration. Complex stability was monitored by iTLC over 144 h (EDTA and stable metal challenges) and 72 h (Pb­(II) challenge). The inertness of macropam was not studied as it did not achieve quantitative labeling at any concentration. Additionally, no kinetic inertness experiments were performed with the [^213^Bi]­Bi­(III)-labeled complexes, as the newly investigated chelators macropapam and macropam were not successfully labeled under the tested conditions.

In the presence of excess EDTA (Figure 8A and Table S6, Supporting Information), DOTAM maintained quantitative integrity (100%) throughout 144 h, consistent with its established kinetic inertness.^15^ Macropa and macropapam complexes showed progressive dissociation: macropa retained 84% intact complex at 31 h, dropping to 12% by 144 h, while macropapam dissociated more rapidly, decreasing to 53% intact at 2 h and only 9% at 24 h. These findings indicate that the macropa-based ligands, although effective for initial radiolabeling, exhibit lower kinetic inertness against a competing ligand when compared to DOTAM.

**Figure 8:**
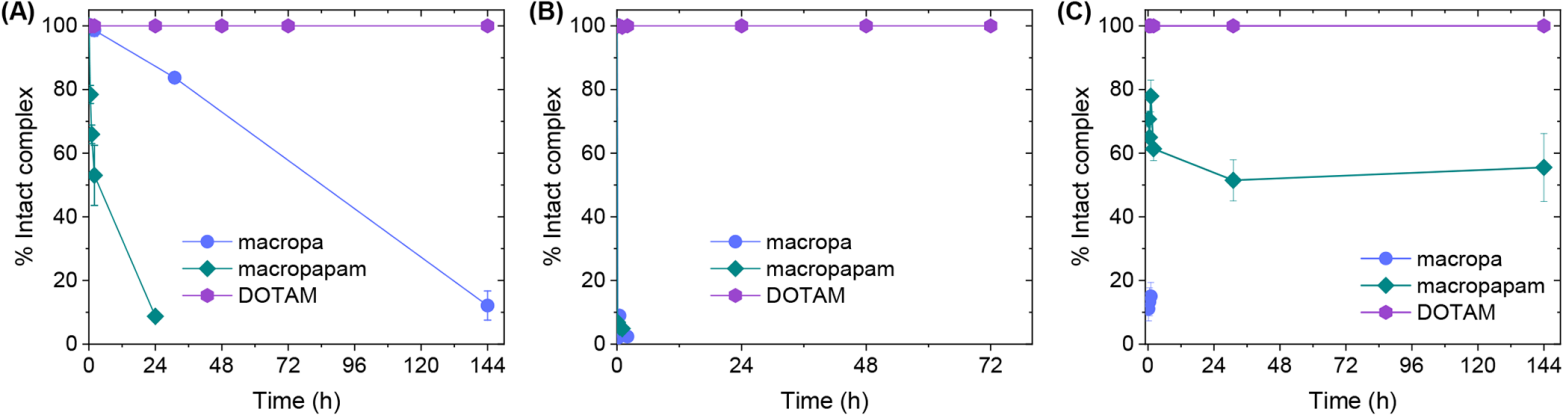
Kinetic inertness studies of the [^203^Pb]­Pb­(II)-labeled complexes (*n* = 3 for each data point). (A) Excess EDTA challenge (20 equiv). (B) Excess stable Pb­(II) challenge (20 equiv). (C) Stable metal cocktail challenge (10 equiv each of ZnCl_2_, FeCl_3_, CuCl_2_, MgCl_2_, and CoCl_2_).

When challenged with 20 equiv of nonradioactive Pb­(II) (Figure 8B and Table S7, Supporting Information), DOTAM again exhibited outstanding inertness with nearly 100% intact complex over 72 h. Macropa showed a nearly complete loss of complex (2 ± 1% intact) after 5 min, reflecting rapid dissociation or ligand exchange under these conditions. Macropapam retained low but measurable complexation (∼5–6%) up to 2 h but similarly demonstrated minimal kinetic inertness.

To further assess the kinetic stability of the Pb^2+^ complexes, we challenged the [^203^Pb]­Pb­(II)-labeled macropa, macropapam, and DOTAM complexes with a stable metal cocktail (Figure 8C and Table S8, Supporting Information). DOTAM once again exhibited exceptional kinetic inertness, maintaining 100% intact complex throughout the entire 144-h period. In contrast, macropapam demonstrated moderate stability, retaining on average of 56% intact complex at 144 h, with a gradual decrease observed from 78% at 1 h to 52% at 31 h, which was maintained throughout the rest of the study. Macropa showed substantially lower stability, with only around 15% intact complex detected at 1 h.

Together, these data suggest that while macropa derivatives provide efficient and high-yield radiolabeling with [^203^Pb]­Pb­(II), their complexes have significantly lower kinetic inertness in the presence of competing ligands or excess metal ions than DOTAM. Additionally, the incorporation of softer amide groups does not help with increasing kinetic inertness as was observed for other chelators.^14,15^ This highlights the importance of continued ligand optimization to enhance in vivo stability, particularly for prolonged circulation or therapeutic applications.

### Serum Stability Studies of the Radiocomplexes

To assess the kinetic inertness of the Pb­(II)–chelate complexes under biologically relevant conditions, the [^203^Pb]­Pb­(II)-labeled macropa, macropapam, and DOTAM complexes were incubated in human serum (90 μL of radiolabeling reaction and 90 μL of human serum) at 37 °C and monitored over 72 h using iTLC to quantify the percentage of intact complex (Table 3).

**Table 3:** In Vitro Human Serum Stability (Intact Complex, %) of [^203^Pb]­Pb­(II)-Labeled Complexes at 37 °C

Time (h)	Macropa	Macropapam	DOTAM
1	100 ± 0	100 ± 0	100 ± 0
2	100 ± 0	100 ± 0	100 ± 0
24	100 ± 0	100 ± 0	100 ± 0
48	100 ± 0	100 ± 0	100 ± 0
72	100 ± 0	100 ± 0	100 ± 0

Both macropa and the amide-modified derivative macropapam demonstrated exceptional stability in serum, with no detectable decomposition or transchelation over the entire study period. Each complex remained fully intact (100% intact complex) at all measured time points (1, 2, 24, 48, and 72 h). The commercial standard DOTAM exhibited an identical stability profile, likewise maintaining >99% integrity throughout the incubation. Macropapam, despite its reduced radiolabeling performance under some conditions, also showed complete resistance to serum-mediated degradation at the time points assessed, indicating that once formed, the Pb–Macropam complex is kinetically robust.

These results confirm that macropa, macropapam, and DOTAM form Pb­(II) complexes that remain stable in the presence of endogenous serum proteins. The excellent serum stability of the macropa derivative macropapam, together with the radiolabeling performance, could support its continued evaluation as chelators for ^203^Pb/^212^Pb-based radiopharmaceutical development.

## Conclusions

In this work, we have studied how the systematic incorporation of picolinamide pendants into the macropa scaffold affects radiolabeling and radiocomplex stability for potential use in Pb­(II)- and Bi­(III)-based radiopharmaceuticals. The substitution of the carboxylate groups for amides results in significantly worse RCCs and stabilities, which contrasts with the widespread idea that amides are more adequate donor groups for Pb­(II) than carboxylates. The stability of the [^203^Pb]­Pb­(II)-labeled complexes varies between macropa and macropapam depending on the competitor, being outperformed in all cases by gold-standard chelator DOTAM. However, the radiocomplexes do remain intact in human serum, indicating that they may still be applied in future studies. Additionally, we have provided a comprehensive examination of the coordination chemistry of macropa, macropapam, and macropam with Pb­(II) and Bi­(III). Detailed computational studies were able to justify the fluxional behavior of the Bi­(III) macropa complex in solution, providing essential understanding of the properties of this complex.

## Experimental Section

### General Considerations

Solvents and reagents were used as supplied by commercial sources. NaOH (99.99% trace metal grade), 2-(*N*-morpholino)­ethanesulfonic acid (MES), ethylenediaminetetraacetic acid (EDTA), and ammonium acetate (NH_4_OAc) (ACS grade) were purchased from Fisher Scientific. 1,4,7,10-Tetrakis­(carbamoylmethyl)-1,4,7,10-tetraazacyclododecane (DOTAM, aka TCMC) and 1,4,7,10-tetraazacyclododecane-1,4,7,10-tetraacetic acid (DOTA) were purchased from Macrocyclics (Plano, TX). 6-(Hydroxymethyl)­picolinamide was purchased from BLDPharm. Mass spectra were recorded on an LTQ-Orbitrap Discovery mass spectrometer coupled to a Thermo Accela HPLC in ESI positive mode. A Puriflash XS 420 InterChim Chromatographer equipped with a UV-DAD detector and a 20 g BGB Aquarius C18AQ reverse-phase column (100 Å, spherical, 15 μm), using H_2_O and CH_3_CN + 0.1% TFA as mobile phases (flow rate 15 mL/min) was used for purifications. Lyophilization was carried out in a Biobase BK-FD10 Series Vacuum Freeze-Dryer. Semipreparative HPLC was carried out using a Agilent 1100 series HPLC module using a Phenomenex Luna C18 (250 mm × 100 mm) column at 4.5 mL/min with the following method: A: H_2_O + 0.1% TFA, B: CH_3_CN + 0.1% TFA; 0–0.5 min 0% B; 0.5–8.5 min 0–40% B; 8.5–9.5 min 40% B. NMR spectra were recorded on Bruker AVANCE III 300, Bruker AVANCE 400, or Bruker AVANCE 500 spectrometers. Elemental analyses were performed on a ThermoFisher FlashSmart Elemental Analyzer. Macropa was prepared according to a previously reported procedure.^30^

#### 6-(Chloromethyl)­picolinamide

6-(Hydroxymethyl)­picolinamide (331.7 mg, 2.05 mmol, 1 equiv) was dissolved in SOCl_2_ (2.5 mL, 34.83 mmol, 17 equiv) at 0 °C under Ar. The reaction was left stirring for 1.5 h after which most of the SOCl_2_ is evaporated on a vacuum line, using trap with a saturated KOH solution. The remaining solvent was quenched using 25 mL of a saturated NaHCO_3_ solution, resulting in a suspension which was then extracted using CHCl_3_ (3 × 30 mL). The combined organic phases are dried over Na_2_SO_4_ and evaporated using a rotary evaporator resulting in a white solid (145.9 mg, yield = 42%). ^1^H NMR (300 MHz, CDCl_3_) δ 8.16 (d, 1H), 7.91 (t, 1H), 7.83 (s, br, 1H), 7.64 (d, 1H), 5.74 (s, br, 1H), 4.69 (s, 2H). This reaction was not optimized.

#### 6,6′-((1,4,10,13-Tetraoxa-7,16-diazacyclooctadecane-7,16-diyl)­bis­(methylene))­dipicolinamide (Macropam)

1,7,10,16-Tetraoxa-4,13-diazacyclooctadecane (83.2 mg, 0.317 mmol, 1 equiv) was dissolved in 3 mL of CH_3_CN and K_2_CO_3_ (89.7 mg, 0.649 mmol, 2 equiv) was added. 6-(Chloromethyl)­picolinamide (110.4 mg, 0.634 mmol, 2 equiv) was dissolved in 2 mL of acetonitrile and added dropwise. The reaction mixture was then refluxed for 48 h after which the solvent was evaporated, and the resulting orange oil was dissolved in 2 mL of 9:1 H_2_O:CH_3_CN + 0.1% TFA and purified by flash chromatography on a C18AQ column using H_2_O + 0.1% TFA as the eluent. The compound eluted at 1.74 CV and the fraction of interest was lyophilized obtaining a white solid (115.2 mg, yield = 68%). Alternatively mobile phase containing formic acid (FA) in place of TFA can be used. ^1^H NMR (400 MHz, D_2_O, pD ∼ 3, 342.8 K) δ 8.61 (t, 2H), 8.56 (d, 2H), 8.21 (d, 1H), 5.25 (s, 4H), 4.41 (t, 8H), 4.17 (t, 8H), 4.10 (s, 8H). ^13^C NMR (101 MHz, D_2_O, pD ∼ 3, 342.8 K) δ 168.87, 149.59, 149.03, 140.68, 127.47, 123.48, 70.47, 64.29, 57.71, 55.04. Experimental HR-MS (ESI^+^, %BPI): *m*/*z* 531.2927; calculated for [C_26_H_39_N_6_O_6_]^+^ 531.2926. For radiolabeling, the chelator was repurified using semipreparative HPLC to ensure high purity. Retention time 6.3 min. Elemental analysis calcd (%) for C_26_H_38_N_6_O_6_·2.8TFA·1.2CH_3_CN: C 45.42, H 4.98, N 11.22; found: C 45.51, H 4.63, N 11.12.

#### 6-((16-((6-Carbamoylpyridin-2-yl)­methyl)-1,4,10,13-tetraoxa-7,16-diazacyclooctadecan-7-yl)­methyl)­picolinic acid (Macropapam)

Macropapam, previously named Hppa18c6, was prepared according to the previously reported procedure, without any modifications.^29^ However, to ensure sufficient purity for radiolabeling reactions, as well as the synthesis of the metal complexes, the ligand was repurified using semipreparative HPLC. Retention time 6.4 min. Elemental analysis calcd (%) for C_26_H_37_N_5_O_7_·3TFA·1.2CH_3_CN·4.5H_2_O: C 41.15, H 5.28, N 8.65; found: C 41.19, H 5.33, N 8.45.

### Synthesis of the Pb­(II) and Bi­(III) Complexes

#### Pb­(II) Complexes

Macropa (6.4 mg, 0.012 mmol), macropapam (13.6 mg, 0.026 mmol), or macropam (6.4 mg, 0.012 mmol) was dissolved in D_2_O (0.6 mL). pD was adjusted to ∼4 using a DNO_3_ solution. Pb­(NO_3_)_2_ (4.3 mg, 0.013 mmol for macropa; 9.2 mg, 0.028 mmol for macropapam; and 4.6 mg, 0.014 mmol for macropam) was added, and the pD was adjusted to ∼6–7 using a NaOD solution.

#### [Pb­(macropa)]

^1^H NMR (500 MHz, D_2_O, pD ∼ 6) δ 7.93 (t, *J* = 7.7 Hz, 2H), 7.64 (d, *J* = 7.8 Hz, 2H), 7.50 (d, *J* = 7.6 Hz, 2H), 5.13 (d, *J* = 15.6 Hz, 2H), 4.43 (t, *J* = 10.8 Hz, 2H), 3.93 (t, *J* = 10.8 Hz, 2H), 3.87–3.76 (m, 6H), 3.75–3.64 (m, 4H), 3.57 (d, *J* = 9.2 Hz, 2H), 3.45 (d, *J* = 10.8 Hz, 2H), 3.29 (t, *J* = 11.6 Hz, 2H), 2.59 (m, 4H), 2.45 (d, *J* = 14.3 Hz, 2H). ^13^C NMR (126 MHz, D_2_O, pD ≈ 6) δ 171.96, 159.05, 149.64, 139.90, 127.01, 123.18, 70.04, 69.44, 68.37, 67.14, 59.33, 54.65, 53.51. Experimental HR-MS (ESI^+^, %BPI): *m*/*z* 761.2047 (100), 739.2229 (79); calculated for [C_26_H_34_N_4_NaO_8_Pb]^+^ 761.2035, calculated for [C_26_H_35_N_4_O_8_Pb]^+^ 739.2216.

#### [Pb­(macropapam)]^+^

^1^H NMR (500 MHz, D_2_O, pD ∼ 6) δ 7.98 (dt, *J* = 10.4, 7.9 Hz, 2H), 7.70 (t, *J* = 7.2 Hz, 2H), 7.57 (d, *J* = 7.8 Hz, 1H), 7.51 (d, *J* = 7.6 Hz, 1H), 5.16 (d, *J* = 15.1 Hz, 1H), 4.98 (d, *J* = 16.0 Hz, 1H), 4.46 (t, 1H), 4.37 (t, 1H), 4.01–3.44 (m, 18H), 3.38–3.29 (m, 2H), 2.65–2.55 (m, 4H), 2.53–2.41 (m, 2H). ^13^C NMR (126 MHz, D_2_O, pD ≈ 6) δ 172.33, 168.94, 160.59, 158.82, 149.68, 146.82, 140.07, 139.99, 128.07, 127.33, 123.44, 121.74, 70.11, 70.03, 69.44, 69.39, 68.58, 68.18, 67.18, 67.09, 59.27, 58.93, 54.84, 54.66, 53.40, 53.37. Experimental HR-MS (ESI^+^, %BPI): *m*/*z* 738.2387 (100); calculated for [C_26_H_36_N_5_O_7_Pb]^+^ 738.2376.

#### [Pb­(macropam)]^2+^

^1^H NMR (500 MHz, D_2_O, pD ∼ 7) δ 8.05 (t, *J* = 7.8 Hz, 2H), 7.78 (d, *J* = 7.9 Hz, 2H), 7.62 (d, *J* = 7.6 Hz, 2H), 5.00 (d, *J* = 16.3 Hz, 2H), 4.39 (t, 2H), 4.07–3.84 (m, 6H), 3.84–3.70 (m, 6H), 3.62 (d, *J* = 9.3 Hz, 2H), 3.51 (d, *J* = 9.5 Hz, 2H), 3.38 (t, *J* = 12.0 Hz, 2H), 2.61 (d, *J* = 12.5 Hz, 4H), 2.51 (d, *J* = 13.6 Hz, 2H). ^13^C NMR (126 MHz, D_2_O, pD ≈ 7) δ 169.18, 160.53, 146.49, 140.18, 128.45, 122.06, 70.09, 69.38, 68.40, 67.14, 58.87, 54.90, 53.31. Experimental HR-MS (ESI^+^, %BPI): *m*/*z* 737.2545 (100); calculated for [C_26_H_37_N_6_O_6_Pb]^+^ 737.2536.

#### Bi­(III) Complexes

Macropa (4.3 mg, 0.008 mmol) or macropapam (4.3 mg, 0.008 mmol) was dissolved in D_2_O (0.6 mL). pD was adjusted to ∼2 by using a DNO_3_ solution. Bi­(NO_3_)_3_·5H_2_O (4.3 mg, 0.009 mmol for macropa; 5.3 mg, 0.011 mmol for macropapam) was added. pD was adjusted to ∼6 using a NaOD solution.

#### [Bi­(macropa)]^+^

^1^H NMR (500 MHz, D_2_O, pD ∼ 6) δ 8.47 (t, *J* = 7.8 Hz, 2H), 8.21 (d, *J* = 7.6 Hz, 2H), 8.16 (d, *J* = 7.9 Hz, 2H), 4.64 (d, *J* = 16.3 Hz, 2H), 4.51 (d, *J* = 16.4 Hz, 2H), 3.96 (m, 2H), 3.89 (m, 2H), 3.77 (m, 2H), 3.70 (m, 2H), 3.53 (m, 4H), 3.41 (m, 2H), 3.17 (m, 4H), 2.89 (m, 2H), 2.77 (m, 2H), 2.54 (m, 2H). ^13^C NMR (126 MHz, D_2_O, pD ≈ 6) δ 170.26, 158.35, 147.96, 143.10, 127.82, 125.86, 68.66, 68.21, 67.40, 64.70, 61.26, 55.64, 54.94. Experimental HR-MS (ESI^+^, %BPI): *m*/*z* 739.2186 (100); calculated for [C_26_H_34_BiN_4_O_8_]^+^ 739.2175.

#### [Bi­(macropapam)]^2+^

^1^H NMR (500 MHz, D_2_O, pD ∼ 6) δ 8.57 (t, *J* = 7.9 Hz, 1H), 8.48 (t, *J* = 7.8 Hz, 1H), 8.39 (dd, *J* = 7.9, 0.9 Hz, 1H), 8.26 (d, *J* = 7.9 Hz, 1H), 8.18 (dd, *J* = 7.8, 2.7 Hz, 2H), 4.71 (s, 4H), 4.66 (d, *J* = 16.4 Hz, 1H), 4.56 (d, *J* = 16.5 Hz, 1H), 4.05–3.93 (m, 4H), 3.91–3.76 (m, 1H), 3.72 (ddd, *J* = 11.7, 6.3, 2.9 Hz, 1H), 3.65–3.47 (m, 5H), 3.39–3.30 (m, 1H), 3.25 (q, *J* = 6.5 Hz, 3H), 3.14 (ddd, *J* = 13.9, 7.4, 2.6 Hz, 1H), 2.98 (ddd, *J* = 13.8, 5.5, 2.3 Hz, 1H), 2.93–2.85 (m, 2H), 2.80 (ddt, *J* = 14.2, 8.7, 3.3 Hz, 2H), 2.62 (ddd, *J* = 11.7, 6.5, 3.1 Hz, 1H). ^13^C NMR (126 MHz, D_2_O, pD ∼ 6) δ 170.36, 169.23, 160.82, 158.50, 147.84, 145.72, 143.21, 143.16, 128.91, 128.23, 126.11, 124.69, 69.22, 68.69, 68.52, 68.17, 67.76, 67.47, 64.89, 64.86, 61.25, 61.01, 56.00, 55.46, 55.05, 54.93. Experimental HR-MS (ESI^+^, %BPI): *m*/*z* 738.2346 (100), 369.6205 (49); calculated for [C_26_H_35_BiN_5_O_7_]^+^ 738.2335, calculated for [C_26_H_36_BiN_5_O_7_]^2+^ 369.6204.

### Crystal Structure Determination

Crystallization of ([H_2_macropam]­(PF_6_)_2_·H_2_O, [Bi­(macropa)]­PF_6_ and [Pb­(macropam)]­(PF_6_)_2_·H_2_O) and ([Bi­(macropapam)]­(PF_6_)_2_·2H_2_O) was achieved by adding a drop of a saturated solution of KPF_6_ to an aqueous solution of the compound and allowing for slow evaporation of the solvent. Crystallographic data and the structure refinement parameters corresponding to [H_2_macropam]­(PF_6_)_2_·H_2_O, [Bi­(macropa)]­PF_6_, [Bi­(macropapam)]­(PF_6_)_2_·2H_2_O, and [Pb­(macropam)]­(PF_6_)_2_·H_2_O are given in Table S9, Supporting Information. Crystallographic data were collected at 100 K using a Bruker D8 Venture diffractometer with a Photon 100 CMOS detector and Mo–Kα radiation (λ = 0.71073 Å) generated by an Incoatec high-brilliance microfocus source equipped with Incoatec Helios multilayer optics. The software APEX4 ^43^ ([H_2_macropam]­(PF_6_)_2_·H_2_O, [Bi­(macropa)]­PF_6_, and [Pb­(macropam)]­(PF_6_)_2_·H_2_O) or APEX5 ^44^ ([Bi­(macropapam)]­(PF_6_)_2_·2H_2_O) was used for collecting frames of data, indexing reflections, and the determination of lattice parameters, SAINT^45^ for integration of intensity of reflections, and SADABS^46^ for scaling and empirical absorption correction. SHELXT program^47^ was used for solving the structure by dual-space methods. All non-hydrogen atoms were refined with anisotropic thermal parameters by full-matrix least-squares calculations on F^2^ using the program SHELXL-2014.^48^ Hydrogen atoms of the compound were inserted at calculated positions and constrained with isotropic thermal parameters. CCDC 2489852–2489855 contains the supplementary crystallographic data, which can be obtained free of charge from the Cambridge Crystallographic Data Centre via www.ccdc.ac.uk/data_request/cif.

### Computational Details

All the reported calculations were carried out using Gaussian 16,^49^ employing the TPSSh functional.^50^ Relativistic effects were incorporated through the ECP60MDF small-core relativistic effective core potential, for both Pb­(II) and Bi­(III).^51^ The selected ECP, with 60 electrons in the core, was combined with the corresponding cc-pVTZ basis set, presenting a (12s11p8d1f)/[5s4p3d1f] contraction scheme for Pb­(II) and Bi­(III).^52^ For all other atoms, the Def2-TZVPP basis set was selected.^53^ For the geometry optimization of the different conformations of the Pb­(II) and Bi­(III) complexes, the optimized geometries of the La­(III) macropa complex, which were previously reported,^30^ were used as a starting point and frequency calculations were used to confirm that the optimized structures were indeed stationary points and to obtain zero point energy corrections and thermal contributions. A polarized continuum model (PCM) was used to incorporate bulk solvent effects with the default Gaussian-implemented parameters.^54,55^ The integral = ultrafine keyword was used to set the integration grid. For NBO analysis, the positions of the H atoms of the crystal structures were first optimized by fixing the position of the rest of the atoms, the NBO calculation was then run on the resulting geometry using the NBO program,^56^ included in Gaussian. Potential energy surface (PES) scans were performed using the opt = modredundant keyword, specifying the involved −X–CH_2_–CH_2_–X (X = N or O) dihedral angle in 13 steps of 10°. This was done to find starting geometries for the transition states and to the most favorable pathway. For only these calculations, the smaller Def2-SVP basis set was used.^53^ Transition states were located using the synchronous transit-guided quasi-Newton method,^57^ using the opt = qst3 keyword, with frequency calculations affording a single imaginary frequency. Sample input files along with the optimized geometries are included in the Supporting Information.

### Radiochemistry

**Caution!** Experiments carried out with radioactive isotopes that emit ionizing radiation such as [^203^Pb]­Pb­(II) and [^213^Bi]­Bi­(III) should only be performed by trained personnel at facilities equipped to safely handle and store these isotopes.

[^203^Pb]­Pb­(II) and [^213^Bi]­Bi­(III) were produced and isolated as previously reported, receiving the radiometals as [^203^Pb]­PbCl_2_ in 0.01 M HCl and [^213^Bi]­BiI_4_^–^/[^213^Bi]­BiI_5_^2–^ in a 0.2 M NaI solution made up in 0.1 M HCl.^18,58^ Stock solutions with a concentration of 10^–3^ M of all the tested chelators were prepared in deionized water. Serial dilution was then performed to obtain additional stock solutions with concentrations between 10^–4^ M and 10^–8^ M. Deionized water was obtained from a Millipore Direct-Q 3UV water purification system. Instant thin-layer chromatography paper impregnated with silicic acid (iTLC-SA) was purchased from Agilent Technologies (Santa Clara, CA). These plates were used to monitor the reactions and were developed using EDTA (50 mM, pH 5.5). Under these conditions, the chelated radiometal (either [^203^Pb]­Pb­(II) or [^213^Bi]­Bi­(III)) remains at the baseline (*R*_f_ = 0) while the free radiometal migrates with the solvent front. Radiochemical conversions (RCCs) were determined by iTLC, using a BioScan AR-2000 imaging scanner equipped with P-10 gas and the BioScan WinScan V3_14 software. Human serum from human male AB plasma, USA origin, sterile-filtered was purchased from Sigma-Aldrich.

#### [^203^Pb]­Pb­(II) Radiolabeling Studies

A total reaction volume of 100 μL was used, composed of 10 μL of chelator (or water as a negative control), 80 μL of deionized water, and 10 μL of activity diluted in 1 M NH_4_OAc (pH = 7, 1.35 MBq). The reactions were gently mixed with a vortex and were reacted at ambient temperature, performing each reaction in triplicate. Five μL of the reactions were spotted at 60 min to determine the RCCs.

#### [^213^Bi]­Bi­(III) Radiolabeling Studies

A total reaction volume of 50 μL was used, composed of 5 μL of chelator (or water as a negative control), 40 μL of 0.5 M MES buffer (pH = 5), and 5 μL of activity (∼50–60 kBq). The reactions were carried out within 5 min of the generator elution at ambient temperature, performing each reaction in duplicate. Seven μL aliquots of the reactions were spotted at 5 min to determine the RCCs.

### EDTA, Pb­(II), and Stable Metal Cocktail Challenges

Stock solutions of EDTA (pH = 7) and Pb­(OAc)_2_ were prepared with a concentration of 20 mM, while the stable metal cocktail was prepared using different metals (ZnCl_2_, FeCl_3_, CuCl_2_, MgCl_2_, and CoCl_2_) at a 10 mM concentration. Ten μL of the competitor stock was added to the preformed [^203^Pb]­Pb­(II)-complexes (10^–4^ M chelator concentration, 100 μL volume). Aliquots (5 μL) were taken at different time points and spotted onto the iTLC-SA plates. Each reaction was performed in triplicate.

### Human Serum Stability

90 μL of the human serum was added to the preformed [^203^Pb]­Pb­(II)-complexes (10^–4^ M chelator concentration, 90 μL volume), which were then incubated at 37 °C. Aliquots (5 μL) were taken at different time points and spotted onto the iTLC-SA plates. Each reaction was performed in triplicate.

## Supplementary Material



## Notes

Supplemental figures, UV–vis spectra, NMR, MS, radiochemical data, crystal data and refinement details, computational input files, and optimized geometries (PDF)
