# Supplementary material for: Evaluating the Incorporation of Picolinamide Pendants into the Macropa Scaffold for Pb(II)- and Bi(III)-Based Radiopharmaceuticals
Source: Inorg Chem. 2026 Jun 22;65(26):15150–63. doi: 10.1021/acs.inorgchem.6c02096 (PMC13343464; doi:10.1021/acs.inorgchem.6c02096)
Supplement: Supplementary file 1 [file ic6c02096_si_001.pdf]

# Supporting information for:

# Evaluating the incorporation of picolinamide pendants into the macropa scaffold for Pb(II)- and Bi(III)-based radiopharmaceuticals

Charlene Harriswangler,*[†,‡,§] Parmissa Randhawa,[‡,§] Nicolás Sommariva-Ucha,[†] Luke Wharton,[§] Laura Valencia,[||] Brooke L. McNeil,[‡,§] Hua Yang,[‡,§,⊥] David Esteban-Gómez,[†] Carlos Platas-Iglesias,[†] and Caterina F. Ramogida.*[‡,§]

[†] CICA - Centro Interdisciplinar de Química e Bioloxía and Departamento de Química, Universidade da Coruña, Facultade de Ciencias, 15071, A Coruña, Galicia, Spain.

[‡] Department of Chemistry, Simon Fraser University, 8888 University Drive, Burnaby, BC, V5A 1S6, Canada

[§] Life Sciences Division, TRIUMF, 4004 Wesbrook Mall, Vancouver, BC, V6T 2A3, Canada

[||] Departamento de Química Inorgánica, Facultad de Ciencias, Universidade de Vigo, As Lagoas, Marcosende, 36310 Pontevedra, Spain.

[⊥] Department of Chemistry, University of British Columbia, 2036 Main Mall, Vancouver, BC, V6T 1Z1, Canada

*E-mail: charlene.harriswangler@udc.es
*E-mail: cfr@sfu.ca

# Contents

**Figure S1**: (A) Three [$H_2$macropam]$^+$ units connected through hydrogen bonding in an $R_2^2(8)$ motif, dotted lines represent hydrogen bonding. (B) Close-up of the $R_2^2(8)$ motif. The ORTEP plot is at 50%. .............. 8

**Figure S2**: Sources of helicity in complexes of macropa-based systems where the picolinate arms adopt a *syn* conformation represented through DFT optimized structures and ChemDraw Schemes. ........................... 8

**Figure S3**: $^1$H-NMR (300 MHz, 298 K) of the attempt to prepare [Bi(macropam)]$^{3+}$ at pH 5 (bottom) and pH 7 (top). pH measured with pH paper. The proportion of the signals corresponding to the free ligand (aromatic signals between 7.6 and 8.2 ppm) becomes larger upon increasing the pH. ............................................... 9

**Figure S4**: UV-Vis spectra of the Pb(II) complexes ($5 \times 10^{-5}$ M), showing a maximum at 272 nm corresponding to the picolinate groups. ........................................................................................ 9

**Figure S5**: UV-Vis spectra of the Bi(III) complexes ($5 \times 10^{-5}$ M), showing a maximum at 274 nm corresponding to the picolinate groups and a shoulder around 300 nm corresponding to the 6sp ← 6s transition of Bi(III). ........................................................................................................ 9

**Table S1**: $^1$H-NMR chemical shifts (ppm) and assignment for the Pb(II) and Bi(III) complexes discussed in the manuscript (500 MHz, 298 K, $D_2O$). ........................................................................ 10

**Table S2**: $^{13}$C-NMR chemical shifts (ppm) and assignment for the Pb(II) and Bi(III) complexes discussed in the manuscript (126 MHz, 298 K, $D_2O$). ........................................................................ 11

**Figure S6**: $^1$H-NMR (400 MHz, 203.15 K, MeOD) of [Bi(macropa)]$^+$ showing broadening of the signals of the aliphatic region. ........................................................................................................ 12

**Table S3**: Relative Free Energies (kJ/mol) in Aqueous Solution (PCM) of the Conformations of the Bimacropa Complex on the Pathways Between the Most Stable Conformation Δ(λδλ)(λδλ) and the Conformations of the two Crystal Structures, along with the Transition States (TS). ............................... 12

**Figure S7**: Superimposed structures of different conformations of [Bi(macropa)]$^+$ illustrating that the most important differences between conformations and transition states involve the chelate ring being inverted. X denotes an eclipsed conformation of the 5-membered chelate ring in the transition states. Three different views are included for each set of three structures: a front view of the 5 membered chelate ring, a top view of the complex and a side view of the 5-membered chelate ring. .................................................................. 13

**Estimation of the rate constant for the λ/δ inversion.** ........................................................................ 14

**Table S4**: Radiochemical yields (RCYs, %) for the [$^{203}$Pb]Pb(II) radiolabeling reactions after 60 minutes (n=3), room temperature ........................................................................................................ 14

**Table S5**: Radiochemical yields (RCYs, %) for the [$^{213}$Bi]Bi(III) radiolabeling reactions after 5 minutes (n=2), room temperature. ....................................................................................................... 14

**Table S6**: Stability of the $^{203}$Pb-labeled chelators (intact complex, %) in a 20-fold excess EDTA (n=3). 14

**Table S7**: Stability of the $^{203}$Pb-labeled chelators (intact complex, %) in a 20-fold excess Pb(II)(n=3). .. 15

**Scheme S1**: Structure of **6-(chloromethyl)picolinamide** ........................................................... 16

**Figure S8**: $^1$H NMR spectrum of 6-(chloromethyl)picolinamide (300 MHz, $CDCl_3$, 298 K). .................. 16

**Scheme S2**: Structure of **macropam**. ........................................................................................... 17

**Figure S9**: $^1$H NMR spectrum of **macropam** (400 MHz, $D_2O$, pD ~ 3, 342.8 K). .................................. 17

**Figure S10**: $^{13}$C NMR spectrum of **macropam** (126 MHz, $D_2O$, pD= 7, 298 K). .................................... 18

**Figure S11**: Experimental high resolution mass spectrum (ESI$^+$) of compound **macropam**. ................... 18

**Scheme S3**: Structure of **[Pb(macropa)]** ........................................................................................ 19

**Figure S12**: $^1$H NMR spectrum of complex **[Pb(macropa)]** (500 MHz, $D_2O$, pD = 6, 298 K). ............... 19

**Figure S13**: $^{13}C$ NMR spectrum of complex **[Pb(macropa)]** (126 MHz, $D_2O$, pD = 6, 298 K). ..............20

**Figure S14**: $^{13}C$ DEPT-135 NMR spectrum of complex **[Pb(macropa)]** (126 MHz, $D_2O$, pD = 6, 298 K).20

**Figure S15**: $^{1}H$-$^{1}H$ COSY NMR spectrum of complex **[Pb(macropa)]** (500 MHz, $D_2O$, pD = 6.5, 298 K). ..........21

**Figure S16**: $^{1}H$-$^{13}C$ HSQC NMR spectrum of complex **[Pb(macropa)]** (500-127 MHz, $D_2O$, pD = 6, 298 K). ..........21

**Figure S17**: $^{1}H$-$^{13}C$ HMBC NMR spectrum of complex **[Pb(macropa)]** (500-127 MHz, $D_2O$, pD = 6, 298 K). ..........22

**Figure S18**: $^{1}H$-$^{207}Pb$ HMQC NMR spectrum of complex **[Pb(macropa)]** (400-84 MHz, $D_2O$, pD = 6, 298 K)..........22

**Figure S19**: Experimental high resolution mass spectrum ($ESI^+$) of compound **[Pb(macropa)]** .............23

**Scheme S4**: Structure of **[Pb(macropapam)]$^+$**..........24

**Figure S20**: $^{1}H$ NMR spectrum of complex **[Pb(macropapam)]$^+$** (500 MHz, $D_2O$, pD = 6, 298 K). ......24

**Figure S21**: $^{13}C$ NMR spectrum of complex **[Pb(macropapam)]$^+$** (126 MHz, $D_2O$, pD = 6, 298 K). TFA indicates trifluoroacetic acid..........25

**Figure S22**: $^{13}C$ DEPT-135 NMR spectrum of complex **[Pb(macropapam)]$^+$** (126 MHz, $D_2O$, pD = 6, 298 K)..........25

**Figure S23**: $^{1}H$-$^{1}H$ COSY NMR spectrum of complex **[Pb(macropapam)]$^+$** (500 MHz, $D_2O$, pD = 6, 298 K). ..........26

**Figure S24**: $^{1}H$-$^{13}C$ HSQC NMR spectrum of complex **[Pb(macropapam)]$^+$** (500-126 MHz, $D_2O$, pD = 6, 298 K)..........26

**Figure S25**: $^{1}H$-$^{13}C$ HMBC NMR spectrum of complex **[Pb(macropapam)]$^+$** (500-126 MHz, $D_2O$, pD = 6, 298 K)..........27

**Figure S26**: $^{1}H$-$^{207}Pb$ HMQC NMR spectrum of complex **[Pb(macropapam)]$^+$** (400-84 MHz, $D_2O$, pD = 6, 298 K)..........27

**Figure S27**: Experimental high resolution mass spectrum ($ESI^+$) of compound **[Pb(macropapam)]$^+$**.....28

**Scheme S5**: Structure of [Pb(macropam)]$^{2+}$..........29

**Figure S28**: $^{1}H$ NMR spectrum of complex **[Pb(macropam)]$^{2+}$** (500 MHz, $D_2O$, pD = 7, 298 K). FA indicates formic acid. ..........29

**Figure S29**: $^{13}C$ NMR spectrum of complex **[Pb(macropam)]$^{2+}$** (126 MHz, $D_2O$, pD = 7, 298 K). FA indicates formic acid. ..........30

**Figure S30**: $^{13}C$ DEPT-135 NMR spectrum of complex **[Pb(macropam)]$^{2+}$** (126 MHz, $D_2O$, pD = 7, 298 K). Asterisk corresponds to a formic acid signal. ..........30

**Figure S31**: $^{1}H$-$^{1}H$ COSY NMR spectrum of complex **[Pb(macropam)]$^{2+}$** (500 MHz, $D_2O$, pD= 7, 298 K). ..........31

**Figure S32**: $^{1}H$-$^{13}C$ HSQC NMR spectrum of complex **[Pb(macropam)]$^{2+}$** (500-126 MHz, $D_2O$, pD = 7, 298 K)..........31

**Figure S33**: $^{1}H$-$^{13}C$ HMBC NMR spectrum of complex **[Pb(macropam)]$^{2+}$** (500-126 MHz, $D_2O$, pD = 7, 298 K)..........32

**Figure S34**: $^{1}H$-$^{207}Pb$ HMQC NMR spectrum of complex **[Pb(macropam)]$^{2+}$** (400-84 MHz, $D_2O$, pD = 7, 298 K)..........32

**Figure S35**: Experimental high resolution mass spectrum ($ESI^+$) of compound **$[Pb(macropam)]^{2+}$**. This spectrum displays signals corresponding to the free ligand as well, though in the NMR spectra there is no evidence of other species, indicating that this is likely a process occurring in the MS. ............................33

**Scheme S6**: Structure of $[Bi(macropa)]^+$ ....................................................................................................34

**Figure S36**: $^1H$ NMR spectrum of complex **$[Bi(macropa)]^+$** (500 MHz, $D_2O$, pD = 6, 298 K)................34

**Figure S37**: $^{13}C$ NMR spectrum of complex **$[Bi(macropa)]^+$** (126 MHz, $D_2O$, pD = 6, 298 K). TFA indicates trifluoroacetic acid.........................................................................................................................35

**Figure S38**: $^{13}C$ DEPT-135 NMR spectrum of complex **$[Bi(macropa)]^+$** (126 MHz, $D_2O$, pD = 6, 298 K).35

**Figure S39**: $^1H$-$^1H$ COSY NMR spectrum of complex **$[Bi(macropa)]^+$** (500 MHz, $D_2O$, pD = 6, 298 K).36

**Figure S40**: $^1H$-$^{13}C$ HSQC NMR spectrum of complex **$[Bi(macropa)]^+$** (500-126 MHz, $D_2O$, pD = 6, 298 K). ..........................................................................................................................................36

**Figure S41**: $^1H$-$^{13}C$ HMBC NMR spectrum of complex **$[Bi(macropa)]^+$** (500-126 MHz, $D_2O$, pD = 6, 298 K)..........................................................................................................................................37

**Figure S42**: Experimental high resolution mass spectrum ($ESI^+$) of compound **$[Bi(macropa)]^+$**.............37

**Scheme S7**: Structure of $[Bi(macropapam)]^{2+}$ ..........................................................................................38

**Figure S43**: $^1H$ NMR spectrum of complex **$[Bi(macropapam)]^{2+}$** (500 MHz, $D_2O$, pD = 6, 298 K).......38

**Figure S44**: $^{13}C$ NMR spectrum of complex **$[Bi(macropapam)]^{2+}$** (126 MHz, $D_2O$, pD = 6, 298 K)......39

**Figure S45**: $^{13}C$ DEPT-135 NMR spectrum of complex **$[Bi(macropapam)]^{2+}$** (126 MHz, $D_2O$, pD = 6, 298 K)..........................................................................................................................................39

**Figure S46**: $^1H$-$^1H$ COSY NMR spectrum of complex **$[Bi(macropapam)]^{2+}$** (500 MHz, $D_2O$, pD = 6, 298 K). ..........................................................................................................................................40

**Figure S47**: $^1H$-$^{13}C$ HSQC NMR spectrum of complex **$[Bi(macropapam)]^{2+}$** (500-126 MHz, $D_2O$, pD = 6, 298 K)......................................................................................................................................40

**Figure S48**: $^1H$-$^{13}C$ HMBC NMR spectrum of complex **$[Bi(macropapam)]^{2+}$** (500-126 MHz, $D_2O$, pD = 6, 298 K)......................................................................................................................................41

**Figure S49**: Experimental high resolution mass spectrum ($ESI^+$) of compound **$[Bi(macropapam)]^+$**. ....41

**Table S9**: Crystal data and structure refinement for $[H_2macropam](PF_6)_2 \cdot H_2O$, $[Bi(macropa)]PF_6$, $[Bi(macropapam)](PF_6)_2 \cdot 2H_2O$ and $[Pb(macropam)](PF_6)_2 \cdot H_2O$. ..............................................................42

**Example of a Gaussian Input File for Optimization of a Pb(II) Complex**..........................................43

**Example of a Gaussian Input File for Optimization of a Bi(III) Complex**..........................................47

**Example of a Gaussian Input File for Optimization of Only Hydrogen Atoms**.................................51

**Example of a Gaussian Input File for an NBO Calculation**.................................................................55

**Example of a Gaussian Input File for a PES scan** ................................................................................59

**Example of a Gaussian Input File for a Transition State Optimization** .............................................63

**Table S10**: Cartesian coordinates (Å) of **$[Pb(macropam)]^{2+}$** from the obtained crystal structure with optimized hydrogen positions obtained with DFT calculations. ...............................................................69

**Table S11**: Cartesian coordinates (Å) of **[Pb(macropa)]** from the previously reported crystal structure with optimized hydrogen positions obtained with DFT calculations. ...............................................................71

**Table S12**: Cartesian coordinates (Å) of **$[Bi(macropa)]^+$** from the obtained crystal structure with optimized hydrogen positions obtained with DFT calculations. .................................................................................73

**Table S13**: Cartesian coordinates (Å) of **[Bi(macropapam)]$^{2+}$** from the obtained crystal structure with optimized hydrogen positions obtained with DFT calculations. ........ 75

**Table S14**: Cartesian coordinates (Å) of **[Bi(macropa)]$^{+}$** from the previously reported crystal structure with optimized hydrogen positions obtained with DFT calculations. ........ 77

**Table S15**: Cartesian coordinates (Å) of the Δ(λλλ)(λλλ) conformer of **[Pb(macropa)]** from geometry optimizations (0 imaginary frequencies). ........ 79

**Table S16**: Cartesian coordinates (Å) of the Δ(δδδ)(δδδ) conformer of **[Pb(macropa)]** from geometry optimizations (0 imaginary frequencies). ........ 81

**Table S17**: Cartesian coordinates (Å) of the Δ(δλλ)(δλλ) conformer of **[Pb(macropa)]** from geometry optimizations (0 imaginary frequencies). ........ 83

**Table S18**: Cartesian coordinates (Å) of the Δ(λδλ)(λδλ) conformer of **[Pb(macropa)]** from geometry optimizations (0 imaginary frequencies). ........ 85

**Table S19**: Cartesian coordinates (Å) of the Δ(λλδ)(λλδ) conformer of **[Pb(macropa)]** from geometry optimizations (0 imaginary frequencies). ........ 87

**Table S20**: Cartesian coordinates (Å) of the Δ(δδλ)(δδλ) conformer of **[Pb(macropa)]** from geometry optimizations (0 imaginary frequencies). ........ 89

**Table S21**: Cartesian coordinates (Å) of the Δ(λδδ)(λδδ) conformer of **[Pb(macropa)]** from geometry optimizations (0 imaginary frequencies). ........ 91

**Table S22**: Cartesian coordinates (Å) of the Δ(δλδ)(δλδ) conformer of **[Pb(macropa)]** from geometry optimizations (0 imaginary frequencies). ........ 93

**Table S23**: Cartesian coordinates (Å) of the Δ(λλλ)(λλλ) conformer of **[Pb(macropapam)]$^{+}$** from geometry optimizations (0 imaginary frequencies). ........ 95

**Table S24**: Cartesian coordinates (Å) of the Δ(δδδ)(δδδ) conformer of **[Pb(macropapam)]$^{+}$** from geometry optimizations (0 imaginary frequencies). ........ 97

**Table S25**: Cartesian coordinates (Å) of the Δ(δλλ)(δλλ) conformer of **[Pb(macropapam)]$^{+}$** from geometry optimizations (0 imaginary frequencies). ........ 99

**Table S26**: Cartesian coordinates (Å) of the Δ(λδλ)(λδλ) conformer of **[Pb(macropapam)]$^{+}$**from geometry optimizations (0 imaginary frequencies). ........ 101

**Table S27**: Cartesian coordinates (Å) of the Δ(λλδ)(λλδ) conformer of **[Pb(macropapam)]$^{+}$** from geometry optimizations (0 imaginary frequencies). ........ 103

**Table S28**: Cartesian coordinates (Å) of the Δ(δδλ)(δδλ) conformer of **[Pb(macropapam)]$^{+}$** from geometry optimizations (0 imaginary frequencies). ........ 105

**Table S29**: Cartesian coordinates (Å) of the Δ(λδδ)(λδδ) conformer of **[Pb(macropapam)]$^{+}$** from geometry optimizations (0 imaginary frequencies). ........ 107

**Table S30**: Cartesian coordinates (Å) of the Δ(δλδ)(δλδ) conformer of **[Pb(macropapam)]$^{+}$** from geometry optimizations (0 imaginary frequencies). ........ 109

**Table S31**: Cartesian coordinates (Å) of the Δ(λλλ)(λλλ) conformer of **[Pb(macropam)]$^{2+}$** from geometry optimizations (0 imaginary frequencies). ........ 111

**Table S32**: Cartesian coordinates (Å) of the Δ(δδδ)(δδδ) conformer of **[Pb(macropam)]$^{2+}$** from geometry optimizations (0 imaginary frequencies). ........ 113

**Table S33**: Cartesian coordinates (Å) of the Δ(δλλ)(δλλ) conformer of **[Pb(macropam)]$^{2+}$** from geometry optimizations (0 imaginary frequencies). ........ 115

**Table S34**: Cartesian coordinates (Å) of the Δ(λδλ)(λδλ) conformer of **[Pb(macropam)]$^{2+}$** from geometry optimizations (0 imaginary frequencies). ........ 117

**Table S35**: Cartesian coordinates (Å) of the Δ(λλδ)(λλδ) conformer of **[Pb(macropam)]$^{2+}$** from geometry optimizations (0 imaginary frequencies). .......... 119

**Table S36**: Cartesian coordinates (Å) of the Δ(δδλ)(δδλ) conformer of **[Pb(macropam)]$^{2+}$** from geometry optimizations (0 imaginary frequencies). .......... 121

**Table S37**: Cartesian coordinates (Å) of the Δ(λδδ)(λδδ) conformer of **[Pb(macropam)]$^{2+}$** from geometry optimizations (0 imaginary frequencies). .......... 123

**Table S38**: Cartesian coordinates (Å) of the Δ(δλδ)(δλδ) conformer of **[Pb(macropam)]$^{2+}$** from geometry optimizations (0 imaginary frequencies). .......... 125

**Table S39**: Cartesian coordinates (Å) of the Δ(λλλ)(λλλ) conformer of **[Bi(macropa)]$^{+}$** from geometry optimizations (0 imaginary frequencies). .......... 127

**Table S40**: Cartesian coordinates (Å) of the Δ(δδδ)(δδδ) conformer of **[Bi(macropa)]$^{+}$** from geometry optimizations (0 imaginary frequencies). .......... 129

**Table S41**: Cartesian coordinates (Å) of the Δ(δλλ)(δλλ) conformer of **[Bi(macropa)]$^{+}$** from geometry optimizations (0 imaginary frequencies). .......... 131

**Table S42**: Cartesian coordinates (Å) of the Δ(λδλ)(λδλ) conformer of **[Bi(macropa)]$^{+}$** from geometry optimizations (0 imaginary frequencies). .......... 133

**Table S43**: Cartesian coordinates (Å) of the Δ(λλδ)(λλδ) conformer of **[Bi(macropa)]$^{+}$** from geometry optimizations (0 imaginary frequencies). .......... 135

**Table S44**: Cartesian coordinates (Å) of the Δ(δδλ)(δδλ) conformer of **[Bi(macropa)]$^{+}$** from geometry optimizations (0 imaginary frequencies). .......... 138

**Table S45**: Cartesian coordinates (Å) of the Δ(λδδ)(λδδ) conformer of **[Bi(macropa)]$^{+}$** from geometry optimizations (0 imaginary frequencies). .......... 140

**Table S46**: Cartesian coordinates (Å) of the Δ(δλδ)(δλδ) conformer of **[Bi(macropa)]$^{+}$** from geometry optimizations (0 imaginary frequencies). .......... 142

**Table S47**: Cartesian coordinates (Å) of the Δ(λλλ)(λλλ) conformer of **[Bi(macropapam)]$^{2+}$**from geometry optimizations (0 imaginary frequencies). .......... 144

**Table S48**: Cartesian coordinates (Å) of the Δ(δδδ)(δδδ) conformer of **[Bi(macropapam)]$^{2+}$** from geometry optimizations (0 imaginary frequencies). .......... 146

**Table S49**: Cartesian coordinates (Å) of the Δ(δλλ)(δλλ) conformer of **[Bi(macropapam)]$^{2+}$** from geometry optimizations (0 imaginary frequencies). .......... 148

**Table S50**: Cartesian coordinates (Å) of the Δ(λδλ)(λδλ) conformer of **[Bi(macropapam)]$^{2+}$** from geometry optimizations (0 imaginary frequencies). .......... 150

**Table S51**: Cartesian coordinates (Å) of the Δ(λλδ)(λλδ) conformer of **[Bi(macropapam)]$^{2+}$** from geometry optimizations (0 imaginary frequencies). .......... 152

**Table S52**: Cartesian coordinates (Å) of the Δ(δδλ)(δδλ) conformer of **[Bi(macropapam)]$^{2+}$** from geometry optimizations (0 imaginary frequencies). .......... 154

**Table S53**: Cartesian coordinates (Å) of the Δ(λδδ)(λδδ) conformer of **[Bi(macropapam)]$^{2+}$** from geometry optimizations (0 imaginary frequencies). .......... 156

**Table S54**: Cartesian coordinates (Å) of the Δ(δλδ)(δλδ) conformer of **[Bi(macropapam)]$^{2+}$** from geometry optimizations (0 imaginary frequencies). .......... 158

**Table S55**: Cartesian coordinates obtained for the transition state of the **[Bi(macropa)]$^{+}$** Δ(λδλ)(λδλ) ⇆ Δ(λδλ)(λδδ) equilibrum from geometry optimizations (1 imaginary frequency) .......... 160

**Table S56**: Cartesian coordinates (Å) of the Δ(λδλ)(λδδ) conformer of **[Bi(macropa)]$^{+}$** from geometry optimizations (0 imaginary frequencies). .......... 162

**Table S57**: Cartesian coordinates obtained for the transition state of the **[Bi(macropa)]**$^+$ Δ(λδλ)(λδδ) ⇆ Δ(δδλ)(λδδ) equilibrum from geometry optimizations (1 imaginary frequency). ....................................164

**Table S58**: Cartesian coordinates (Å) of the Δ(δδλ)(λδδ) conformer of **[Bi(macropa)]**$^+$ from geometry optimizations (0 imaginary frequencies). ....................................................................................................166

**Table S59**: Cartesian coordinates obtained for the transition state of the **[Bi(macropa)]**$^+$ Δ(δδλ)(λδδ)⇆ Δ(δλλ)(λδδ) equilibrum from geometry optimizations (1 imaginary frequency). ....................................168

**Table S60**: Cartesian coordinates (Å) of the Δ(δλλ)(λδδ) conformer of **[Bi(macropa)]**$^+$ from geometry optimizations (0 imaginary frequencies). This conformation of the complex corresponds to the previously published crystal structure. ...............................................................................................................170

**Table S61**: Cartesian coordinates obtained for the transition state of the **[Bi(macropa)]**$^+$ Δ(δδλ)(λδδ)⇆ Δ(δδδ)(λδδ) equilibrum from geometry optimizations (1 imaginary frequency). ....................................172

**Table S62**: Cartesian coordinates (Å) of the Δ(δδδ)(λδδ) conformer of **[Bi(macropa)]**$^+$ from geometry optimizations (0 imaginary frequencies). ....................................................................................................174

**Table S63**: Cartesian coordinates obtained for the transition state of the **[Bi(macropa)]**$^+$ Δ(δδδ)(λδδ)⇆ Δ(δδδ)(δδδ) equilibrum from geometry optimizations (1 imaginary frequency).....................................176

**Table S64**: Cartesian coordinates obtained for the transition state of the **[Bi(macropa)]**$^+$ Δ(δδλ)(λδδ)⇆ Δ(δδλ)(δδδ) equilibrum from geometry optimizations (1 imaginary frequency). ....................................178

**Table S65**: Cartesian coordinates (Å) of the Δ(δδλ)(δδδ) conformer of **[Bi(macropa)]**$^+$ from geometry optimizations (0 imaginary frequencies). ....................................................................................................180

**Table S66**: Cartesian coordinates obtained for the transition state of the **[Bi(macropa)]**$^+$ Δ(δδλ)(δδδ) ⇆ Δ(δδδ)(δδδ) equilibrum from geometry optimizations (1 imaginary frequency).....................................182

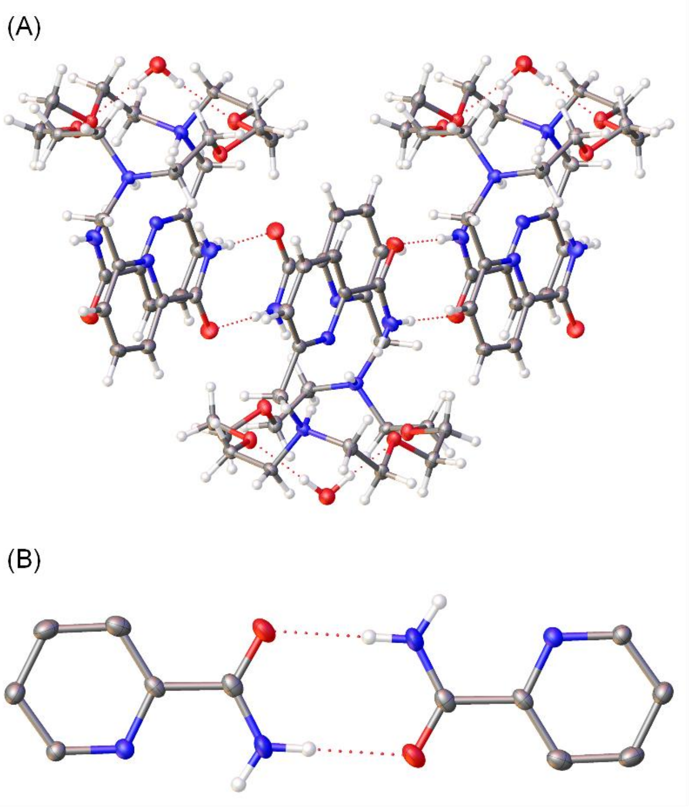


**Figure S1**: (A) Three $[H_2macropam]^+$ units connected through hydrogen bonding in an $R_2^2(8)$ motif, dotted lines represent hydrogen bonding. (B) Close-up of the $R_2^2(8)$ motif. The ORTEP plot is at 50%.

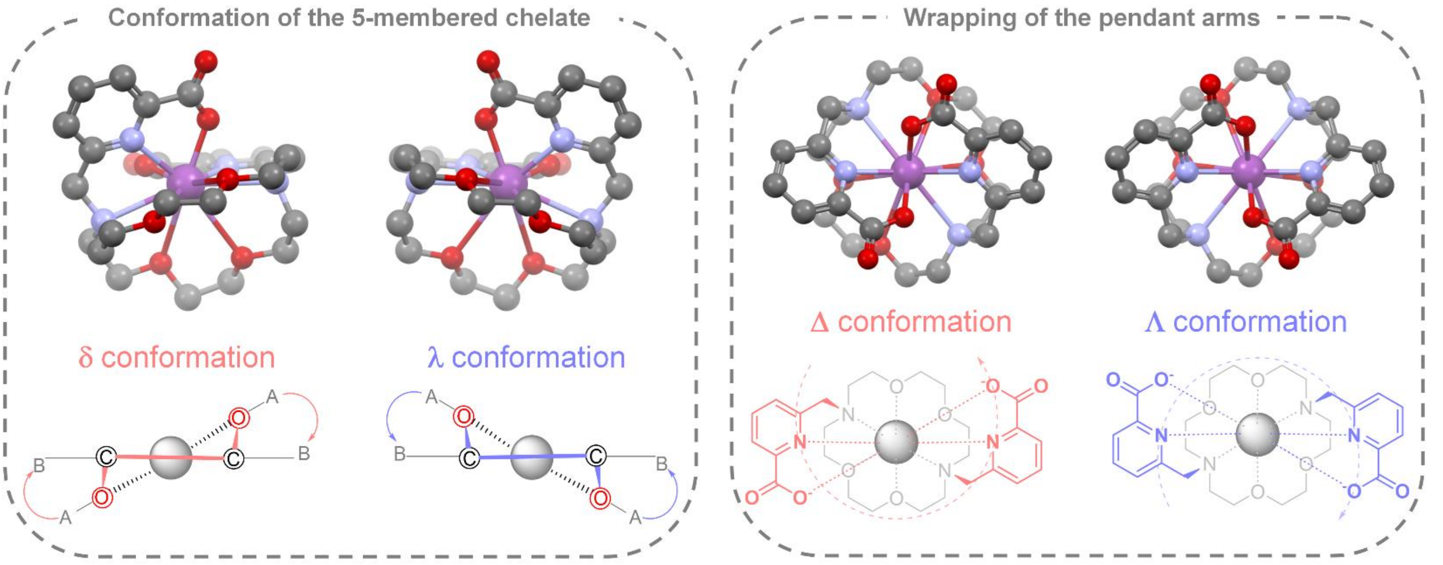


**Figure S2**: Sources of helicity in complexes of macropa-based systems where the picolinate arms adopt a *syn* conformation represented through DFT optimized structures and ChemDraw Schemes.

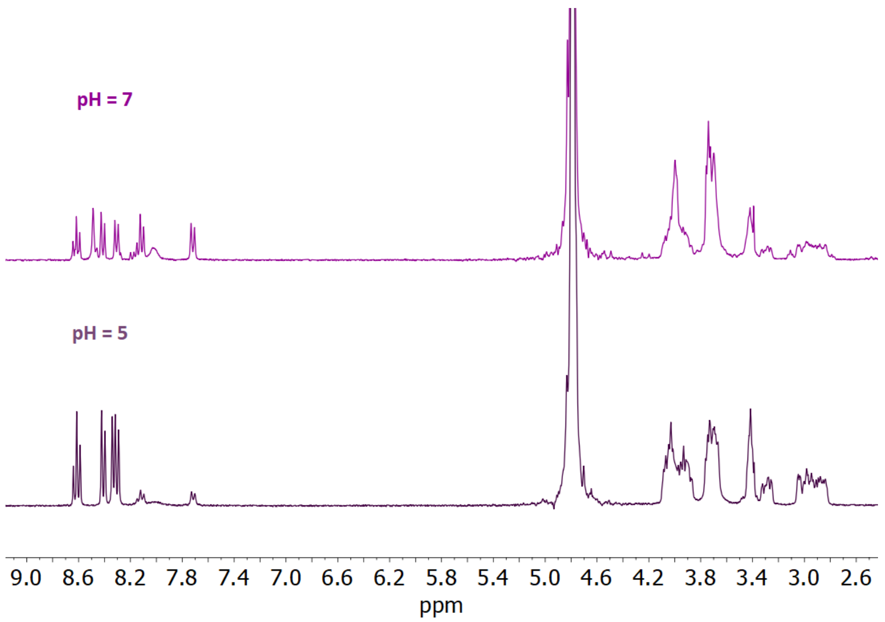


**Figure S3**: $^1$H-NMR (300 MHz, 298 K) of the attempt to prepare $[Bi(macropam)]^{3+}$ at pH 5 (bottom) and pH 7 (top). pH measured with pH paper. The proportion of the signals corresponding to the free ligand (aromatic signals between 7.6 and 8.2 ppm) becomes larger upon increasing the pH.

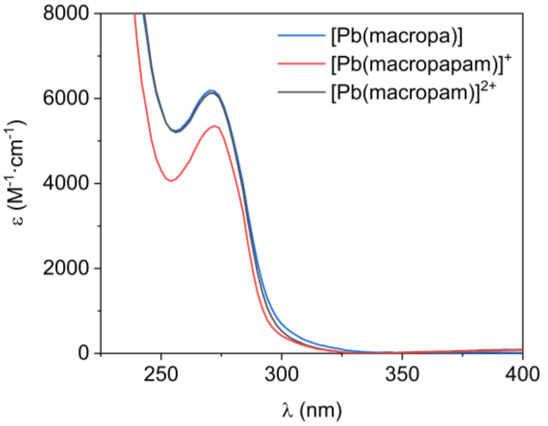


**Figure S4**: UV-Vis spectra of the Pb(II) complexes ($5 \times 10^{-5}$ M), showing a maximum at 272 nm corresponding to the picolinate groups**.**

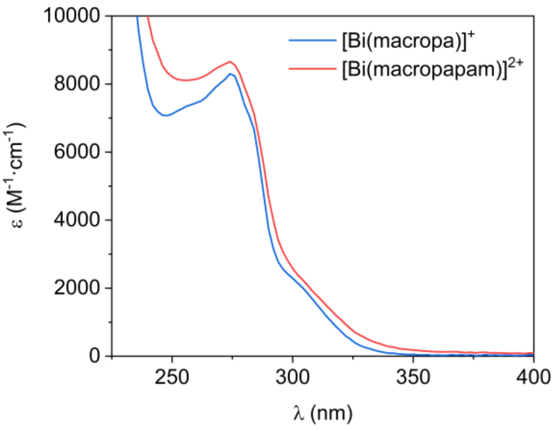


**Figure S5**: UV-Vis spectra of the Bi(III) complexes ($5 \times 10^{-5}$ M), showing a maximum at 274 nm corresponding to the picolinate groups and a shoulder around 300 nm corresponding to the 6sp ← 6s transition of Bi(III).

**Table S1**: $^{1}$H-NMR chemical shifts (ppm) and assignment for the Pb(II) and Bi(III) complexes discussed in the manuscript (500 MHz, 298 K, $D_2O$).

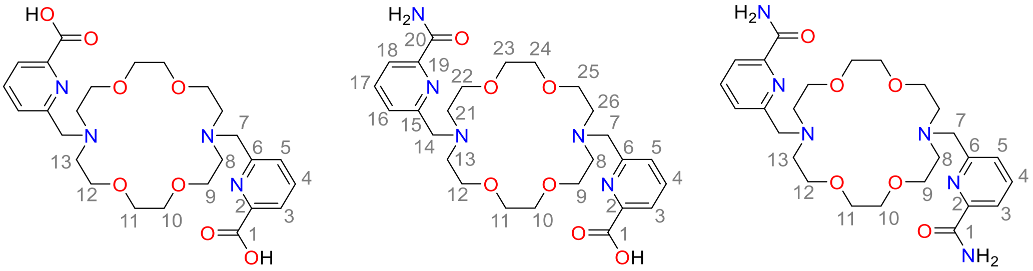

| | [Pb(macropa)] | [Pb(macropapam)]$^{+}$ | [Pb(macropam)]$^{2+}$ | [Bi(macropa)]$^{+}$ | [Bi(macropapam)]$^{2+}$ |
|---|---|---|---|---|---|
| **H3** | 7.50 | 7.51 | 7.62 | 8.21 | 8.18 |
| **H4** | 7.93 | 7.98 | 8.05 | 8.47 | 8.48 |
| **H5** | 7.64 | 7.70 | 7.78 | 8.16 | 8.18 |
| **H7a** | 5.13 | 5.16 | 5.00 | 4.64 | 4.66 |
| **H7b** | 3.87 – 3.76 | 4.01 – 3.44 | 4.07 – 3.84 | 4.51 | 4.56 |
| **H8ax** | 2.59 | | 2.61 | 3.17 | |
| **H8eq** | 2.45 | | 2.51 | 2.89 | |
| **H9ax** | 3.93 | | 4.07 – 3.84 | 3.53 | |
| **H9eq** | 3.45 | | 3.51 | 3.89 | |
| **H10ax** | 3.87 – 3.76 | | 4.07 – 3.84 or 3.84 – 3.70 | 3.70 or 2.54 | |
| **H10eq** | 3.75 – 3.64 or 3.57 | | 3.62, 4.07 – 3.84, or 3.84 – 3.70 | 3.70 or 2.54 | |
| **H11ax** | 3.87 – 3.76 | | 4.07 – 3.84 or 3.84 – 3.70 | 3.53 or 2.77 | |
| **H11eq** | 3.75 – 3.64 or 3.57 | | 3.62, 4.07 – 3.84, or 3.84 – 3.70 | 3.53 or 2.77 | |
| **H12ax** | 4.43 | | 4.39 | 3.77 | |
| **H12eq** | 3.75 – 3.64 | | 3.84 – 3.70 | 3.96 | |
| **H13ax** | 3.29 | | 3.38 | 3.41 | |
| **H13eq** | 2.59 | | 2.61 | 3.17 | |
| **H14a** | | 4.98 | | | 4.71 |
| **H14b** | | 4.01 – 3.44 | | | 4.71 |
| **H16** | | 7.70 | | | 8.26 |
| **H17** | | 7.98 | | | 8.57 |
| **H18** | | 7.57 | | | 8.39 |
| **H21ax** | | | | | |
| **H21eq** | | | | | |
| **H22ax** | | | | | |
| **H22eq** | | | | | |
| **H23ax** | | | | | |
| **H23eq** | | | | | |
| **H24ax** | | | | | |
| **H24eq** | | | | | |
| **H25ax** | | | | | |
| **H25eq** | | | | | |
| **H26ax** | | | | | |
| **H26eq** | | | | | |

**Table S2**: $^{13}$C-NMR chemical shifts (ppm) and assignment for the Pb(II) and Bi(III) complexes discussed in the manuscript (126 MHz, 298 K, $D_2O$).

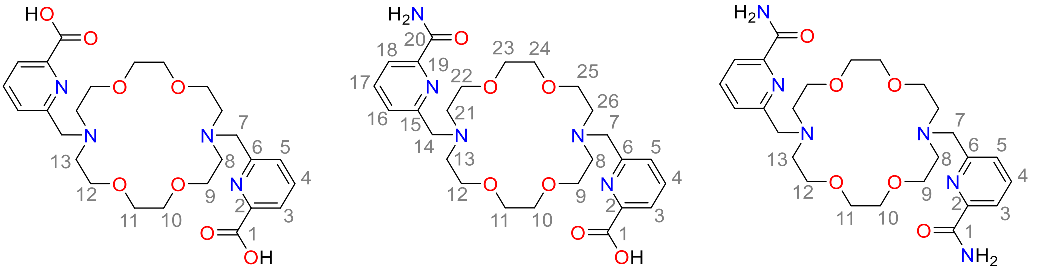


| | [Pb(macropa)] | $[Pb(macropapam)]^+$ | $[Pb(macropam)]^{2+}$ | $[Bi(macropa)]^+$ | $[Bi(macropapam)]^{2+}$ |
|---|---|---|---|---|---|
| **C1** | 171.96 | 172.33 | 169.18 | 170.26 | 170.36 |
| **C2** | 149.64 | 149.68 | 146.49 | 147.96 | 147.84 |
| **C3** | 123.18 | 123.44 | 122.06 | 125.86 | 126.11 |
| **C4** | 139.90 | 140.07 or 139.99 | 140.18 | 143.10 | 143.16 |
| **C5** | 127.01 | 127.33 | 128.45 | 127.82 | 128.23 |
| **C6** | 159.05 | 158.82 | 160.53 | 158.35 | 158.50 |
| **C7** | 59.33 | 59.27 | 58.87 | 61.26 | 61.01 |
| **C8** | 54.65 | | 54.90 | 54.94 | |
| **C9** | 68.37 | | 68.40 | 68.66 | |
| **C10** | 70.04 or 69.44 | | 69.38 or 70.09 | 67.40 | |
| **C11** | 70.04 or 69.44 | | 69.38 or 70.09 | 68.21 | |
| **C12** | 67.14 | | 67.14 | 64.70 | |
| **C13** | 53.51 | | 53.31 | 55.64 | |
| **C14** | | 58.93 | | | 61.25 |
| **C15** | | 160.59 | | | 160.82 |
| **C16** | | 128.07 | | | 128.91 |
| **C17** | | 140.07 or 139.99 | | | 143.21 |
| **C18** | | 121.74 | | | 124.69 |
| **C19** | | 146.82 | | | 145.72 |
| **C20** | | 168.94 | | | 169.23 |
| **C21** | | | | | |
| **C22** | | | | | |
| **C23** | | | | | |
| **C24** | | | | | |
| **C25** | | | | | |
| **C26** | | | | | |

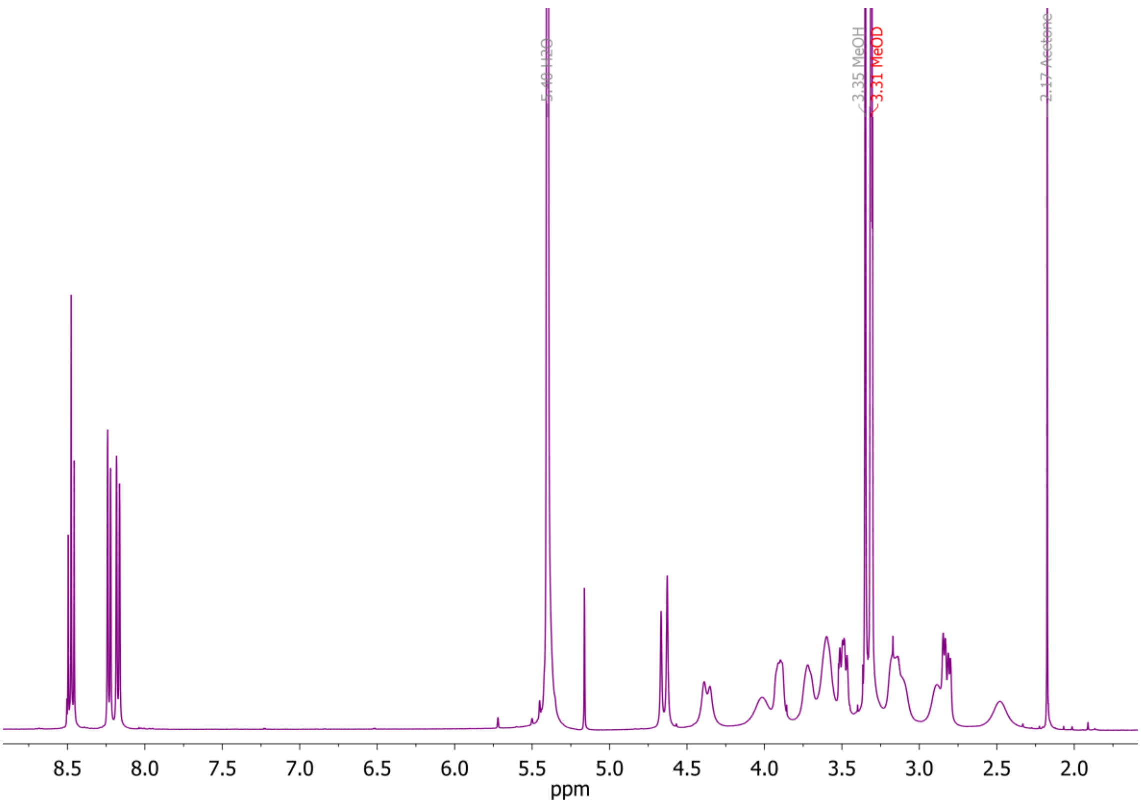


**Figure S6**: $^1$H-NMR (400 MHz, 203.15 K, MeOD) of $[Bi(macropa)]^+$ showing broadening of the signals of the aliphatic region.

**Table S3**: Relative Free Energies (kJ/mol) in Aqueous Solution (PCM) of the Conformations of the Bimacropa Complex on the Pathways Between the Most Stable Conformation Δ(λδλ)(λδλ) and the Conformations of the two Crystal Structures, along with the Transition States (TS).

| | |
|---|---|
| **Δ(λδλ)(λδλ)** | 0 |
| **Δ(λδλ)(λδλ) to Δ(λδλ)(λδδ) TS** | 37.24268 |
| **Δ(λδλ)(λδδ)** | 7.80035 |
| **Δ(λδλ)(λδδ) to Δ(δδλ)(λδδ) TS** | 44.88288 |
| **Δ(δδλ)(λδδ)** | 4.46335 |
| **Δ(δδλ)(λδδ) to Δ(δλλ)(λδδ)TS** | 27.678 |
| **Δ(δλλ)(λδδ)** | 5.84173 |
| **Δ(δδλ)(λδδ) to Δ(δδδ)(λδδ)TS** | 36.06646 |
| **Δ(δδδ)(λδδ)** | 9.9165 |
| **Δ(δδδ)(λδδ) to Δ(δδδ)(δδδ)TS** | 49.67179 |
| **Δ(δδλ)(λδδ) to Δ(δδλ)(δδδ)TS** | 36.41565 |
| **Δ(δδλ)(δδδ)** | 31.11215 |
| **Δ(δδλ)(δδδ) to Δ(δδδ)(δδδ)TS** | 36.0192 |
| **Δ(δδδ)(δδδ)** | 5.74196 |

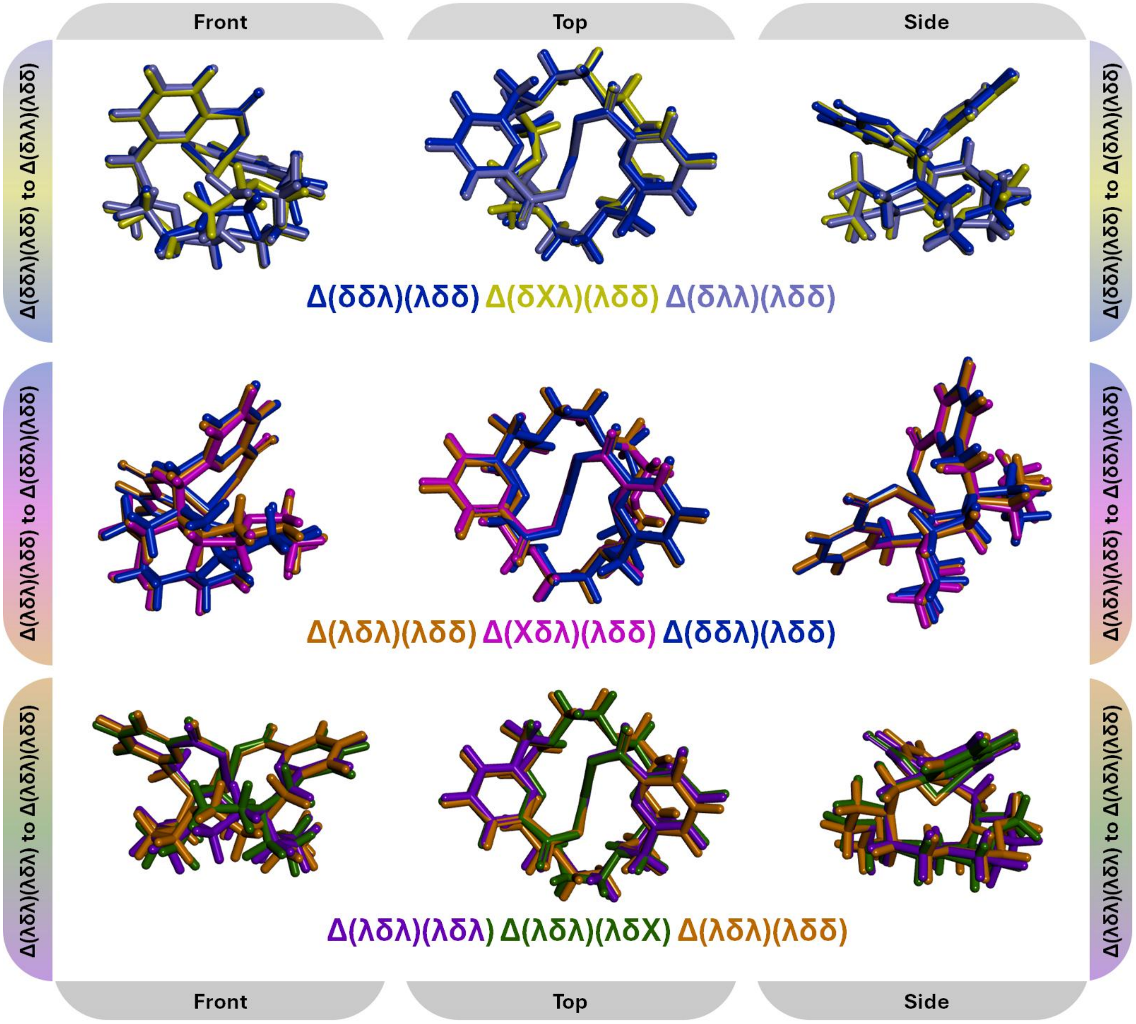


**Figure S7**: Superimposed structures of different conformations of $[Bi(macropa)]^+$ illustrating that the most important differences between conformations and transition states involve the chelate ring being inverted. X denotes an eclipsed conformation of the 5-membered chelate ring in the transition states. Three different views are included for each set of three structures: a front view of the 5 membered chelate ring, a top view of the complex and a side view of the 5-membered chelate ring.

**Estimation of the rate constant for the λ/δ inversion.**

The Eyring equation (1) can be used to determine the rate of a process ($k$) at a given temperature knowing the Gibbs free energy of activation ($\Delta G^{\ddagger}$).[1] In our case, the Gibbs free energy of activation is the difference between the energy of our minima and the energy of our transition states as determined through DFT calculations.

$$k = \frac{\kappa k_B T}{h} e^{-\frac{\Delta G^{\ddagger}}{RT}} \qquad (1)$$

All the processes studied have Gibbs free energies of activation under 50 kJ·mol$^{-1}$, the highest being that between Δ(δδδ)(λδδ) and Δ(δδδ)(δδδ), with a Gibbs free energy of activation of around 40 kJ·mol$^{-1}$ . Therefore, calculated the rate of a process with an activation of 50 kJ·mol$^{-1}$, giving 10681.70 Hz. From this value we can calculate the lifetime ($\tau = \frac{1}{k}$) of the process: $\tau = 93\ \mu s$.

**Table S4**: Radiochemical yields (RCYs, %) for the [$^{203}$Pb]Pb(II) radiolabeling reactions after 60 minutes (n=3), room temperature .

| Concentration (M) | **H2macropa** | **Hmacropapam** | **macropam** | **DOTAM** |
|---|---|---|---|---|
| **10$^{-4}$** | 100 ± 0 | 100 ± 0 | 65 ± 12 | 100 ± 0 |
| **10$^{-5}$** | 100 ± 0 | 96 ± 3 | – | 100 ± 0 |
| **10$^{-6}$** | 100 ± 0 | 90 ± 2 | – | 100 ± 0 |
| **10$^{-7}$** | 84 ± 4 | 11 ± 1 | – | 37 ± 7 |
| **10$^{-8}$** | 9 ± 5 | 2 ± 0 | – | 5 ± 0 |
| **10$^{-9}$** | 3 ± 4 | – | – | – |

**Table S5**: Radiochemical yields (RCYs, %) for the [$^{213}$Bi]Bi(III) radiolabeling reactions after 5 minutes (n=2), room temperature.

| Concentration (M) | **H2macropa** | **Hmacropapam** | **macropam** | **DOTAM** | **DOTA** |
|---|---|---|---|---|---|
| **10$^{-4}$** | 98 ± 3 | 15 ± 6 | 4 ± 1 | 13 ± 3 | 2 ± 3 |
| **10$^{-5}$** | 87 ± 5 | 8 ± 1 | 1 ± 2 | – | – |
| **10$^{-6}$** | 50 ± 9 | 7 ± 2 | – | – | – |
| **10$^{-7}$** | 25 ± 1 | 7 ± 2 | – | – | – |
| **10$^{-8}$** | 0 ± 0 | 1 ± 2 | – | – | – |

**Table S6**: Stability of the $^{203}$Pb-labeled chelators (intact complex, %) in a 20-fold excess EDTA (n=3).

| Time (h) | **H2macropa** | **Hmacropapam** | **DOTAM** |
|---|---|---|---|
| **0** | 100 ± 0 | 100 ± 0 | 100 ± 0 |
| **0.0833** | – | 100 ± 0 | 100 ± 0 |
| **0.1667** | 100 ± 0 | – | – |
| **0.5** | 100 ± 0 | 78 ± 3 | 100 ± 0 |
| **1** | 100 ± 0 | 66 ± 3 | 100 ± 0 |
| **2** | 99 ± 2 | 53 ± 10 | 100 ± 0 |
| **24** | – | 9 ± 1 | 100 ± 0 |
| **31** | 84 ± 1 | – | – |
| **48** | – | – | 100 ± 0 |
| **72** | – | – | 100 ± 0 |
| **144** | 12 ± 5 | – | 100 ± 0 |

[1] Laidler, K. J.; King, M. C. Development of Transition-State Theory. *J. Phys. Chem.* **1983**, *87* (15), 2657–2664. https://doi.org/10.1021/j100238a002.

**Table S7**: Stability of the $^{203}Pb$-labeled chelators (intact complex, %) in a 20-fold excess Pb(II)(n=3).

| Time (h) | **$H_2$macropa** | **Hmacropapam** | **DOTAM** |
|---|---|---|---|
| **0** | 100 ± 0 | 100 ± 0 | 100 ± 0 |
| **0.0833** | – | 7 ± 1 | 100 ± 0 |
| **0.1667** | 2 ± 1 | – | – |
| **0.5** | 9 ± 1 | 5 ± 0 | 100 ± 0 |
| **1** | 4 ± 1 | 5 ± 1 | 100 ± 1 |
| **2** | 2 ± 0 | – | 100 ± 0 |
| **24** | – | – | 100 ± 0 |
| **48** | – | – | 100 ± 0 |
| **72** | – | – | 100 ± 0 |

**Table S8**: Stability of the $^{203}Pb$-labeled chelators (intact complex, %) in a stable metal cocktail containing 10 equivalents each of $ZnCl_2$, $FeCl_3$, $CuCl_2$, $MgCl_2$, and $CoCl_2$ (n=3).

| Time (h) | **$H_2$macropa** | **Hmacropapam** | **DOTAM** |
|---|---|---|---|
| **0** | 100 ± 0 | 100 ± 0 | 100 ± 0 |
| **0.1667** | 11 ± 4 | 71 ± 3 | 100 ± 0 |
| **0.5** | 13 ± 4 | 65 ± 4 | 100 ± 0 |
| **1** | 15 ± 4 | 78 ± 5 | 100 ± 0 |
| **2** | – | 61 ± 4 | 100 ± 0 |
| **31** | – | 52 ± 7 | 100 ± 0 |
| **144** | – | 56 ± 11 | 100 ± 0 |

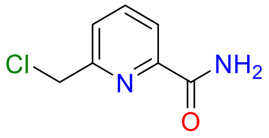


**Scheme S1**: Structure of **6-(chloromethyl)picolinamide**

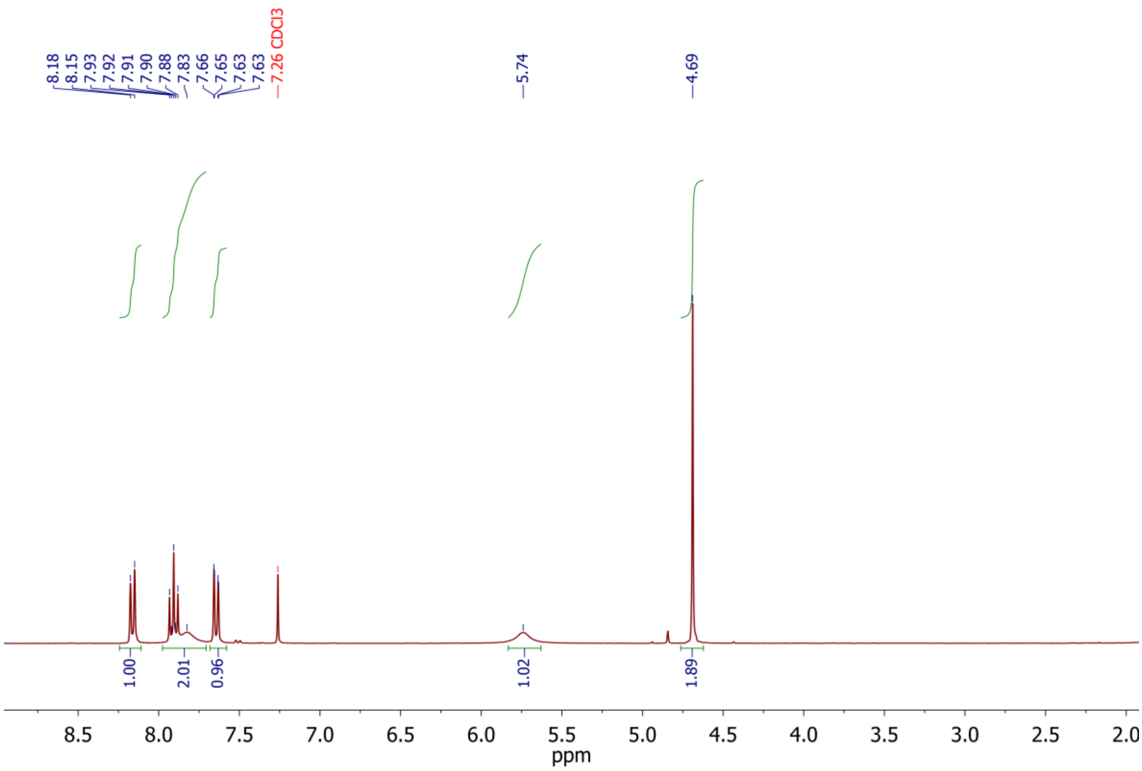


**Figure S8**: $^{1}$H NMR spectrum of 6-(chloromethyl)picolinamide (300 MHz, $CDCl_3$, 298 K).

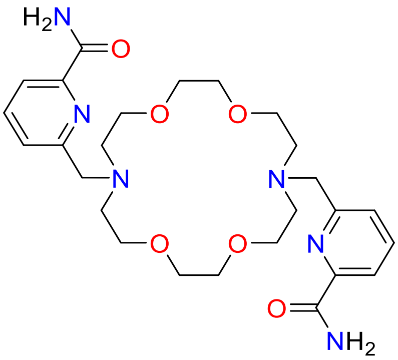


**Scheme S2**: Structure of **macropam**.

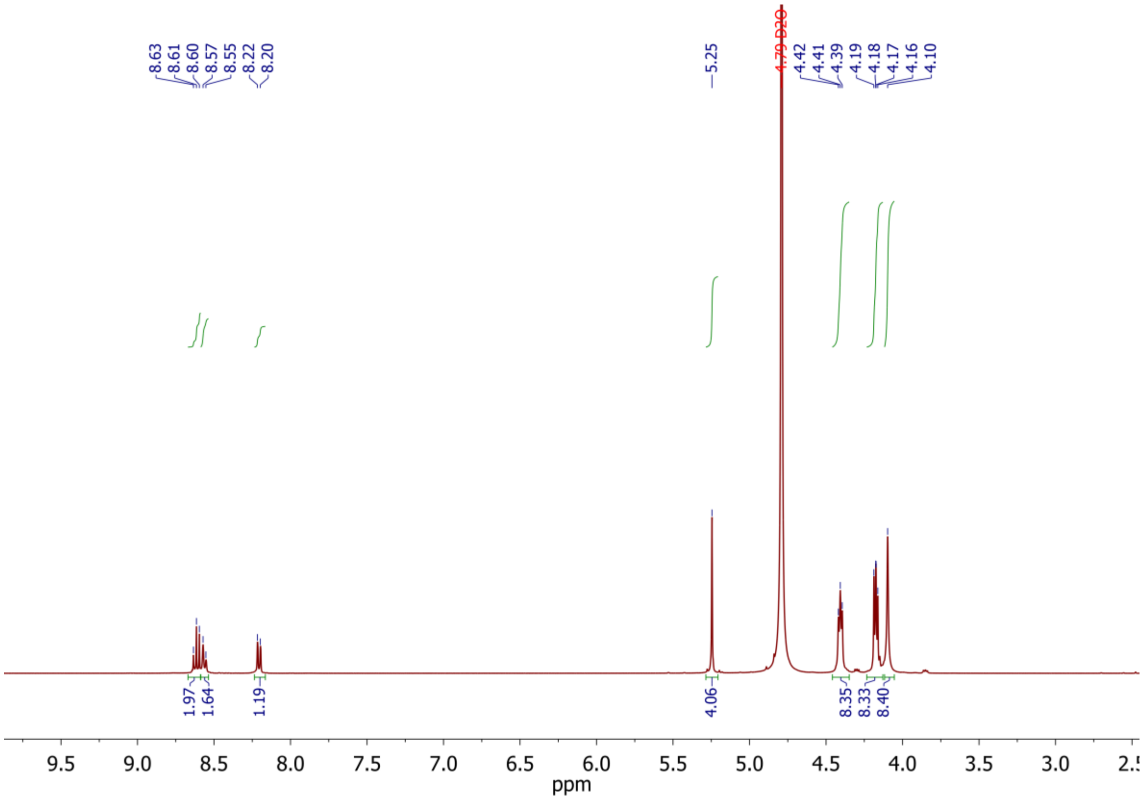


**Figure S9**: $^{1}$H NMR spectrum of **macropam** (400 MHz, $D_2O$, pD ~ 3, 342.8 K).

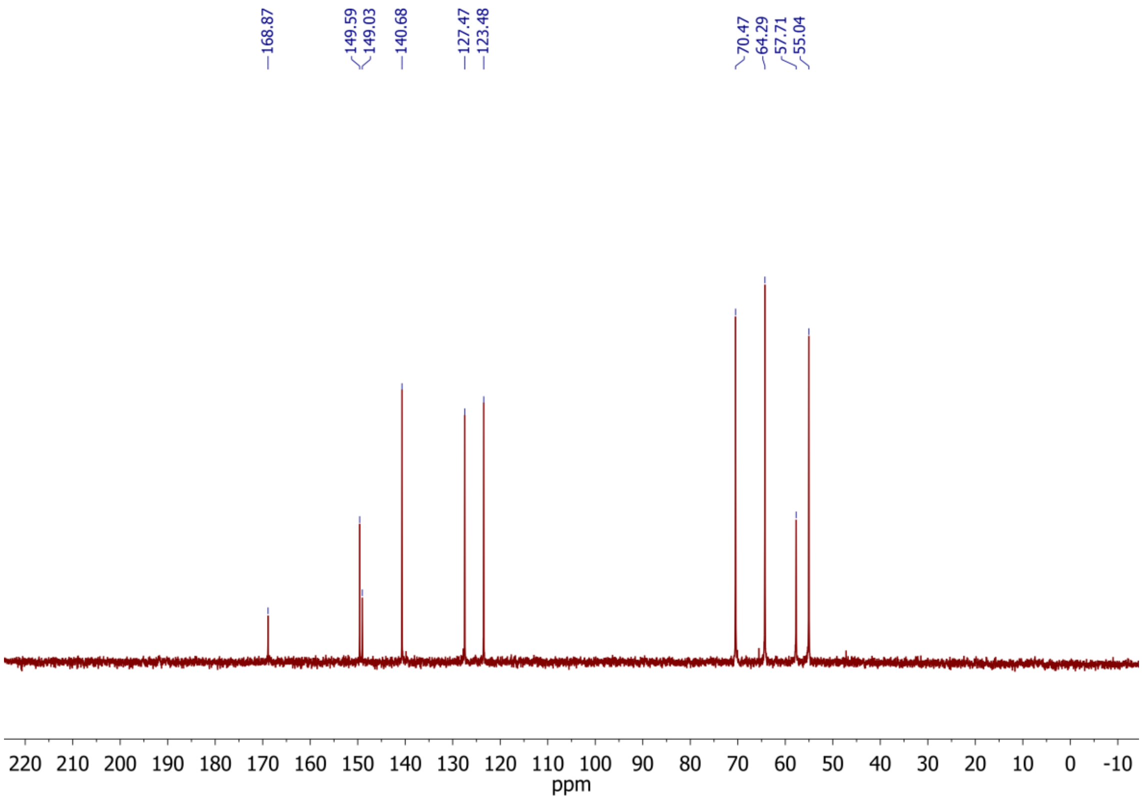


**Figure S10**: $^{13}$C NMR spectrum of **macropam** (126 MHz, $D_2O$, pD= 7, 298 K).

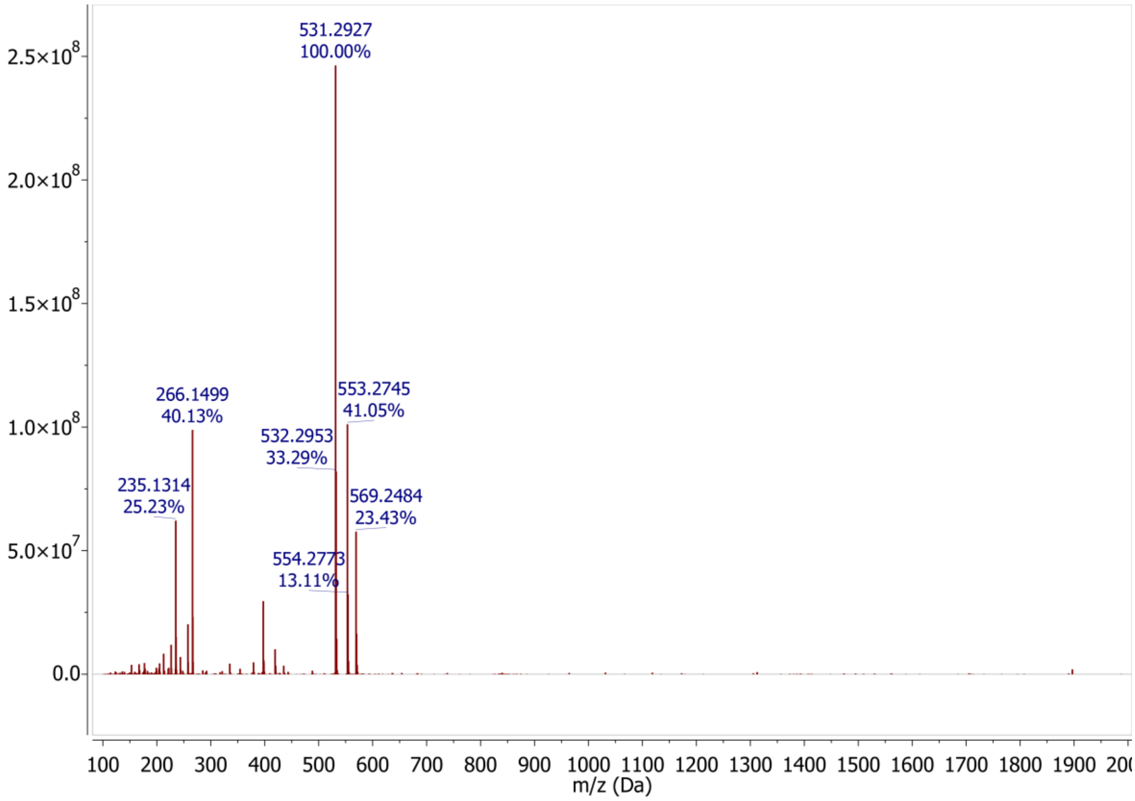


**Figure S11**: Experimental high resolution mass spectrum ($ESI^+$) of compound **macropam**.

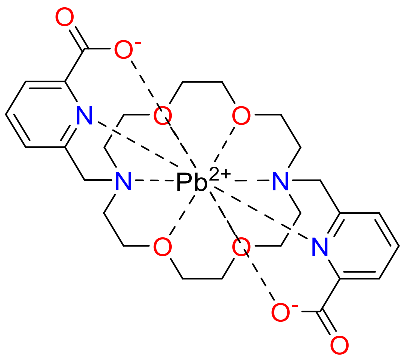

**Scheme S3**: Structure of **[Pb(macropa)]**

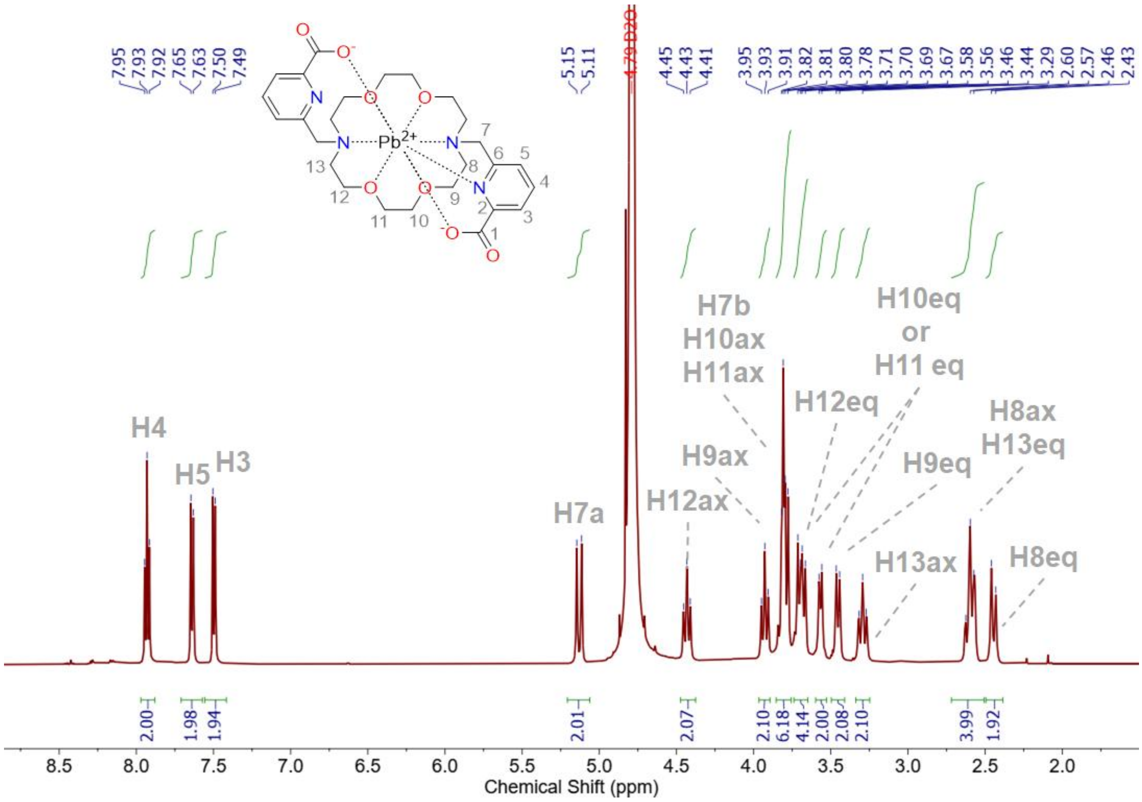


**Figure S12**: $^{1}H$ NMR spectrum of complex **[Pb(macropa)]** (500 MHz, $D_2O$, pD = 6, 298 K).

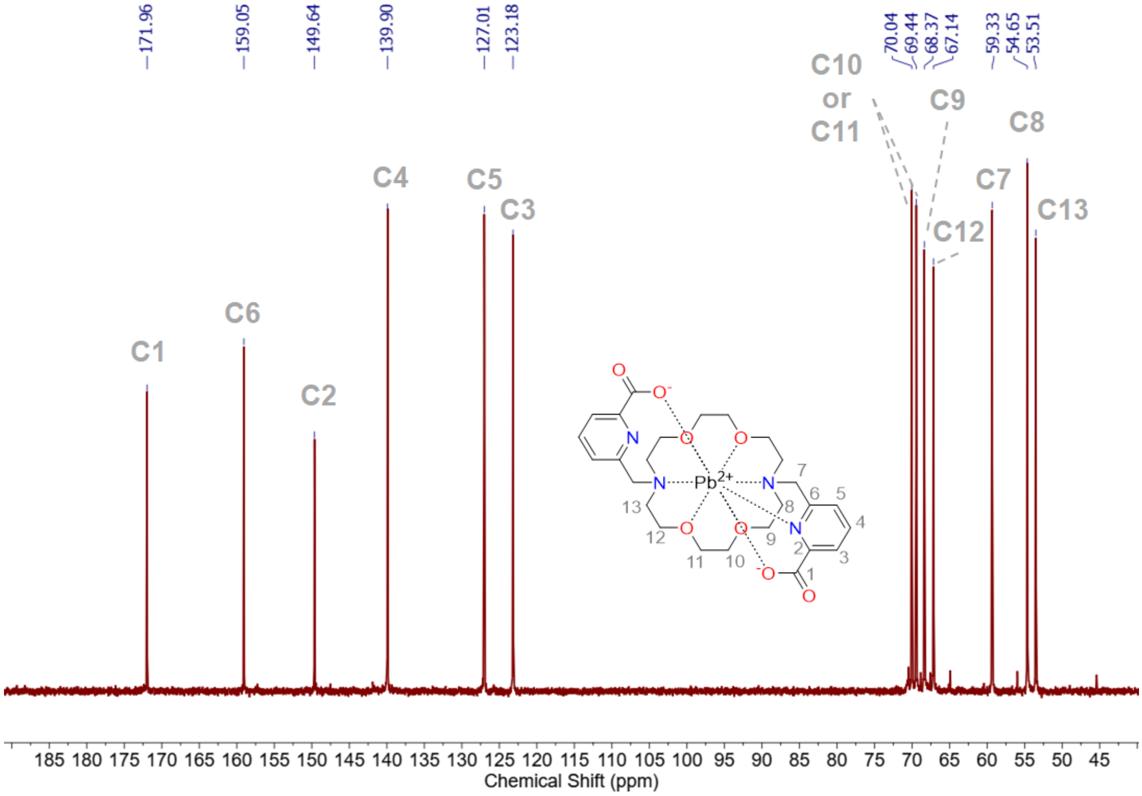


**Figure S13**: $^{13}C$ NMR spectrum of complex **[Pb(macropa)]** (126 MHz, $D_2O$, pD = 6, 298 K).

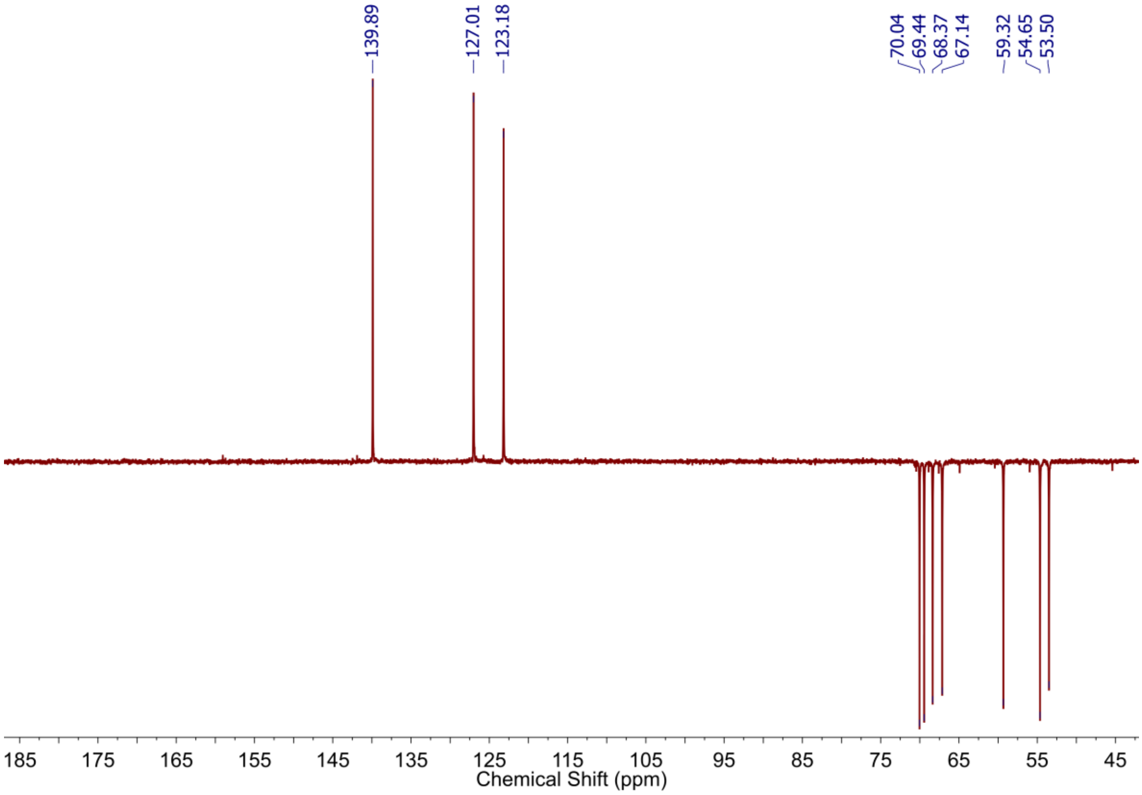


**Figure S14**: $^{13}C$ DEPT-135 NMR spectrum of complex **[Pb(macropa)]** (126 MHz, $D_2O$, pD = 6, 298 K).

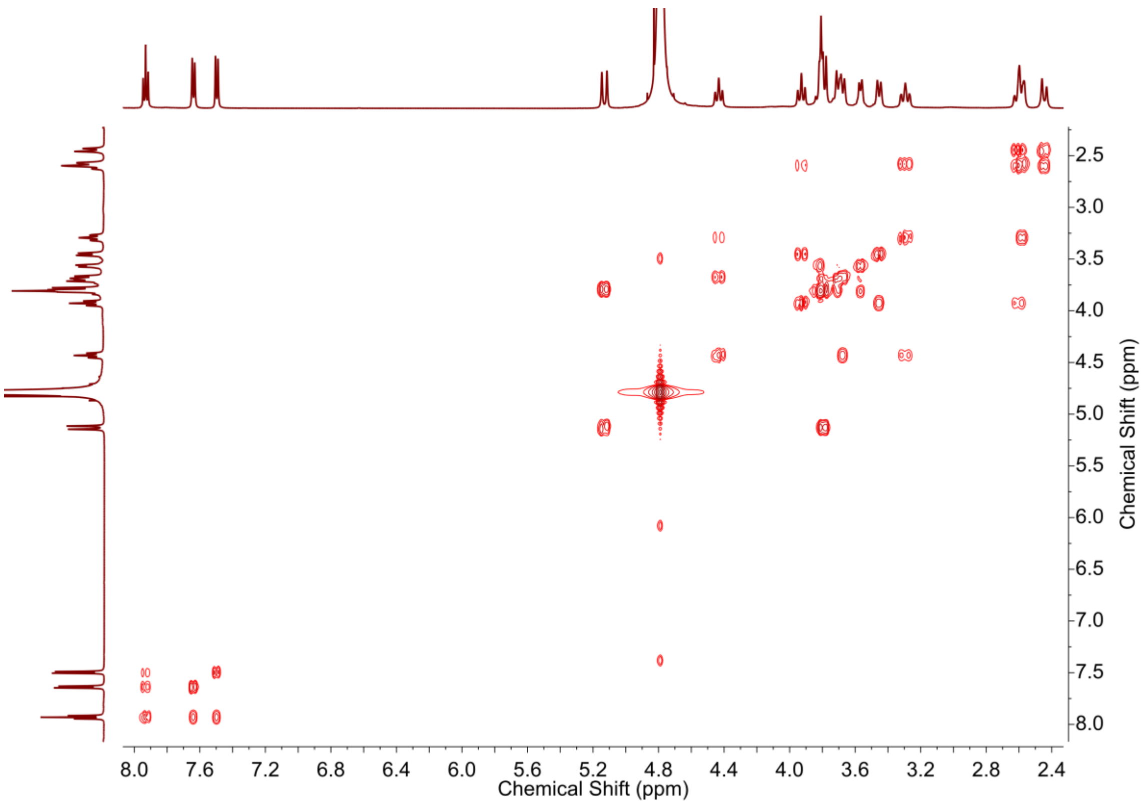


**Figure S15**: $^{1}H$-$^{1}H$ COSY NMR spectrum of complex **[Pb(macropa)]** (500 MHz, $D_2O$, pD = 6.5, 298 K).

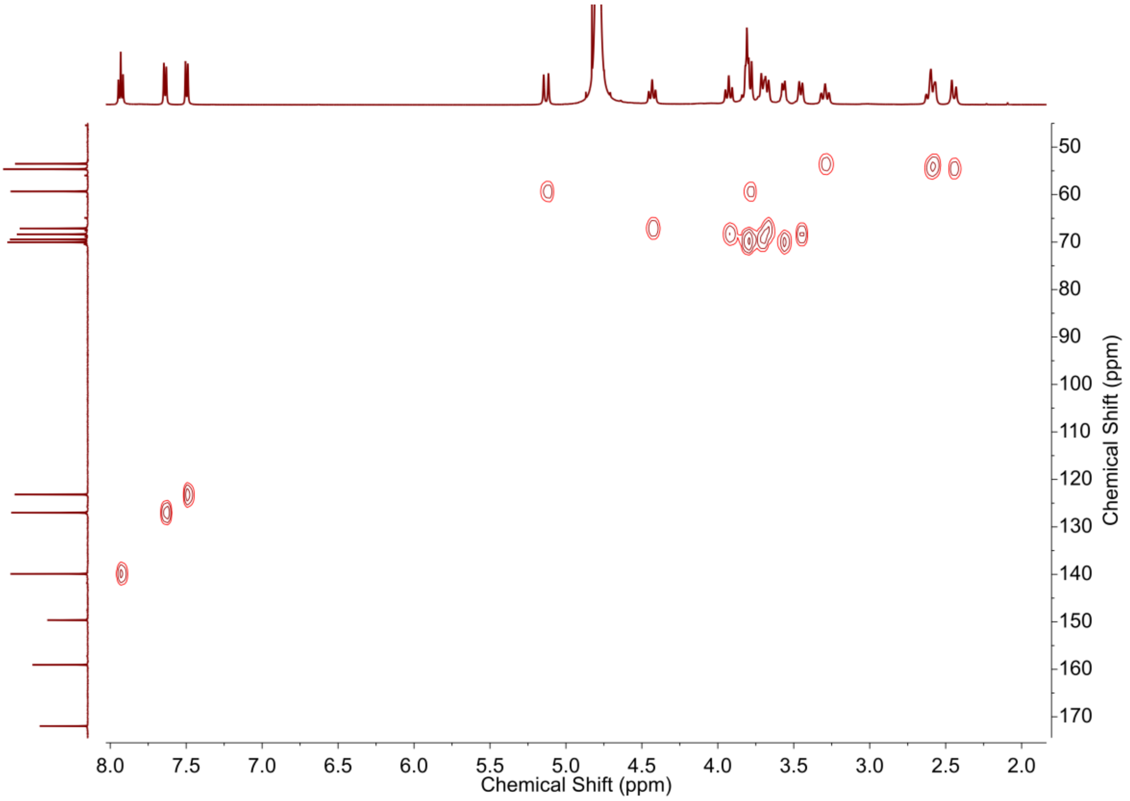


**Figure S16**: $^{1}H$-$^{13}C$ HSQC NMR spectrum of complex **[Pb(macropa)]** (500-127 MHz, $D_2O$, pD = 6, 298 K).

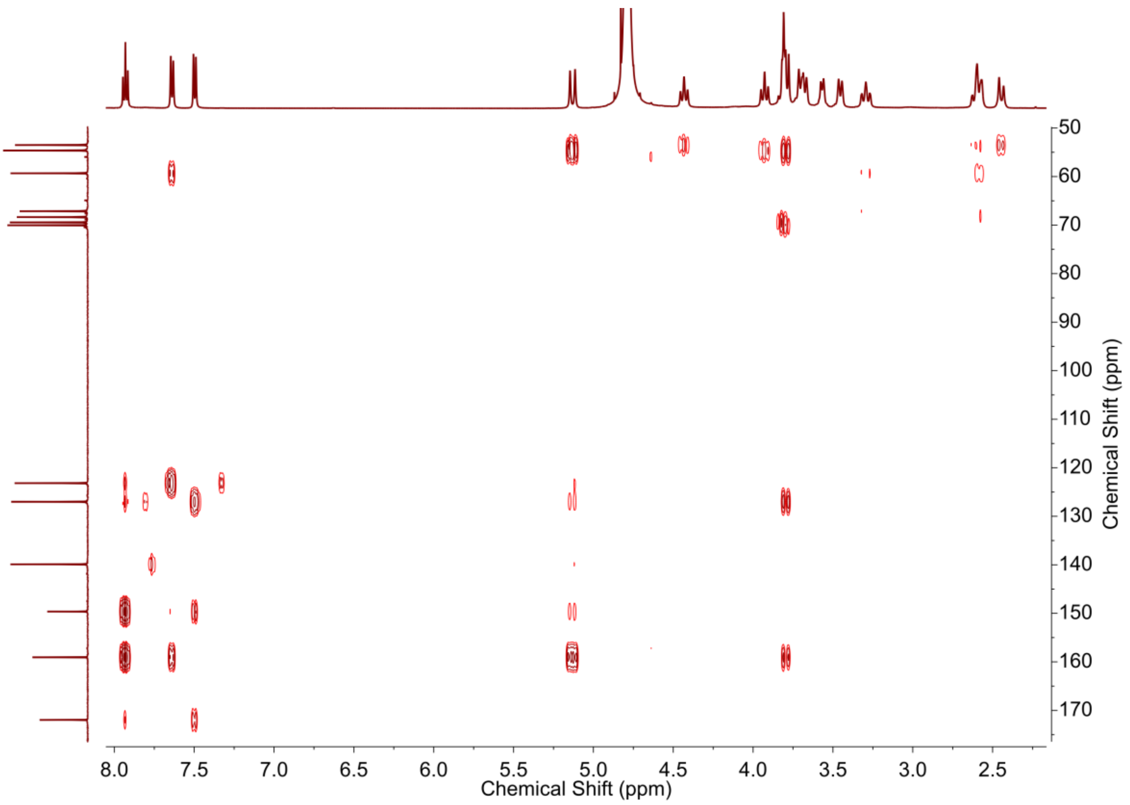


**Figure S17**: ¹H-¹³C HMBC NMR spectrum of complex **[Pb(macropa)]** (500-127 MHz, $D_2O$, pD = 6, 298 K).

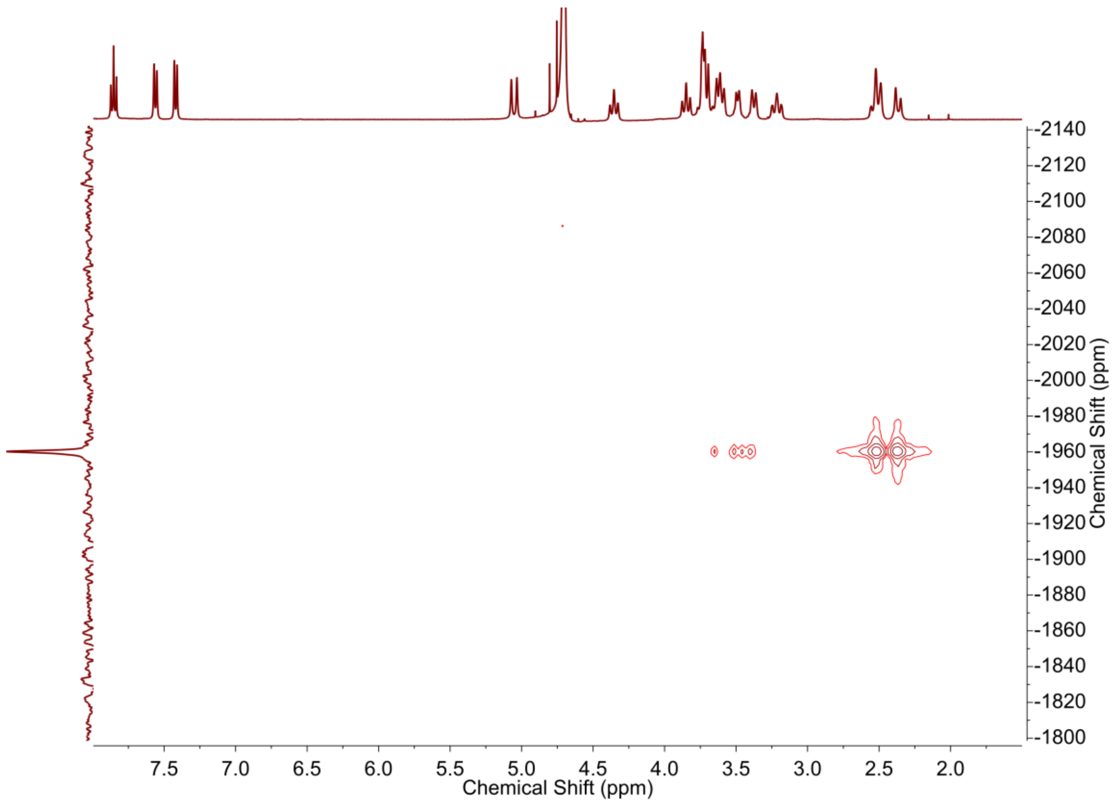


**Figure S18**: ¹H-²⁰⁷Pb HMQC NMR spectrum of complex **[Pb(macropa)]** (400-84 MHz, $D_2O$, pD = 6, 298 K).

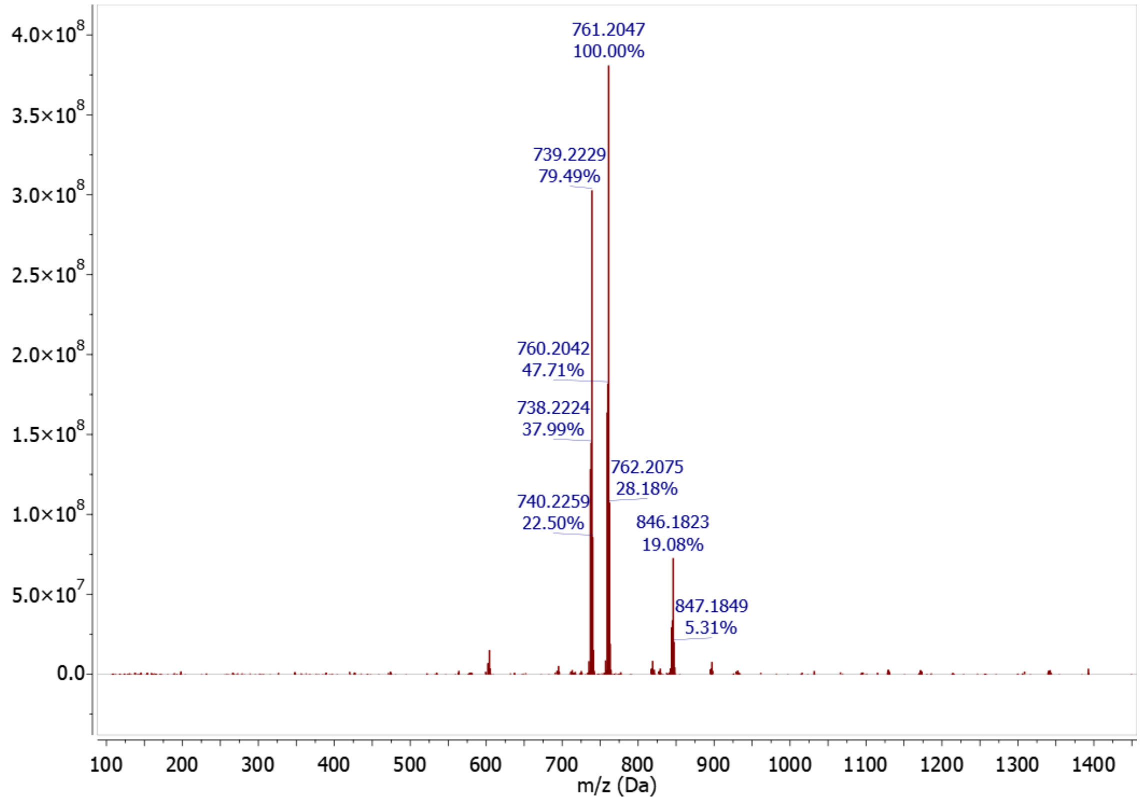


**Figure S19**: Experimental high resolution mass spectrum (ESI$^+$) of compound **[Pb(macropa)]**

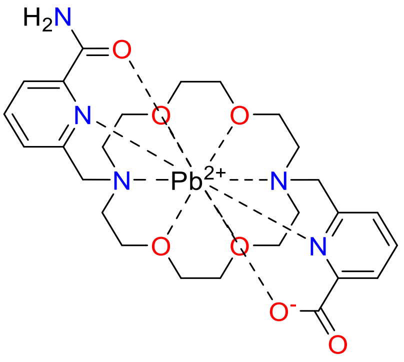


**Scheme S4**: Structure of **[Pb(macropapam)]**$^{+}$

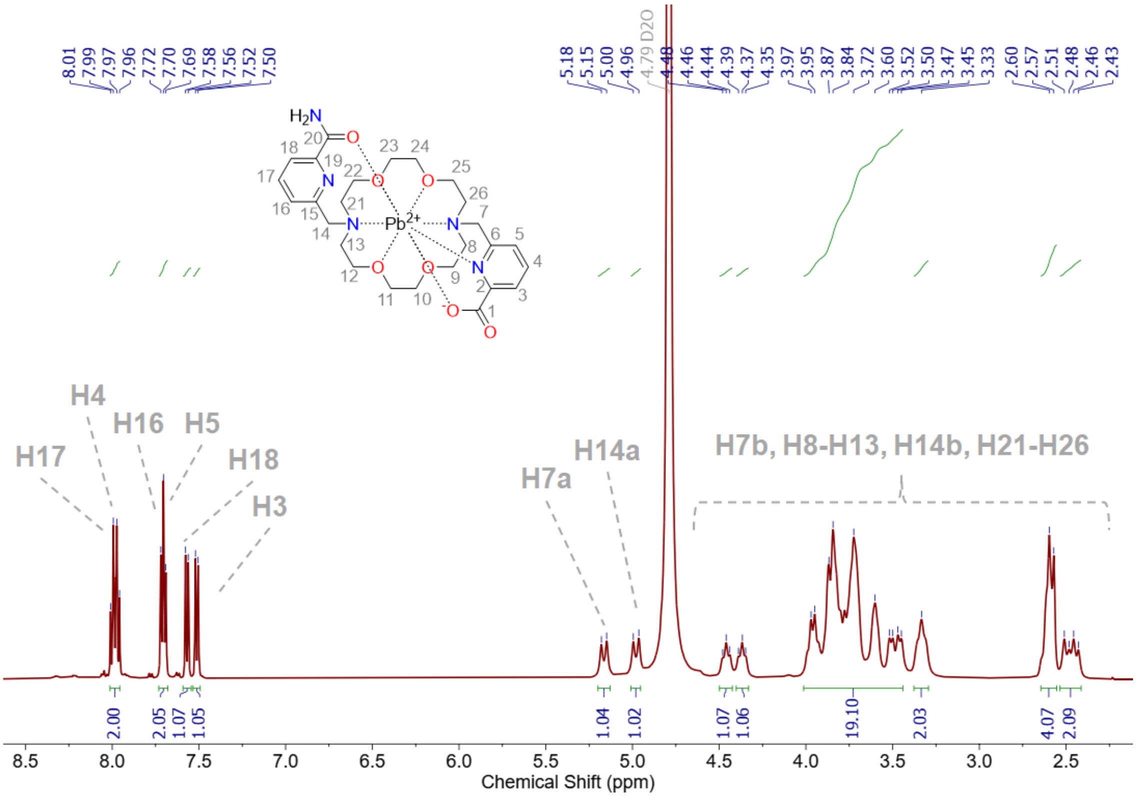


**Figure S20**: $^{1}$H NMR spectrum of complex **[Pb(macropapam)]**$^{+}$ (500 MHz, $D_2O$, pD = 6, 298 K).

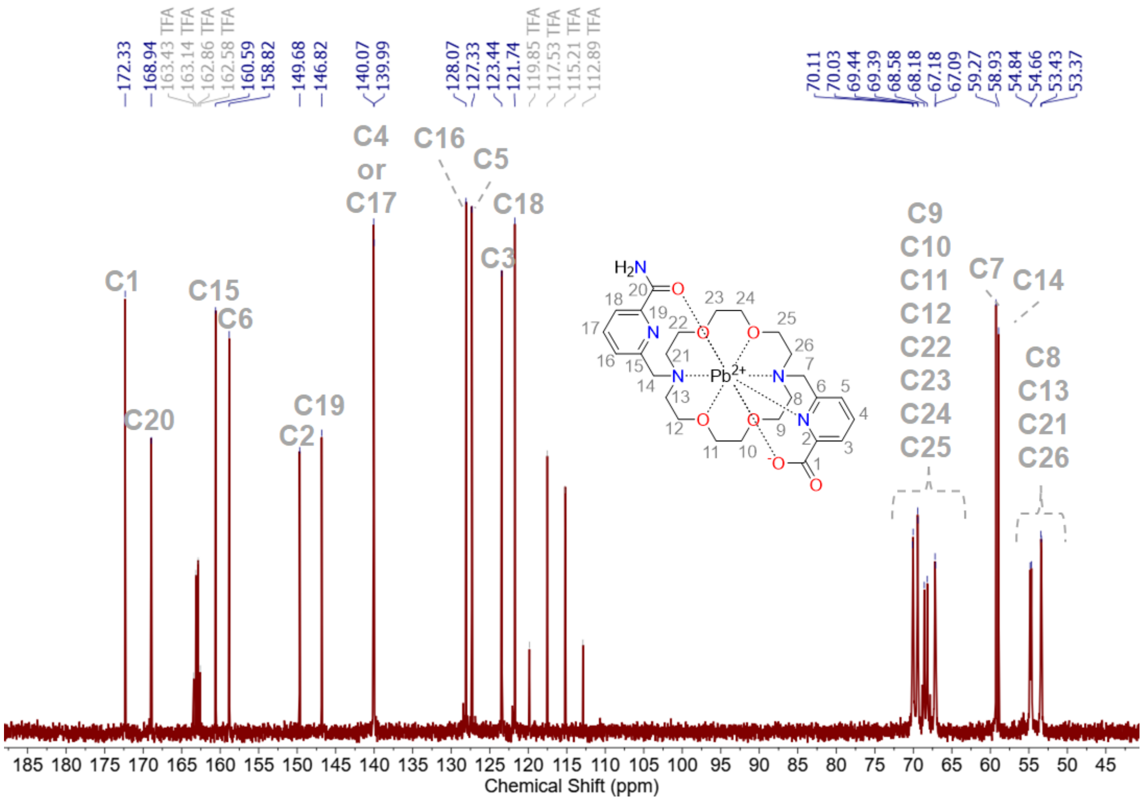


**Figure S21**: $^{13}$C NMR spectrum of complex **[Pb(macropapam)]$^{+}$** (126 MHz, $D_2O$, pD = 6, 298 K). TFA indicates trifluoroacetic acid.

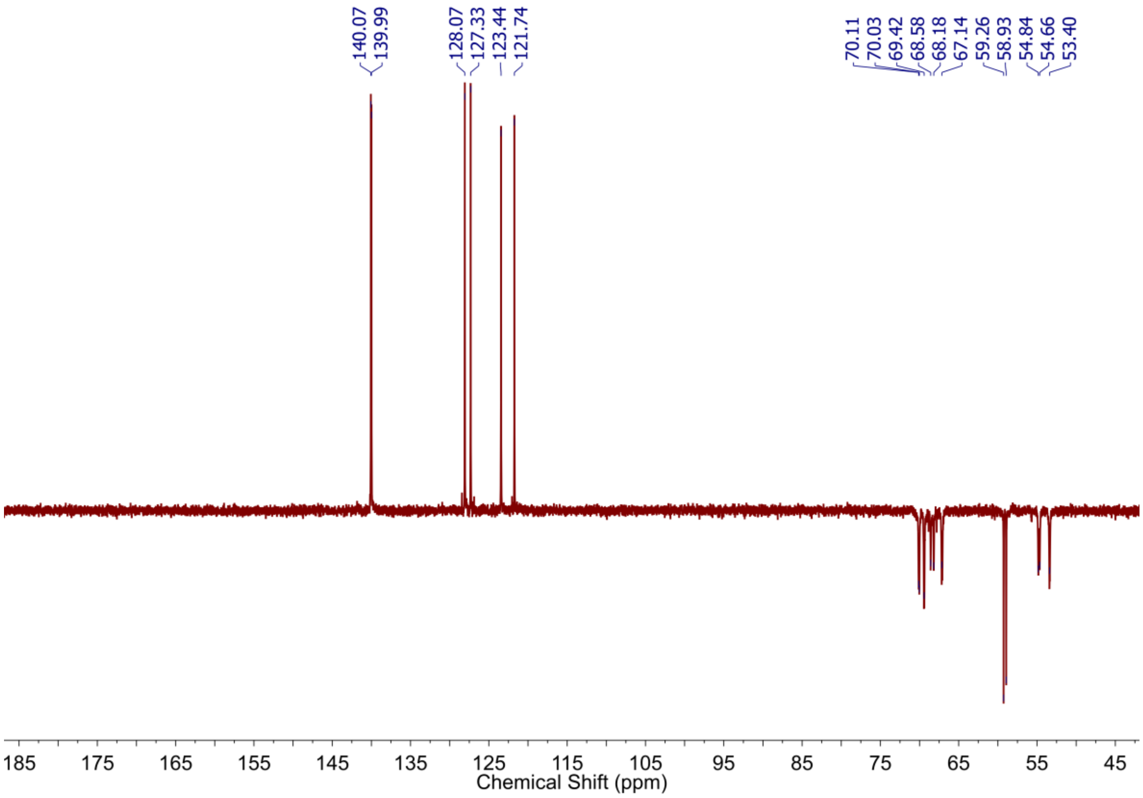


**Figure S22**: $^{13}$C DEPT-135 NMR spectrum of complex **[Pb(macropapam)]$^{+}$** (126 MHz, $D_2O$, pD = 6, 298 K).

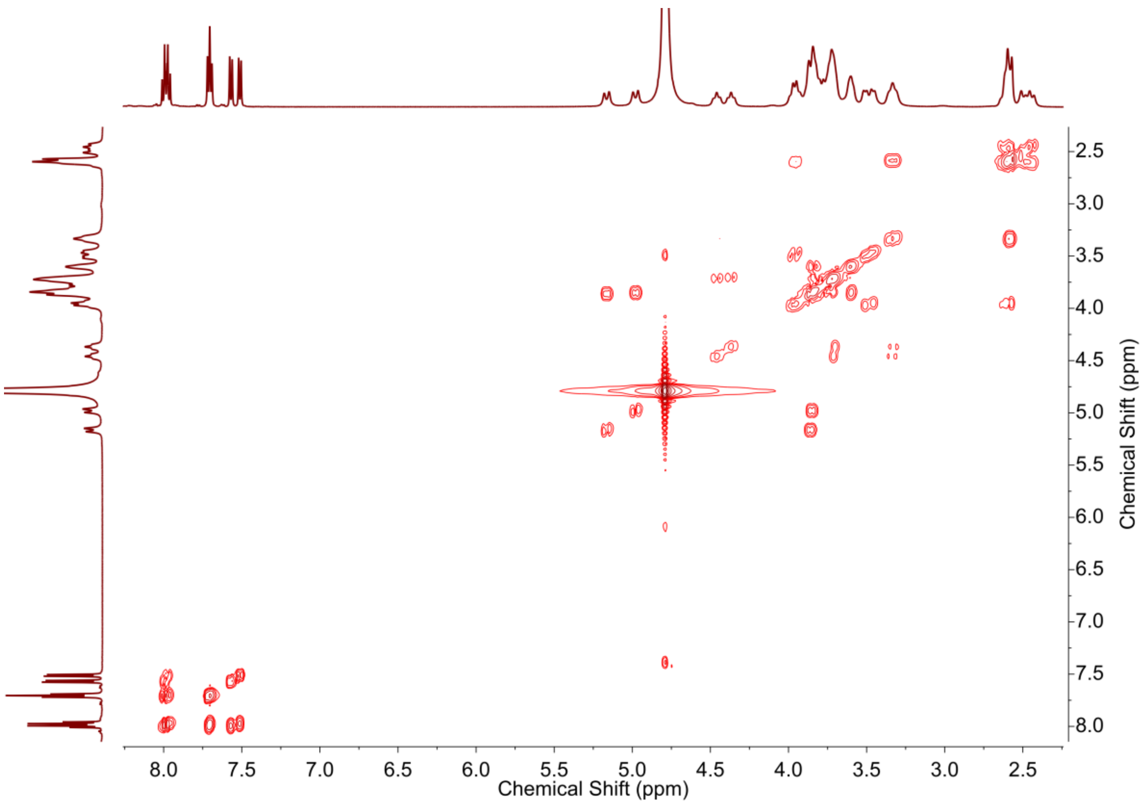


**Figure S23**: $^{1}H$-$^{1}H$ COSY NMR spectrum of complex **[Pb(macropapam)]$^{+}$** (500 MHz, $D_2O$, pD = 6, 298 K).

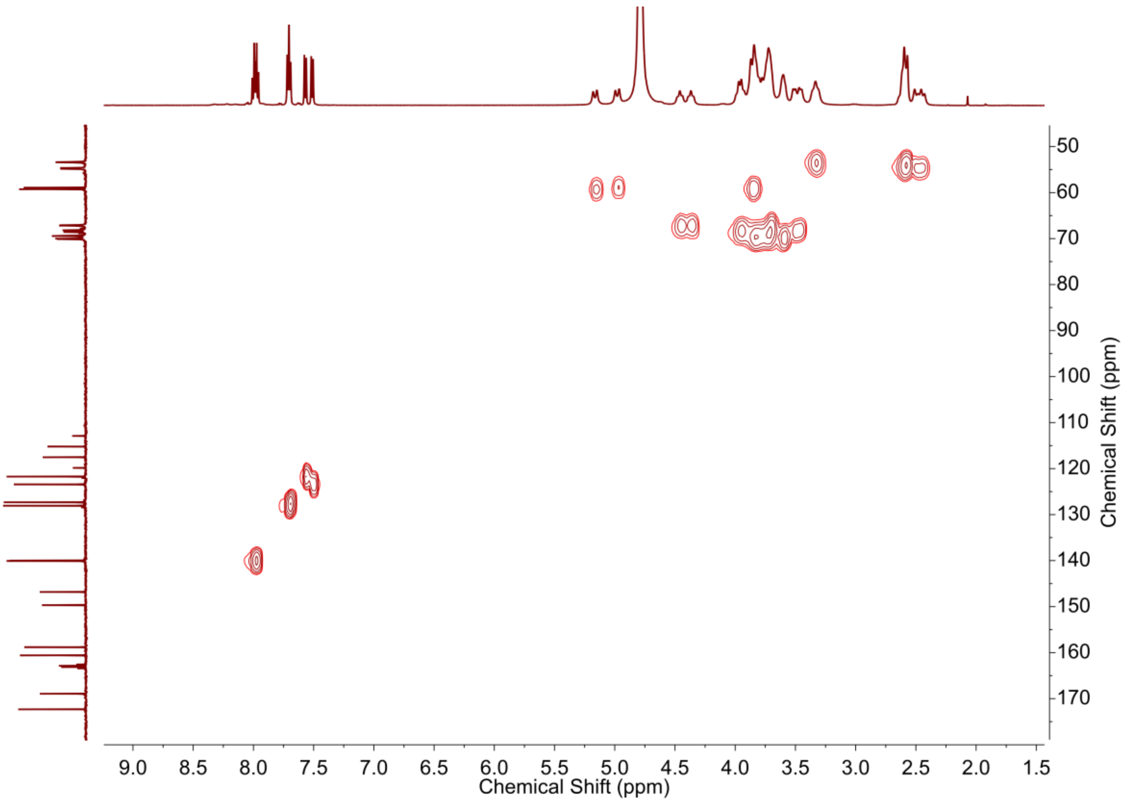


**Figure S24**: $^{1}H$-$^{13}C$ HSQC NMR spectrum of complex **[Pb(macropapam)]$^{+}$** (500-126 MHz, $D_2O$, pD = 6, 298 K).

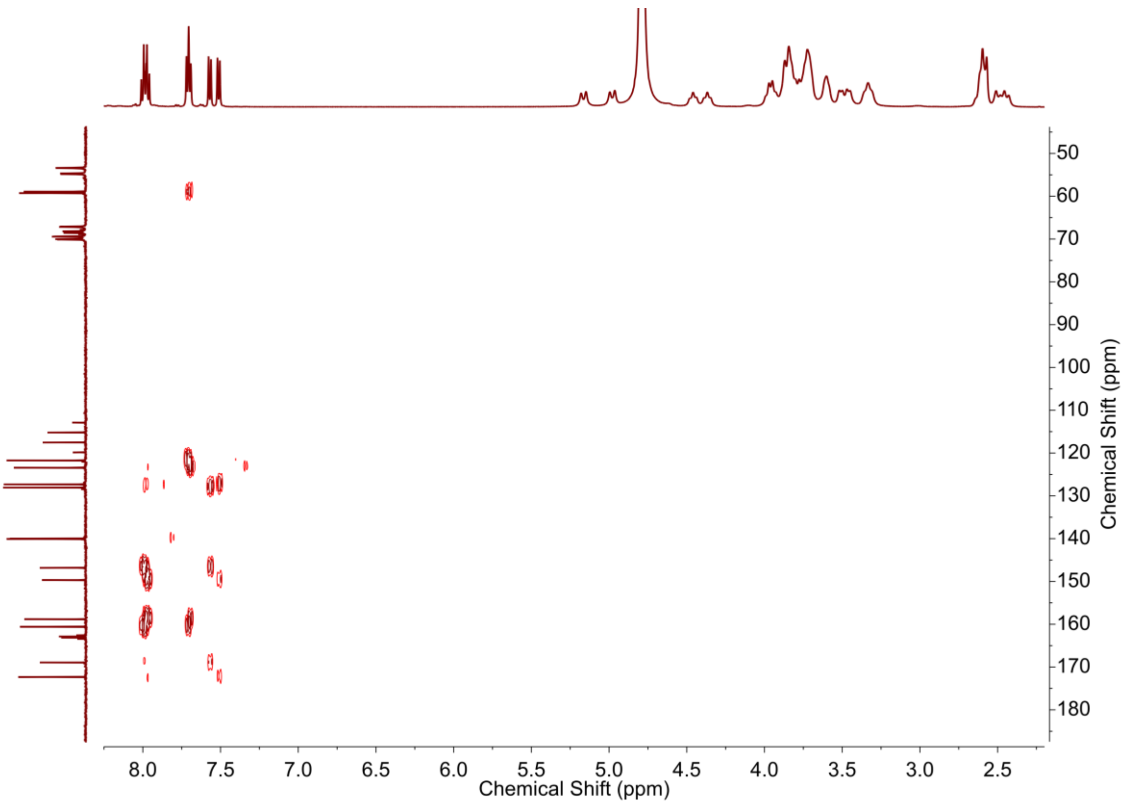


**Figure S25**: $^{1}H$-$^{13}C$ HMBC NMR spectrum of complex **[Pb(macropapam)]$^{+}$** (500-126 MHz, $D_2O$, pD = 6, 298 K).

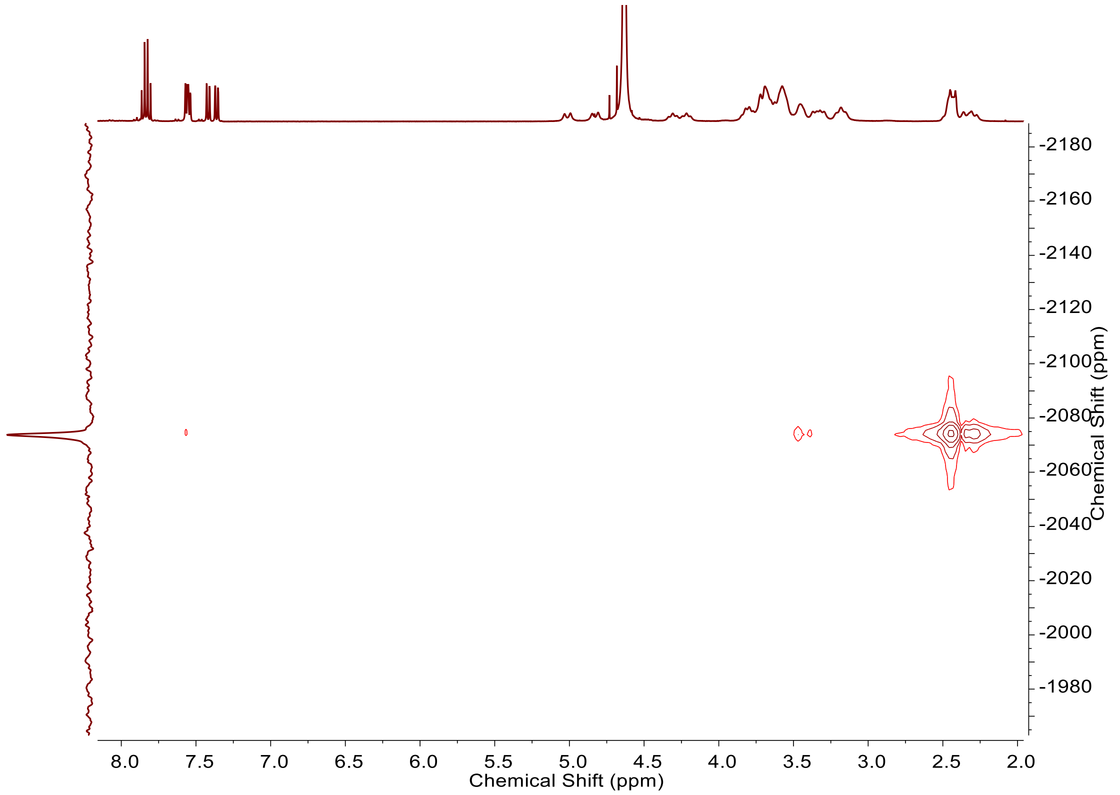


**Figure S26**: $^{1}H$-$^{207}Pb$ HMQC NMR spectrum of complex **[Pb(macropapam)]$^{+}$** (400-84 MHz, $D_2O$, pD = 6, 298 K).

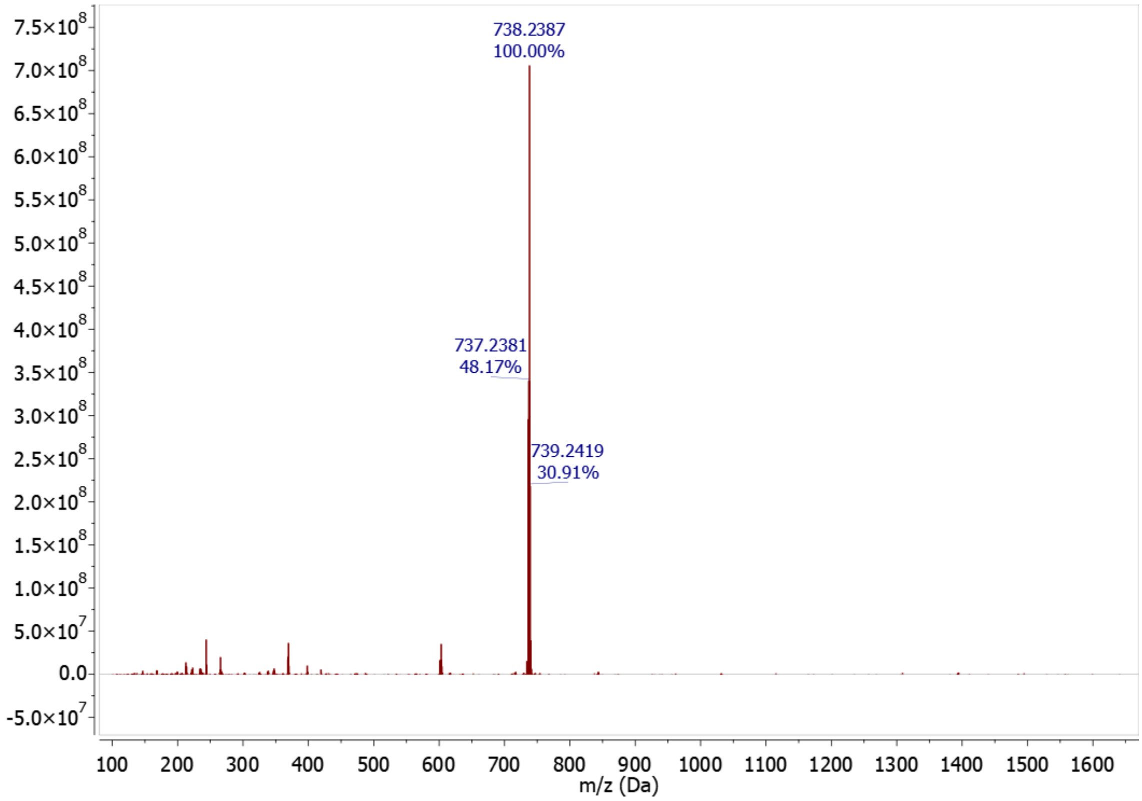


**Figure S27**: Experimental high resolution mass spectrum ($ESI^+$) of compound **[Pb(macropapam)]**$^+$.

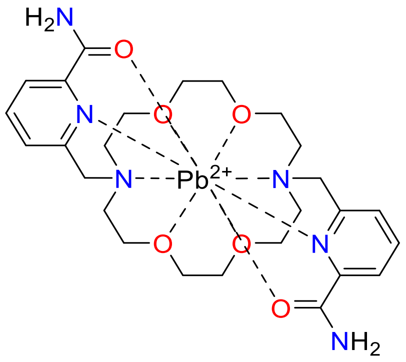


**Scheme S5**: Structure of [Pb(macropam)]$^{2+}$

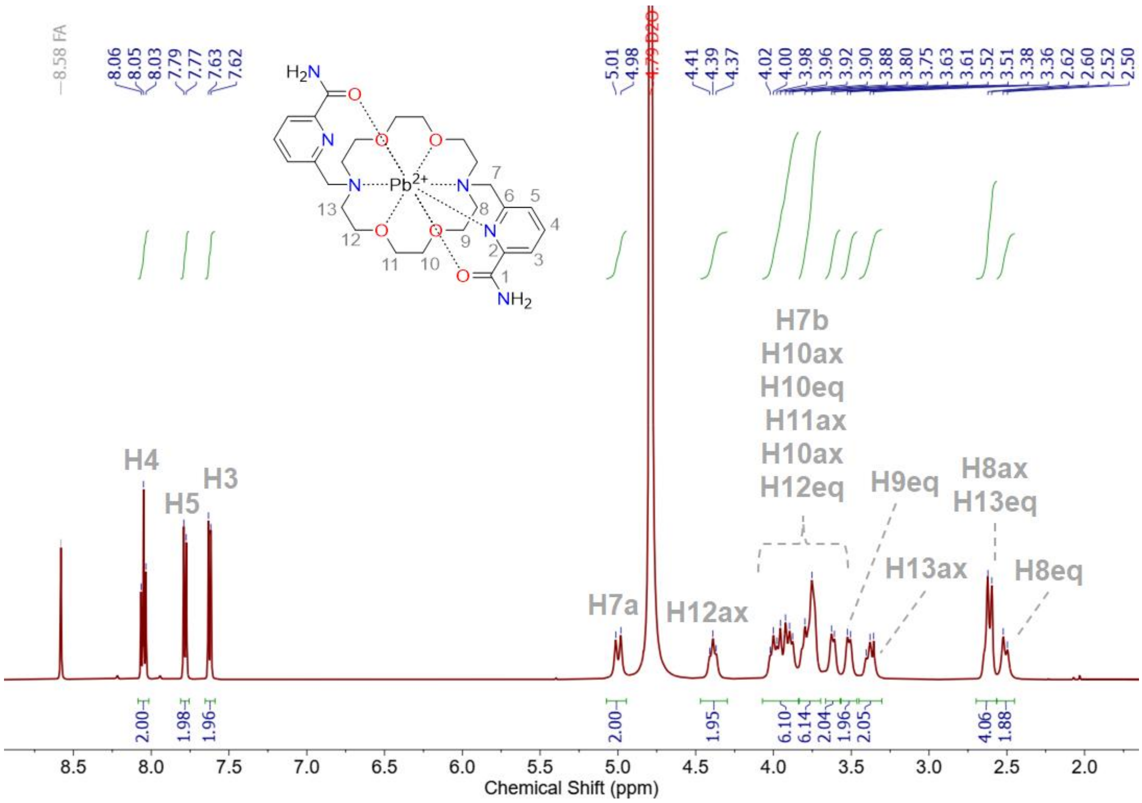


**Figure S28**: $^{1}H$ NMR spectrum of complex **[Pb(macropam)]$^{2+}$** (500 MHz, $D_2O$, pD = 7, 298 K). FA indicates formic acid.

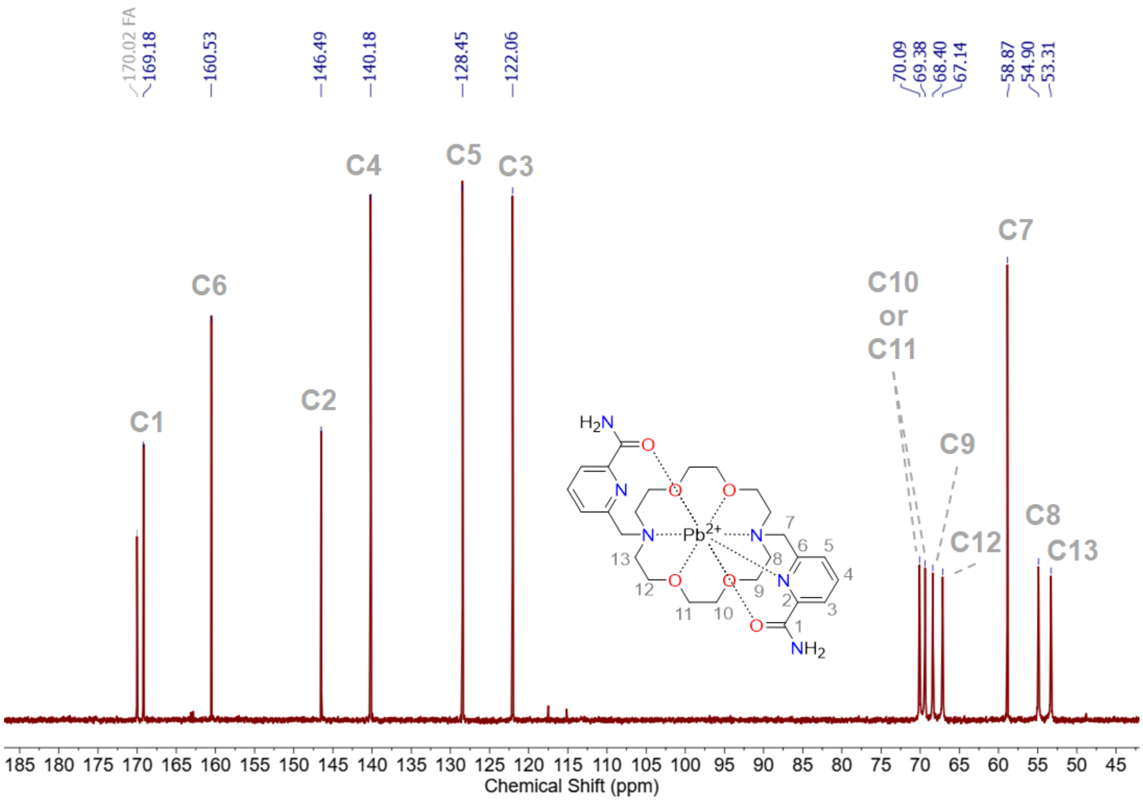


**Figure S29**: $^{13}C$ NMR spectrum of complex **[Pb(macropam)]$^{2+}$** (126 MHz, $D_2O$, pD = 7, 298 K). FA indicates formic acid.

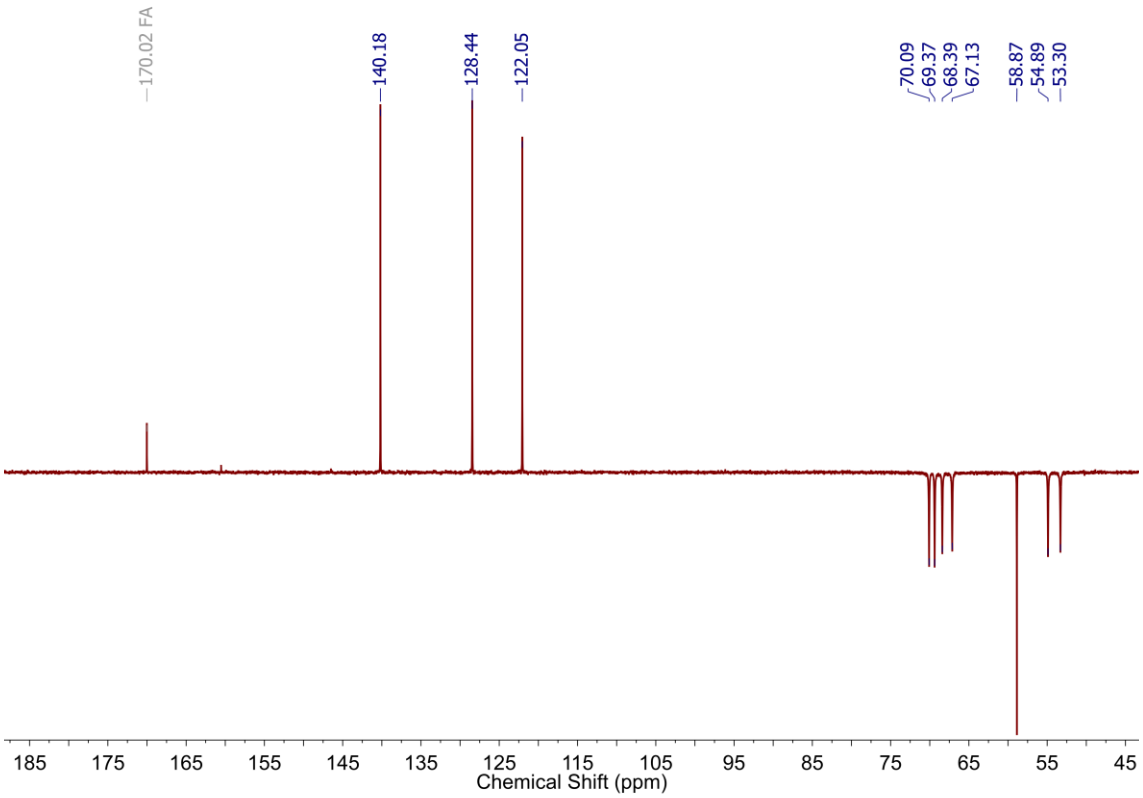


**Figure S30**: $^{13}C$ DEPT-135 NMR spectrum of complex **[Pb(macropam)]$^{2+}$** (126 MHz, $D_2O$, pD = 7, 298 K). Asterisk corresponds to a formic acid signal.

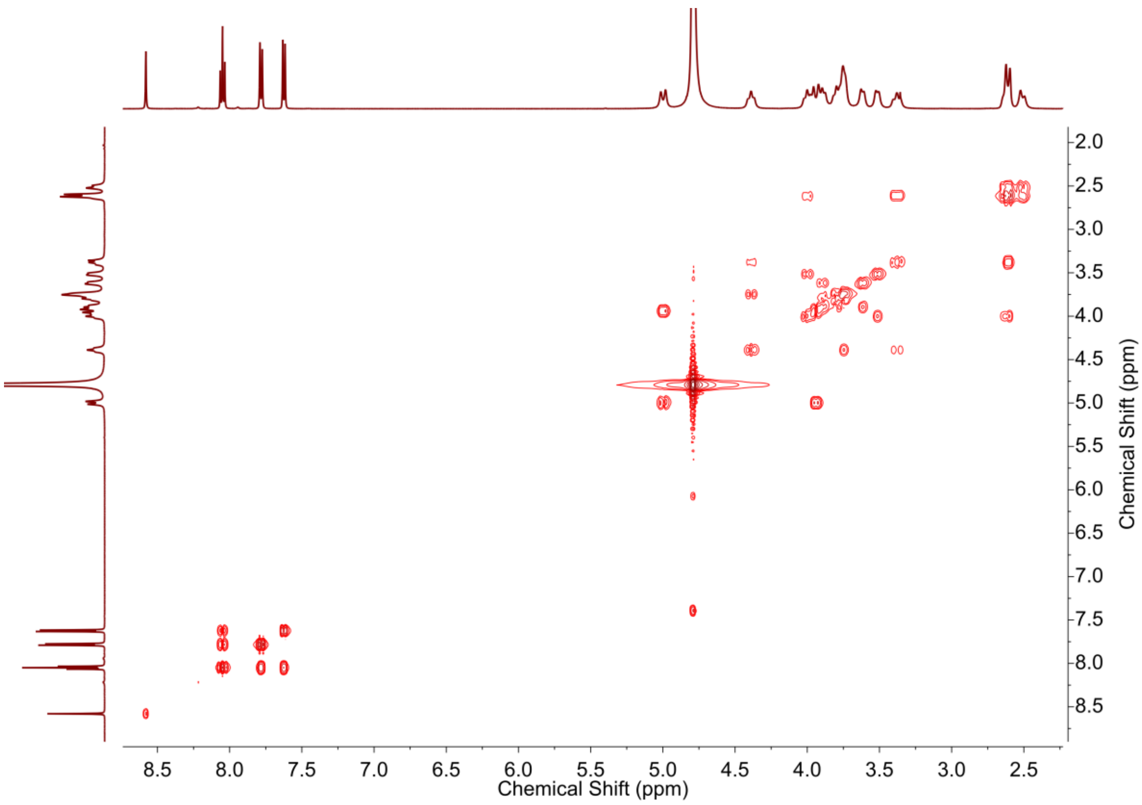


**Figure S31**: $^{1}$H-$^{1}$H COSY NMR spectrum of complex **[Pb(macropam)]$^{2+}$** (500 MHz, $D_2O$, pD= 7, 298 K).

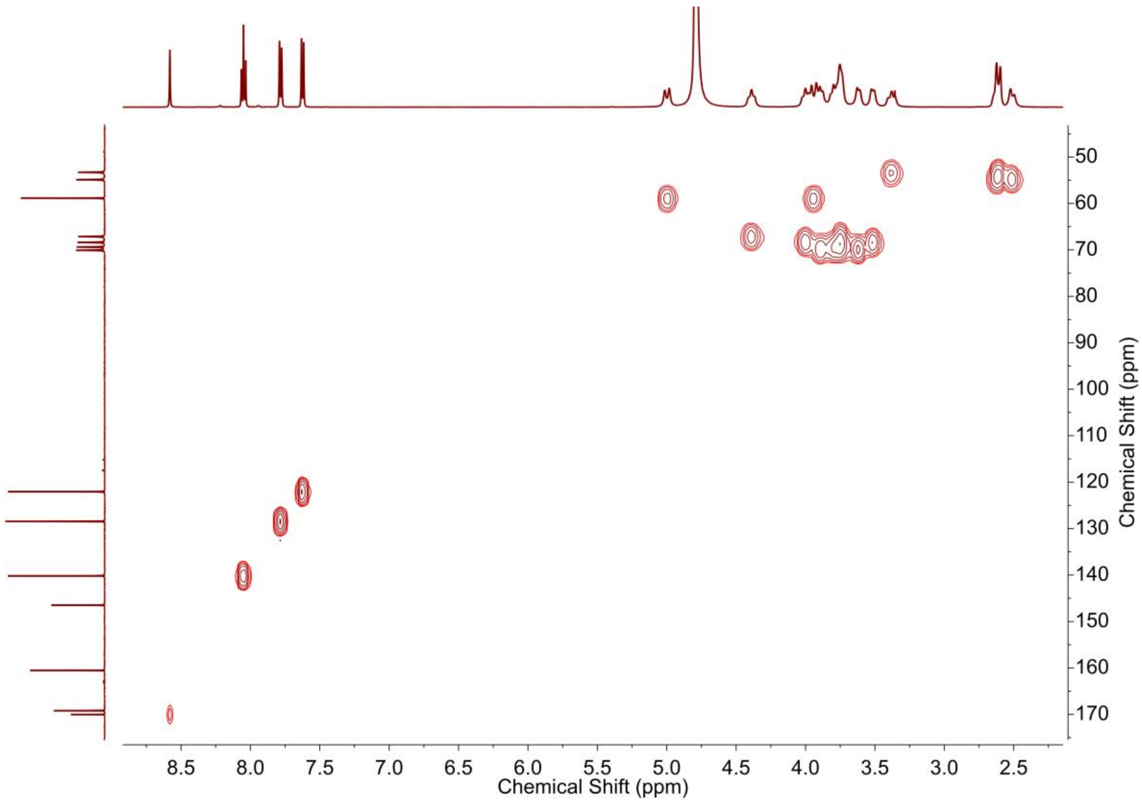


**Figure S32**: $^{1}$H-$^{13}$C HSQC NMR spectrum of complex **[Pb(macropam)]$^{2+}$** (500-126 MHz, $D_2O$, pD = 7, 298 K).

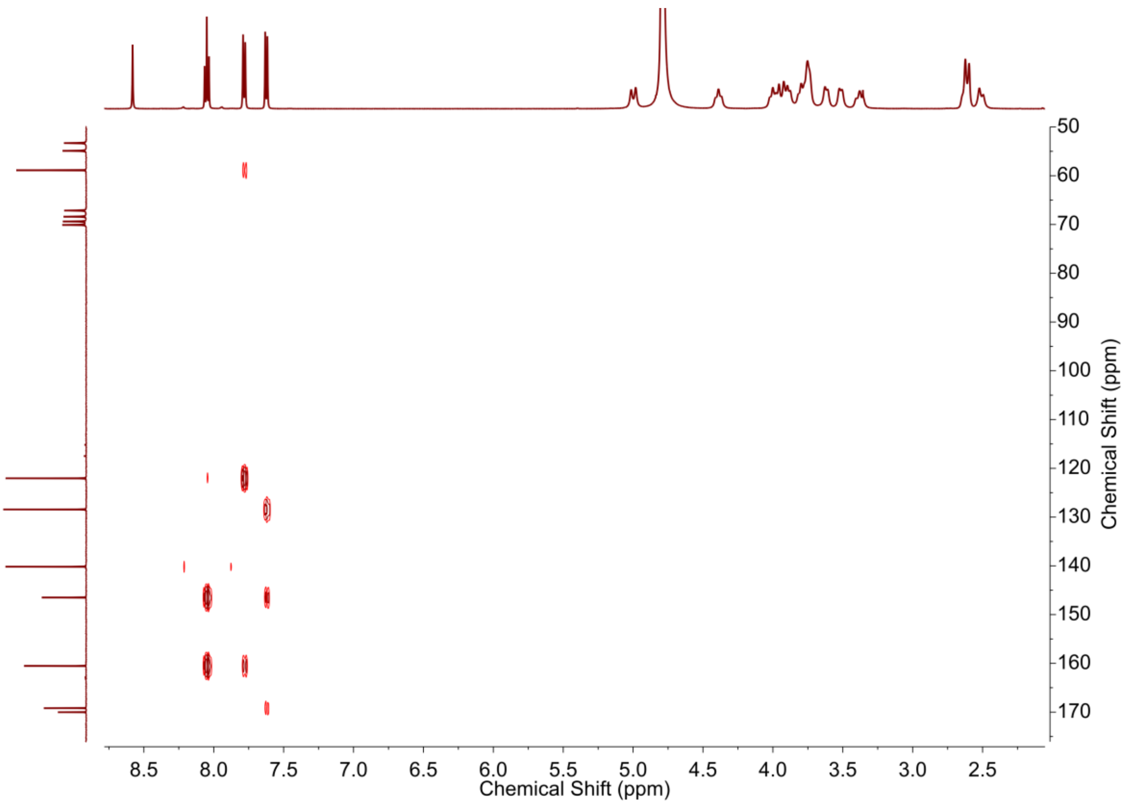


**Figure S33**: $^{1}H$-$^{13}C$ HMBC NMR spectrum of complex **[Pb(macropam)]$^{2+}$** (500-126 MHz, $D_2O$, pD = 7, 298 K).

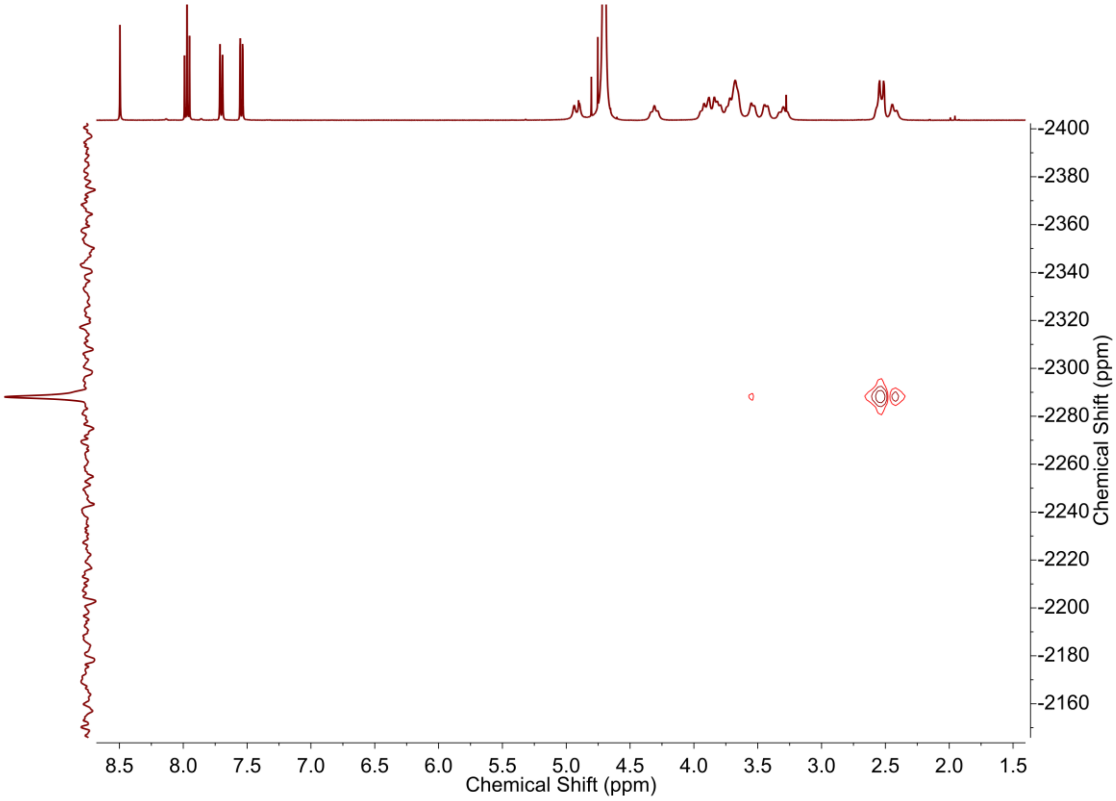


**Figure S34**: $^{1}H$-$^{207}Pb$ HMQC NMR spectrum of complex **[Pb(macropam)]$^{2+}$** (400-84 MHz, $D_2O$, pD = 7, 298 K).

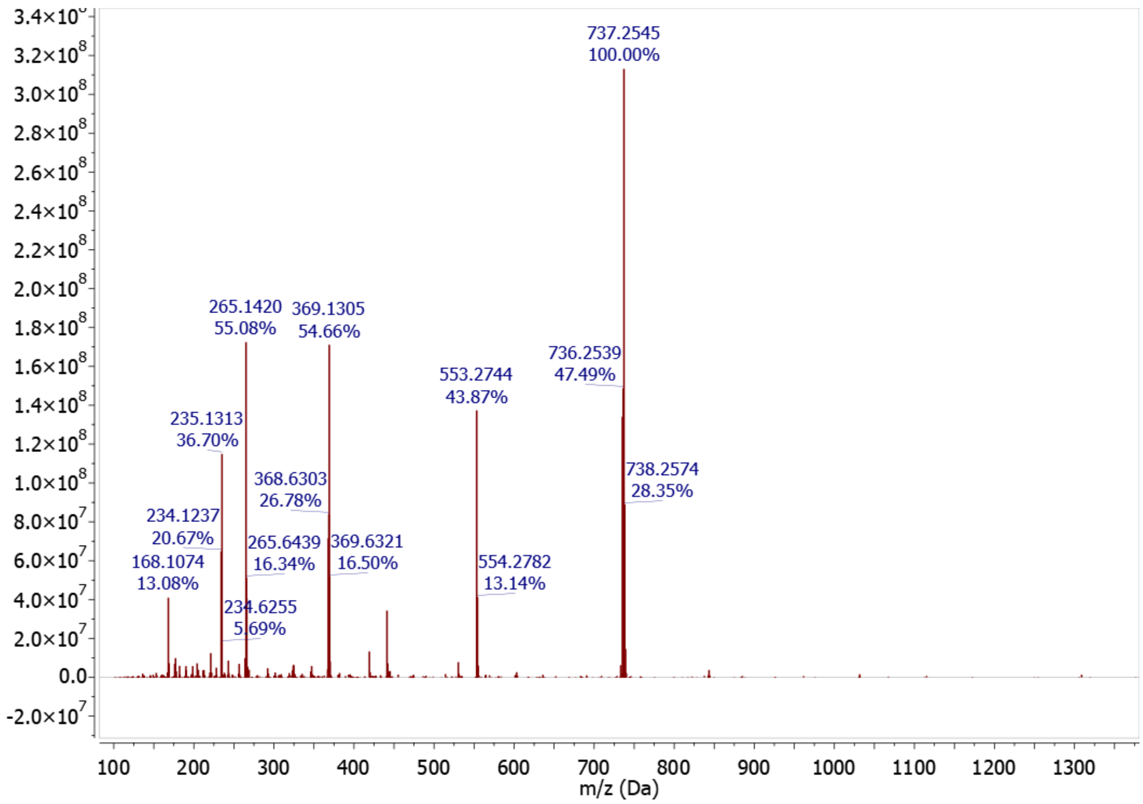


**Figure S35**: Experimental high resolution mass spectrum (ESI$^+$) of compound **[Pb(macropam)]$^{2+}$**. This spectrum displays signals corresponding to the free ligand as well, though in the NMR spectra there is no evidence of other species, indicating that this is likely a process occurring in the MS.

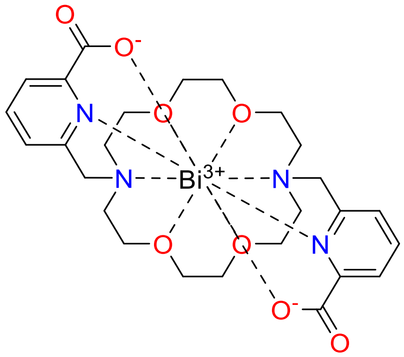


**Scheme S6**: Structure of $[Bi(macropa)]^+$

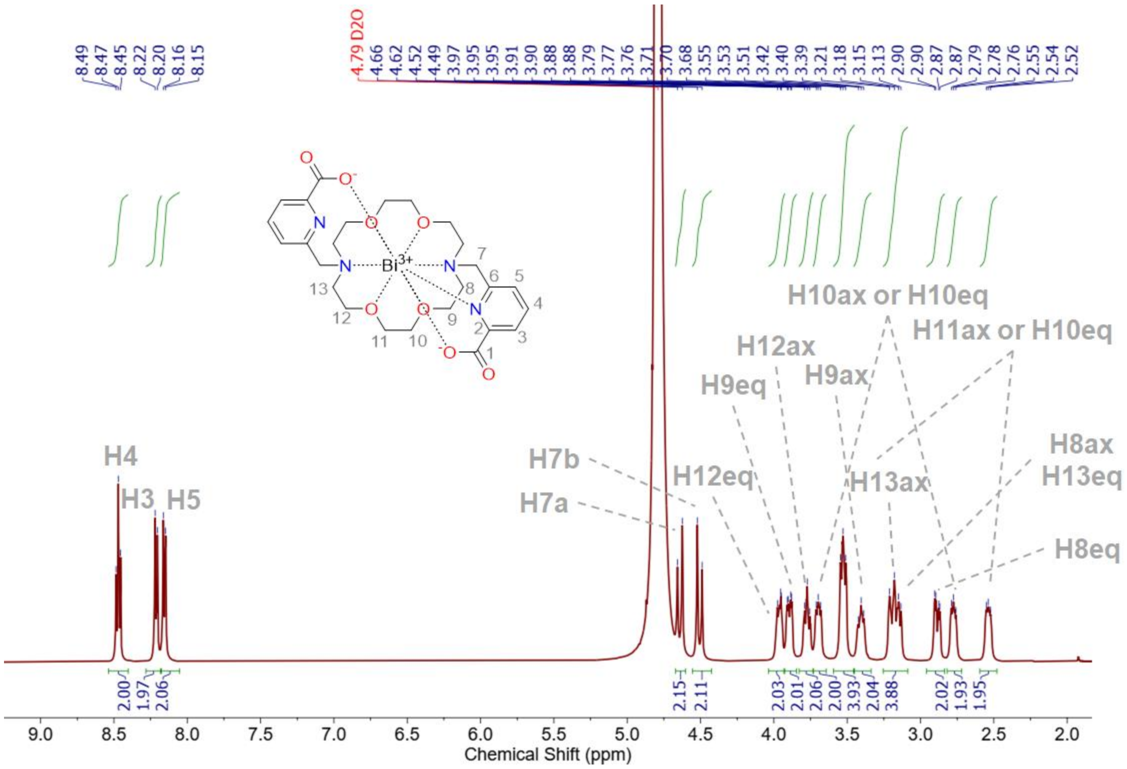


**Figure S36**: $^{1}$H NMR spectrum of complex **$[Bi(macropa)]^+$** (500 MHz, $D_2O$, pD = 6, 298 K).

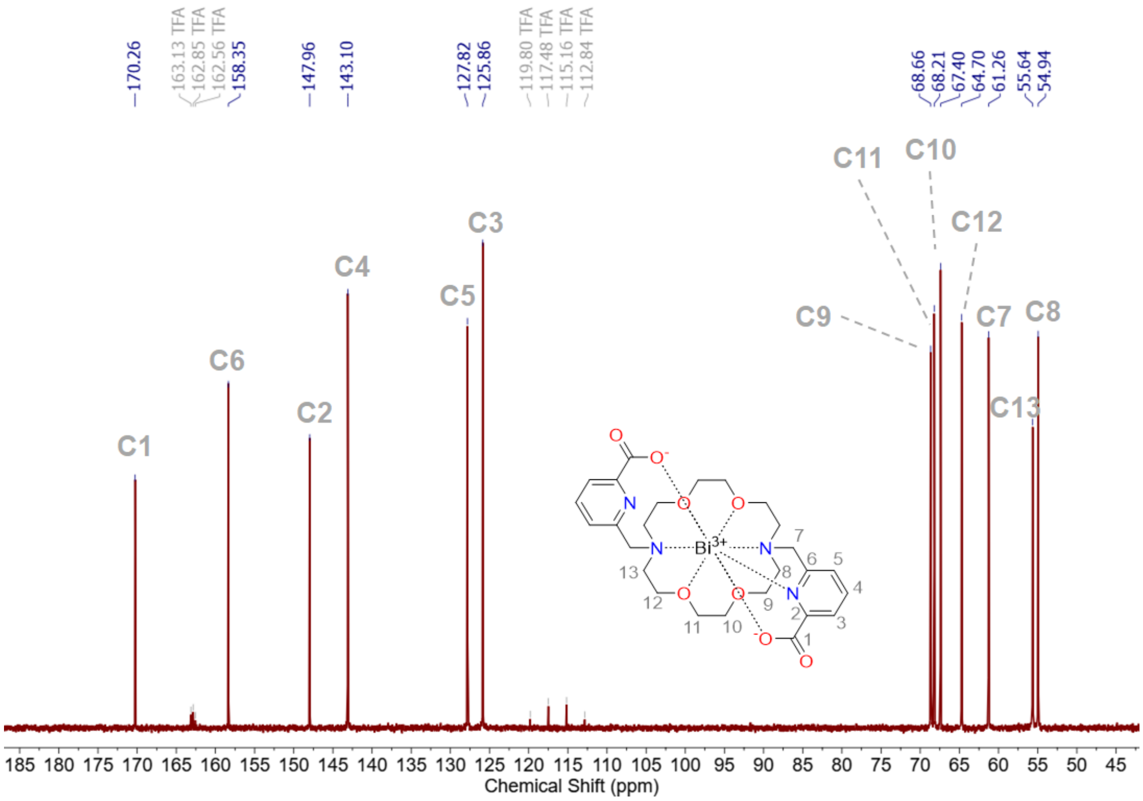


**Figure S37**: $^{13}C$ NMR spectrum of complex **[Bi(macropa)]$^+$** (126 MHz, $D_2O$, pD = 6, 298 K). TFA indicates trifluoroacetic acid.

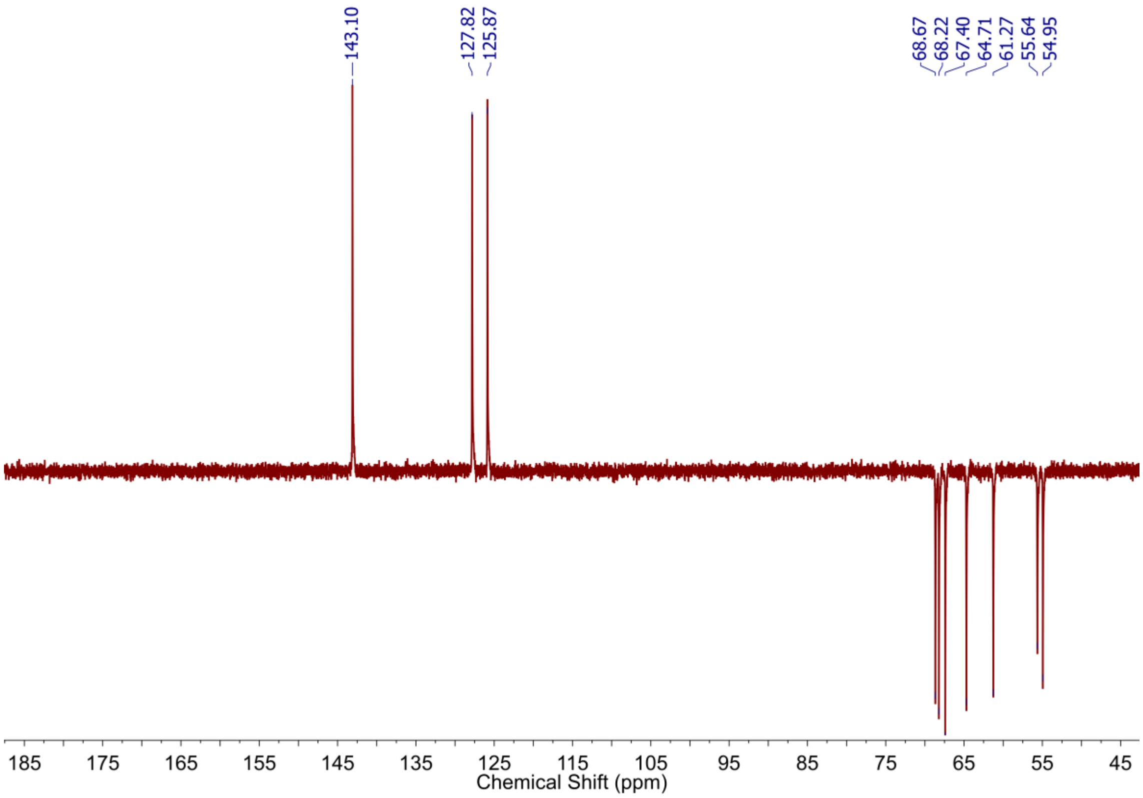


**Figure S38**: $^{13}C$ DEPT-135 NMR spectrum of complex **[Bi(macropa)]$^+$** (126 MHz, $D_2O$, pD = 6, 298 K).

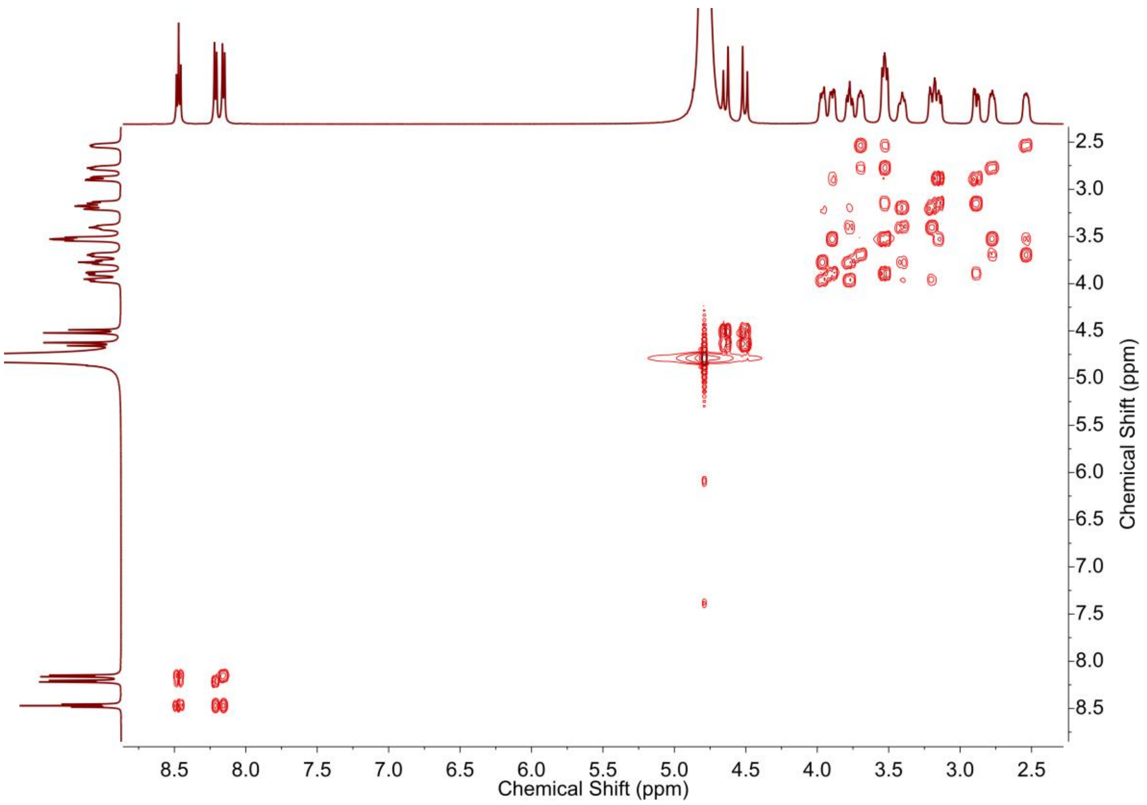


**Figure S39**: $^{1}H$-$^{1}H$ COSY NMR spectrum of complex **[Bi(macropa)]**$^{+}$ (500 MHz, $D_2O$, pD = 6, 298 K).

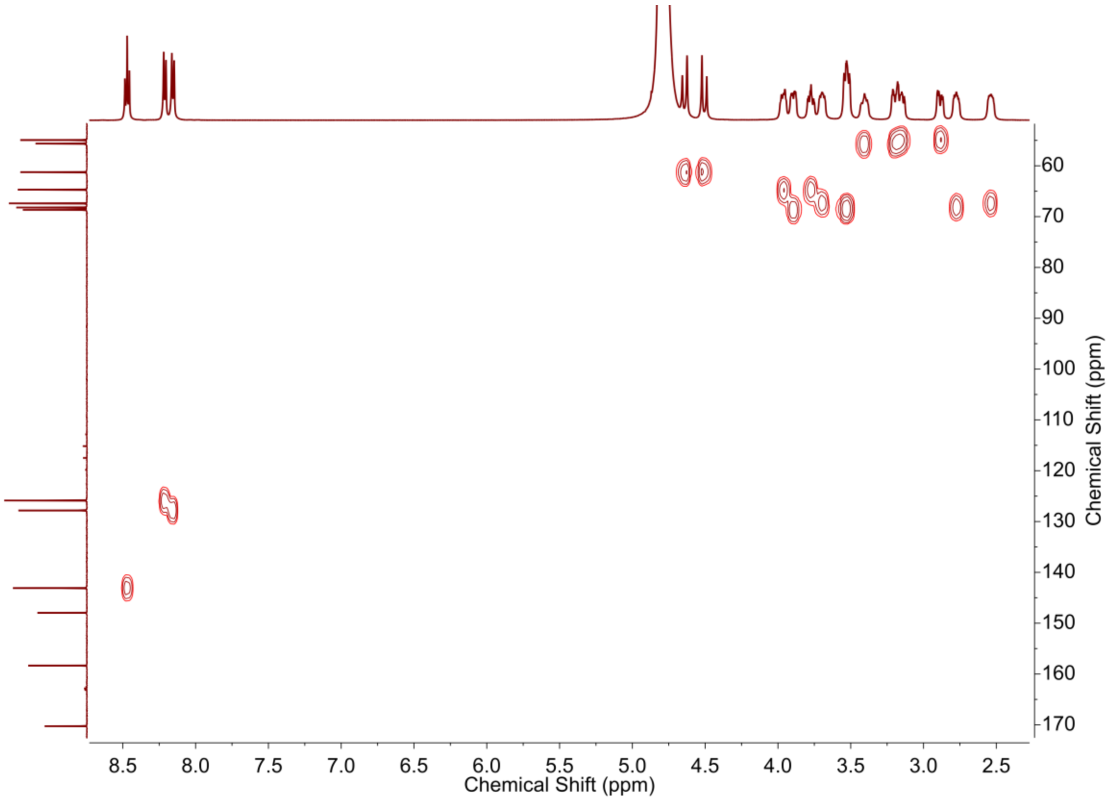


**Figure S40**: $^{1}H$-$^{13}C$ HSQC NMR spectrum of complex **[Bi(macropa)]**$^{+}$ (500-126 MHz, $D_2O$, pD = 6, 298 K).

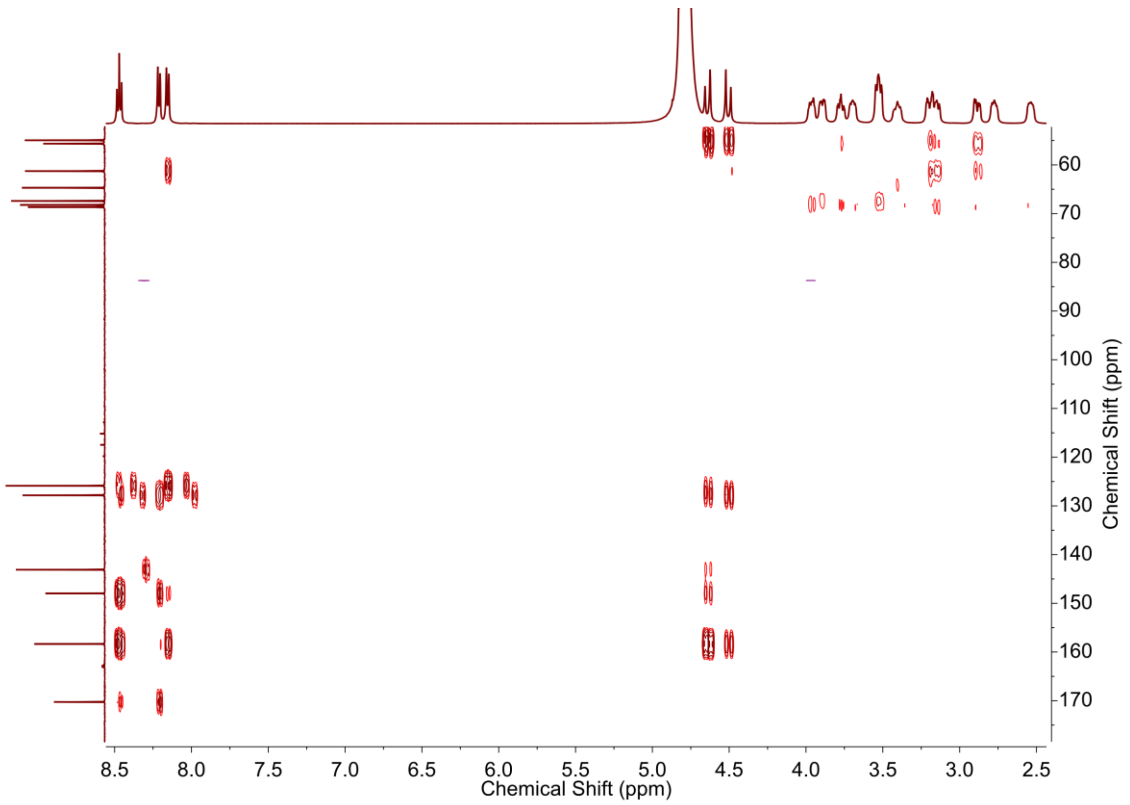


**Figure S41**: $^{1}H$-$^{13}C$ HMBC NMR spectrum of complex **[Bi(macropa)]**$^{+}$ (500-126 MHz, $D_2O$, pD = 6, 298 K).

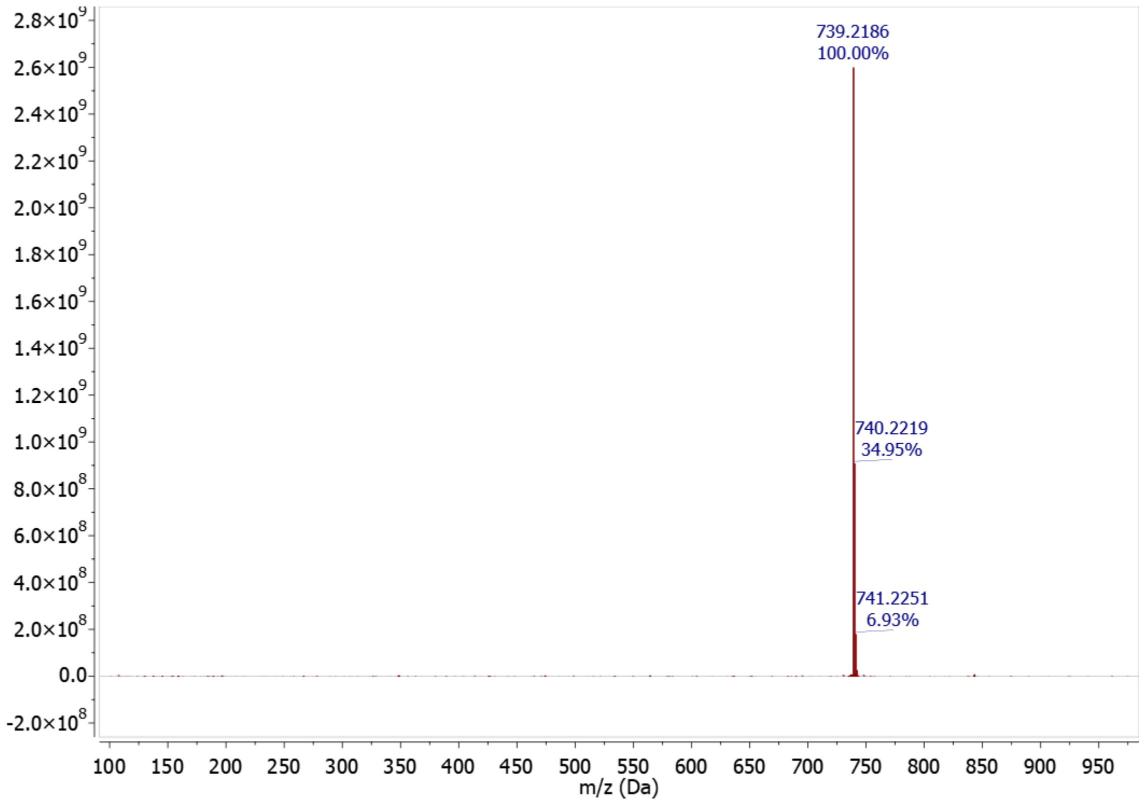


**Figure S42**: Experimental high resolution mass spectrum (ESI$^{+}$) of compound **[Bi(macropa)]**$^{+}$.

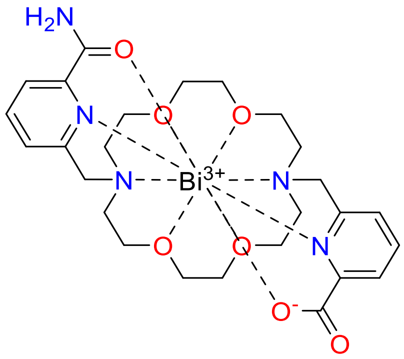


**Scheme S7**: Structure of $[Bi(macropapam)]^{2+}$

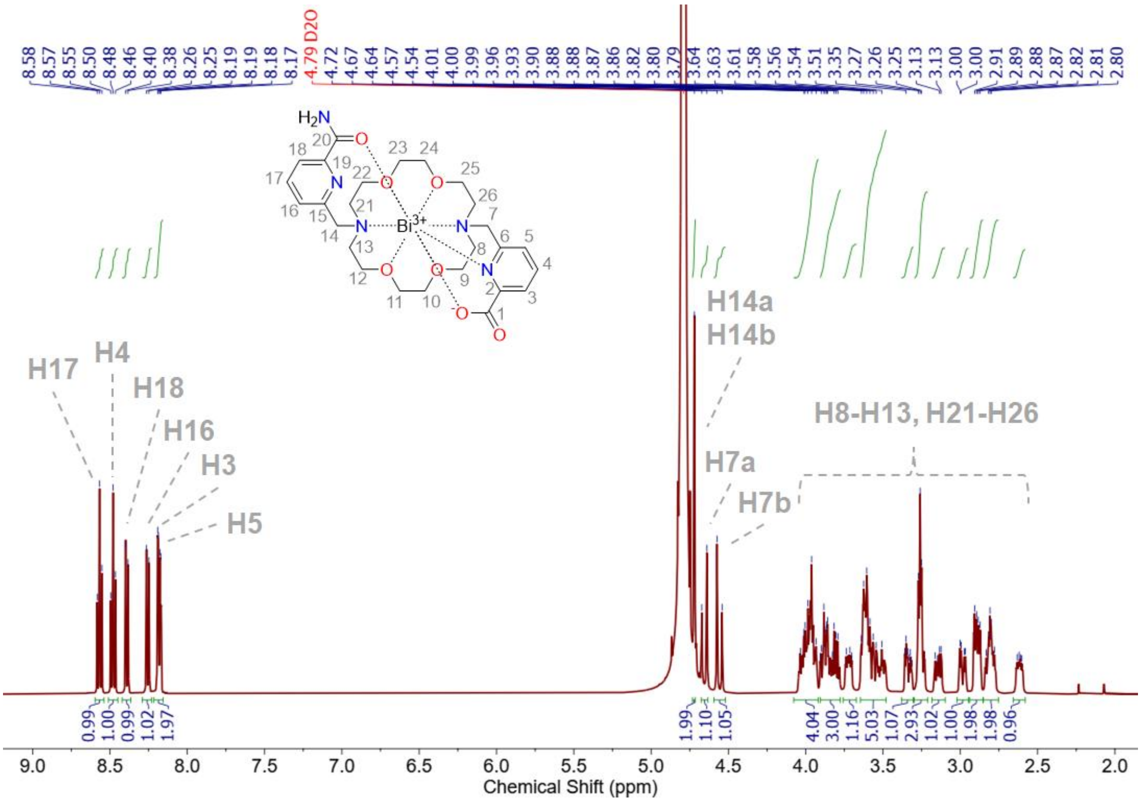


**Figure S43**: $^{1}H$ NMR spectrum of complex **$[Bi(macropapam)]^{2+}$** (500 MHz, $D_2O$, pD = 6, 298 K).

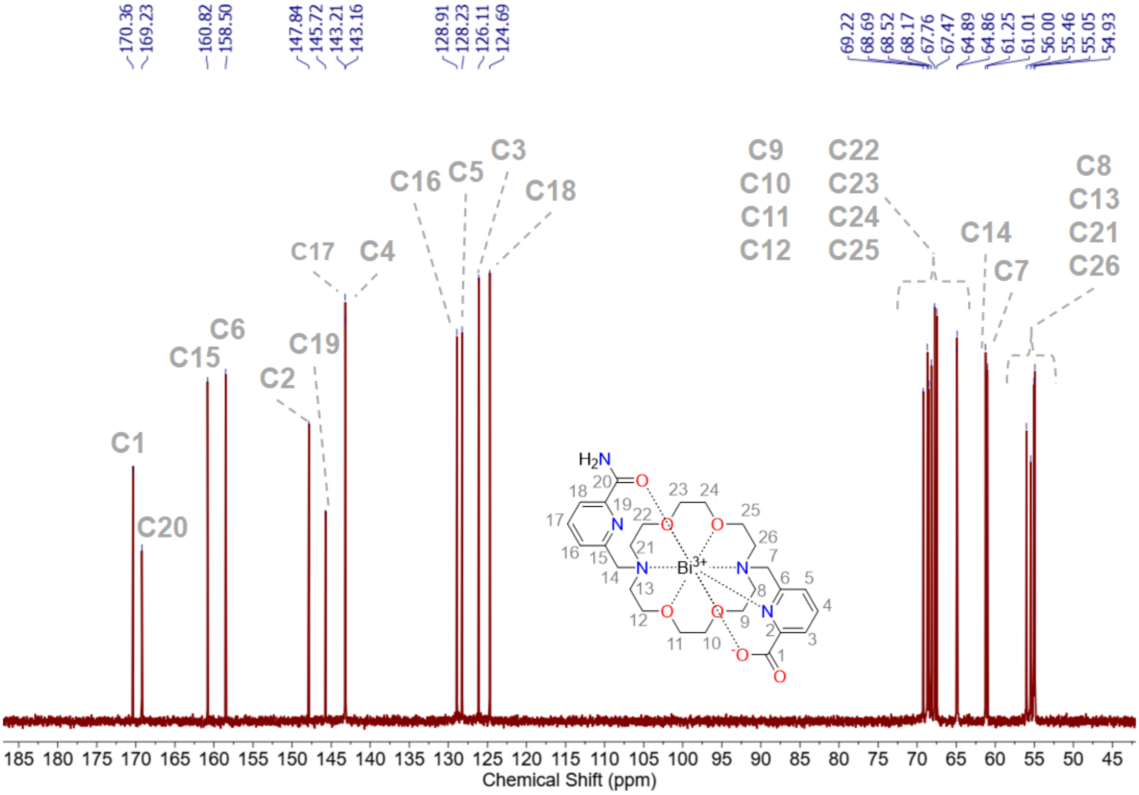


**Figure S44**: $^{13}$C NMR spectrum of complex **[Bi(macropapam)]**$^{2+}$ (126 MHz, $D_2O$, pD = 6, 298 K).

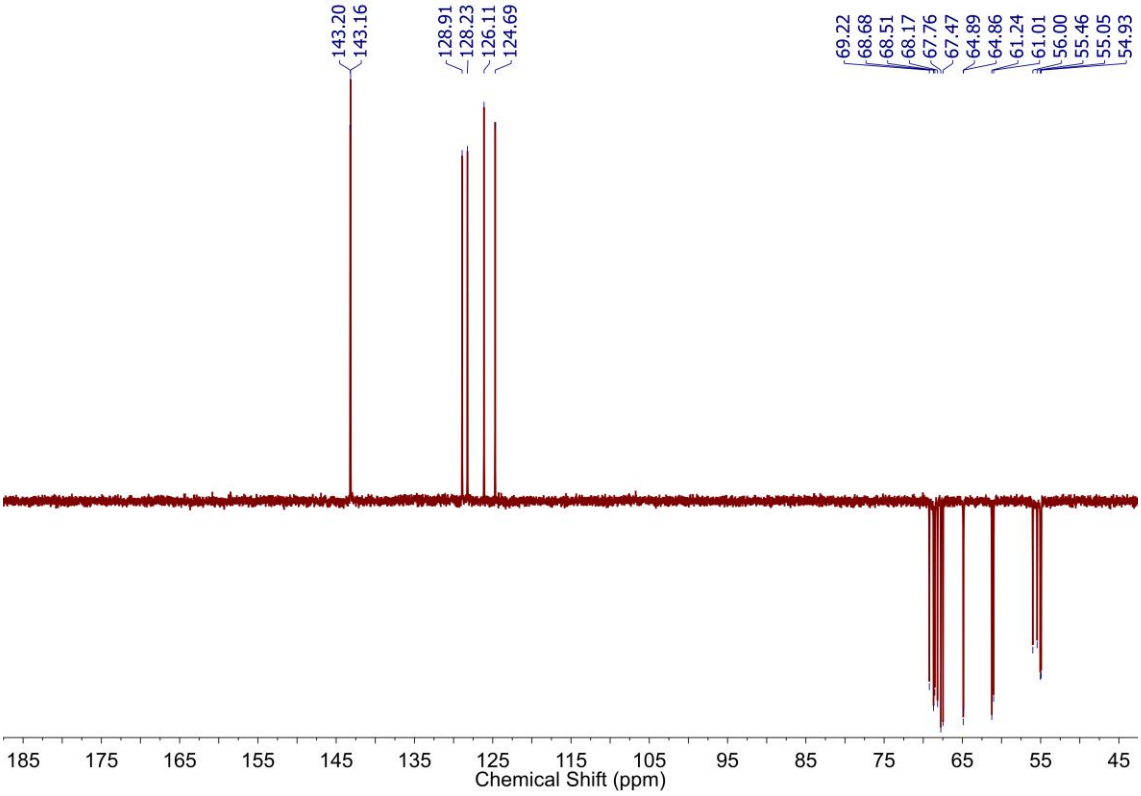


**Figure S45**: $^{13}$C DEPT-135 NMR spectrum of complex **[Bi(macropapam)]**$^{2+}$ (126 MHz, $D_2O$, pD = 6, 298 K).

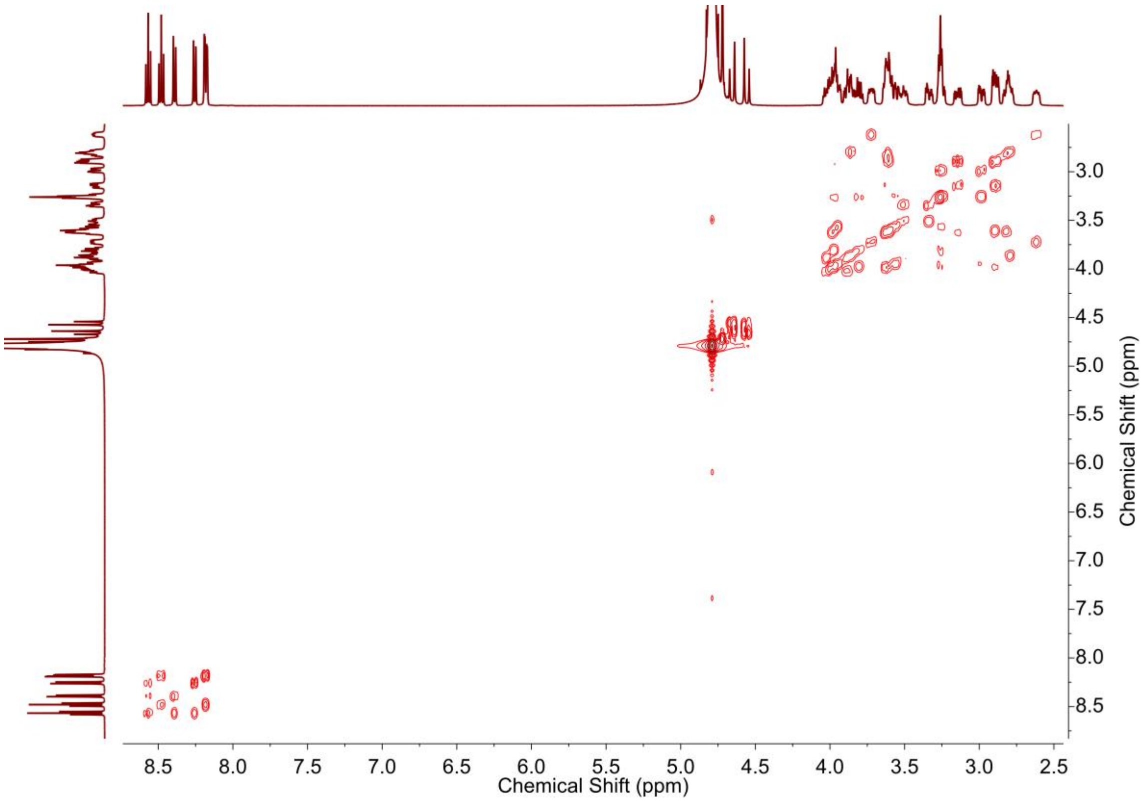


**Figure S46**: $^{1}H$-$^{1}H$ COSY NMR spectrum of complex **[Bi(macropapam)]**$^{2+}$ (500 MHz, $D_2O$, pD = 6, 298 K).

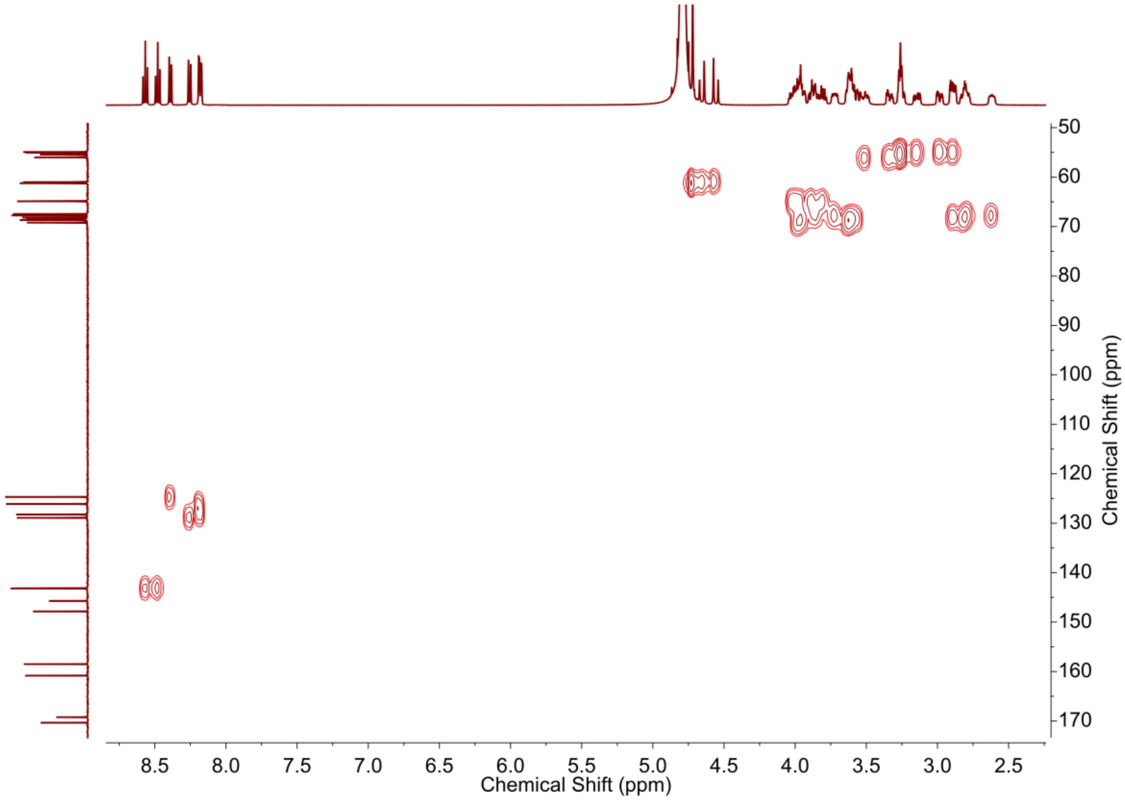


**Figure S47**: $^{1}H$-$^{13}C$ HSQC NMR spectrum of complex **[Bi(macropapam)]**$^{2+}$ (500-126 MHz, $D_2O$, pD = 6, 298 K).

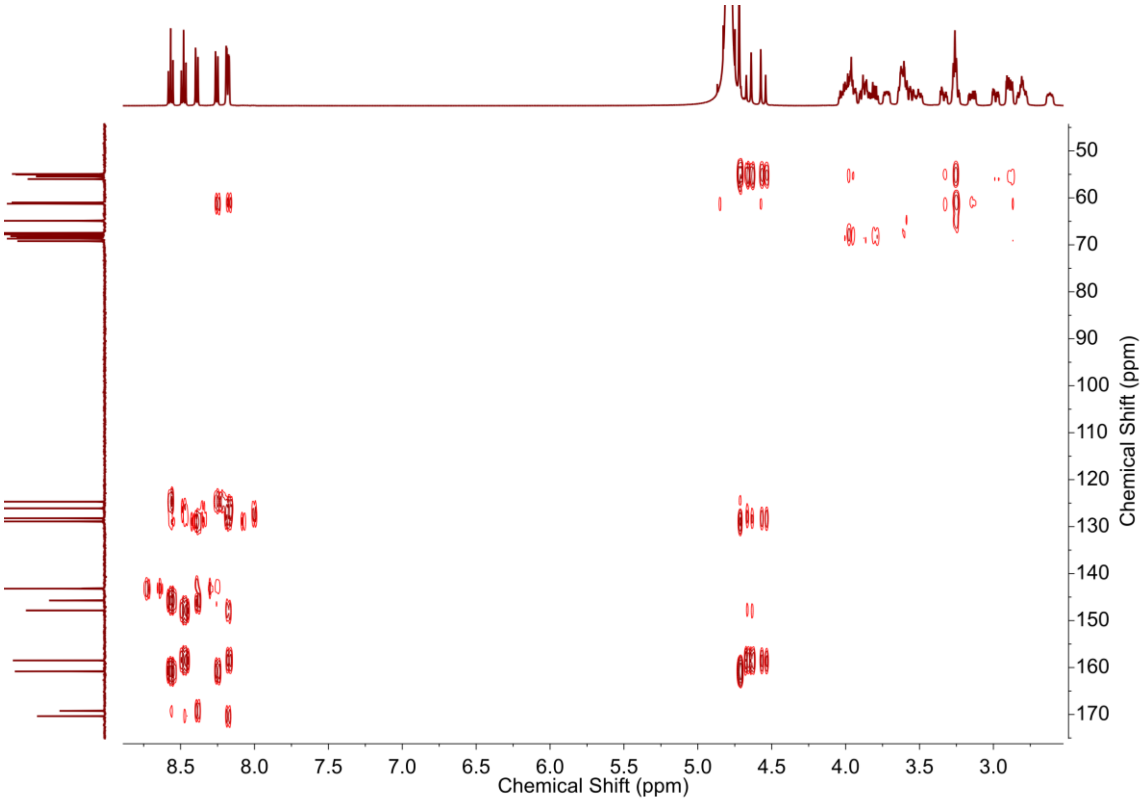


**Figure S48**: $^{1}H$-$^{13}C$ HMBC NMR spectrum of complex **[Bi(macropapam)]**$^{2+}$ (500-126 MHz, $D_2O$, pD = 6, 298 K).

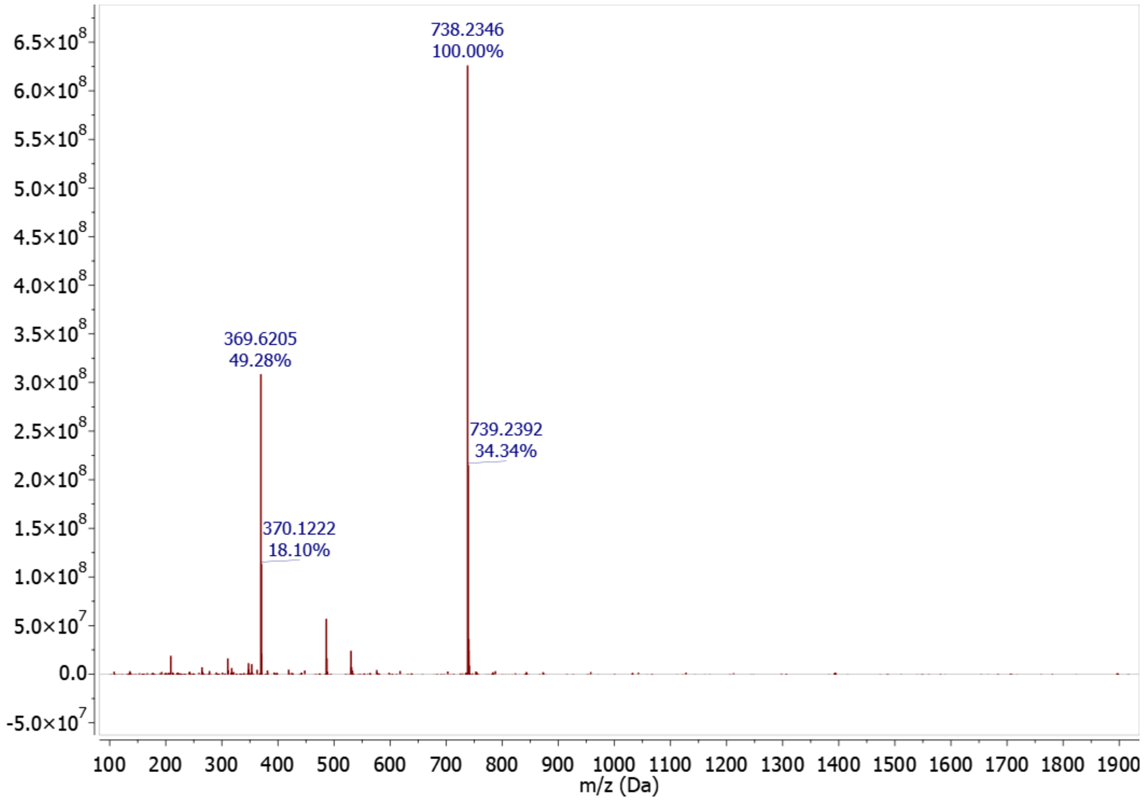


**Figure S49**: Experimental high resolution mass spectrum (ESI$^{+}$) of compound **[Bi(macropapam)]**$^{+}$.

**Table S9**: Crystal data and structure refinement for $[H_2macropam](PF_6)_2·H_2O$, $[Bi(macropa)]PF_6$, $[Bi(macropapam)](PF_6)_2·2H_2O$ and $[Pb(macropam)](PF_6)_2·H_2O$.

| | **$[H_2macropam](PF_6)_2·H_2O$** | **$[Bi(macropa)]PF_6·2H_2O$** | **$[Bi(macropapam)](PF_6)_2·2H_2O$** | **$[Pb(macropam)](PF_6)_2·H_2O$** |
|---|---|---|---|---|
| Empirical formula | $C_{26}H_{42}F_{12}N_6O_7P_2$ | $C_{26}H_{38}F_6N_4O_{10}PBi$ | $C_{26}H_{40}F_{12}N_5O_9P_2Bi$ | $C_{26}H_{40}F_{12}N_6O_7P_2Pb$ |
| Molecular weight MW | 840.59 | 920.55 | 1065.55 | 1045.77 |
| Crystal system | Monoclinic | Monoclinic | Triclinic | Monoclinic |
| Space group | C2/c | C2/c | P-1 | C2/c |
| a/Å | 25.613(2) | 20.5086(17) | 10.9636(5) | 24.0586(10) |
| b/Å | 13.1961(10) | 8.1570(7) | 12.9614(7) | 11.8110(5) |
| c/Å | 10.3678(9) | 18.4984(14) | 14.2373(7) | 25.4967(9) |
| α/º | | | 85.047(2) | |
| β/º | 90.147(3) | 94.821(3)v | 75.603(2) | 96.7570(10) |
| γ/º | | | 65.788(2) | |
| Volume ($Å^3$) | 3504.2(5) | 3083.6(4) | 1786.98(16) | 7194.7(5) |
| Z | 4 | 4 | 2 | 8 |
| $\rho_{calc}$ ($g/cm^3$) | 1.593 | 1.983 | 1.980 | 1.931 |
| μ ($mm^{-1}$) | 0.241 | 5.869 | 5.143 | 4.893 |
| θ range | 1.964º - 26.419º | 2.21º - 28.333º | 2.11º - 28.348º | 1.923º - 28.313º |
| $R_{int}$ | 0.0599 | 0.0482 | 0.0290 | 0.0439 |
| Measured reflections | 35583 | 51952 | 85313 | 90109 |
| Independent reflections / unique (I >2σ (I) | 3598 / 3268 | 3841/ 3729 | 8925/7935 | 8935/6975 |
| Goodness-of-fit on $F^2$ | 1.095 | 1.092 | 1.065 | 1.064 |
| $R_1$ | 0.0377 | 0.0142 | 0.0392 | 0.0499 |
| $wR_2$ (all data) | 0.0935 | 0.0345 | 0879 | 0.1214 |
| Larg. diff. peak and hole ($eÅ^{-3}$) | 0.26 and -0.28 | 0.60 and -0.99 | 1.98 and -1.82 | 6.46 and -1.23 |

**Example of a Gaussian Input File for Optimization of a Pb(II) Complex**

```
%chk=Pbmacropa_opt_F1.chk
# rTPSSh/gen pseudo=cards scf=tight
scrf=(pcm,solvent=water) opt freq=noraman integral=ultrafinegrid

Pbmacropa_opt_F1

 0 1
C      -3.71877800    1.30088100    0.52789800
N      -2.67010300    0.63221300    1.33647800
O      -2.13228000    1.72662900   -1.23838100
C      -3.47348900    1.27921000   -0.97647000
C      -2.46053600    1.35778000    2.61407800
C      -1.72273200    2.68071800    2.42369900
O      -0.37560100    2.50558000    1.94965400
C       0.58847900    2.29061300    2.99622700
C       1.97158900    2.52002600    2.42449400
O       2.13223400    1.72667200    1.23837900
O       0.37553600    2.50560000   -1.94964600
C      -0.58853800    2.29062200   -2.99622200
C      -1.97165300    2.52000000   -2.42448700
N       2.67007800    0.63227700   -1.33648300
C       2.46049600    1.35784900   -2.61407800
C       1.72266400    2.68077000   -2.42369000
C       3.47345300    1.27928000    0.97646900
C       3.71874200    1.30095700   -0.52790000
C      -3.00917700   -0.78519400    1.61866900
C       3.00917500   -0.78512200   -1.61868600
C       2.87213800   -1.68556000   -0.40980700
C      -2.87211100   -1.68562500    0.40978800
C       1.64963500   -2.20539500    1.50176300
C       2.45274300   -3.31081900    1.77816300
C       3.51114100   -3.59145100    0.91590100
C       3.73444000   -2.76155800   -0.18452000
C      -3.73438500   -2.76164300    0.18449300
C      -3.51105100   -3.59153500   -0.91592200
C      -2.45264500   -3.31088400   -1.77816900
C      -1.64957000   -2.20543800   -1.50176500
Pb     -0.00001000    0.50493800    0.00000000
H      -3.77306300    2.34880800    0.83153100
H      -4.70930700    0.86407800    0.73024700
H      -4.18940100    1.96174500   -1.44961000
H      -3.60580300    0.28482500   -1.41281700
H       3.60578600    0.28489700    1.41281500
H       4.18935000    1.96182900    1.44961000
H       3.77301300    2.34888600   -0.83152900
H       4.70927800    0.86417000   -0.73024900
H      -4.02644100   -0.87012300    2.03252200
H      -2.30391100   -1.13933000    2.37674400
H       4.02643800   -0.87002900   -2.03254700
H       2.30391100   -1.13926800   -2.37675800
H       2.23455200   -3.90763800    2.65623900
H       4.16497000   -4.43858100    1.10208100
H       4.56186900   -2.94593600   -0.86268100
H      -4.56181900   -2.94604000    0.86264300
H      -4.16485700   -4.43868100   -1.10210900
H      -2.23442300   -3.90770700   -2.65623600
N      -1.84691500   -1.42798000   -0.42102400
N       1.84694400   -1.42793900    0.42101500
C       0.51278400   -1.79828500    2.43262000
```

| | | | |
|---|---|---|---|
| O | -0.03555700 | -0.65407200 | 2.13120000 |
| O | 0.20607800 | -2.53167500 | 3.36263200 |
| C | -0.51271000 | -1.79830600 | -2.43260100 |
| O | 0.03555200 | -0.65404600 | -2.13121500 |
| O | -0.20592800 | -2.53172000 | -3.36256900 |
| H | 0.49241100 | 1.26969400 | 3.37936700 |
| H | 0.41878600 | 3.01032600 | 3.80765100 |
| H | 2.70955100 | 2.22820400 | 3.18104900 |
| H | 2.12404500 | 3.57542600 | 2.16616400 |
| H | -0.49244900 | 1.26970900 | -3.37937200 |
| H | -0.41886100 | 3.01034600 | -3.80763900 |
| H | -2.12413000 | 3.57539400 | -2.16614600 |
| H | -2.70960900 | 2.22817100 | -3.18104400 |
| H | 2.20791400 | 3.32223900 | -1.68378100 |
| H | 1.70487300 | 3.22929100 | -3.37355000 |
| H | 3.41843500 | 1.55845000 | -3.12313700 |
| H | 1.86842200 | 0.71029400 | -3.26400900 |
| H | -1.70495200 | 3.22923200 | 3.37356400 |
| H | -2.20799700 | 3.32218200 | 1.68379600 |
| H | -3.41848000 | 1.55835800 | 3.12313800 |
| H | -1.86844900 | 0.71023200 | 3.26400500 |

Pb 0
S 11 1.00
544.675 0.000319
36.5128 0.024214
22.7761 -0.185466
14.2262 0.546090
6.89500 -0.870515
4.30969 -0.203578
1.80085 0.908924
0.890768 0.532000
0.318968 0.032284
0.148352 -0.005268
0.063288 0.001052
S 11 1.00
544.675 -0.000146
36.5128 -0.008348
22.7761 0.069448
14.2262 -0.220729
6.89500 0.400345
4.30969 0.062964
1.80085 -0.531559
0.890768 -0.447946
0.318968 0.362706
0.148352 0.696673
0.063288 0.244099
S 1 1.00
0.318968 1.0
S 1 1.00
0.148352 1.0
S 1 1.00
0.0681 1.0
P 9 1.00
18.6489 -0.017800
11.6679 0.179357
7.29349 -0.416725
2.02849 0.559518
1.04097 0.492518
0.514190 0.132370

0.228651 0.008059
0.095828 0.000799
0.039229 -0.000027
P 9 1.00
18.6489 0.003093
11.6679 -0.045316
7.29349 0.117392
2.02849 -0.200762
1.04097 -0.184882
0.514190 -0.005412
0.228651 0.369878
0.095828 0.546891
0.039229 0.229493
P 1 1.00
0.3696 1.0
P 1 1.00
0.0586 1.0
D 8 1.00
61.3970 0.000325
12.3735 0.013211
6.92686 -0.072781
2.33087 0.269721
1.21019 0.426586
0.600516 0.338139
0.280787 0.135866
0.1093 0.014281
D 1 1.00
0.280787 1.0
D 1 1.00
0.1093 1.0
F 1 1.00
0.2900 1.0
****
H C N O 0
Def2tzvpp
****

Pb 0
ECP60MDF 5 60
H-Komponente
1
2 1.000000 0.000000
S-H
2
2 12.296303 281.285499
2 8.632634 62.520217
P-H
4
2 10.241790 72.276897
2 8.924176 144.591083
2 6.581342 4.758693
2 6.255403 9.940621
D-H
4
2 7.754336 35.848507
2 7.720281 53.724342
2 4.970264 10.115256
2 4.563789 14.833731
F-H
2

2 3.887512 12.209892
2 3.811963 16.190291
G-H
2
2 5.691577 -9.096665
2 5.715567 -11.531996

**Example of a Gaussian Input File for Optimization of a Bi(III) Complex**

```
%chk=Bimacropaam_opt_F5.chk
# rTPSSh/gen pseudo=cards scf=tight
scrf=(pcm,solvent=water) opt freq=noraman integral=ultrafinegrid

Bimacropaam_opt_F5

 2 1
C          -3.75693400   -1.18122600    0.04956300
N          -2.79700200   -0.80985800   -1.01909500
O          -2.26107300   -2.73627300    1.10697900
C          -3.09516800   -1.60798000    1.34670800
C          -2.76927800   -1.84691500   -2.06861600
C          -1.54269500   -1.82990200   -2.95958000
O          -0.42830500   -2.34588900   -2.22893700
C           0.71786200   -2.53651500   -3.05319200
C           1.75078200   -3.35282500   -2.29789900
O           2.24532900   -2.74459800   -1.10828200
O           0.41528700   -2.35071300    2.22753700
C          -0.73171100   -2.53680100    3.05162500
C          -1.76875700   -3.34755500    2.29595800
N           2.79227100   -0.82448500    1.01945300
C           2.75873100   -1.86188300    2.06849300
C           1.53163500   -1.83948800    2.95868500
C           3.08723700   -1.62185200   -1.34695800
C           3.75071200   -1.20026500   -0.04900400
C          -3.11537500    0.50625900   -1.58643300
C           3.11717900    0.48972200    1.58748200
C           2.95680000    1.59375900    0.56997200
C          -2.94891500    1.60889400   -0.56842000
C           1.76045300    2.41531500   -1.24156500
C           2.58517800    3.53053900   -1.34316200
C           3.62975000    3.66641600   -0.43676700
C           3.82966400    2.67932100    0.52129800
C          -3.81656100    2.69856300   -0.51835700
C          -3.61124600    3.68402600    0.44025300
C          -2.56669700    3.54247800    1.34580600
C          -1.74745700    2.42333300    1.24289800
Bi         -0.00013900   -0.22304800   -0.00065100
H          -4.41753000   -1.97927000   -0.30541800
H          -4.39455300   -0.32459000    0.28130600
H          -3.87409400   -1.86746100    2.07688500
H          -2.51150400   -0.77582500    1.75750800
H           2.50954000   -0.78565000   -1.75797100
H           3.86501900   -1.88631500   -2.07655500
H           4.40627100   -2.00262200    0.30553000
H           4.39359700   -0.34720000   -0.27937100
H          -4.13836700    0.54045400   -1.98938600
H          -2.42425600    0.69816900   -2.41031200
H           4.14019800    0.51842600    1.99077500
H           2.42674500    0.68478700    2.41118700
H           2.40320400    4.25330900   -2.12592800
H           4.29451700    4.52013800   -0.48733300
H           4.65556300    2.73880400    1.21898600
H          -4.64267200    2.76247700   -1.21540000
H          -4.27191300    4.54085800    0.49189700
H          -2.38070000    4.26377900    2.12896800
N          -1.93009500    1.50042500    0.29201100
N           1.93803600    1.49086100   -0.29120700
C           0.63790100    2.16059600   -2.23756100
```

| | | | |
|---|---|---|---|
| O | -0.02815200 | 1.08192600 | -2.07148600 |
| C | -0.62563400 | 2.16242100 | 2.23810900 |
| O | 0.03418400 | 1.07998500 | 2.07170500 |
| O | -0.44766200 | 2.99159700 | 3.14308700 |
| H | 1.72149800 | -2.48742700 | 3.82379500 |
| H | 1.30310500 | -0.83423900 | 3.32927100 |
| H | 3.65168200 | -1.78778600 | 2.70890000 |
| H | 2.79145100 | -2.83060400 | 1.57026500 |
| H | -1.73586000 | -2.47708200 | -3.82452600 |
| H | -1.31001100 | -0.82570800 | -3.33045600 |
| H | -2.80628000 | -2.81567500 | -1.57075400 |
| H | -3.66232200 | -1.76823500 | -2.70835400 |
| H | 1.11660000 | -1.56725200 | -3.37610600 |
| H | 0.43896800 | -3.09891900 | -3.95434600 |
| H | 2.58300400 | -3.57345100 | -2.97964800 |
| H | 1.30261500 | -4.29487100 | -1.97858300 |
| H | -1.12583300 | -1.56602600 | 3.37566500 |
| H | -0.45537300 | -3.10142100 | 3.95219500 |
| H | -1.32523300 | -4.29149700 | 1.97576200 |
| H | -2.60168900 | -3.56474100 | 2.97797200 |
| N | 0.45781229 | 3.02636293 | -3.18170963 |
| H | 1.24579691 | 3.44248081 | -3.63550050 |
| H | -0.46938747 | 3.27951447 | -3.45778010 |

Bi 0
S 11 1.00
798.633 0.000389
95.0023 0.002104
21.2520 -0.129681
13.2919 0.665119
8.31210 -0.774022
5.19476 -0.457747
1.90972 0.901072
0.962271 0.544534
0.356026 0.033769
0.168327 -0.005233
0.073265 0.001036
S 11 1.00
798.633 -0.000191
95.0023 -0.000885
21.2520 0.055053
13.2919 -0.302406
8.31210 0.396483
5.19476 0.178323
1.90972 -0.570493
0.962271 -0.479259
0.356026 0.398000
0.168327 0.700968
0.073265 0.231598
S 1 1.00
0.356026 1.0
S 1 1.00
0.168327 1.0
S 1 1.00
0.0784 1.0
P 9 1.00
19.2259 -0.016152
12.0378 0.176031
7.53621 -0.420103
2.16084 0.552674

1.13036 0.499562
0.566778 0.134495
0.271608 0.008046
0.117769 0.001031
0.049304 -0.000162
P 9 1.00
19.2259 0.002733
12.0378 -0.048843
7.53621 0.131840
2.16084 -0.225271
1.13036 -0.207876
0.566778 -0.015585
0.271608 0.393849
0.117769 0.550880
0.049304 0.211150
P 1 1.00
0.4469 1.0
P 1 1.00
0.0743 1.0
D 8 1.00
65.0224 0.000395
13.6908 0.011201
7.09591 -0.072317
2.52090 0.263286
1.34066 0.427325
0.682558 0.337534
0.327714 0.133787
0.1306 0.014699
D 1 1.00
0.327714 1.0
D 1 1.00
0.1306 1.0
F 1 1.00
0.3164 1.0
****
H C N O 0
Def2tzvpp
****

Bi 0
ECP60MDF 5 60
H-Komponente
1
2 1.000000 0.000000
S-H
2
2 13.043090 283.264227
2 8.221682 62.471959
P-H
4
2 10.467777 72.001499
2 9.118901 144.002277
2 6.754791 5.007945
2 6.252592 9.991550
D-H
4
2 8.081474 36.396259
2 7.890595 54.597664
2 4.955556 9.984294
2 4.704559 14.981485

F-H
2
2 4.214546 13.713383
2 4.133400 18.194308
G-H
2
2 6.205709 -10.247443
2 6.227782 -12.955710

**Example of a Gaussian Input File for Optimization of Only Hydrogen Atoms**

```
%chk=Bimacropa_optH.chk
# rTPSSh/gen pseudo=cards scf=tight
scrf=(pcm,solvent=water) opt=modredundant integral=ultrafinegrid

Bimacropa_optH

1 1
 Bi     9.08830000   3.35400000  13.82470000
 O     10.09010000   4.08970000  11.23320000
 O      8.35990000   5.82290000  12.47910000
 O     10.06760000   1.69780000  12.67190000
 O     10.15240000   0.54090000  10.77420000
 N      6.34030000   4.25100000  14.01270000
 N      7.50360000   2.27190000  12.32670000
 C      6.01160000   1.36220000  10.20370000
 C     11.00670000   6.38940000  14.60740000
 C      9.87040000   5.64790000  16.59080000
 C      5.57790000   3.06870000  13.57420000
 C     12.77400000   2.02050000  16.38890000
 C     11.51360000   3.87460000  11.17460000
 C     12.00370000   2.45690000  15.31700000
 C      8.47950000   5.32240000  17.04050000
 C      5.93560000   4.64130000  15.38730000
 C     12.24100000   4.64130000  12.26220000
 C      8.62860000   1.24310000  16.07040000
 C     10.08930000   1.56700000  16.30100000
 C     12.13150000   5.37710000  14.56520000
 C      7.37620000   1.09470000  10.25520000
 C     10.80040000   1.09470000  17.39430000
 C      9.54800000   1.24310000  11.57900000
 C      8.08740000   1.56700000  11.34840000
 C      6.04510000   5.37710000  13.08430000
 C      6.17290000   2.45690000  12.33240000
 C      9.69710000   5.32240000  10.60890000
 C      6.66300000   3.87460000  16.47480000
 C      8.30620000   5.64790000  11.05870000
 C     12.59880000   3.06870000  14.07520000
 C      5.40260000   2.02050000  11.26050000
 C      7.16990000   6.38940000  13.04210000
 O      8.08650000   4.08970000  16.41620000
 O      9.81670000   5.82290000  15.17030000
 O      8.10900000   1.69780000  14.97750000
 O      8.02420000   0.54090000  16.87520000
 N     11.83630000   4.25100000  13.63670000
 N     10.67300000   2.27190000  15.32280000
 C     12.16500000   1.36220000  17.44570000
 H      4.46490000   2.17210000  11.25260000
 H     12.67610000   1.09490000  18.20060000
 H      6.88960000   7.16950000  12.50040000
 H      7.36030000   6.70750000  13.96020000
 H     13.53580000   3.33170000  14.25610000
 H     12.60520000   2.39540000  13.34930000
 H      7.98820000   6.47690000  10.62110000
 H      7.68770000   4.91070000  10.82560000
 H      6.32490000   4.16190000  17.35970000
 H      6.47410000   2.90760000  16.37560000
 H      7.80660000   0.60410000   9.56440000
 H     13.21460000   4.49320000  12.16130000
 H     12.06930000   5.60850000  12.14070000
```

| | | | |
|---|---|---|---|
| H | 5.21230000 | 5.82730000 | 13.37320000 |
| H | 5.89660000 | 5.01740000 | 12.17380000 |
| H | 10.37000000 | 0.60410000 | 18.08510000 |
| H | 4.96200000 | 4.49320000 | 15.48810000 |
| H | 6.10730000 | 5.60850000 | 15.50870000 |
| H | 12.96430000 | 5.82730000 | 14.27630000 |
| H | 12.28000000 | 5.01740000 | 15.47560000 |
| H | 9.72260000 | 5.22740000 | 9.62380000 |
| H | 10.31750000 | 6.04820000 | 10.86950000 |
| H | 8.45400000 | 5.22740000 | 18.02570000 |
| H | 7.85910000 | 6.04820000 | 16.77990000 |
| H | 11.85170000 | 4.16190000 | 10.28970000 |
| H | 11.70250000 | 2.90760000 | 11.27390000 |
| H | 10.18840000 | 6.47690000 | 17.02830000 |
| H | 10.48890000 | 4.91070000 | 16.82380000 |
| H | 5.50050000 | 1.09490000 | 9.44890000 |
| H | 13.71170000 | 2.17210000 | 16.39690000 |
| H | 4.64080000 | 3.33170000 | 13.39330000 |
| H | 5.57140000 | 2.39540000 | 14.30010000 |
| H | 11.28700000 | 7.16950000 | 15.14910000 |
| H | 10.81630000 | 6.70750000 | 13.68930000 |

1 F
2 F
3 F
4 F
5 F
6 F
7 F
8 F
9 F
10 F
11 F
12 F
13 F
14 F
15 F
16 F
17 F
18 F
19 F
20 F
21 F
22 F
23 F
24 F
25 F
26 F
27 F
28 F
29 F
30 F
31 F
32 F
33 F
34 F
35 F
36 F
37 F
38 F

39 F

Bi 0
S 11 1.00
798.633 0.000389
95.0023 0.002104
21.2520 -0.129681
13.2919 0.665119
8.31210 -0.774022
5.19476 -0.457747
1.90972 0.901072
0.962271 0.544534
0.356026 0.033769
0.168327 -0.005233
0.073265 0.001036
S 11 1.00
798.633 -0.000191
95.0023 -0.000885
21.2520 0.055053
13.2919 -0.302406
8.31210 0.396483
5.19476 0.178323
1.90972 -0.570493
0.962271 -0.479259
0.356026 0.398000
0.168327 0.700968
0.073265 0.231598
S 1 1.00
0.356026 1.0
S 1 1.00
0.168327 1.0
S 1 1.00
0.0784 1.0
P 9 1.00
19.2259 -0.016152
12.0378 0.176031
7.53621 -0.420103
2.16084 0.552674
1.13036 0.499562
0.566778 0.134495
0.271608 0.008046
0.117769 0.001031
0.049304 -0.000162
P 9 1.00
19.2259 0.002733
12.0378 -0.048843
7.53621 0.131840
2.16084 -0.225271
1.13036 -0.207876
0.566778 -0.015585
0.271608 0.393849
0.117769 0.550880
0.049304 0.211150
P 1 1.00
0.4469 1.0
P 1 1.00
0.0743 1.0
D 8 1.00
65.0224 0.000395
13.6908 0.011201

7.09591 -0.072317
2.52090 0.263286
1.34066 0.427325
0.682558 0.337534
0.327714 0.133787
0.1306 0.014699
D 1 1.00
0.327714 1.0
D 1 1.00
0.1306 1.0
F 1 1.00
0.3164 1.0
****
H C N O 0
Def2tzvpp
****

Bi 0
ECP60MDF 5 60
H-Komponente
1
2 1.000000 0.000000
S-H
2
2 13.043090 283.264227
2 8.221682 62.471959
P-H
4
2 10.467777 72.001499
2 9.118901 144.002277
2 6.754791 5.007945
2 6.252592 9.991550
D-H
4
2 8.081474 36.396259
2 7.890595 54.597664
2 4.955556 9.984294
2 4.704559 14.981485
F-H
2
2 4.214546 13.713383
2 4.133400 18.194308
G-H
2
2 6.205709 -10.247443
2 6.227782 -12.955710

**Example of a Gaussian Input File for an NBO Calculation**

```
%chk=Bimacropa_pub_NBO.chk
# rTPSSh/gen pseudo=cards scf=tight integral=ultrafinegrid Pop=full Pop=nbo

Bimacropa_pub_NBO

 1 1
Bi      -0.00967500   -0.07761800   -0.07457300
O       -1.43351200    2.48167400    0.51296000
O       -1.95457900    0.29499600    2.03720900
O        1.63179300   -2.11578900    0.78358600
O        1.32469500   -0.16157900    2.66497400
O       -0.35455500    1.36244400   -1.77897700
O        0.31254200   -1.57664400   -1.69586800
O       -1.82327800    2.52335300   -2.99179500
O        1.81211400   -2.87403000   -2.72419700
N        1.53245800    2.25065100    0.81223600
N        2.15808400    0.26119400   -1.14651400
N       -1.25231600   -2.28896900    0.81555400
N       -2.21251700   -0.37277200   -1.01494400
C        0.72588800    3.45113300    1.13887500
C       -2.62403900    0.63217000   -1.80134200
C       -1.53580500    1.60533900   -2.22875600
C       -3.92771700    0.73556200   -2.24077000
C       -0.52952300    3.57343000    0.29561600
C       -4.83969200   -0.23462400   -1.83503300
C       -2.19633700    2.62867700    1.71421700
C       -3.06455500   -1.34217300   -0.65084000
C       -4.40610900   -1.28272400   -1.04327400
C       -2.92165700    1.34611500    1.97003700
C        2.56876000   -0.80630900   -1.85255700
C        1.50913100   -1.86364000   -2.11136500
C       -2.49257200   -2.55198800    0.04763300
C       -2.53510200   -0.92921700    2.48049900
C        3.84766500   -0.90301400   -2.35953700
C       -1.55951500   -2.05737600    2.24903100
C        4.73146400    0.14801000   -2.11389600
C       -0.35442700   -3.46061500    0.65416700
C        2.97513100    1.30144900   -0.96268000
C        4.29272100    1.26066900   -1.42524800
C        0.99805300   -3.30067000    1.30264000
C        2.39369800    2.54377400   -0.34070500
C        2.78283300   -1.71472900    1.54310300
C        2.38587000    1.92987600    1.98473700
C        2.39213700   -1.09456800    2.86676200
C        1.62714900    1.18060600    3.05515900
H        2.20510300    1.17529800    3.98426100
H        0.66525200    1.64863200    3.26632300
H        2.04321500   -1.84740600    3.57802300
H        3.26673400   -0.60693400    3.30540200
H        2.81000300    2.85197300    2.40538800
H        3.22755000    1.32153700    1.64689000
H        3.30197300   -0.99145800    0.91680800
H        3.44379100   -2.57308400    1.70170700
H        3.19933400    3.23900900   -0.07432300
H        1.78286700    3.02685300   -1.10732100
H        0.92062700   -3.23745900    2.39001800
H        1.61231600   -4.17216900    1.05771800
H        4.94767600    2.10373900   -1.24776000
H       -0.22122600   -3.60940500   -0.41595400
```

| | | | |
|---|---|---|---|
| H | -0.82358000 | -4.36030500 | 1.07383800 |
| H | 5.75058800 | 0.09795700 | -2.47669600 |
| H | -0.62856600 | -1.81858400 | 2.76200500 |
| H | -1.97553000 | -2.97030500 | 2.69522000 |
| H | 4.13504800 | -1.77843400 | -2.92419700 |
| H | -3.48458100 | -1.09858700 | 1.95991900 |
| H | -2.75550200 | -0.86674300 | 3.55186100 |
| H | -3.25441100 | -3.02583700 | 0.67445900 |
| H | -2.24816800 | -3.26745200 | -0.74102000 |
| H | -3.46390200 | 1.41874900 | 2.91887200 |
| H | -3.64272400 | 1.12948400 | 1.17375500 |
| H | -5.08702100 | -2.06570200 | -0.73447900 |
| H | -2.91454800 | 3.44837400 | 1.60141000 |
| H | -1.54059100 | 2.85858400 | 2.55923000 |
| H | -5.87533400 | -0.17906000 | -2.14577500 |
| H | -1.03148900 | 4.51879000 | 0.52711300 |
| H | -0.29960700 | 3.55764900 | -0.76815500 |
| H | -4.20792500 | 1.55917100 | -2.88191700 |
| H | 1.33143000 | 4.35991500 | 1.01871200 |
| H | 0.45179900 | 3.39840400 | 2.19191300 |

Bi 0
S 11 1.00
798.633 0.000389
95.0023 0.002104
21.2520 -0.129681
13.2919 0.665119
8.31210 -0.774022
5.19476 -0.457747
1.90972 0.901072
0.962271 0.544534
0.356026 0.033769
0.168327 -0.005233
0.073265 0.001036
S 11 1.00
798.633 -0.000191
95.0023 -0.000885
21.2520 0.055053
13.2919 -0.302406
8.31210 0.396483
5.19476 0.178323
1.90972 -0.570493
0.962271 -0.479259
0.356026 0.398000
0.168327 0.700968
0.073265 0.231598
S 1 1.00
0.356026 1.0
S 1 1.00
0.168327 1.0
S 1 1.00
0.0784 1.0
P 9 1.00
19.2259 -0.016152
12.0378 0.176031
7.53621 -0.420103
2.16084 0.552674
1.13036 0.499562
0.566778 0.134495
0.271608 0.008046

0.117769 0.001031
0.049304 -0.000162
P 9 1.00
19.2259 0.002733
12.0378 -0.048843
7.53621 0.131840
2.16084 -0.225271
1.13036 -0.207876
0.566778 -0.015585
0.271608 0.393849
0.117769 0.550880
0.049304 0.211150
P 1 1.00
0.4469 1.0
P 1 1.00
0.0743 1.0
D 8 1.00
65.0224 0.000395
13.6908 0.011201
7.09591 -0.072317
2.52090 0.263286
1.34066 0.427325
0.682558 0.337534
0.327714 0.133787
0.1306 0.014699
D 1 1.00
0.327714 1.0
D 1 1.00
0.1306 1.0
F 1 1.00
0.3164 1.0
****
H C N O 0
Def2tzvpp
****

Bi 0
ECP60MDF 5 60
H-Komponente
1
2 1.000000 0.000000
S-H
2
2 13.043090 283.264227
2 8.221682 62.471959
P-H
4
2 10.467777 72.001499
2 9.118901 144.002277
2 6.754791 5.007945
2 6.252592 9.991550
D-H
4
2 8.081474 36.396259
2 7.890595 54.597664
2 4.955556 9.984294
2 4.704559 14.981485
F-H
2
2 4.214546 13.713383

2 4.133400 18.194308
G-H
2
2 6.205709 -10.247443
2 6.227782 -12.955710

**Example of a Gaussian Input File for a PES scan**

```
%chk=Bimacropa_PES_step_4_rev_2.chk
# rTPSSh/gen pseudo=cards scf=tight
scrf=(pcm,solvent=water) opt=modredundant integral=ultrafinegrid

Bimacropa_PES_step_4_rev_2

1 1
 C      -2.14094200    2.31458100   -1.80206900
 N      -1.72812900    2.32937600   -0.38390400
 O      -2.08132600   -0.06273600   -2.01463600
 C      -2.91256900    1.07529800   -2.20092200
 C      -0.98753700    3.57099300   -0.05109000
 C       0.21816700    3.83154300   -0.92930600
 O       1.04162700    2.67508900   -0.98288200
 C       2.06219400    2.79707600   -1.96553900
 C       2.74310800    1.46240200   -2.12578500
 O       1.74661600    0.50779200   -2.47314400
 O      -1.04792900   -2.41973000   -1.04647100
 C      -1.73076000   -2.39817400   -2.29677300
 C      -2.75437000   -1.29219600   -2.26371300
 N       1.93044700   -2.09979900   -1.02928500
 C       1.19698000   -3.25832000   -1.57203200
 C      -0.14074500   -3.51624000   -0.89567900
 C       2.25600900   -0.68418600   -3.05519400
 C       2.89883400   -1.60257500   -2.02659900
 C      -2.87404600    2.22718600    0.53207900
 C       2.61134400   -2.44040300    0.22245300
 C       3.08764200   -1.22364100    0.98234600
 C      -3.23925100    0.82868500    0.97133400
 C       2.52396600    0.83086200    1.93284100
 C       3.76133100    0.94395300    2.56906700
 C       4.69296600   -0.07963700    2.38438500
 C       4.34623000   -1.18217200    1.59936600
 C      -4.54367300    0.51497100    1.38246200
 C      -4.80399600   -0.73566300    1.94453700
 C      -3.75257000   -1.64147100    2.10934400
 C      -2.48410400   -1.26533400    1.67083200
 Bi     -0.03497300    0.05902300    0.02787200
 H      -1.23371400    2.36421500   -2.42053300
 H      -2.76279400    3.20068700   -2.03852500
 H      -3.19914800    1.16301000   -3.26449700
 H      -3.84609700    0.96386700   -1.61871500
 H      -0.66621200    3.47612700    0.99362800
 H      -1.65326300    4.45260600   -0.13540300
 H       0.77755100    4.68666100   -0.50724400
 H      -0.08045100    4.11381900   -1.95571000
 H       2.80117800    3.56309700   -1.66732500
 H       1.61217800    3.10447700   -2.92738100
 H       3.26085000    1.17838800   -1.19229300
 H       3.50072100    1.53896900   -2.92626400
 H      -2.24177900   -3.36251300   -2.46483200
 H      -1.02429000   -2.22955000   -3.12832300
 H      -3.27254500   -1.25305600   -3.23814900
 H      -3.50818400   -1.47793700   -1.47680400
 H       1.81136900   -4.17903300   -1.50640000
 H       1.02683700   -3.07795400   -2.64307000
 H      -0.59256500   -4.42725000   -1.32433000
 H      -0.02764800   -3.67047400    0.18524900
 H       2.99743900   -0.43558300   -3.83647700
```

| | | | |
|---|---|---|---|
| H | 1.40383700 | -1.17187500 | -3.55063600 |
| H | 3.39543300 | -2.44610100 | -2.54540300 |
| H | 3.69215600 | -1.05184200 | -1.49892800 |
| H | -3.76627700 | 2.73892300 | 0.12785900 |
| H | -2.60602900 | 2.76215100 | 1.45745700 |
| H | 3.46287200 | -3.12743600 | 0.05527000 |
| H | 1.88994300 | -2.95433100 | 0.87674000 |
| H | 3.95681200 | 1.81154100 | 3.19913800 |
| H | 5.67416700 | -0.03038600 | 2.86057400 |
| H | 5.04044400 | -2.01288000 | 1.46311500 |
| H | -5.33781300 | 1.25461600 | 1.26845300 |
| H | -5.81521600 | -0.99445700 | 2.26437400 |
| H | -3.88544800 | -2.62040900 | 2.56936700 |
| N | -2.25377400 | -0.07189100 | 1.09567900 |
| N | 2.22416700 | -0.20460800 | 1.13289400 |
| C | 1.41142200 | 1.82644900 | 2.18849900 |
| O | 0.24521600 | 1.48884700 | 1.71465900 |
| O | 1.62610700 | 2.83661900 | 2.84807200 |
| C | -1.27570200 | -2.15183400 | 1.87258100 |
| O | -0.14219200 | -1.63457000 | 1.49935900 |
| O | -1.40187600 | -3.25798900 | 2.38759000 |

42 9 8 40 S 13 -10.0

Bi 0
S 11 1.00
798.633 0.000389
95.0023 0.002104
21.2520 -0.129681
13.2919 0.665119
8.31210 -0.774022
5.19476 -0.457747
1.90972 0.901072
0.962271 0.544534
0.356026 0.033769
0.168327 -0.005233
0.073265 0.001036
S 11 1.00
798.633 -0.000191
95.0023 -0.000885
21.2520 0.055053
13.2919 -0.302406
8.31210 0.396483
5.19476 0.178323
1.90972 -0.570493
0.962271 -0.479259
0.356026 0.398000
0.168327 0.700968
0.073265 0.231598
S 1 1.00
0.356026 1.0
S 1 1.00
0.168327 1.0
S 1 1.00
0.0784 1.0
P 9 1.00
19.2259 -0.016152
12.0378 0.176031
7.53621 -0.420103
2.16084 0.552674

1.13036 0.499562
0.566778 0.134495
0.271608 0.008046
0.117769 0.001031
0.049304 -0.000162
P 9 1.00
19.2259 0.002733
12.0378 -0.048843
7.53621 0.131840
2.16084 -0.225271
1.13036 -0.207876
0.566778 -0.015585
0.271608 0.393849
0.117769 0.550880
0.049304 0.211150
P 1 1.00
0.4469 1.0
P 1 1.00
0.0743 1.0
D 8 1.00
65.0224 0.000395
13.6908 0.011201
7.09591 -0.072317
2.52090 0.263286
1.34066 0.427325
0.682558 0.337534
0.327714 0.133787
0.1306 0.014699
D 1 1.00
0.327714 1.0
D 1 1.00
0.1306 1.0
F 1 1.00
0.3164 1.0
****
H C N O 0
Def2svp
****

Bi 0
ECP60MDF 5 60
H-Komponente
1
2 1.000000 0.000000
S-H
2
2 13.043090 283.264227
2 8.221682 62.471959
P-H
4
2 10.467777 72.001499
2 9.118901 144.002277
2 6.754791 5.007945
2 6.252592 9.991550
D-H
4
2 8.081474 36.396259
2 7.890595 54.597664
2 4.955556 9.984294
2 4.704559 14.981485

F-H
2
2 4.214546 13.713383
2 4.133400 18.194308
G-H
2
2 6.205709 -10.247443
2 6.227782 -12.955710

**Example of a Gaussian Input File for a Transition State Optimization**

%chk=Bimacropa_F4_C1_TS.chk
# rTPSSh/gen pseudo=cards scf=tight
scrf=(pcm,solvent=water) opt=qst3 freq=noraman integral=ultrafinegrid

Bimacropa F4

1 1

| | | | |
|---|---|---|---|
| C | 2.32792700 | -2.20890000 | -1.95484600 |
| N | 1.68402300 | -2.32162100 | -0.62918600 |
| O | 2.33060200 | 0.17102800 | -2.05012500 |
| C | 3.17518500 | -0.97226000 | -2.12755500 |
| C | 0.88570800 | -3.57127800 | -0.56466700 |
| C | -0.24570700 | -3.64907000 | -1.56141000 |
| O | -1.13637100 | -2.55282300 | -1.36770200 |
| C | -2.11234000 | -2.51066300 | -2.40719200 |
| C | -3.07297500 | -1.38694100 | -2.13582700 |
| O | -2.33076700 | -0.17125200 | -2.04991800 |
| O | 1.13637400 | 2.55286300 | -1.36852800 |
| C | 2.11237600 | 2.51042100 | -2.40794900 |
| C | 3.07288600 | 1.38665000 | -2.13628700 |
| N | -1.68384400 | 2.32173700 | -0.62935200 |
| C | -0.88556700 | 3.57143800 | -0.56559300 |
| C | 0.24551400 | 3.64884200 | -1.56272800 |
| C | -3.17546600 | 0.97197100 | -2.12700700 |
| C | -2.32828600 | 2.20873000 | -1.95471200 |
| C | 2.68852000 | -2.39133200 | 0.44356200 |
| C | -2.68779000 | 2.39174900 | 0.44386500 |
| C | -3.12032300 | 1.06976100 | 1.02254400 |
| C | 3.12065700 | -1.06928700 | 1.02241500 |
| C | -2.50551300 | -1.02175700 | 1.82226700 |
| C | -3.75613200 | -1.24265800 | 2.37662900 |
| C | -4.72633900 | -0.25846100 | 2.22425900 |
| C | -4.40084600 | 0.91224900 | 1.55520000 |
| C | 4.40109900 | -0.91162600 | 1.55520600 |
| C | 4.72627400 | 0.25897200 | 2.22462700 |
| C | 3.75580000 | 1.24285300 | 2.37727100 |
| C | 2.50529000 | 1.02186800 | 1.82266900 |
| Bi | 0.00011000 | -0.00004600 | 0.02491600 |
| H | 1.54562900 | -2.19007400 | -2.71294600 |
| H | 2.95983800 | -3.08938700 | -2.14249400 |
| H | 3.65688300 | -1.00966000 | -3.11174100 |
| H | 3.97003300 | -0.90706500 | -1.37521000 |
| H | 0.47828600 | -3.63676200 | 0.44266300 |
| H | 1.53490900 | -4.44322400 | -0.73062200 |
| H | -0.77999700 | -4.59325900 | -1.40017600 |
| H | 0.11367300 | -3.65006700 | -2.59633100 |
| H | -2.66403500 | -3.45763700 | -2.44441500 |
| H | -1.61164800 | -2.36040700 | -3.37112000 |
| H | -3.62517800 | -1.55372700 | -1.20384800 |
| H | -3.79300700 | -1.33037500 | -2.96009900 |
| H | 2.66417500 | 3.45733300 | -2.44530900 |
| H | 1.61173500 | 2.36006600 | -3.37189200 |
| H | 3.79294500 | 1.32981200 | -2.96051700 |
| H | 3.62507200 | 1.55360400 | -1.20432600 |
| H | -0.47774400 | 3.63728700 | 0.44155500 |
| H | -1.53483800 | 4.44331700 | -0.73161300 |
| H | -0.11412600 | 3.64912500 | -2.59756500 |
| H | 0.77965000 | 4.59324600 | -1.40221500 |
| H | -3.97000500 | 0.90670700 | -1.37433800 |

| | | | |
|---|---|---|---|
| H | -3.65757100 | 1.00930700 | -3.11099800 |
| H | -1.54629100 | 2.18998800 | -2.71312300 |
| H | -2.96042700 | 3.08909600 | -2.14218200 |
| H | 3.57282300 | -2.95734400 | 0.12583300 |
| H | 2.23982400 | -2.94381900 | 1.27309300 |
| H | -3.57195100 | 2.95832400 | 0.12672500 |
| H | -2.23827800 | 2.94369600 | 1.27331400 |
| H | -3.94081600 | -2.15989100 | 2.91704000 |
| H | -5.71826100 | -0.39487200 | 2.63570100 |
| H | -5.12416000 | 1.70973400 | 1.44544100 |
| H | 5.12461100 | -1.70891300 | 1.44530300 |
| H | 5.71812900 | 0.39548700 | 2.63619500 |
| H | 3.94017100 | 2.15993600 | 2.91804000 |
| N | 2.21193100 | -0.09485500 | 1.13903100 |
| N | -2.21180100 | 0.09513500 | 1.13904300 |
| C | -1.36900100 | -1.98835900 | 2.02793000 |
| O | -0.21796100 | -1.60963900 | 1.55578100 |
| O | -1.54875600 | -3.03051100 | 2.64720700 |
| C | 1.36857900 | 1.98818400 | 2.02862500 |
| O | 0.21785000 | 1.61002300 | 1.55536000 |
| O | 1.54796700 | 3.02968100 | 2.64913300 |

Bimacropa C1

1 1

| | | | |
|---|---|---|---|
| C | -2.23602800 | 2.43613900 | -1.70289500 |
| N | -1.73997300 | 2.36709700 | -0.31079000 |
| O | -2.16018200 | 0.08668100 | -2.10898600 |
| C | -3.02093600 | 1.22248400 | -2.13821000 |
| C | -1.03024500 | 3.62635600 | 0.03829800 |
| C | 0.13933600 | 3.97096200 | -0.85172700 |
| O | 1.09213900 | 2.91662800 | -0.81101300 |
| C | 2.17820100 | 3.17284300 | -1.69679000 |
| C | 3.18301000 | 2.06228300 | -1.56281200 |
| O | 2.53098000 | 0.83435300 | -1.87221100 |
| O | -1.18824200 | -2.35970100 | -1.28138100 |
| C | -1.84628200 | -2.22700700 | -2.54368200 |
| C | -2.85579700 | -1.11824500 | -2.43350400 |
| N | 1.78725400 | -1.99350200 | -1.36866200 |
| C | 1.03730400 | -3.12753300 | -1.96108100 |
| C | -0.25848200 | -3.45088900 | -1.24328400 |
| C | 3.45508800 | -0.22489500 | -2.07575400 |
| C | 2.68505700 | -1.47427200 | -2.42631800 |
| C | -2.85129700 | 2.22829300 | 0.64527700 |
| C | 2.54894700 | -2.47007800 | -0.20511500 |
| C | 2.99231600 | -1.40281400 | 0.76540700 |
| C | -3.23503400 | 0.81806700 | 1.00723700 |
| C | 2.45222400 | 0.47069700 | 2.02863100 |
| C | 3.65187500 | 0.42067900 | 2.72232100 |
| C | 4.55146600 | -0.59172900 | 2.40910500 |
| C | 4.21311400 | -1.51712500 | 1.43248400 |
| C | -4.53382000 | 0.51977100 | 1.42084400 |
| C | -4.82260400 | -0.74588900 | 1.90966900 |
| C | -3.80149800 | -1.68505100 | 2.00224600 |
| C | -2.53693500 | -1.32540400 | 1.56533600 |
| Bi | -0.05674000 | 0.03044000 | 0.00118700 |
| H | -1.37591300 | 2.54088100 | -2.36357400 |
| H | -2.87387600 | 3.32211900 | -1.83308600 |
| H | -3.38344500 | 1.38456300 | -3.15962200 |
| H | -3.89546600 | 1.04513700 | -1.50124600 |

| | | | |
|---|---|---|---|
| H | -0.67636900 | 3.51400700 | 1.06209200 |
| H | -1.73284200 | 4.47113800 | 0.00424400 |
| H | 0.58956100 | 4.89746200 | -0.47373300 |
| H | -0.16646100 | 4.15666000 | -1.88782800 |
| H | 2.65413100 | 4.12826900 | -1.44399100 |
| H | 1.80704200 | 3.23010700 | -2.72737100 |
| H | 3.59373400 | 2.02420900 | -0.54686400 |
| H | 4.00751300 | 2.24038600 | -2.26341200 |
| H | -2.35561100 | -3.16387800 | -2.79413300 |
| H | -1.12458000 | -1.99577100 | -3.33288100 |
| H | -3.37347400 | -1.00408100 | -3.39235600 |
| H | -3.59766400 | -1.33857400 | -1.65775000 |
| H | 1.66156100 | -4.03204400 | -1.98984900 |
| H | 0.81887200 | -2.86736200 | -2.99676300 |
| H | -0.71432500 | -4.33157500 | -1.70774100 |
| H | -0.09664100 | -3.66414700 | -0.18840800 |
| H | 4.08894700 | -0.35615000 | -1.19145000 |
| H | 4.11642200 | 0.01638500 | -2.91757400 |
| H | 2.06383800 | -1.25529500 | -3.29597600 |
| H | 3.40787100 | -2.25074600 | -2.71815800 |
| H | -3.73842100 | 2.77505800 | 0.30513100 |
| H | -2.53608000 | 2.69677100 | 1.58161500 |
| H | 3.42559200 | -3.05196700 | -0.51937400 |
| H | 1.90055800 | -3.14302900 | 0.36158600 |
| H | 3.84933100 | 1.15362500 | 3.49127900 |
| H | 5.49888200 | -0.66619900 | 2.92759300 |
| H | 4.88260500 | -2.32884200 | 1.17942800 |
| H | -5.29930400 | 1.28265000 | 1.36360100 |
| H | -5.82732700 | -0.99177200 | 2.22895600 |
| H | -3.95984500 | -2.67511900 | 2.40480100 |
| N | -2.27496500 | -0.11125400 | 1.05756700 |
| N | 2.15017400 | -0.40517500 | 1.05963400 |
| C | 1.38125100 | 1.47033000 | 2.38949400 |
| O | 0.23555300 | 1.32148700 | 1.79025300 |
| O | 1.60376700 | 2.32856200 | 3.23555900 |
| C | -1.35976100 | -2.25415300 | 1.70028300 |
| O | -0.21362000 | -1.76969600 | 1.32453200 |
| O | -1.50910600 | -3.37237600 | 2.18040800 |

TS Bimacropa F4 C1

1 1

| | | | |
|---|---|---|---|
| C | 2.03369700 | 2.50926700 | 1.85340300 |
| N | 1.58663200 | 2.45091000 | 0.45027400 |
| O | 2.04847100 | 0.15381200 | 2.18823700 |
| C | 2.85537600 | 1.31624900 | 2.28728400 |
| C | 0.73350500 | 3.61338800 | 0.11689100 |
| C | -0.49738000 | 3.74922600 | 0.98904100 |
| O | -1.23187200 | 2.53017700 | 1.00457200 |
| C | -2.19619700 | 2.52382900 | 2.04921700 |
| C | -3.03297100 | 1.27580200 | 1.95415000 |
| O | -2.17491800 | 0.14063100 | 1.97754800 |
| O | 1.14879000 | -2.24400400 | 1.27391400 |
| C | 1.74071100 | -2.16472300 | 2.57019700 |
| C | 2.73385100 | -1.03056300 | 2.56799900 |
| N | -1.54637100 | -2.43968800 | 0.65664400 |
| C | -0.75520000 | -3.68993800 | 0.58163200 |
| C | 0.76828200 | -3.54046000 | 0.81307200 |
| C | -2.87349000 | -1.05453500 | 2.29343800 |
| C | -1.98395800 | -2.25262500 | 2.05632400 |

| | | | |
|---|---|---|---|
| C | 2.71632400 | 2.42098400 | -0.48613100 |
| C | -2.67872900 | -2.50256300 | -0.27762500 |
| C | -3.14870900 | -1.17785700 | -0.83822500 |
| C | 3.19299300 | 1.05170100 | -0.91362600 |
| C | -2.56448400 | 0.88160500 | -1.76359800 |
| C | -3.86493600 | 1.12050700 | -2.20714800 |
| C | -4.84287900 | 0.16259500 | -1.92729700 |
| C | -4.47981400 | -1.00219600 | -1.24881300 |
| C | 4.52591100 | 0.84450200 | -1.30219100 |
| C | 4.89242800 | -0.36840000 | -1.88674200 |
| C | 3.91278900 | -1.34020000 | -2.10615500 |
| C | 2.61252400 | -1.07120600 | -1.68127100 |
| Bi | 0.02445400 | -0.02762300 | -0.02889800 |
| H | 1.14364600 | 2.54919300 | 2.49595800 |
| H | 2.62561000 | 3.42857100 | 2.03924400 |
| H | 3.17423200 | 1.46908400 | 3.33482600 |
| H | 3.77351200 | 1.19821700 | 1.68136100 |
| H | 0.42810200 | 3.49933600 | -0.93099600 |
| H | 1.30556500 | 4.55892700 | 0.20910100 |
| H | -1.12378600 | 4.56582700 | 0.58519200 |
| H | -0.23032800 | 4.02796500 | 2.02447400 |
| H | -2.85843900 | 3.40506100 | 1.97017000 |
| H | -1.68293500 | 2.56537700 | 3.02796600 |
| H | -3.63857500 | 1.27223300 | 1.02891800 |
| H | -3.72403100 | 1.25194800 | 2.81507000 |
| H | 2.26452700 | -3.10371200 | 2.80957900 |
| H | 0.96362800 | -1.99223500 | 3.33597500 |
| H | 3.16449300 | -0.91952800 | 3.58007100 |
| H | 3.55748400 | -1.23827300 | 1.86008300 |
| H | -0.88901500 | -4.13052500 | -0.41366400 |
| H | -1.16929500 | -4.41883100 | 1.30219400 |
| H | 1.11331200 | -4.29793300 | 1.53208600 |
| H | 1.29496000 | -3.70932000 | -0.13547600 |
| H | -3.80099000 | -1.12160700 | 1.69526600 |
| H | -3.17499300 | -1.04514900 | 3.35702400 |
| H | -1.08412600 | -2.16562100 | 2.68149800 |
| H | -2.53859300 | -3.14798200 | 2.40339000 |
| H | 3.57333900 | 3.01224100 | -0.11359900 |
| H | 2.38649600 | 2.90993500 | -1.41714900 |
| H | -3.54119800 | -3.04853200 | 0.14908000 |
| H | -2.34825500 | -3.07915700 | -1.15518300 |
| H | -4.07724700 | 2.03546000 | -2.75994000 |
| H | -5.87591800 | 0.31314700 | -2.24676100 |
| H | -5.21535900 | -1.78168700 | -1.04297400 |
| H | 5.25897200 | 1.63956500 | -1.15587700 |
| H | 5.92700000 | -0.54365500 | -2.18810500 |
| H | 4.12314800 | -2.28859100 | -2.60006700 |
| N | 2.28194900 | 0.07933500 | -1.06918300 |
| N | -2.23501200 | -0.22535400 | -1.07538400 |
| C | -1.42714500 | 1.82527700 | -2.09108800 |
| O | -0.24985200 | 1.42479300 | -1.70822900 |
| O | -1.64449800 | 2.86173300 | -2.70976100 |
| C | 1.47638400 | -2.02917400 | -1.96466100 |
| O | 0.30720700 | -1.65668000 | -1.53427400 |
| O | 1.69188400 | -3.06751400 | -2.58264900 |

Bi 0
S 11 1.00
798.633 0.000389
95.0023 0.002104

21.2520 -0.129681
13.2919 0.665119
8.31210 -0.774022
5.19476 -0.457747
1.90972 0.901072
0.962271 0.544534
0.356026 0.033769
0.168327 -0.005233
0.073265 0.001036
S 11 1.00
798.633 -0.000191
95.0023 -0.000885
21.2520 0.055053
13.2919 -0.302406
8.31210 0.396483
5.19476 0.178323
1.90972 -0.570493
0.962271 -0.479259
0.356026 0.398000
0.168327 0.700968
0.073265 0.231598
S 1 1.00
0.356026 1.0
S 1 1.00
0.168327 1.0
S 1 1.00
0.0784 1.0
P 9 1.00
19.2259 -0.016152
12.0378 0.176031
7.53621 -0.420103
2.16084 0.552674
1.13036 0.499562
0.566778 0.134495
0.271608 0.008046
0.117769 0.001031
0.049304 -0.000162
P 9 1.00
19.2259 0.002733
12.0378 -0.048843
7.53621 0.131840
2.16084 -0.225271
1.13036 -0.207876
0.566778 -0.015585
0.271608 0.393849
0.117769 0.550880
0.049304 0.211150
P 1 1.00
0.4469 1.0
P 1 1.00
0.0743 1.0
D 8 1.00
65.0224 0.000395
13.6908 0.011201
7.09591 -0.072317
2.52090 0.263286
1.34066 0.427325
0.682558 0.337534
0.327714 0.133787
0.1306 0.014699

D 1 1.00
0.327714 1.0
D 1 1.00
0.1306 1.0
F 1 1.00
0.3164 1.0
****
H C N O 0
Def2tzvpp
****

Bi 0
ECP60MDF 5 60
H-Komponente
1
2 1.000000 0.000000
S-H
2
2 13.043090 283.264227
2 8.221682 62.471959
P-H
4
2 10.467777 72.001499
2 9.118901 144.002277
2 6.754791 5.007945
2 6.252592 9.991550
D-H
4
2 8.081474 36.396259
2 7.890595 54.597664
2 4.955556 9.984294
2 4.704559 14.981485
F-H
2
2 4.214546 13.713383
2 4.133400 18.194308
G-H
2
2 6.205709 -10.247443
2 6.227782 -12.955710

**Table S10**: Cartesian coordinates (Å) of **[Pb(macropam)]$^{2+}$** from the obtained crystal structure with optimized hydrogen positions obtained with DFT calculations.

| Center Number | Atomic Number | Coordinates (Angstroms) X | Y | Z |
|---|---|---|---|---|
| 1 | 82 | -0.007565 | -0.572292 | 0.010888 |
| 2 | 8 | -1.106562 | -2.177914 | 2.248866 |
| 3 | 7 | 2.967769 | -0.750134 | -0.388813 |
| 4 | 6 | -3.292261 | -1.851182 | 1.343634 |
| 5 | 8 | 1.683934 | -1.684547 | 2.095449 |
| 6 | 7 | -2.889854 | -0.836766 | 0.344230 |
| 7 | 6 | -2.440437 | -1.827075 | 2.593980 |
| 8 | 8 | 1.243125 | -2.264484 | -2.226483 |
| 9 | 7 | 1.550556 | 1.417699 | 0.650637 |
| 10 | 6 | -0.249176 | -2.113842 | 3.400162 |
| 11 | 8 | -1.564889 | -1.872481 | -2.050146 |
| 12 | 7 | -0.902403 | 2.813525 | 2.814366 |
| 13 | 6 | 1.098924 | -2.580506 | 3.033357 |
| 14 | 8 | -0.594830 | 0.752096 | 2.068700 |
| 15 | 7 | -1.602131 | 1.425137 | -0.637149 |
| 16 | 6 | 2.982723 | -2.113327 | 1.697683 |
| 17 | 8 | 0.580438 | 0.869813 | -2.129838 |
| 18 | 7 | 0.981340 | 3.084359 | -2.413702 |
| 19 | 6 | 3.632484 | -1.022614 | 0.915414 |
| 20 | 6 | 3.397112 | -1.755980 | -1.369819 |
| 21 | 6 | 2.537593 | -1.788347 | -2.590063 |
| 22 | 6 | 0.379107 | -2.281771 | -3.364078 |
| 23 | 6 | -0.949965 | -2.796041 | -2.947176 |
| 24 | 6 | -2.894191 | -2.279675 | -1.695432 |
| 25 | 6 | -3.558288 | -1.149621 | -0.949235 |
| 26 | 6 | 3.316800 | 0.587070 | -0.833851 |
| 27 | 6 | 2.726269 | 1.676702 | 0.039251 |
| 28 | 6 | 3.353589 | 2.928051 | 0.177623 |
| 29 | 6 | 2.771099 | 3.880792 | 0.975969 |
| 30 | 6 | 1.592736 | 3.617233 | 1.615389 |
| 31 | 6 | 1.026649 | 2.380486 | 1.449662 |
| 32 | 6 | -0.218504 | 1.932359 | 2.144372 |
| 33 | 6 | -3.344045 | 0.486120 | 0.786800 |
| 34 | 6 | -2.829264 | 1.581352 | -0.120354 |
| 35 | 6 | -3.615710 | 2.692216 | -0.409270 |
| 36 | 6 | -3.109643 | 3.656552 | -1.266800 |
| 37 | 6 | -1.851058 | 3.495856 | -1.807062 |
| 38 | 6 | -1.120487 | 2.385540 | -1.469050 |
| 39 | 6 | 0.224672 | 2.067542 | -2.032795 |
| 40 | 1 | -3.201330 | -2.838255 | 0.888029 |
| 41 | 1 | -4.347468 | -1.716883 | 1.629339 |
| 42 | 1 | -2.443798 | -0.840737 | 3.069186 |
| 43 | 1 | -2.841562 | -2.554807 | 3.312484 |
| 44 | 1 | -0.650691 | -2.761865 | 4.190148 |
| 45 | 1 | -0.225787 | -1.082604 | 3.766669 |
| 46 | 1 | -1.718265 | 2.532101 | 3.331846 |
| 47 | 1 | -0.652418 | 3.785192 | 2.850377 |
| 48 | 1 | 1.723543 | -2.629403 | 3.935672 |
| 49 | 1 | 1.052899 | -3.590457 | 2.603149 |

| | | | | |
|---|---|---|---|---|
| 50 | 1 | 2.903053 | -3.049593 | 1.129637 |
| 51 | 1 | 3.587548 | -2.322896 | 2.590179 |
| 52 | 1 | 1.925063 | 2.882425 | -2.705633 |
| 53 | 1 | 0.859433 | 3.982507 | -1.974022 |
| 54 | 1 | 3.593291 | -0.104823 | 1.502715 |
| 55 | 1 | 4.693654 | -1.272499 | 0.758225 |
| 56 | 1 | 3.359994 | -2.737796 | -0.894359 |
| 57 | 1 | 4.443839 | -1.582941 | -1.672567 |
| 58 | 1 | 2.440189 | -0.800286 | -3.051933 |
| 59 | 1 | 2.982244 | -2.467154 | -3.330898 |
| 60 | 1 | 0.794338 | -2.941536 | -4.137697 |
| 61 | 1 | 0.294195 | -1.269186 | -3.773779 |
| 62 | 1 | -1.583482 | -2.922388 | -3.835056 |
| 63 | 1 | -0.848276 | -3.775897 | -2.462072 |
| 64 | 1 | -2.849519 | -3.204855 | -1.108404 |
| 65 | 1 | -3.466157 | -2.491824 | -2.607517 |
| 66 | 1 | -3.516078 | -0.253403 | -1.568274 |
| 67 | 1 | -4.617736 | -1.398745 | -0.785449 |
| 68 | 1 | 2.918965 | 0.720238 | -1.841388 |
| 69 | 1 | 4.411002 | 0.725176 | -0.880444 |
| 70 | 1 | 4.312716 | 3.094256 | -0.300579 |
| 71 | 1 | 3.247336 | 4.848097 | 1.104448 |
| 72 | 1 | 1.149802 | 4.369586 | 2.257942 |
| 73 | 1 | -2.950855 | 0.656392 | 1.789889 |
| 74 | 1 | -4.443580 | 0.535425 | 0.828892 |
| 75 | 1 | -4.618614 | 2.759972 | -0.003427 |
| 76 | 1 | -3.710692 | 4.515857 | -1.545673 |
| 77 | 1 | -1.453504 | 4.227629 | -2.501757 |

---------------------------------------------------------------------

**Table S11**: Cartesian coordinates (Å) of **[Pb(macropa)]** from the previously reported crystal structure with optimized hydrogen positions obtained with DFT calculations.

| Center Number | Atomic Number | Coordinates (Angstroms) X | Y | Z |
|---|---|---|---|---|
| 1 | 82 | 0.085178 | -0.495298 | 0.017149 |
| 2 | 6 | -1.471084 | 2.254994 | 1.375492 |
| 3 | 6 | -0.217348 | 2.064082 | 2.212098 |
| 4 | 7 | 1.407144 | 1.606726 | -0.700321 |
| 5 | 7 | 2.992715 | -0.331094 | 0.496271 |
| 6 | 7 | -2.765157 | -1.138555 | -0.464342 |
| 7 | 7 | -1.767617 | 1.256387 | 0.528998 |
| 8 | 8 | -0.575772 | 0.588891 | -2.194111 |
| 9 | 8 | -0.883958 | 2.545056 | -3.219279 |
| 10 | 8 | 1.319621 | -1.957310 | 2.330858 |
| 11 | 8 | -1.496578 | -1.879039 | 2.065161 |
| 12 | 8 | -0.710974 | -2.474544 | -2.150706 |
| 13 | 8 | 1.975548 | -1.698635 | -1.857087 |
| 14 | 8 | 0.413994 | 0.990066 | 2.074716 |
| 15 | 8 | 0.074310 | 2.990162 | 3.010535 |
| 16 | 6 | -0.326008 | 1.802045 | -2.385332 |
| 17 | 6 | 0.787517 | 2.426070 | -1.571027 |
| 18 | 6 | 1.190049 | 3.742328 | -1.762621 |
| 19 | 6 | -2.282621 | 3.357324 | 1.545346 |
| 20 | 6 | 2.290190 | 4.195341 | -1.057161 |
| 21 | 6 | -3.470411 | 3.415473 | 0.820923 |
| 22 | 6 | 2.975256 | 3.331092 | -0.230925 |
| 23 | 6 | -2.935332 | 1.296383 | -0.139334 |
| 24 | 6 | -3.810428 | 2.377437 | -0.009034 |
| 25 | 6 | 2.491489 | 2.037673 | -0.057808 |
| 26 | 6 | 3.179692 | 1.077389 | 0.883333 |
| 27 | 6 | -3.287241 | 0.114921 | -1.008526 |
| 28 | 6 | 3.461730 | -1.228520 | 1.571286 |
| 29 | 6 | 3.755041 | -0.596163 | -0.732704 |
| 30 | 6 | 2.540241 | -1.341393 | 2.742329 |
| 31 | 6 | 3.332867 | -1.851816 | -1.441158 |
| 32 | 6 | 0.420432 | -2.048479 | 3.440686 |
| 33 | 6 | 1.571269 | -2.732786 | -2.749848 |
| 34 | 6 | -0.835390 | -2.721333 | 3.008257 |
| 35 | 6 | 0.193420 | -2.426689 | -3.242778 |
| 36 | 6 | -2.733650 | -2.441808 | 1.657465 |
| 37 | 6 | -2.048427 | -2.231096 | -2.597746 |
| 38 | 6 | -3.448455 | -1.440965 | 0.803927 |
| 39 | 6 | -2.982831 | -2.240220 | -1.423143 |
| 40 | 1 | -4.018497 | -2.223261 | -1.797800 |
| 41 | 1 | -2.852472 | -3.181630 | -0.887814 |
| 42 | 1 | -4.473255 | -1.798529 | 0.614963 |
| 43 | 1 | -3.532870 | -0.515253 | 1.373774 |
| 44 | 1 | -2.348487 | -3.024907 | -3.294094 |
| 45 | 1 | -2.074631 | -1.277715 | -3.135048 |
| 46 | 1 | -3.350298 | -2.659760 | 2.539037 |
| 47 | 1 | -2.557447 | -3.392210 | 1.137756 |
| 48 | 1 | 0.163567 | -1.431955 | -3.701089 |
| 49 | 1 | -0.090069 | -3.171395 | -3.998815 |

| | | | | |
|---|---|---|---|---|
| 50 | 1 | -0.621959 | -3.696788 | 2.553012 |
| 51 | 1 | -1.482650 | -2.881478 | 3.879531 |
| 52 | 1 | 1.595035 | -3.702739 | -2.236684 |
| 53 | 1 | 2.259946 | -2.777342 | -3.602828 |
| 54 | 1 | 0.207502 | -1.041873 | 3.816432 |
| 55 | 1 | 0.887050 | -2.632767 | 4.244316 |
| 56 | 1 | 3.972759 | -1.993852 | -2.320824 |
| 57 | 1 | 3.429256 | -2.746803 | -0.814879 |
| 58 | 1 | 3.021174 | -1.960664 | 3.510809 |
| 59 | 1 | 2.315101 | -0.364879 | 3.184341 |
| 60 | 1 | 4.834168 | -0.650925 | -0.518708 |
| 61 | 1 | 3.601412 | 0.242028 | -1.413568 |
| 62 | 1 | 4.451378 | -0.907299 | 1.933028 |
| 63 | 1 | 3.590639 | -2.222576 | 1.141343 |
| 64 | 1 | -4.381155 | 0.076235 | -1.128419 |
| 65 | 1 | -2.844464 | 0.270608 | -1.993536 |
| 66 | 1 | 4.248156 | 1.334904 | 0.942644 |
| 67 | 1 | 2.745694 | 1.214916 | 1.874726 |
| 68 | 1 | -4.749071 | 2.373471 | -0.549442 |
| 69 | 1 | 3.869771 | 3.646433 | 0.291945 |
| 70 | 1 | -4.134896 | 4.264538 | 0.933073 |
| 71 | 1 | 2.636399 | 5.215053 | -1.181753 |
| 72 | 1 | -1.989498 | 4.128986 | 2.242270 |
| 73 | 1 | 0.649208 | 4.364699 | -2.460853 |

---------------------------------------------------------------------

**Table S12**: Cartesian coordinates (Å) of **[Bi(macropa)]**$^{+}$ from the obtained crystal structure with optimized hydrogen positions obtained with DFT calculations.

| Center Number | Atomic Number | Coordinates (Angstroms) X | Y | Z |
|---|---|---|---|---|
| 1 | 83 | 0.000012 | -0.000006 | -0.046702 |
| 2 | 8 | 1.228629 | -2.491973 | 0.689052 |
| 3 | 8 | 1.483399 | -0.375114 | 2.422211 |
| 4 | 8 | 0.185194 | -1.501267 | -1.702871 |
| 5 | 8 | 1.524214 | -2.848693 | -2.859740 |
| 6 | 7 | 1.722439 | 2.149449 | 0.850261 |
| 7 | 7 | 2.175388 | 0.151735 | -1.128798 |
| 8 | 6 | 4.747904 | -0.187944 | -2.038482 |
| 9 | 6 | -1.874973 | -0.881620 | 2.988709 |
| 10 | 6 | -2.565039 | 1.297552 | 2.247164 |
| 11 | 6 | 2.561365 | 2.413520 | -0.332042 |
| 12 | 6 | -4.382591 | -0.975869 | -1.380196 |
| 13 | 6 | 0.307847 | -3.579150 | 0.473972 |
| 14 | 6 | -3.072229 | -1.134852 | -0.943789 |
| 15 | 6 | -1.954125 | 2.625559 | 1.921639 |
| 16 | 6 | 0.984965 | 3.378051 | 1.240534 |
| 17 | 6 | -0.985023 | -3.377944 | 1.240662 |
| 18 | 6 | -1.341217 | 1.858871 | -2.157643 |
| 19 | 6 | -2.500000 | 0.940158 | -1.833729 |
| 20 | 6 | -2.605559 | -1.737909 | 1.976425 |
| 21 | 6 | 3.785988 | -1.157228 | -2.305966 |
| 22 | 6 | -3.786023 | 1.157193 | -2.306037 |
| 23 | 6 | 1.341254 | -1.858967 | -2.157560 |
| 24 | 6 | 2.499968 | -0.940315 | -1.833675 |
| 25 | 6 | 2.605496 | 1.738045 | 1.976373 |
| 26 | 6 | 3.072258 | 1.134804 | -0.943814 |
| 27 | 6 | 1.954136 | -2.625493 | 1.921757 |
| 28 | 6 | -0.307827 | 3.579158 | 0.473827 |
| 29 | 6 | 2.564975 | -1.297406 | 2.247232 |
| 30 | 6 | -2.561407 | -2.413617 | -0.331962 |
| 31 | 6 | 4.382624 | 0.975804 | -1.380207 |
| 32 | 6 | 1.874903 | 0.881796 | 2.988686 |
| 33 | 8 | -1.228610 | 2.491989 | 0.688944 |
| 34 | 8 | -1.483391 | 0.375200 | 2.422186 |
| 35 | 8 | -0.185159 | 1.501189 | -1.702932 |
| 36 | 8 | -1.524171 | 2.848568 | -2.859864 |
| 37 | 7 | -1.722421 | -2.149426 | 0.850336 |
| 38 | 7 | -2.175431 | -0.151723 | -1.128806 |
| 39 | 6 | -4.747867 | 0.187852 | -2.038521 |
| 40 | 1 | 5.103107 | 1.764652 | -1.206579 |
| 41 | 1 | -5.769885 | 0.326863 | -2.368194 |
| 42 | 1 | 2.512892 | 0.709871 | 3.862004 |
| 43 | 1 | 0.948249 | 1.348437 | 3.321202 |
| 44 | 1 | -3.401538 | -3.074930 | -0.089026 |
| 45 | 1 | -1.945135 | -2.912338 | -1.083170 |
| 46 | 1 | 3.144375 | -1.375052 | 3.173374 |
| 47 | 1 | 3.234404 | -0.973880 | 1.444108 |
| 48 | 1 | -0.773969 | 4.518729 | 0.785068 |
| 49 | 1 | -0.136644 | 3.617569 | -0.599797 |

| | | | | |
|---|---|---|---|---|
| 50 | 1 | 4.006446 | -2.055134 | -2.865343 |
| 51 | 1 | -1.622345 | -4.262762 | 1.112210 |
| 52 | 1 | -0.764323 | -3.302371 | 2.305747 |
| 53 | 1 | 3.028827 | 2.622176 | 2.469276 |
| 54 | 1 | 3.444768 | 1.175148 | 1.566047 |
| 55 | 1 | -4.006460 | 2.055086 | -2.865434 |
| 56 | 1 | 1.622307 | 4.262842 | 1.112027 |
| 57 | 1 | 0.764292 | 3.302507 | 2.305624 |
| 58 | 1 | -3.028917 | -2.622014 | 2.469347 |
| 59 | 1 | -3.444788 | -1.175023 | 1.566004 |
| 60 | 1 | 2.733579 | -3.385439 | 1.804417 |
| 61 | 1 | 1.283069 | -2.931625 | 2.728557 |
| 62 | 1 | -2.733518 | 3.385556 | 1.804275 |
| 63 | 1 | -1.283053 | 2.931679 | 2.728440 |
| 64 | 1 | 0.773954 | -4.518704 | 0.785309 |
| 65 | 1 | 0.136687 | -3.617631 | -0.599652 |
| 66 | 1 | -3.144401 | 1.375223 | 3.173320 |
| 67 | 1 | -3.234453 | 0.973965 | 1.444060 |
| 68 | 1 | 5.769908 | -0.327008 | -2.368166 |
| 69 | 1 | -5.103080 | -1.764714 | -1.206576 |
| 70 | 1 | 3.401463 | 3.074920 | -0.089198 |
| 71 | 1 | 1.945065 | 2.912155 | -1.083287 |
| 72 | 1 | -2.512994 | -0.709633 | 3.861994 |
| 73 | 1 | -0.948367 | -1.348308 | 3.321304 |

---------------------------------------------------------------------

**Table S13**: Cartesian coordinates (Å) of **[Bi(macropapam)]$^{2+}$** from the obtained crystal structure with optimized hydrogen positions obtained with DFT calculations.

| Center Number | Atomic Number | Coordinates (Angstroms) X | Y | Z |
|---|---|---|---|---|
| 1 | 83 | -0.012300 | -0.087818 | 0.000185 |
| 2 | 6 | -0.366687 | -3.489225 | 0.628577 |
| 3 | 6 | 2.481640 | -0.803006 | -1.971072 |
| 4 | 6 | 1.379179 | -1.740688 | -2.263315 |
| 5 | 6 | 3.758290 | -0.881157 | -2.508628 |
| 6 | 7 | -1.277015 | -2.313273 | 0.738652 |
| 7 | 8 | 1.575859 | -2.155135 | 0.726627 |
| 8 | 6 | 0.940348 | -3.289817 | 1.306826 |
| 9 | 6 | 4.674811 | 0.102234 | -2.230618 |
| 10 | 7 | 1.631078 | 2.163722 | 0.908113 |
| 11 | 8 | 1.466856 | -0.259422 | 2.623782 |
| 12 | 6 | 2.799714 | -1.805626 | 1.385467 |
| 13 | 6 | 2.998162 | 1.223166 | -0.967022 |
| 14 | 6 | 4.307393 | 1.159138 | -1.460048 |
| 15 | 7 | -2.207073 | -0.303212 | -0.986669 |
| 16 | 8 | -1.267597 | 2.425543 | 0.639204 |
| 17 | 6 | 2.528062 | -1.208790 | 2.737552 |
| 18 | 6 | -2.644393 | 0.759405 | -1.679631 |
| 19 | 6 | -1.560622 | 1.713412 | -2.067129 |
| 20 | 6 | 2.463885 | 2.476577 | -0.289851 |
| 21 | 7 | 2.140362 | 0.236648 | -1.185781 |
| 22 | 8 | -1.958122 | 0.212733 | 2.023211 |
| 23 | 6 | 1.800858 | 1.060249 | 3.088475 |
| 24 | 6 | -3.971616 | 0.880381 | -2.055929 |
| 25 | 7 | -1.873971 | 2.745585 | -2.722988 |
| 26 | 6 | -4.845252 | -0.130609 | -1.691603 |
| 27 | 8 | -0.362661 | 1.402915 | -1.773044 |
| 28 | 6 | 2.540047 | 1.816568 | 2.037574 |
| 29 | 6 | -3.048572 | -1.301337 | -0.687340 |
| 30 | 6 | -4.397534 | -1.219815 | -1.022100 |
| 31 | 8 | 0.238201 | -1.543472 | -1.719881 |
| 32 | 6 | 0.856736 | 3.382744 | 1.235849 |
| 33 | 6 | -2.462770 | -2.545008 | -0.117033 |
| 34 | 8 | 1.547842 | -2.675947 | -3.117238 |
| 35 | 6 | -0.369468 | 3.527907 | 0.463050 |
| 36 | 6 | -1.702736 | -2.148305 | 2.164380 |
| 37 | 6 | -2.087686 | 2.554768 | 1.796158 |
| 38 | 6 | -2.630485 | -0.992519 | 2.369206 |
| 39 | 6 | -2.873255 | 1.299077 | 1.934181 |
| 40 | 1 | -3.482666 | 1.348835 | 2.842632 |
| 41 | 1 | -3.543653 | 1.151374 | 1.080518 |
| 42 | 1 | -3.546833 | -1.090484 | 1.776493 |
| 43 | 1 | -2.929858 | -0.955376 | 3.422208 |
| 44 | 1 | -2.762027 | 3.409671 | 1.679824 |
| 45 | 1 | -1.472675 | 2.714918 | 2.687099 |
| 46 | 1 | -2.193343 | -3.066229 | 2.508773 |
| 47 | 1 | -0.808939 | -1.991960 | 2.767477 |
| 48 | 1 | -0.878967 | 4.457074 | 0.737465 |
| 49 | 1 | -0.168384 | 3.573640 | -0.607427 |

| | | | | |
|---|---|---|---|---|
| 50 | 1 | -3.226529 | -3.120582 | 0.414756 |
| 51 | 1 | -2.141987 | -3.151629 | -0.968130 |
| 52 | 1 | 0.618799 | 3.345394 | 2.301289 |
| 53 | 1 | 1.481148 | 4.277109 | 1.101787 |
| 54 | 1 | -5.062109 | -2.031294 | -0.756425 |
| 55 | 1 | 2.987136 | 2.727903 | 2.455893 |
| 56 | 1 | 3.358178 | 1.208394 | 1.647963 |
| 57 | 1 | -5.890471 | -0.048923 | -1.962184 |
| 58 | 1 | -1.161253 | 3.384279 | -3.050247 |
| 59 | 1 | -2.825509 | 2.972868 | -2.965251 |
| 60 | 1 | -4.336837 | 1.724378 | -2.623410 |
| 61 | 1 | 2.392559 | 0.987989 | 4.005358 |
| 62 | 1 | 0.852188 | 1.533201 | 3.342062 |
| 63 | 1 | 3.291319 | 3.144911 | -0.029551 |
| 64 | 1 | 1.835277 | 2.987233 | -1.021400 |
| 65 | 1 | 2.227343 | -1.965658 | 3.466458 |
| 66 | 1 | 3.440841 | -0.738762 | 3.113282 |
| 67 | 1 | 4.992594 | 1.968653 | -1.244326 |
| 68 | 1 | 3.289954 | -1.086887 | 0.732836 |
| 69 | 1 | 3.439126 | -2.689851 | 1.470107 |
| 70 | 1 | 5.678610 | 0.035360 | -2.631395 |
| 71 | 1 | -0.853307 | -4.378751 | 1.046228 |
| 72 | 1 | -0.198424 | -3.657845 | -0.434189 |
| 73 | 1 | 0.831466 | -3.157626 | 2.385572 |
| 74 | 1 | 1.563857 | -4.174895 | 1.143405 |
| 75 | 1 | 3.999301 | -1.719873 | -3.147373 |

---------------------------------------------------------------------

**Table S14**: Cartesian coordinates (Å) of **[Bi(macropa)]$^+$** from the previously reported crystal structure with optimized hydrogen positions obtained with DFT calculations.

| Center Number | Atomic Number | X | Y | Z |
|---|---|---|---|---|
| 1 | 83 | -0.009675 | -0.077618 | -0.074573 |
| 2 | 8 | -1.433512 | 2.481674 | 0.512960 |
| 3 | 8 | -1.954579 | 0.294996 | 2.037209 |
| 4 | 8 | 1.631793 | -2.115789 | 0.783586 |
| 5 | 8 | 1.324695 | -0.161579 | 2.664974 |
| 6 | 8 | -0.354555 | 1.362444 | -1.778977 |
| 7 | 8 | 0.312542 | -1.576644 | -1.695868 |
| 8 | 8 | -1.823278 | 2.523353 | -2.991795 |
| 9 | 8 | 1.812114 | -2.874030 | -2.724197 |
| 10 | 7 | 1.532458 | 2.250651 | 0.812236 |
| 11 | 7 | 2.158084 | 0.261194 | -1.146514 |
| 12 | 7 | -1.252316 | -2.288969 | 0.815554 |
| 13 | 7 | -2.212517 | -0.372772 | -1.014944 |
| 14 | 6 | 0.725888 | 3.451133 | 1.138875 |
| 15 | 6 | -2.624039 | 0.632170 | -1.801342 |
| 16 | 6 | -1.535805 | 1.605339 | -2.228756 |
| 17 | 6 | -3.927717 | 0.735562 | -2.240770 |
| 18 | 6 | -0.529523 | 3.573430 | 0.295616 |
| 19 | 6 | -4.839692 | -0.234624 | -1.835033 |
| 20 | 6 | -2.196337 | 2.628677 | 1.714217 |
| 21 | 6 | -3.064555 | -1.342173 | -0.650840 |
| 22 | 6 | -4.406109 | -1.282724 | -1.043274 |
| 23 | 6 | -2.921657 | 1.346115 | 1.970037 |
| 24 | 6 | 2.568760 | -0.806309 | -1.852557 |
| 25 | 6 | 1.509131 | -1.863640 | -2.111365 |
| 26 | 6 | -2.492572 | -2.551988 | 0.047633 |
| 27 | 6 | -2.535102 | -0.929217 | 2.480499 |
| 28 | 6 | 3.847665 | -0.903014 | -2.359537 |
| 29 | 6 | -1.559515 | -2.057376 | 2.249031 |
| 30 | 6 | 4.731464 | 0.148010 | -2.113896 |
| 31 | 6 | -0.354427 | -3.460615 | 0.654167 |
| 32 | 6 | 2.975131 | 1.301449 | -0.962680 |
| 33 | 6 | 4.292721 | 1.260669 | -1.425248 |
| 34 | 6 | 0.998053 | -3.300670 | 1.302640 |
| 35 | 6 | 2.393698 | 2.543774 | -0.340705 |
| 36 | 6 | 2.782833 | -1.714729 | 1.543103 |
| 37 | 6 | 2.385870 | 1.929876 | 1.984737 |
| 38 | 6 | 2.392137 | -1.094568 | 2.866762 |
| 39 | 6 | 1.627149 | 1.180606 | 3.055159 |
| 40 | 1 | 2.205103 | 1.175298 | 3.984261 |
| 41 | 1 | 0.665252 | 1.648632 | 3.266323 |
| 42 | 1 | 2.043215 | -1.847406 | 3.578023 |
| 43 | 1 | 3.266734 | -0.606934 | 3.305402 |
| 44 | 1 | 2.810003 | 2.851973 | 2.405388 |
| 45 | 1 | 3.227550 | 1.321537 | 1.646890 |
| 46 | 1 | 3.301973 | -0.991458 | 0.916808 |
| 47 | 1 | 3.443791 | -2.573084 | 1.701707 |
| 48 | 1 | 3.199334 | 3.239009 | -0.074323 |
| 49 | 1 | 1.782867 | 3.026853 | -1.107321 |

| | | | | |
|---|---|---|---|---|
| 50 | 1 | 0.920627 | -3.237459 | 2.390018 |
| 51 | 1 | 1.612316 | -4.172169 | 1.057718 |
| 52 | 1 | 4.947676 | 2.103739 | -1.247760 |
| 53 | 1 | -0.221226 | -3.609405 | -0.415954 |
| 54 | 1 | -0.823580 | -4.360305 | 1.073838 |
| 55 | 1 | 5.750588 | 0.097957 | -2.476696 |
| 56 | 1 | -0.628566 | -1.818584 | 2.762005 |
| 57 | 1 | -1.975530 | -2.970305 | 2.695220 |
| 58 | 1 | 4.135048 | -1.778434 | -2.924197 |
| 59 | 1 | -3.484581 | -1.098587 | 1.959919 |
| 60 | 1 | -2.755502 | -0.866743 | 3.551861 |
| 61 | 1 | -3.254411 | -3.025837 | 0.674459 |
| 62 | 1 | -2.248168 | -3.267452 | -0.741020 |
| 63 | 1 | -3.463902 | 1.418749 | 2.918872 |
| 64 | 1 | -3.642724 | 1.129484 | 1.173755 |
| 65 | 1 | -5.087021 | -2.065702 | -0.734479 |
| 66 | 1 | -2.914548 | 3.448374 | 1.601410 |
| 67 | 1 | -1.540591 | 2.858584 | 2.559230 |
| 68 | 1 | -5.875334 | -0.179060 | -2.145775 |
| 69 | 1 | -1.031489 | 4.518790 | 0.527113 |
| 70 | 1 | -0.299607 | 3.557649 | -0.768155 |
| 71 | 1 | -4.207925 | 1.559171 | -2.881917 |
| 72 | 1 | 1.331430 | 4.359915 | 1.018712 |
| 73 | 1 | 0.451799 | 3.398404 | 2.191913 |

---------------------------------------------------------------------

**Table S15**: Cartesian coordinates (Å) of the Δ(λλλ)(λλλ) conformer of **[Pb(macropa)]** from geometry optimizations (0 imaginary frequencies).

| Center Number | Atomic Number | Coordinates (Angstroms) X | Y | Z |
|---|---|---|---|---|
| 1 | 6 | -3.131674 | -1.386719 | 1.883723 |
| 2 | 7 | -2.241477 | -1.910179 | 0.834617 |
| 3 | 8 | -2.335788 | 0.873992 | 1.746680 |
| 4 | 6 | -3.515172 | 0.076429 | 1.755263 |
| 5 | 6 | -1.715470 | -3.242518 | 1.211156 |
| 6 | 6 | -0.914339 | -3.328173 | 2.507014 |
| 7 | 8 | 0.259562 | -2.525292 | 2.557226 |
| 8 | 6 | 1.341463 | -3.046992 | 1.791048 |
| 9 | 6 | 2.581880 | -2.236623 | 2.081135 |
| 10 | 8 | 2.335959 | -0.873292 | 1.747052 |
| 11 | 8 | -0.259286 | 2.526149 | 2.556286 |
| 12 | 6 | -1.341235 | 3.047668 | 1.790059 |
| 13 | 6 | -2.581654 | 2.237405 | 2.080454 |
| 14 | 7 | 2.241678 | 1.910463 | 0.833810 |
| 15 | 6 | 1.715693 | 3.242927 | 1.209905 |
| 16 | 6 | 0.914630 | 3.328995 | 2.505781 |
| 17 | 6 | 3.515410 | -0.075829 | 1.755044 |
| 18 | 6 | 3.132088 | 1.387417 | 1.882945 |
| 19 | 6 | -2.936181 | -2.078130 | -0.451164 |
| 20 | 6 | 2.936168 | 2.077915 | -0.452154 |
| 21 | 6 | 3.188040 | 0.817182 | -1.243379 |
| 22 | 6 | -3.188115 | -0.817707 | -1.242863 |
| 23 | 6 | 2.359565 | -1.170327 | -2.097045 |
| 24 | 6 | 3.523678 | -1.400754 | -2.823017 |
| 25 | 6 | 4.554395 | -0.473418 | -2.739231 |
| 26 | 6 | 4.386667 | 0.647997 | -1.937911 |
| 27 | 6 | -4.386745 | -0.648868 | -1.937485 |
| 28 | 6 | -4.554553 | 0.472255 | -2.739193 |
| 29 | 6 | -3.523937 | 1.399686 | -2.823225 |
| 30 | 6 | -2.359809 | 1.169585 | -2.097185 |
| 31 | 82 | -0.000030 | 0.000013 | 0.081425 |
| 32 | 1 | -2.619285 | -1.496777 | 2.837983 |
| 33 | 1 | -4.060625 | -1.978319 | 1.936713 |
| 34 | 1 | -4.130694 | 0.331178 | 2.626870 |
| 35 | 1 | -4.110055 | 0.282004 | 0.858682 |
| 36 | 1 | 4.110082 | -0.281843 | 0.858422 |
| 37 | 1 | 4.131098 | -0.330269 | 2.626627 |
| 38 | 1 | 2.619972 | 1.497979 | 2.837293 |
| 39 | 1 | 4.061107 | 1.978955 | 1.935415 |
| 40 | 1 | -3.890023 | -2.609559 | -0.316429 |
| 41 | 1 | -2.295109 | -2.709753 | -1.071341 |
| 42 | 1 | 3.890010 | 2.609442 | -0.317803 |
| 43 | 1 | 2.294965 | 2.709253 | -1.072485 |
| 44 | 1 | 3.597934 | -2.292666 | -3.428620 |
| 45 | 1 | 5.477594 | -0.623159 | -3.285754 |
| 46 | 1 | 5.171266 | 1.388093 | -1.843197 |
| 47 | 1 | -5.171279 | -1.389003 | -1.842547 |
| 48 | 1 | -5.477738 | 0.621717 | -3.285815 |
| 49 | 1 | -3.598290 | 2.291430 | -3.429065 |

| | | | | |
|---|---|---|---|---|
| 50 | 7 | -2.202172 | 0.082256 | -1.331525 |
| 51 | 7 | 2.202037 | -0.082765 | -1.331703 |
| 52 | 6 | 1.204860 | -2.160119 | -2.148591 |
| 53 | 8 | 0.191578 | -1.897773 | -1.414119 |
| 54 | 8 | 1.322536 | -3.148834 | -2.888793 |
| 55 | 6 | -1.205235 | 2.159507 | -2.148964 |
| 56 | 8 | -0.191633 | 1.897125 | -1.414946 |
| 57 | 8 | -1.323354 | 3.148392 | -2.888869 |
| 58 | 1 | 1.110467 | -3.008392 | 0.721251 |
| 59 | 1 | 1.529321 | -4.092873 | 2.067875 |
| 60 | 1 | 3.409110 | -2.636345 | 1.481450 |
| 61 | 1 | 2.854821 | -2.307854 | 3.141329 |
| 62 | 1 | -1.110328 | 3.008794 | 0.720254 |
| 63 | 1 | -1.529052 | 4.093625 | 2.066629 |
| 64 | 1 | -2.854489 | 2.308906 | 3.140657 |
| 65 | 1 | -3.408933 | 2.636996 | 1.480749 |
| 66 | 1 | 1.510289 | 3.030377 | 3.368465 |
| 67 | 1 | 0.648114 | 4.385186 | 2.644668 |
| 68 | 1 | 2.542131 | 3.967352 | 1.309502 |
| 69 | 1 | 1.095159 | 3.577937 | 0.376960 |
| 70 | 1 | -0.647792 | -4.384318 | 2.646193 |
| 71 | 1 | -1.509961 | -3.029317 | 3.369641 |
| 72 | 1 | -2.541921 | -3.966890 | 1.311046 |
| 73 | 1 | -1.095004 | -3.577835 | 0.378283 |

---------------------------------------------------------------------

E(RTPSSh) = - -2025.73682721 Hartree
Zero-point correction= 0.592167 (Hartree/Particle)
Thermal correction to Energy= 0.629834
Thermal correction to Enthalpy= 0.630778
Thermal correction to Gibbs Free Energy= 0.520743
Sum of electronic and zero-point Energies= -2025.144660
Sum of electronic and thermal Energies= -2025.106994
Sum of electronic and thermal Enthalpies= -2025.106050
Sum of electronic and thermal Free Energies= -2025.216084

**Table S16**: Cartesian coordinates (Å) of the Δ(δδδ)(δδδ) conformer of **[Pb(macropa)]** from geometry optimizations (0 imaginary frequencies).

---------------------------------------------------------------------

| Center Number | Atomic Number | Coordinates (Angstroms) X | Y | Z |
|---|---|---|---|---|
| 1 | 6 | 3.741691 | -1.232306 | -0.017479 |
| 2 | 7 | 2.799106 | -0.866808 | 1.056445 |
| 3 | 8 | 2.159548 | -1.520758 | -1.778363 |
| 4 | 6 | 3.150899 | -2.208848 | -1.015716 |
| 5 | 6 | 2.795254 | -1.892228 | 2.117276 |
| 6 | 6 | 1.457958 | -2.061051 | 2.812194 |
| 7 | 8 | 0.562859 | -2.696081 | 1.895129 |
| 8 | 6 | -0.589375 | -3.277981 | 2.500544 |
| 9 | 6 | -1.654252 | -2.262406 | 2.887910 |
| 10 | 8 | -2.158462 | -1.521493 | 1.778425 |
| 11 | 8 | -0.561141 | -2.697068 | -1.894733 |
| 12 | 6 | 0.591473 | -3.278352 | -2.500020 |
| 13 | 6 | 1.655759 | -2.262252 | -2.887633 |
| 14 | 7 | -2.798436 | -0.868595 | -1.056469 |
| 15 | 6 | -2.793874 | -1.894203 | -2.117113 |
| 16 | 6 | -1.456419 | -2.062415 | -2.811886 |
| 17 | 6 | -3.149396 | -2.210428 | 1.015963 |
| 18 | 6 | -3.740816 | -1.234521 | 0.017480 |
| 19 | 6 | 3.114407 | 0.452955 | 1.619134 |
| 20 | 6 | -3.114555 | 0.450890 | -1.619376 |
| 21 | 6 | -2.976626 | 1.557585 | -0.600819 |
| 22 | 6 | 2.975616 | 1.559502 | 0.600530 |
| 23 | 6 | -1.807274 | 2.410114 | 1.211990 |
| 24 | 6 | -2.664973 | 3.501548 | 1.312967 |
| 25 | 6 | -3.710036 | 3.610435 | 0.404348 |
| 26 | 6 | -3.879807 | 2.618199 | -0.554356 |
| 27 | 6 | 3.878054 | 2.620753 | 0.554030 |
| 28 | 6 | 3.707509 | 3.612905 | -0.404613 |
| 29 | 6 | 2.662455 | 3.503301 | -1.313159 |
| 30 | 6 | 1.805533 | 2.411265 | -1.212171 |
| 31 | 82 | 0.000085 | -0.217989 | 0.000003 |
| 32 | 1 | -0.298291 | -3.844400 | 3.395009 |
| 33 | 1 | -0.981020 | -3.983091 | 1.765559 |
| 34 | 1 | -1.243779 | -1.521497 | 3.575786 |
| 35 | 1 | -2.474841 | -2.783186 | 3.397875 |
| 36 | 1 | 0.300748 | -3.845158 | -3.394362 |
| 37 | 1 | 0.983542 | -3.983055 | -1.764870 |
| 38 | 1 | 2.476635 | -2.782728 | -3.397454 |
| 39 | 1 | 1.244884 | -1.521765 | -3.575719 |
| 40 | 1 | 4.130585 | 0.481683 | 2.040671 |
| 41 | 1 | 2.410085 | 0.645331 | 2.432326 |
| 42 | 1 | -4.130697 | 0.478907 | -2.041037 |
| 43 | 1 | -2.410234 | 0.643622 | -2.432481 |
| 44 | 1 | -2.504669 | 4.227912 | 2.097210 |
| 45 | 1 | -4.397925 | 4.445847 | 0.453380 |
| 46 | 1 | -4.705624 | 2.654421 | -1.253857 |
| 47 | 1 | 4.703913 | 2.657515 | 1.253452 |
| 48 | 1 | 4.394806 | 4.448802 | -0.453671 |
| 49 | 1 | 2.501563 | 4.229574 | -2.097365 |
| 50 | 7 | 1.954407 | 1.483799 | -0.260584 |
| 51 | 7 | -1.955425 | 1.482585 | 0.260372 |
| 52 | 6 | -0.677431 | 2.197796 | 2.212242 |
| 53 | 8 | 0.046376 | 1.161001 | 2.040473 |
| 54 | 8 | -0.554811 | 3.028823 | 3.127520 |

---------------------------------------------------------------------

| | | | | |
|---|---|---|---|---|
| 55 | 6 | 0.675831 | 2.198190 | -2.212429 |
| 56 | 8 | -0.047119 | 1.160799 | -2.040822 |
| 57 | 8 | 0.552590 | 3.029249 | -3.127613 |
| 58 | 1 | 4.665976 | -1.659278 | 0.398452 |
| 59 | 1 | 3.944102 | -2.563288 | -1.685970 |
| 60 | 1 | 2.719641 | -3.080711 | -0.510695 |
| 61 | 1 | -4.664783 | -1.662210 | -0.398416 |
| 62 | 1 | -4.018281 | -0.330183 | 0.559957 |
| 63 | 1 | -3.942372 | -2.565152 | 1.686334 |
| 64 | 1 | -2.717599 | -3.082185 | 0.511222 |
| 65 | 1 | -1.054439 | -1.098522 | -3.143083 |
| 66 | 1 | -1.590022 | -2.702290 | -3.692799 |
| 67 | 1 | -3.052313 | -2.853426 | -1.666240 |
| 68 | 1 | -3.564722 | -1.672388 | -2.869663 |
| 69 | 1 | 1.591935 | -2.700872 | 3.693087 |
| 70 | 1 | 1.055583 | -1.097340 | 3.143443 |
| 71 | 1 | 3.566016 | -1.669798 | 2.869730 |
| 72 | 1 | 3.054228 | -2.851386 | 1.666576 |
| 73 | 1 | 4.018500 | -0.327882 | -0.560138 |

---------------------------------------------------------------------

E(RTPSSh) = -2025.73985069 Hartree

Zero-point correction= 0.592001 (Hartree/Particle)

Thermal correction to Energy= 0.629491

Thermal correction to Enthalpy= 0.630435

Thermal correction to Gibbs Free Energy= 0.521259

Sum of electronic and zero-point Energies= -2025.147850

Sum of electronic and thermal Energies= -2025.110360

Sum of electronic and thermal Enthalpies= -2025.109416

Sum of electronic and thermal Free Energies= -2025.218592

**Table S17**: Cartesian coordinates (Å) of the Δ(δλλ)(δλλ) conformer of **[Pb(macropa)]** from geometry optimizations (0 imaginary frequencies).

| Center Number | Atomic Number | Coordinates (Angstroms) X | Y | Z |
|---|---|---|---|---|
| 1 | 6 | -3.605790 | -1.331701 | -0.630158 |
| 2 | 7 | -2.615319 | -0.584958 | -1.430724 |
| 3 | 8 | -2.152921 | -1.868317 | 1.227376 |
| 4 | 6 | -3.056750 | -2.423229 | 0.272872 |
| 5 | 6 | -2.370183 | -1.166557 | -2.763756 |
| 6 | 6 | -1.792977 | -2.573317 | -2.780931 |
| 7 | 8 | -0.531113 | -2.695745 | -2.128151 |
| 8 | 6 | 0.582203 | -2.407413 | -2.973984 |
| 9 | 6 | 1.847203 | -2.809140 | -2.257773 |
| 10 | 8 | 2.153458 | -1.868212 | -1.227046 |
| 11 | 8 | 0.531820 | -2.695295 | 2.128537 |
| 12 | 6 | -0.581468 | -2.406870 | 2.974371 |
| 13 | 6 | -1.846489 | -2.808906 | 2.258376 |
| 14 | 7 | 2.615583 | -0.584146 | 1.430747 |
| 15 | 6 | 2.370632 | -1.165648 | 2.763838 |
| 16 | 6 | 1.793699 | -2.572526 | 2.781215 |
| 17 | 6 | 3.057677 | -2.422553 | -0.272584 |
| 18 | 6 | 3.606288 | -1.330637 | 0.630233 |
| 19 | 6 | -3.082681 | 0.795372 | -1.642679 |
| 20 | 6 | 3.082473 | 0.796360 | 1.642513 |
| 21 | 6 | 3.098971 | 1.618708 | 0.380301 |
| 22 | 6 | -3.099456 | 1.617877 | -0.380582 |
| 23 | 6 | 1.997558 | 2.253773 | -1.555413 |
| 24 | 6 | 3.014567 | 3.138459 | -1.901397 |
| 25 | 6 | 4.112692 | 3.252041 | -1.059380 |
| 26 | 6 | 4.160606 | 2.475236 | 0.091536 |
| 27 | 6 | -4.161339 | 2.474125 | -0.091945 |
| 28 | 6 | -4.113645 | 3.251159 | 1.058834 |
| 29 | 6 | -3.015480 | 3.138069 | 1.900856 |
| 30 | 6 | -1.998236 | 2.253573 | 1.555042 |
| 31 | 82 | -0.000010 | -0.091387 | 0.000049 |
| 32 | 1 | -4.083866 | 0.816187 | -2.097731 |
| 33 | 1 | -2.384513 | 1.262739 | -2.340100 |
| 34 | 1 | 4.083649 | 0.817589 | 2.097576 |
| 35 | 1 | 2.384158 | 1.263593 | 2.339877 |
| 36 | 1 | 2.928302 | 3.705204 | -2.817590 |
| 37 | 1 | 4.926454 | 3.925184 | -1.300387 |
| 38 | 1 | 5.009603 | 2.522475 | 0.761830 |
| 39 | 1 | -5.010364 | 2.520989 | -0.762231 |
| 40 | 1 | -4.927613 | 3.924094 | 1.299728 |
| 41 | 1 | -2.929333 | 3.705054 | 2.816913 |
| 42 | 7 | -2.040241 | 1.524718 | 0.433138 |
| 43 | 7 | 2.039778 | 1.525123 | -0.433370 |
| 44 | 6 | 0.792658 | 2.060210 | -2.463418 |
| 45 | 8 | -0.063398 | 1.190332 | -2.090544 |
| 46 | 8 | 0.731520 | 2.742897 | -3.498745 |
| 47 | 6 | -0.793292 | 2.060516 | 2.463102 |
| 48 | 8 | 0.062997 | 1.190757 | 2.090496 |
| 49 | 8 | -0.732336 | 2.743507 | 3.498243 |

| | | | | |
|---|---|---|---|---|
| 50 | 1 | -4.372116 | -1.780811 | -1.279850 |
| 51 | 1 | -3.910642 | -2.875876 | 0.793959 |
| 52 | 1 | -2.544932 | -3.216558 | -0.279091 |
| 53 | 1 | 4.372862 | -1.779309 | 1.279940 |
| 54 | 1 | 4.118594 | -0.612758 | -0.012979 |
| 55 | 1 | 3.911744 | -2.874828 | -0.793708 |
| 56 | 1 | 2.546333 | -3.216087 | 0.279526 |
| 57 | 1 | -1.712296 | -3.806900 | 1.824884 |
| 58 | 1 | -2.677925 | -2.850910 | 2.972831 |
| 59 | 1 | -0.610786 | -1.342920 | 3.236073 |
| 60 | 1 | -0.499987 | -2.990419 | 3.899994 |
| 61 | 1 | -4.118462 | -0.613925 | 0.012883 |
| 62 | 1 | 2.678703 | -2.851303 | -2.972146 |
| 63 | 1 | 1.713053 | -3.807015 | -1.823986 |
| 64 | 1 | 0.500839 | -2.991184 | -3.899474 |
| 65 | 1 | 0.611434 | -1.343520 | -3.235931 |
| 66 | 1 | -3.307679 | -1.198170 | -3.345412 |
| 67 | 1 | -1.686832 | -0.488077 | -3.277509 |
| 68 | 1 | -1.701151 | -2.884862 | -3.828464 |
| 69 | 1 | -2.462608 | -3.281526 | -2.292472 |
| 70 | 1 | 2.463444 | -3.280653 | 2.292800 |
| 71 | 1 | 1.702008 | -2.883957 | 3.828797 |
| 72 | 1 | 1.687172 | -0.487248 | 3.277552 |
| 73 | 1 | 3.308160 | -1.197021 | 3.345462 |

---------------------------------------------------------------------

E(RTPSSh) = -2025.73860244 Hartree
Zero-point correction= 0.592301 (Hartree/Particle)
Thermal correction to Energy= 0.629942
Thermal correction to Enthalpy= 0.630886
Thermal correction to Gibbs Free Energy= 0.521198
Sum of electronic and zero-point Energies= -2025.146302
Sum of electronic and thermal Energies= -2025.108661
Sum of electronic and thermal Enthalpies= -2025.107717
Sum of electronic and thermal Free Energies= -2025.217404

**Table S18**: Cartesian coordinates (Å) of the Δ(λδλ)(λδλ) conformer of **[Pb(macropa)]** from geometry optimizations (0 imaginary frequencies).

| Center Number | Atomic Number | Coordinates (Angstroms) X | Y | Z |
|---|---|---|---|---|
| 1 | 6 | 3.280382 | 1.614140 | 1.712824 |
| 2 | 7 | 2.310970 | 1.997209 | 0.671832 |
| 3 | 8 | 2.411119 | -0.570713 | 2.165371 |
| 4 | 6 | 3.592226 | 0.133085 | 1.799519 |
| 5 | 6 | 1.713446 | 3.310393 | 0.990017 |
| 6 | 6 | 0.747734 | 3.309659 | 2.157619 |
| 7 | 8 | -0.435717 | 2.603919 | 1.801802 |
| 8 | 6 | -1.394052 | 2.619800 | 2.853114 |
| 9 | 6 | -2.662114 | 1.953395 | 2.385213 |
| 10 | 8 | -2.411192 | 0.570294 | 2.165359 |
| 11 | 8 | 0.435602 | -2.604213 | 1.801311 |
| 12 | 6 | 1.393925 | -2.620366 | 2.852630 |
| 13 | 6 | 2.662006 | -1.953868 | 2.384905 |
| 14 | 7 | -2.310996 | -1.997352 | 0.671381 |
| 15 | 6 | -1.713489 | -3.310627 | 0.989241 |
| 16 | 6 | -0.747756 | -3.310253 | 2.156827 |
| 17 | 6 | -3.592303 | -0.133475 | 1.799459 |
| 18 | 6 | -3.280415 | -1.614500 | 1.712454 |
| 19 | 6 | 2.944738 | 2.088100 | -0.650290 |
| 20 | 6 | -2.944768 | -2.087955 | -0.650762 |
| 21 | 6 | -3.227559 | -0.766237 | -1.322888 |
| 22 | 6 | 3.227558 | 0.766535 | -1.322700 |
| 23 | 6 | -2.411730 | 1.280675 | -2.038672 |
| 24 | 6 | -3.601935 | 1.580332 | -2.694847 |
| 25 | 6 | -4.635756 | 0.654481 | -2.653006 |
| 26 | 6 | -4.445061 | -0.536051 | -1.962863 |
| 27 | 6 | 4.445056 | 0.536525 | -1.962736 |
| 28 | 6 | 4.635791 | -0.653864 | -2.653121 |
| 29 | 6 | 3.602019 | -1.579759 | -2.695102 |
| 30 | 6 | 2.411820 | -1.280290 | -2.038820 |
| 31 | 82 | 0.000003 | 0.000028 | 0.007272 |
| 32 | 1 | 2.876853 | 1.916480 | 2.679083 |
| 33 | 1 | 4.228385 | 2.158077 | 1.573196 |
| 34 | 1 | 4.361384 | -0.008425 | 2.570970 |
| 35 | 1 | 3.990529 | -0.265232 | 0.860310 |
| 36 | 1 | 1.168115 | 3.641119 | 0.105371 |
| 37 | 1 | 2.497191 | 4.056209 | 1.199138 |
| 38 | 1 | 0.496003 | 4.352612 | 2.392350 |
| 39 | 1 | 1.183805 | 2.868351 | 3.062422 |
| 40 | 1 | -1.621617 | 3.655788 | 3.137104 |
| 41 | 1 | -0.995330 | 2.101745 | 3.734985 |
| 42 | 1 | -3.022081 | 2.424420 | 1.461571 |
| 43 | 1 | -3.433522 | 2.076576 | 3.156835 |
| 44 | 1 | 1.621477 | -3.656424 | 3.136371 |
| 45 | 1 | 0.995194 | -2.102523 | 3.734621 |
| 46 | 1 | 3.433407 | -2.077250 | 3.156502 |
| 47 | 1 | 3.021963 | -2.424693 | 1.461155 |
| 48 | 1 | -1.168185 | -3.641125 | 0.104492 |
| 49 | 1 | -2.497243 | -4.056489 | 1.198175 |

| | | | | |
|---|---|---|---|---|
| 50 | 1 | -1.183872 | -2.869401 | 3.061828 |
| 51 | 1 | -0.495895 | -4.353279 | 2.391099 |
| 52 | 1 | -3.990701 | 0.265031 | 0.860371 |
| 53 | 1 | -4.361402 | 0.007838 | 2.571008 |
| 54 | 1 | -2.876877 | -1.917008 | 2.678653 |
| 55 | 1 | -4.228409 | -2.158432 | 1.572730 |
| 56 | 1 | 3.877095 | 2.670775 | -0.606428 |
| 57 | 1 | 2.246202 | 2.621676 | -1.298782 |
| 58 | 1 | -3.877131 | -2.670624 | -0.607010 |
| 59 | 1 | -2.246244 | -2.621412 | -1.299361 |
| 60 | 1 | -3.688976 | 2.519507 | -3.222242 |
| 61 | 1 | -5.576601 | 0.854313 | -3.151329 |
| 62 | 1 | -5.226881 | -1.283367 | -1.912719 |
| 63 | 1 | 5.226848 | 1.283862 | -1.912457 |
| 64 | 1 | 5.576634 | -0.853544 | -3.151511 |
| 65 | 1 | 3.689078 | -2.518835 | -3.222669 |
| 66 | 7 | 2.239176 | -0.136596 | -1.364525 |
| 67 | 7 | -2.239140 | 0.136850 | -1.364578 |
| 68 | 6 | -1.231495 | 2.240006 | -2.094792 |
| 69 | 8 | -0.156044 | 1.855694 | -1.522697 |
| 70 | 8 | -1.385067 | 3.311889 | -2.701372 |
| 71 | 6 | 1.231636 | -2.239681 | -2.095067 |
| 72 | 8 | 0.156177 | -1.855464 | -1.522944 |
| 73 | 8 | 1.385250 | -3.311475 | -2.701806 |

---------------------------------------------------------------------

E(RTPSSh) = -2025.74583539 Hartree
Zero-point correction= 0.591763 (Hartree/Particle)
Thermal correction to Energy= 0.629501
Thermal correction to Enthalpy= 0.630445
Thermal correction to Gibbs Free Energy= 0.520305
Sum of electronic and zero-point Energies= -2025.154072
Sum of electronic and thermal Energies= -2025.116334
Sum of electronic and thermal Enthalpies= -2025.115390
Sum of electronic and thermal Free Energies= -2025.225530

**Table S19**: Cartesian coordinates (Å) of the Δ(λλδ)(λλδ) conformer of **[Pb(macropa)]** from geometry optimizations (0 imaginary frequencies).

| Center Number | Atomic Number | Coordinates (Angstroms) X | Y | Z |
|---|---|---|---|---|
| 1 | 6 | -3.756934 | -1.181226 | 0.049563 |
| 2 | 7 | -2.797002 | -0.809858 | -1.019095 |
| 3 | 8 | -2.261073 | -2.736273 | 1.106979 |
| 4 | 6 | -3.095168 | -1.607980 | 1.346708 |
| 5 | 6 | -2.769278 | -1.846915 | -2.068616 |
| 6 | 6 | -1.542695 | -1.829902 | -2.959580 |
| 7 | 8 | -0.428305 | -2.345889 | -2.228937 |
| 8 | 6 | 0.717862 | -2.536515 | -3.053192 |
| 9 | 6 | 1.750782 | -3.352825 | -2.297899 |
| 10 | 8 | 2.245329 | -2.744598 | -1.108282 |
| 11 | 8 | 0.415287 | -2.350713 | 2.227537 |
| 12 | 6 | -0.731711 | -2.536801 | 3.051625 |
| 13 | 6 | -1.768757 | -3.347555 | 2.295958 |
| 14 | 7 | 2.792271 | -0.824485 | 1.019453 |
| 15 | 6 | 2.758731 | -1.861883 | 2.068493 |
| 16 | 6 | 1.531635 | -1.839488 | 2.958685 |
| 17 | 6 | 3.087237 | -1.621852 | -1.346958 |
| 18 | 6 | 3.750712 | -1.200265 | -0.049004 |
| 19 | 6 | -3.115375 | 0.506259 | -1.586433 |
| 20 | 6 | 3.117179 | 0.489722 | 1.587482 |
| 21 | 6 | 2.956800 | 1.593759 | 0.569972 |
| 22 | 6 | -2.948915 | 1.608894 | -0.568420 |
| 23 | 6 | 1.760453 | 2.415315 | -1.241565 |
| 24 | 6 | 2.585178 | 3.530539 | -1.343162 |
| 25 | 6 | 3.629750 | 3.666416 | -0.436767 |
| 26 | 6 | 3.829664 | 2.679321 | 0.521298 |
| 27 | 6 | -3.816561 | 2.698563 | -0.518357 |
| 28 | 6 | -3.611246 | 3.684026 | 0.440253 |
| 29 | 6 | -2.566697 | 3.542478 | 1.345806 |
| 30 | 6 | -1.747457 | 2.423333 | 1.242898 |
| 31 | 82 | -0.000139 | -0.223048 | -0.000651 |
| 32 | 1 | -4.417530 | -1.979270 | -0.305418 |
| 33 | 1 | -4.394553 | -0.324590 | 0.281306 |
| 34 | 1 | -3.874094 | -1.867461 | 2.076885 |
| 35 | 1 | -2.511504 | -0.775825 | 1.757508 |
| 36 | 1 | 2.509540 | -0.785650 | -1.757971 |
| 37 | 1 | 3.865019 | -1.886315 | -2.076555 |
| 38 | 1 | 4.406271 | -2.002622 | 0.305530 |
| 39 | 1 | 4.393597 | -0.347200 | -0.279371 |
| 40 | 1 | -4.138367 | 0.540454 | -1.989386 |
| 41 | 1 | -2.424256 | 0.698169 | -2.410312 |
| 42 | 1 | 4.140198 | 0.518426 | 1.990775 |
| 43 | 1 | 2.426745 | 0.684787 | 2.411187 |
| 44 | 1 | 2.403204 | 4.253309 | -2.125928 |
| 45 | 1 | 4.294517 | 4.520138 | -0.487333 |
| 46 | 1 | 4.655563 | 2.738804 | 1.218986 |
| 47 | 1 | -4.642672 | 2.762477 | -1.215400 |
| 48 | 1 | -4.271913 | 4.540858 | 0.491897 |
| 49 | 1 | -2.380700 | 4.263779 | 2.128968 |

| | | | | |
|---|---|---|---|---|
| 50 | 7 | -1.930095 | 1.500425 | 0.292011 |
| 51 | 7 | 1.938036 | 1.490861 | -0.291207 |
| 52 | 6 | 0.637901 | 2.160596 | -2.237561 |
| 53 | 8 | -0.028152 | 1.081926 | -2.071486 |
| 54 | 8 | 0.465242 | 2.990645 | -3.142758 |
| 55 | 6 | -0.625634 | 2.162421 | 2.238109 |
| 56 | 8 | 0.034184 | 1.079985 | 2.071705 |
| 57 | 8 | -0.447662 | 2.991597 | 3.143087 |
| 58 | 1 | 1.721498 | -2.487427 | 3.823795 |
| 59 | 1 | 1.303105 | -0.834239 | 3.329271 |
| 60 | 1 | 3.651682 | -1.787786 | 2.708900 |
| 61 | 1 | 2.791451 | -2.830604 | 1.570265 |
| 62 | 1 | -1.735860 | -2.477082 | -3.824526 |
| 63 | 1 | -1.310011 | -0.825708 | -3.330456 |
| 64 | 1 | -2.806280 | -2.815675 | -1.570754 |
| 65 | 1 | -3.662322 | -1.768235 | -2.708354 |
| 66 | 1 | 1.116600 | -1.567252 | -3.376106 |
| 67 | 1 | 0.438968 | -3.098919 | -3.954346 |
| 68 | 1 | 2.583004 | -3.573451 | -2.979648 |
| 69 | 1 | 1.302615 | -4.294871 | -1.978583 |
| 70 | 1 | -1.125833 | -1.566026 | 3.375665 |
| 71 | 1 | -0.455373 | -3.101421 | 3.952195 |
| 72 | 1 | -1.325233 | -4.291497 | 1.975762 |
| 73 | 1 | -2.601689 | -3.564741 | 2.977972 |

---------------------------------------------------------------------

E(RTPSSh) = -2025.73531718 Hartree
Zero-point correction= 0.591924 (Hartree/Particle)
Thermal correction to Energy= 0.629605
Thermal correction to Enthalpy= 0.630550
Thermal correction to Gibbs Free Energy= 0.520374
Sum of electronic and zero-point Energies= -2025.143393
Sum of electronic and thermal Energies= -2025.105712
Sum of electronic and thermal Enthalpies= -2025.104768
Sum of electronic and thermal Free Energies= -2025.214943

**Table S20**: Cartesian coordinates (Å) of the Δ(δδλ)(δδλ) conformer of **[Pb(macropa)]** from geometry optimizations (0 imaginary frequencies).

| Center Number | Atomic Number | Coordinates (Angstroms) X | Y | Z |
|---|---|---|---|---|
| 1 | 6 | 3.817411 | -0.852255 | 0.273952 |
| 2 | 7 | 2.833852 | -0.355965 | 1.249450 |
| 3 | 8 | 2.262851 | -2.398489 | -0.725284 |
| 4 | 6 | 3.592616 | -2.263019 | -0.236230 |
| 5 | 6 | 2.836930 | -1.125894 | 2.507548 |
| 6 | 6 | 1.863621 | -2.290958 | 2.553859 |
| 7 | 8 | 0.531603 | -1.782482 | 2.563638 |
| 8 | 6 | -0.434523 | -2.810582 | 2.741036 |
| 9 | 6 | -1.815009 | -2.186326 | 2.745121 |
| 10 | 8 | -2.198861 | -1.607188 | 1.498275 |
| 11 | 8 | -0.061956 | -2.606995 | -2.197789 |
| 12 | 6 | 0.680670 | -3.777816 | -1.880840 |
| 13 | 6 | 2.137426 | -3.421928 | -1.706585 |
| 14 | 7 | -2.508010 | -0.983732 | -1.440946 |
| 15 | 6 | -2.189189 | -1.631112 | -2.727713 |
| 16 | 6 | -1.382684 | -2.908707 | -2.632193 |
| 17 | 6 | -2.628991 | -2.560264 | 0.526582 |
| 18 | 6 | -3.365797 | -1.830182 | -0.583370 |
| 19 | 6 | 3.111876 | 1.060181 | 1.533056 |
| 20 | 6 | -3.158939 | 0.301051 | -1.739153 |
| 21 | 6 | -3.296532 | 1.195817 | -0.534323 |
| 22 | 6 | 2.972303 | 1.945648 | 0.317942 |
| 23 | 6 | -2.278944 | 2.179050 | 1.299131 |
| 24 | 6 | -3.430197 | 2.894545 | 1.615580 |
| 25 | 6 | -4.552672 | 2.736260 | 0.814653 |
| 26 | 6 | -4.486902 | 1.871989 | -0.271644 |
| 27 | 6 | 3.879769 | 2.975631 | 0.074705 |
| 28 | 6 | 3.700315 | 3.791033 | -1.035960 |
| 29 | 6 | 2.631160 | 3.543865 | -1.886618 |
| 30 | 6 | 1.768393 | 2.494715 | -1.584297 |
| 31 | 82 | 0.001930 | 0.001054 | -0.013908 |
| 32 | 1 | 2.567554 | -0.446377 | 3.318334 |
| 33 | 1 | 3.845577 | -1.504814 | 2.729455 |
| 34 | 1 | 2.046656 | -2.854625 | 3.478687 |
| 35 | 1 | 1.986407 | -2.970922 | 1.706594 |
| 36 | 1 | -0.274086 | -3.315279 | 3.703753 |
| 37 | 1 | -0.337080 | -3.564008 | 1.949144 |
| 38 | 1 | -1.839944 | -1.371022 | 3.469895 |
| 39 | 1 | -2.550166 | -2.943480 | 3.043619 |
| 40 | 1 | 0.598030 | -4.515152 | -2.690124 |
| 41 | 1 | 0.283842 | -4.232360 | -0.963902 |
| 42 | 1 | 2.676178 | -4.325505 | -1.395155 |
| 43 | 1 | 2.566866 | -3.074345 | -2.654647 |
| 44 | 1 | -1.626183 | -0.909825 | -3.323000 |
| 45 | 1 | -3.116910 | -1.866536 | -3.274585 |
| 46 | 1 | -1.850760 | -3.633463 | -1.954960 |
| 47 | 1 | -1.347560 | -3.371741 | -3.627129 |
| 48 | 1 | 4.119279 | 1.201964 | 1.953306 |
| 49 | 1 | 2.384449 | 1.393406 | 2.276481 |

| | | | | |
|---|---|---|---|---|
| 50 | 1 | -4.150046 | 0.157699 | -2.193670 |
| 51 | 1 | -2.525890 | 0.815893 | -2.465325 |
| 52 | 1 | -3.422522 | 3.547158 | 2.476969 |
| 53 | 1 | -5.468619 | 3.270916 | 1.035032 |
| 54 | 1 | -5.345128 | 1.715818 | -0.912988 |
| 55 | 1 | 4.715226 | 3.125133 | 0.747049 |
| 56 | 1 | 4.393331 | 4.598143 | -1.240299 |
| 57 | 1 | 2.452168 | 4.132784 | -2.774900 |
| 58 | 7 | 1.934821 | 1.728036 | -0.500092 |
| 59 | 7 | -2.219854 | 1.357096 | 0.245431 |
| 60 | 6 | -1.028443 | 2.302077 | 2.156113 |
| 61 | 8 | -0.030359 | 1.582814 | 1.813578 |
| 62 | 8 | -1.061161 | 3.082610 | 3.120483 |
| 63 | 6 | 0.595339 | 2.163370 | -2.495625 |
| 64 | 8 | -0.161902 | 1.204850 | -2.123588 |
| 65 | 8 | 0.461227 | 2.833009 | -3.532193 |
| 66 | 1 | 3.793827 | -0.177463 | -0.582748 |
| 67 | 1 | 4.835798 | -0.813161 | 0.697266 |
| 68 | 1 | 4.314014 | -2.436266 | -1.043591 |
| 69 | 1 | 3.782058 | -3.017076 | 0.538019 |
| 70 | 1 | -3.902806 | -2.569888 | -1.192003 |
| 71 | 1 | -4.121315 | -1.198641 | -0.111817 |
| 72 | 1 | -3.318402 | -3.272744 | 0.997278 |
| 73 | 1 | -1.773371 | -3.120838 | 0.138160 |

---------------------------------------------------------------------

E(RTPSSh) = -2025.74002574
Zero-point correction= 0.591830 (Hartree/Particle)
Thermal correction to Energy= 0.629531
Thermal correction to Enthalpy= 0.630476
Thermal correction to Gibbs Free Energy= 0.520911
Sum of electronic and zero-point Energies= -2025.148196
Sum of electronic and thermal Energies= -2025.110494
Sum of electronic and thermal Enthalpies= -2025.109550
Sum of electronic and thermal Free Energies= -2025.219115

**Table S21**: Cartesian coordinates (Å) of the Δ(λδδ)(λδδ) conformer of **[Pb(macropa)]** from geometry optimizations (0 imaginary frequencies).

| Center Number | Atomic Number | Coordinates (Angstroms) X | Y | Z |
|---|---|---|---|---|
| 1 | 6 | 3.774256 | 1.577512 | -0.478071 |
| 2 | 7 | 2.757902 | 0.984153 | -1.371525 |
| 3 | 8 | 2.299602 | 1.899244 | 1.372242 |
| 4 | 6 | 3.558315 | 1.341553 | 1.007679 |
| 5 | 6 | 2.607195 | 1.800593 | -2.596317 |
| 6 | 6 | 1.236278 | 1.719347 | -3.239446 |
| 7 | 8 | 0.320214 | 2.449666 | -2.417627 |
| 8 | 6 | -0.899353 | 2.818053 | -3.059830 |
| 9 | 6 | -2.045329 | 1.870258 | -2.770322 |
| 10 | 8 | -2.299398 | 1.903385 | -1.366908 |
| 11 | 8 | -0.320042 | 2.440327 | 2.424545 |
| 12 | 6 | 0.899142 | 2.807447 | 3.068222 |
| 13 | 6 | 2.045717 | 1.861385 | 2.775536 |
| 14 | 7 | -2.758208 | 0.979793 | 1.373986 |
| 15 | 6 | -2.607593 | 1.792649 | 2.601172 |
| 16 | 6 | -1.236920 | 1.708886 | 3.244461 |
| 17 | 6 | -3.558133 | 1.344448 | -1.004289 |
| 18 | 6 | -3.774381 | 1.575872 | 0.482132 |
| 19 | 6 | 3.093230 | -0.391096 | -1.763717 |
| 20 | 6 | -3.093756 | -0.396546 | 1.762140 |
| 21 | 6 | -3.049544 | -1.403053 | 0.639748 |
| 22 | 6 | 3.049489 | -1.400745 | -0.644123 |
| 23 | 6 | -1.938634 | -2.247463 | -1.209828 |
| 24 | 6 | -2.883592 | -3.254727 | -1.379617 |
| 25 | 6 | -3.949936 | -3.321081 | -0.493108 |
| 26 | 6 | -4.040386 | -2.377046 | 0.522270 |
| 27 | 6 | 4.040593 | -2.374813 | -0.529512 |
| 28 | 6 | 3.950591 | -3.321644 | 0.483306 |
| 29 | 6 | 2.884415 | -3.257999 | 1.370200 |
| 30 | 6 | 1.939136 | -2.250544 | 1.203327 |
| 31 | 82 | -0.000215 | 0.306092 | 0.000692 |
| 32 | 1 | 3.754383 | 2.656906 | -0.632542 |
| 33 | 1 | 4.785201 | 1.230738 | -0.741028 |
| 34 | 1 | 4.360986 | 1.858525 | 1.550564 |
| 35 | 1 | 3.600090 | 0.284227 | 1.280995 |
| 36 | 1 | -0.751614 | 2.890474 | -4.143011 |
| 37 | 1 | -1.158772 | 3.809052 | -2.679315 |
| 38 | 1 | -1.814032 | 0.847365 | -3.089062 |
| 39 | 1 | -2.931460 | 2.214540 | -3.319585 |
| 40 | 1 | 0.751256 | 2.875972 | 4.151620 |
| 41 | 1 | 1.157992 | 3.799956 | 2.691236 |
| 42 | 1 | 2.931670 | 2.204465 | 3.325860 |
| 43 | 1 | 1.815105 | 0.837311 | 3.090968 |
| 44 | 1 | -3.599728 | 0.287940 | -1.280774 |
| 45 | 1 | -4.360750 | 1.862962 | -1.545772 |
| 46 | 1 | -3.754532 | 2.654794 | 0.639896 |
| 47 | 1 | -4.785379 | 1.228302 | 0.743833 |
| 48 | 1 | 4.088933 | -0.430796 | -2.231546 |
| 49 | 1 | 2.359916 | -0.705047 | -2.510879 |

| | | | | |
|---|---|---|---|---|
| 50 | 1 | -4.089652 | -0.437538 | 2.229441 |
| 51 | 1 | -2.360785 | -0.712644 | 2.508732 |
| 52 | 1 | -2.770016 | -3.949115 | -2.200013 |
| 53 | 1 | -4.708782 | -4.086860 | -0.598210 |
| 54 | 1 | -4.870559 | -2.384123 | 1.217209 |
| 55 | 1 | 4.870633 | -2.379769 | -1.224628 |
| 56 | 1 | 4.709660 | -4.087511 | 0.586157 |
| 57 | 1 | 2.771189 | -3.954634 | 2.188734 |
| 58 | 7 | 2.017418 | -1.358864 | 0.208345 |
| 59 | 7 | -2.017350 | -1.358534 | -0.212418 |
| 60 | 6 | -0.782209 | -2.092899 | -2.185167 |
| 61 | 8 | -0.007616 | -1.096183 | -1.990186 |
| 62 | 8 | -0.681006 | -2.921570 | -3.103157 |
| 63 | 6 | 0.782798 | -2.099105 | 2.179255 |
| 64 | 8 | 0.007448 | -1.102472 | 1.986829 |
| 65 | 8 | 0.682374 | -2.929977 | 3.095351 |
| 66 | 1 | -1.289514 | 2.177301 | 4.234828 |
| 67 | 1 | -0.902317 | 0.673605 | 3.364807 |
| 68 | 1 | -3.366690 | 1.513973 | 3.348106 |
| 69 | 1 | -2.779276 | 2.836967 | 2.339567 |
| 70 | 1 | 1.288953 | 2.189994 | -4.228766 |
| 71 | 1 | 0.900902 | 0.684584 | -3.362086 |
| 72 | 1 | 2.779411 | 2.844094 | -2.331802 |
| 73 | 1 | 3.365934 | 1.523764 | -3.344296 |

---------------------------------------------------------------------

E(RTPSSh) = -2025.73277336 Hartree
Zero-point correction= 0.591972 (Hartree/Particle)
Thermal correction to Energy= 0.629693
Thermal correction to Enthalpy= 0.630637
Thermal correction to Gibbs Free Energy= 0.519969
Sum of electronic and zero-point Energies= -2025.140801
Sum of electronic and thermal Energies= -2025.103080
Sum of electronic and thermal Enthalpies= -2025.102136
Sum of electronic and thermal Free Energies= -2025.212805

**Table S22**: Cartesian coordinates (Å) of the Δ(δλδ)(δλδ) conformer of **[Pb(macropa)]** from geometry optimizations (0 imaginary frequencies).

| Center Number | Atomic Number | Coordinates (Angstroms) X | Y | Z |
|---|---|---|---|---|
| 1 | 6 | 3.796859 | -1.259377 | -0.074146 |
| 2 | 7 | 2.888835 | -0.824359 | 1.002466 |
| 3 | 8 | 2.129409 | -1.924086 | -1.657468 |
| 4 | 6 | 3.247403 | -2.395449 | -0.911824 |
| 5 | 6 | 2.970475 | -1.759703 | 2.137412 |
| 6 | 6 | 1.793176 | -1.702962 | 3.088017 |
| 7 | 8 | 0.641697 | -2.228324 | 2.431581 |
| 8 | 6 | -0.463646 | -2.344881 | 3.320714 |
| 9 | 6 | -1.646261 | -2.902871 | 2.572056 |
| 10 | 8 | -2.128526 | -1.923230 | 1.658256 |
| 11 | 8 | -0.640898 | -2.230771 | -2.430212 |
| 12 | 6 | 0.464434 | -2.348428 | -3.319183 |
| 13 | 6 | 1.647194 | -2.905120 | -2.569802 |
| 14 | 7 | -2.888519 | -0.826012 | -1.002417 |
| 15 | 6 | -2.969762 | -1.762023 | -2.136825 |
| 16 | 6 | -1.792240 | -1.705771 | -3.087183 |
| 17 | 6 | -3.246184 | -2.395900 | 0.912899 |
| 18 | 6 | -3.796363 | -1.260760 | 0.074410 |
| 19 | 6 | 3.229098 | 0.533609 | 1.443577 |
| 20 | 6 | -3.229051 | 0.531613 | -1.444334 |
| 21 | 6 | -3.019944 | 1.566179 | -0.366136 |
| 22 | 6 | 3.019503 | 1.567570 | 0.364888 |
| 23 | 6 | -1.727963 | 2.334787 | 1.397261 |
| 24 | 6 | -2.575434 | 3.417494 | 1.610204 |
| 25 | 6 | -3.684119 | 3.565671 | 0.787149 |
| 26 | 6 | -3.917711 | 2.620644 | -0.204005 |
| 27 | 6 | 3.916866 | 2.622296 | 0.202176 |
| 28 | 6 | 3.682680 | 3.566903 | -0.789231 |
| 29 | 6 | 2.573778 | 3.418102 | -1.611886 |
| 30 | 6 | 1.726794 | 2.335120 | -1.398441 |
| 31 | 82 | 0.000187 | -0.274895 | 0.000115 |
| 32 | 1 | 4.270654 | 0.593933 | 1.795133 |
| 33 | 1 | 2.577007 | 0.785655 | 2.282378 |
| 34 | 1 | -4.270560 | 0.591529 | -1.796117 |
| 35 | 1 | -2.576837 | 0.783353 | -2.283131 |
| 36 | 1 | -2.355836 | 4.107057 | 2.412900 |
| 37 | 1 | -4.367709 | 4.394600 | 0.925071 |
| 38 | 1 | -4.788359 | 2.687410 | -0.844288 |
| 39 | 1 | 4.787636 | 2.689584 | 0.842236 |
| 40 | 1 | 4.365937 | 4.396026 | -0.927628 |
| 41 | 1 | 2.353644 | 4.107409 | -2.414654 |
| 42 | 7 | 1.940936 | 1.447631 | -0.419357 |
| 43 | 7 | -1.941475 | 1.446964 | 0.418360 |
| 44 | 6 | -0.525653 | 2.094946 | 2.299555 |
| 45 | 8 | 0.182596 | 1.066840 | 2.037707 |
| 46 | 8 | -0.330850 | 2.896136 | 3.228284 |
| 47 | 6 | 0.524244 | 2.094636 | -2.300273 |
| 48 | 8 | -0.182847 | 1.065714 | -2.038660 |
| 49 | 8 | 0.328222 | 2.896214 | -3.228445 |

| | | | | |
|---|---|---|---|---|
| 50 | 1 | 4.773076 | -1.562354 | 0.335486 |
| 51 | 1 | 4.032641 | -2.736339 | -1.599228 |
| 52 | 1 | 2.948217 | -3.254508 | -0.298183 |
| 53 | 1 | -4.772377 | -1.564640 | -0.335035 |
| 54 | 1 | -3.974245 | -0.412611 | 0.736802 |
| 55 | 1 | -4.031192 | -2.736853 | 1.600525 |
| 56 | 1 | -2.946365 | -3.255154 | 0.299841 |
| 57 | 1 | -1.585297 | -0.686045 | -3.429424 |
| 58 | 1 | -2.025421 | -2.321877 | -3.965583 |
| 59 | 1 | -3.027798 | -2.775348 | -1.737376 |
| 60 | 1 | -3.893302 | -1.588187 | -2.712014 |
| 61 | 1 | 2.026556 | -2.318594 | 3.966695 |
| 62 | 1 | 1.586300 | -0.683058 | 3.429757 |
| 63 | 1 | 3.894137 | -1.585439 | 2.712267 |
| 64 | 1 | 3.028493 | -2.773254 | 1.738548 |
| 65 | 1 | 1.358419 | -3.815407 | -2.028815 |
| 66 | 1 | 2.436879 | -3.166659 | -3.286185 |
| 67 | 1 | 0.712335 | -1.368326 | -3.744421 |
| 68 | 1 | 0.208975 | -3.028800 | -4.142247 |
| 69 | 1 | 3.974175 | -0.411563 | -0.737118 |
| 70 | 1 | -2.435985 | -3.163487 | 3.288727 |
| 71 | 1 | -1.357259 | -3.813896 | 2.032434 |
| 72 | 1 | -0.208078 | -3.024019 | 4.144754 |
| 73 | 1 | -0.711701 | -1.364182 | 3.744475 |

---------------------------------------------------------------------

E(RTPSSh) = -2025.74986545
Zero-point correction= 0.591596 (Hartree/Particle)
Thermal correction to Energy= 0.629341
Thermal correction to Enthalpy= 0.630285
Thermal correction to Gibbs Free Energy= 0.519956
Sum of electronic and zero-point Energies= -2025.158269
Sum of electronic and thermal Energies= -2025.120525
Sum of electronic and thermal Enthalpies= -2025.119580
Sum of electronic and thermal Free Energies= -2025.229909

**Table S23**: Cartesian coordinates (Å) of the Δ(λλλ)(λλλ) conformer of **[Pb(macropapam)]$^+$** from geometry optimizations (0 imaginary frequencies).

| Center Number | Atomic Number | Coordinates (Angstroms) X | Y | Z |
|---|---|---|---|---|
| 1 | 6 | 3.375398 | 1.115598 | -1.682906 |
| 2 | 7 | 2.378094 | 0.056046 | -1.917359 |
| 3 | 8 | 2.399891 | 1.818817 | 0.401225 |
| 4 | 6 | 3.631879 | 1.463458 | -0.227129 |
| 5 | 6 | 1.951314 | 0.061192 | -3.335182 |
| 6 | 6 | 1.183230 | 1.295112 | -3.796363 |
| 7 | 8 | -0.065443 | 1.502943 | -3.143086 |
| 8 | 6 | -1.104395 | 0.642626 | -3.606317 |
| 9 | 6 | -2.419159 | 1.115544 | -3.034028 |
| 10 | 8 | -2.321353 | 1.135257 | -1.611887 |
| 11 | 8 | 0.176170 | 3.178191 | 1.162817 |
| 12 | 6 | 1.213375 | 2.965301 | 2.122592 |
| 13 | 6 | 2.524044 | 2.847496 | 1.382669 |
| 14 | 7 | -2.354920 | 1.445195 | 1.269743 |
| 15 | 6 | -1.903500 | 2.429578 | 2.280718 |
| 16 | 6 | -1.067581 | 3.579941 | 1.735453 |
| 17 | 6 | -3.568813 | 1.332078 | -0.952219 |
| 18 | 6 | -3.331401 | 2.067723 | 0.355288 |
| 19 | 6 | 2.917866 | -1.279761 | -1.620054 |
| 20 | 6 | -2.931712 | 0.300492 | 1.991095 |
| 21 | 6 | -3.103935 | -0.958649 | 1.178458 |
| 22 | 6 | 3.016788 | -1.630777 | -0.155105 |
| 23 | 6 | -2.210186 | -2.487531 | -0.302632 |
| 24 | 6 | -3.323242 | -3.315716 | -0.204729 |
| 25 | 6 | -4.363197 | -2.930292 | 0.633470 |
| 26 | 6 | -4.253983 | -1.738991 | 1.330742 |
| 27 | 6 | 4.081750 | -2.403170 | 0.310775 |
| 28 | 6 | 4.114150 | -2.780063 | 1.646240 |
| 29 | 6 | 3.092891 | -2.358816 | 2.488479 |
| 30 | 6 | 2.071338 | -1.578505 | 1.958298 |
| 31 | 82 | 0.035968 | 0.304092 | -0.037863 |
| 32 | 1 | 3.017541 | 2.021639 | -2.169000 |
| 33 | 1 | 4.338140 | 0.849717 | -2.148236 |
| 34 | 1 | 4.316555 | 2.317740 | -0.209342 |
| 35 | 1 | 4.102600 | 0.646855 | 0.328202 |
| 36 | 1 | -4.068879 | 0.368177 | -0.812997 |
| 37 | 1 | -4.224028 | 1.959622 | -1.566715 |
| 38 | 1 | -2.960352 | 3.058638 | 0.099888 |
| 39 | 1 | -4.300662 | 2.200159 | 0.860943 |
| 40 | 1 | 3.905074 | -1.418751 | -2.084286 |
| 41 | 1 | 2.239304 | -2.005269 | -2.074323 |
| 42 | 1 | -3.898630 | 0.565506 | 2.441443 |
| 43 | 1 | -2.239409 | 0.057259 | 2.800256 |
| 44 | 1 | -3.405203 | -4.230209 | -0.775530 |
| 45 | 1 | -5.247349 | -3.547464 | 0.727723 |
| 46 | 1 | -5.049060 | -1.401083 | 1.982835 |
| 47 | 1 | 4.870917 | -2.695327 | -0.370501 |
| 48 | 1 | 4.930538 | -3.381977 | 2.026248 |
| 49 | 1 | 3.071900 | -2.608056 | 3.539800 |

| | | | | |
|---|---|---|---|---|
| 50 | 7 | 2.031807 | -1.240847 | 0.662145 |
| 51 | 7 | -2.104951 | -1.336976 | 0.376317 |
| 52 | 6 | -1.038116 | -2.784257 | -1.195549 |
| 53 | 8 | -0.179963 | -1.917587 | -1.417090 |
| 54 | 6 | 0.960761 | -1.049422 | 2.851751 |
| 55 | 8 | 0.119574 | -0.253545 | 2.307095 |
| 56 | 8 | 0.950707 | -1.404515 | 4.039179 |
| 57 | 1 | -0.913854 | -0.390711 | -3.300038 |
| 58 | 1 | -1.161821 | 0.677980 | -4.701755 |
| 59 | 1 | -3.209511 | 0.427715 | -3.357788 |
| 60 | 1 | -2.662000 | 2.122220 | -3.394959 |
| 61 | 1 | 1.018274 | 2.055614 | 2.698546 |
| 62 | 1 | 1.268503 | 3.816856 | 2.811090 |
| 63 | 1 | 2.777617 | 3.790896 | 0.885328 |
| 64 | 1 | 3.315694 | 2.603423 | 2.100317 |
| 65 | 1 | -1.589082 | 4.125423 | 0.948780 |
| 66 | 1 | -0.886022 | 4.282706 | 2.557055 |
| 67 | 1 | -2.767336 | 2.866201 | 2.807300 |
| 68 | 1 | -1.315920 | 1.879807 | 3.017044 |
| 69 | 1 | 1.023379 | 1.195175 | -4.877495 |
| 70 | 1 | 1.752336 | 2.210688 | -3.635061 |
| 71 | 1 | 2.823711 | -0.044105 | -4.001008 |
| 72 | 1 | 1.329040 | -0.822699 | -3.482743 |
| 73 | 7 | -0.963043 | -3.999549 | -1.749352 |
| 74 | 1 | -1.608112 | -4.740799 | -1.535302 |
| 75 | 1 | -0.182221 | -4.213836 | -2.350334 |

---------------------------------------------------------------------

E(RTPSSh) = -2006.31485783 Hartree
Zero-point correction= 0.617550 (Hartree/Particle)
Thermal correction to Energy= 0.655828
Thermal correction to Enthalpy= 0.656772
Thermal correction to Gibbs Free Energy= 0.545909
Sum of electronic and zero-point Energies= -2005.697308
Sum of electronic and thermal Energies= -2005.659030
Sum of electronic and thermal Enthalpies= -2005.658086
Sum of electronic and thermal Free Energies= -2005.768949

**Table S24**: Cartesian coordinates (Å) of the Δ(δδδ)(δδδ) conformer of **[Pb(macropapam)]$^+$** from geometry optimizations (0 imaginary frequencies).

| Center Number | Atomic Number | Coordinates (Angstroms) X | Y | Z |
|---|---|---|---|---|
| 1 | 6 | 3.895692 | -0.367477 | -0.139902 |
| 2 | 7 | 2.935352 | -0.221460 | 0.971935 |
| 3 | 8 | 2.323318 | -1.012189 | -1.808084 |
| 4 | 6 | 3.494538 | -1.454453 | -1.115997 |
| 5 | 6 | 3.221210 | -1.207192 | 2.033360 |
| 6 | 6 | 1.996367 | -1.662562 | 2.801183 |
| 7 | 8 | 1.201437 | -2.464575 | 1.923766 |
| 8 | 6 | 0.216080 | -3.259042 | 2.583338 |
| 9 | 6 | -1.007870 | -2.465820 | 3.014858 |
| 10 | 8 | -1.690976 | -1.853199 | 1.921887 |
| 11 | 8 | -0.057364 | -2.720874 | -1.842276 |
| 12 | 6 | 1.163409 | -3.055272 | -2.505804 |
| 13 | 6 | 1.955614 | -1.828097 | -2.922719 |
| 14 | 7 | -2.559975 | -1.415335 | -0.901823 |
| 15 | 6 | -2.422653 | -2.474950 | -1.921881 |
| 16 | 6 | -1.139621 | -2.392720 | -2.722667 |
| 17 | 6 | -2.577067 | -2.727904 | 1.223596 |
| 18 | 6 | -3.372123 | -1.901194 | 0.233042 |
| 19 | 6 | 2.953928 | 1.144666 | 1.513290 |
| 20 | 6 | -3.158254 | -0.215430 | -1.498190 |
| 21 | 6 | -3.170326 | 0.966473 | -0.557855 |
| 22 | 6 | 2.492825 | 2.164823 | 0.498123 |
| 23 | 6 | -2.183205 | 2.088856 | 1.206283 |
| 24 | 6 | -3.154892 | 3.083652 | 1.188226 |
| 25 | 6 | -4.171051 | 2.995896 | 0.242247 |
| 26 | 6 | -4.193605 | 1.915543 | -0.624740 |
| 27 | 6 | 3.076346 | 3.427984 | 0.417339 |
| 28 | 6 | 2.623115 | 4.322002 | -0.545782 |
| 29 | 6 | 1.626788 | 3.921629 | -1.427730 |
| 30 | 6 | 1.097596 | 2.642154 | -1.294337 |
| 31 | 82 | 0.102764 | -0.349275 | -0.056141 |
| 32 | 1 | 0.655342 | -3.746393 | 3.463170 |
| 33 | 1 | -0.060796 | -4.034695 | 1.867720 |
| 34 | 1 | -0.720845 | -1.649348 | 3.679290 |
| 35 | 1 | -1.690064 | -3.127614 | 3.562898 |
| 36 | 1 | 0.954027 | -3.673180 | -3.386966 |
| 37 | 1 | 1.730890 | -3.656773 | -1.793984 |
| 38 | 1 | 2.852266 | -2.138397 | -3.471800 |
| 39 | 1 | 1.357795 | -1.186918 | -3.571631 |
| 40 | 1 | 3.955845 | 1.424899 | 1.870248 |
| 41 | 1 | 2.279691 | 1.175044 | 2.372433 |
| 42 | 1 | -4.187616 | -0.405474 | -1.837098 |
| 43 | 1 | -2.565197 | 0.056826 | -2.374557 |
| 44 | 1 | -3.137390 | 3.915207 | 1.879185 |
| 45 | 1 | -4.943222 | 3.753246 | 0.199351 |
| 46 | 1 | -4.991998 | 1.798270 | -1.346364 |
| 47 | 1 | 3.879650 | 3.695773 | 1.092157 |
| 48 | 1 | 3.060039 | 5.310256 | -0.621255 |
| 49 | 1 | 1.262677 | 4.564842 | -2.216338 |

| | | | | |
|---|---|---|---|---|
| 50 | 7 | 1.509261 | 1.806488 | -0.334678 |
| 51 | 7 | -2.179076 | 1.077346 | 0.328768 |
| 52 | 6 | -1.066332 | 2.021401 | 2.213817 |
| 53 | 8 | -0.168723 | 1.174904 | 2.106302 |
| 54 | 6 | 0.055911 | 2.109519 | -2.269036 |
| 55 | 8 | -0.328989 | 0.902698 | -2.086916 |
| 56 | 8 | -0.327122 | 2.861810 | -3.176884 |
| 57 | 1 | 4.906211 | -0.575594 | 0.238495 |
| 58 | 1 | 4.303478 | -1.610323 | -1.838935 |
| 59 | 1 | 3.306629 | -2.404090 | -0.602881 |
| 60 | 1 | -4.225195 | -2.493202 | -0.127304 |
| 61 | 1 | -3.775220 | -1.039928 | 0.766036 |
| 62 | 1 | -3.266930 | -3.193649 | 1.937989 |
| 63 | 1 | -2.020513 | -3.527270 | 0.721231 |
| 64 | 1 | -0.995456 | -1.397438 | -3.155566 |
| 65 | 1 | -1.178926 | -3.125136 | -3.536822 |
| 66 | 1 | -2.433857 | -3.439734 | -1.413587 |
| 67 | 1 | -3.279770 | -2.460227 | -2.610276 |
| 68 | 1 | 2.317558 | -2.267456 | 3.657589 |
| 69 | 1 | 1.420606 | -0.811357 | 3.180810 |
| 70 | 1 | 3.962373 | -0.807607 | 2.740289 |
| 71 | 1 | 3.663504 | -2.088668 | 1.567428 |
| 72 | 1 | 3.941042 | 0.574032 | -0.687784 |
| 73 | 7 | -1.088786 | 2.893607 | 3.229438 |
| 74 | 1 | -0.362701 | 2.846363 | 3.927457 |
| 75 | 1 | -1.845182 | 3.538251 | 3.382726 |

---------------------------------------------------------------------

E(RTPSSh) = -2006.31924624 Hartree

Zero-point correction= 0.617191 (Hartree/Particle)

Thermal correction to Energy= 0.655345

Thermal correction to Enthalpy= 0.656289

Thermal correction to Gibbs Free Energy= 0.546208

Sum of electronic and zero-point Energies= -2005.702055

Sum of electronic and thermal Energies= -2005.663901

Sum of electronic and thermal Enthalpies= -2005.662957

Sum of electronic and thermal Free Energies= -2005.773038

**Table S25**: Cartesian coordinates (Å) of the Δ(δλλ)(δλλ) conformer of **[Pb(macropapam)]$^+$** from geometry optimizations (0 imaginary frequencies).

| Center Number | Atomic Number | Coordinates (Angstroms) X | Y | Z |
|---|---|---|---|---|
| 1 | 6 | 3.800868 | -0.677987 | 0.270817 |
| 2 | 7 | 2.757374 | -0.196974 | 1.200593 |
| 3 | 8 | 2.291597 | -1.522514 | -1.421343 |
| 4 | 6 | 3.426568 | -1.850370 | -0.618004 |
| 5 | 6 | 2.770143 | -0.875287 | 2.511070 |
| 6 | 6 | 2.367573 | -2.343044 | 2.505331 |
| 7 | 8 | 1.048949 | -2.581404 | 2.012919 |
| 8 | 6 | 0.038119 | -2.484626 | 3.017803 |
| 9 | 6 | -1.261024 | -3.002178 | 2.454543 |
| 10 | 8 | -1.776772 | -2.081544 | 1.492452 |
| 11 | 8 | -0.270815 | -2.688886 | -1.857018 |
| 12 | 6 | 0.636663 | -2.402679 | -2.926797 |
| 13 | 6 | 2.037742 | -2.543856 | -2.391383 |
| 14 | 7 | -2.618667 | -0.934150 | -1.101696 |
| 15 | 6 | -2.474440 | -1.697938 | -2.357845 |
| 16 | 6 | -1.612607 | -2.948586 | -2.275652 |
| 17 | 6 | -2.892965 | -2.626985 | 0.795308 |
| 18 | 6 | -3.509766 | -1.572890 | -0.108077 |
| 19 | 6 | 2.950128 | 1.243420 | 1.443122 |
| 20 | 6 | -3.167518 | 0.387752 | -1.451833 |
| 21 | 6 | -3.117368 | 1.379157 | -0.317760 |
| 22 | 6 | 2.658948 | 2.090083 | 0.229697 |
| 23 | 6 | -1.998595 | 2.211257 | 1.523874 |
| 24 | 6 | -2.966467 | 3.186567 | 1.736485 |
| 25 | 6 | -4.051025 | 3.245360 | 0.868115 |
| 26 | 6 | -4.137314 | 2.321566 | -0.159458 |
| 27 | 6 | 3.416926 | 3.225902 | -0.053238 |
| 28 | 6 | 3.096375 | 3.995016 | -1.164530 |
| 29 | 6 | 2.048500 | 3.592441 | -1.982973 |
| 30 | 6 | 1.348113 | 2.438622 | -1.648070 |
| 31 | 82 | 0.048308 | -0.316032 | -0.069147 |
| 32 | 1 | 3.969663 | 1.461320 | 1.792683 |
| 33 | 1 | 2.261470 | 1.533708 | 2.238285 |
| 34 | 1 | -4.205068 | 0.310741 | -1.806571 |
| 35 | 1 | -2.560276 | 0.782501 | -2.268410 |
| 36 | 1 | -2.905436 | 3.884702 | 2.559462 |
| 37 | 1 | -4.824192 | 3.989472 | 1.009443 |
| 38 | 1 | -4.983659 | 2.316197 | -0.834268 |
| 39 | 1 | 4.247590 | 3.491199 | 0.588591 |
| 40 | 1 | 3.669406 | 4.883777 | -1.398980 |
| 41 | 1 | 1.771579 | 4.136116 | -2.875102 |
| 42 | 7 | 1.637196 | 1.724475 | -0.552709 |
| 43 | 7 | -2.064561 | 1.345219 | 0.502495 |
| 44 | 6 | -0.819440 | 1.994343 | 2.432758 |
| 45 | 8 | -0.036539 | 1.056479 | 2.229984 |
| 46 | 6 | 0.244949 | 1.906036 | -2.548080 |
| 47 | 8 | -0.267419 | 0.783079 | -2.211866 |
| 48 | 8 | -0.064479 | 2.571398 | -3.547300 |
| 49 | 1 | 4.714947 | -0.948033 | 0.820139 |

| | | | | |
|---|---|---|---|---|
| 50 | 1 | 4.285788 | -2.057956 | -1.268141 |
| 51 | 1 | 3.209596 | -2.762415 | -0.055941 |
| 52 | 1 | -4.381342 | -2.022481 | -0.606702 |
| 53 | 1 | -3.886121 | -0.780414 | 0.540224 |
| 54 | 1 | -3.664983 | -2.931784 | 1.514271 |
| 55 | 1 | -2.580687 | -3.525628 | 0.255883 |
| 56 | 1 | 2.152030 | -3.528490 | -1.923985 |
| 57 | 1 | 2.760427 | -2.459309 | -3.211400 |
| 58 | 1 | 0.465476 | -1.394830 | -3.317635 |
| 59 | 1 | 0.494925 | -3.128419 | -3.735350 |
| 60 | 1 | 4.059969 | 0.152654 | -0.387632 |
| 61 | 1 | -1.985882 | -3.126661 | 3.268637 |
| 62 | 1 | -1.097426 | -3.978911 | 1.983404 |
| 63 | 1 | 0.317080 | -3.103929 | 3.878883 |
| 64 | 1 | -0.079346 | -1.449292 | 3.356006 |
| 65 | 1 | 3.772427 | -0.813218 | 2.966677 |
| 66 | 1 | 2.084520 | -0.323650 | 3.156308 |
| 67 | 1 | 2.452431 | -2.716948 | 3.532378 |
| 68 | 1 | 3.038783 | -2.939740 | 1.889146 |
| 69 | 1 | -2.014423 | -3.675603 | -1.572727 |
| 70 | 1 | -1.605313 | -3.419338 | -3.264738 |
| 71 | 1 | -2.026407 | -1.018893 | -3.084360 |
| 72 | 1 | -3.462367 | -1.994963 | -2.744670 |
| 73 | 7 | -0.661095 | 2.827515 | 3.468466 |
| 74 | 1 | -1.283848 | 3.592611 | 3.662738 |
| 75 | 1 | 0.111004 | 2.678483 | 4.099482 |

---------------------------------------------------------------------

E(RTPSSh) = -2006.31716325 Hartree
Zero-point correction= 0.617632 (Hartree/Particle)
Thermal correction to Energy= 0.655931
Thermal correction to Enthalpy= 0.656875
Thermal correction to Gibbs Free Energy= 0.545743
Sum of electronic and zero-point Energies= -2005.699531
Sum of electronic and thermal Energies= -2005.661232
Sum of electronic and thermal Enthalpies= -2005.660288
Sum of electronic and thermal Free Energies= -2005.771420

**Table S26**: Cartesian coordinates (Å) of the Δ(λδλ)(λδλ) conformer of **[Pb(macropapam)]**$^{+}$from geometry optimizations (0 imaginary frequencies).

| Center Number | Atomic Number | Coordinates (Angstroms) X | Y | Z |
|---|---|---|---|---|
| 1 | 6 | 3.155053 | 1.765092 | 1.663822 |
| 2 | 7 | 2.202463 | 2.065313 | 0.577192 |
| 3 | 8 | 2.383114 | -0.445115 | 2.145030 |
| 4 | 6 | 3.541859 | 0.307801 | 1.807610 |
| 5 | 6 | 1.583463 | 3.389928 | 0.807625 |
| 6 | 6 | 0.596423 | 3.437561 | 1.953618 |
| 7 | 8 | -0.547367 | 2.652072 | 1.628537 |
| 8 | 6 | -1.536989 | 2.715446 | 2.651914 |
| 9 | 6 | -2.772031 | 1.988967 | 2.189717 |
| 10 | 8 | -2.458146 | 0.610146 | 2.018922 |
| 11 | 8 | 0.488308 | -2.543746 | 1.857300 |
| 12 | 6 | 1.447881 | -2.504580 | 2.907171 |
| 13 | 6 | 2.691187 | -1.807976 | 2.419457 |
| 14 | 7 | -2.253968 | -2.030890 | 0.706400 |
| 15 | 6 | -1.643201 | -3.325551 | 1.074402 |
| 16 | 6 | -0.676594 | -3.266904 | 2.238808 |
| 17 | 6 | -3.615215 | -0.167863 | 1.727292 |
| 18 | 6 | -3.235318 | -1.633722 | 1.730760 |
| 19 | 6 | 2.872386 | 2.104026 | -0.730020 |
| 20 | 6 | -2.883278 | -2.174883 | -0.612645 |
| 21 | 6 | -3.159527 | -0.884985 | -1.347080 |
| 22 | 6 | 3.195109 | 0.761433 | -1.337524 |
| 23 | 6 | -2.363780 | 1.146607 | -2.123403 |
| 24 | 6 | -3.528523 | 1.383564 | -2.845906 |
| 25 | 6 | -4.541873 | 0.435342 | -2.803135 |
| 26 | 6 | -4.352902 | -0.715327 | -2.049250 |
| 27 | 6 | 4.409785 | 0.564654 | -2.000856 |
| 28 | 6 | 4.645591 | -0.639148 | -2.643245 |
| 29 | 6 | 3.664045 | -1.623172 | -2.609325 |
| 30 | 6 | 2.483465 | -1.356044 | -1.925083 |
| 31 | 82 | -0.031281 | 0.096982 | 0.117395 |
| 32 | 1 | 2.699815 | 2.073298 | 2.604473 |
| 33 | 1 | 4.074607 | 2.357018 | 1.536600 |
| 34 | 1 | 4.281794 | 0.230678 | 2.615156 |
| 35 | 1 | 4.003614 | -0.096096 | 0.900152 |
| 36 | 1 | 1.052976 | 3.660899 | -0.105133 |
| 37 | 1 | 2.356066 | 4.152420 | 0.990663 |
| 38 | 1 | 0.291890 | 4.481803 | 2.098648 |
| 39 | 1 | 1.027698 | 3.087516 | 2.898871 |
| 40 | 1 | -1.793774 | 3.762215 | 2.856760 |
| 41 | 1 | -1.150891 | 2.265970 | 3.575177 |
| 42 | 1 | -3.138189 | 2.412767 | 1.246441 |
| 43 | 1 | -3.556712 | 2.102807 | 2.948115 |
| 44 | 1 | 1.709444 | -3.525277 | 3.215568 |
| 45 | 1 | 1.033496 | -1.978085 | 3.776515 |
| 46 | 1 | 3.461153 | -1.867514 | 3.199310 |
| 47 | 1 | 3.076579 | -2.297244 | 1.516150 |
| 48 | 1 | -1.097185 | -3.688762 | 0.203405 |
| 49 | 1 | -2.419978 | -4.068724 | 1.314713 |

| | | | | |
|---|---|---|---|---|
| 50 | 1 | -1.121288 | -2.808364 | 3.130564 |
| 51 | 1 | -0.399784 | -4.295768 | 2.504521 |
| 52 | 1 | -4.051587 | 0.150296 | 0.774757 |
| 53 | 1 | -4.370546 | -0.008323 | 2.507614 |
| 54 | 1 | -2.811288 | -1.857302 | 2.709415 |
| 55 | 1 | -4.156794 | -2.229264 | 1.630833 |
| 56 | 1 | 3.793117 | 2.702855 | -0.686079 |
| 57 | 1 | 2.186017 | 2.595850 | -1.423048 |
| 58 | 1 | -3.817898 | -2.751318 | -0.547667 |
| 59 | 1 | -2.190359 | -2.743714 | -1.236647 |
| 60 | 1 | -3.612515 | 2.293328 | -3.422887 |
| 61 | 1 | -5.464415 | 0.587311 | -3.349978 |
| 62 | 1 | -5.117882 | -1.479630 | -1.996696 |
| 63 | 1 | 5.150739 | 1.353556 | -2.007218 |
| 64 | 1 | 5.576605 | -0.813308 | -3.167081 |
| 65 | 1 | 3.826364 | -2.559958 | -3.123914 |
| 66 | 7 | 2.256863 | -0.188989 | -1.306449 |
| 67 | 7 | -2.192547 | 0.041402 | -1.385430 |
| 68 | 6 | -1.207759 | 2.132092 | -2.179211 |
| 69 | 8 | -0.154236 | 1.815301 | -1.524504 |
| 70 | 8 | -1.347770 | 3.156550 | -2.861521 |
| 71 | 6 | 1.329429 | -2.316081 | -1.854532 |
| 72 | 8 | 0.230631 | -1.942602 | -1.417329 |
| 73 | 7 | 1.519501 | -3.565384 | -2.289710 |
| 74 | 1 | 2.409896 | -3.907252 | -2.608401 |
| 75 | 1 | 0.749666 | -4.215866 | -2.257483 |

---------------------------------------------------------------------

E(RTPSSh) = -2006.32372540 Hartree
Zero-point correction= 0.617241 (Hartree/Particle)
Thermal correction to Energy= 0.655547
Thermal correction to Enthalpy= 0.656491
Thermal correction to Gibbs Free Energy= 0.545966
Sum of electronic and zero-point Energies= -2005.706485
Sum of electronic and thermal Energies= -2005.668179
Sum of electronic and thermal Enthalpies= -2005.667234
Sum of electronic and thermal Free Energies= -2005.777759

**Table S27**: Cartesian coordinates (Å) of the Δ(λλδ)(λλδ) conformer of **[Pb(macropapam)]$^+$** from geometry optimizations (0 imaginary frequencies).

| Center Number | Atomic Number | Coordinates (Angstroms) X | Y | Z |
|---|---|---|---|---|
| 1 | 6 | 3.830941 | 0.787643 | -0.246422 |
| 2 | 7 | 2.867341 | 0.661858 | 0.876787 |
| 3 | 8 | 3.116760 | -1.293201 | -1.191785 |
| 4 | 6 | 3.388249 | 0.070423 | -1.509344 |
| 5 | 6 | 3.422992 | -0.209713 | 1.931030 |
| 6 | 6 | 2.407743 | -0.769100 | 2.906957 |
| 7 | 8 | 1.619457 | -1.758668 | 2.243804 |
| 8 | 6 | 0.758393 | -2.456253 | 3.139702 |
| 9 | 6 | 0.158520 | -3.650353 | 2.422050 |
| 10 | 8 | -0.633906 | -3.321922 | 1.282994 |
| 11 | 8 | 0.580787 | -2.291319 | -2.137325 |
| 12 | 6 | 1.636435 | -1.972091 | -3.047187 |
| 13 | 6 | 2.961897 | -2.137003 | -2.330285 |
| 14 | 7 | -2.112454 | -1.984692 | -0.796348 |
| 15 | 6 | -1.683917 | -2.989307 | -1.792993 |
| 16 | 6 | -0.671285 | -2.491913 | -2.802295 |
| 17 | 6 | -1.875082 | -2.701173 | 1.603176 |
| 18 | 6 | -2.749206 | -2.663253 | 0.363721 |
| 19 | 6 | 2.502108 | 1.978492 | 1.412797 |
| 20 | 6 | -3.022451 | -1.002042 | -1.396716 |
| 21 | 6 | -3.305285 | 0.148191 | -0.460876 |
| 22 | 6 | 1.730490 | 2.794234 | 0.402539 |
| 23 | 6 | -2.560331 | 1.491390 | 1.271103 |
| 24 | 6 | -3.750765 | 2.207801 | 1.298733 |
| 25 | 6 | -4.748149 | 1.865384 | 0.390541 |
| 26 | 6 | -4.533064 | 0.814171 | -0.485721 |
| 27 | 6 | 1.881506 | 4.176396 | 0.301750 |
| 28 | 6 | 1.149030 | 4.868959 | -0.655075 |
| 29 | 6 | 0.313178 | 4.163541 | -1.513041 |
| 30 | 6 | 0.223157 | 2.784610 | -1.362453 |
| 31 | 82 | 0.180902 | -0.361105 | -0.061907 |
| 32 | 1 | 4.807994 | 0.406014 | 0.065119 |
| 33 | 1 | 3.969394 | 1.841917 | -0.498439 |
| 34 | 1 | 4.190129 | 0.128582 | -2.256525 |
| 35 | 1 | 2.497953 | 0.552279 | -1.929751 |
| 36 | 1 | -1.711763 | -1.683719 | 1.977847 |
| 37 | 1 | -2.390319 | -3.271634 | 2.387019 |
| 38 | 1 | -3.034891 | -3.681225 | 0.080148 |
| 39 | 1 | -3.668744 | -2.139030 | 0.632136 |
| 40 | 1 | 3.386254 | 2.548119 | 1.734007 |
| 41 | 1 | 1.871120 | 1.828519 | 2.291474 |
| 42 | 1 | -3.975282 | -1.460874 | -1.696276 |
| 43 | 1 | -2.548022 | -0.598101 | -2.293589 |
| 44 | 1 | -3.924644 | 3.005870 | 2.007041 |
| 45 | 1 | -5.688454 | 2.401343 | 0.387288 |
| 46 | 1 | -5.303599 | 0.499630 | -1.177850 |
| 47 | 1 | 2.571352 | 4.693163 | 0.957026 |
| 48 | 1 | 1.248824 | 5.943643 | -0.745451 |
| 49 | 1 | -0.250918 | 4.649463 | -2.296532 |

| | | | | |
|---|---|---|---|---|
| 50 | 7 | 0.896518 | 2.132762 | -0.407367 |
| 51 | 7 | -2.341841 | 0.505492 | 0.390579 |
| 52 | 6 | -1.423214 | 1.690521 | 2.236915 |
| 53 | 8 | -0.424288 | 0.957618 | 2.181588 |
| 54 | 6 | -0.597113 | 1.928345 | -2.313859 |
| 55 | 8 | -0.525759 | 0.660510 | -2.133264 |
| 56 | 8 | -1.244636 | 2.495607 | -3.203265 |
| 57 | 1 | -0.545694 | -3.260406 | -3.573873 |
| 58 | 1 | -0.982100 | -1.562068 | -3.288044 |
| 59 | 1 | -2.555039 | -3.381069 | -2.338922 |
| 60 | 1 | -1.236927 | -3.817051 | -1.243351 |
| 61 | 1 | 2.951952 | -1.237813 | 3.736402 |
| 62 | 1 | 1.756478 | 0.005760 | 3.325703 |
| 63 | 1 | 3.906175 | -1.050801 | 1.434326 |
| 64 | 1 | 4.194948 | 0.325407 | 2.505491 |
| 65 | 1 | -0.013200 | -1.778984 | 3.525078 |
| 66 | 1 | 1.334692 | -2.829391 | 3.996453 |
| 67 | 1 | -0.439304 | -4.225997 | 3.140268 |
| 68 | 1 | 0.959832 | -4.286542 | 2.044071 |
| 69 | 1 | 1.497626 | -0.957287 | -3.436043 |
| 70 | 1 | 1.614417 | -2.668208 | -3.894373 |
| 71 | 1 | 3.043054 | -3.156606 | -1.951045 |
| 72 | 1 | 3.776119 | -1.960593 | -3.043956 |
| 73 | 7 | -1.536198 | 2.655405 | 3.154962 |
| 74 | 1 | -2.341985 | 3.252317 | 3.230792 |
| 75 | 1 | -0.790919 | 2.783851 | 3.821985 |

---------------------------------------------------------------------

E(RTPSSh) = -2006.31306274 Hartree
Zero-point correction= 0.617178 (Hartree/Particle)
Thermal correction to Energy= 0.655482
Thermal correction to Enthalpy= 0.656427
Thermal correction to Gibbs Free Energy= 0.545928
Sum of electronic and zero-point Energies= -2005.695885
Sum of electronic and thermal Energies= -2005.657580
Sum of electronic and thermal Enthalpies= -2005.656636
Sum of electronic and thermal Free Energies= -2005.767135

**Table S28**: Cartesian coordinates (Å) of the Δ(δδλ)(δδλ) conformer of **[Pb(macropapam)]**$^{+}$ from geometry optimizations (0 imaginary frequencies).

| Center Number | Atomic Number | Coordinates (Angstroms) X | Y | Z |
|---|---|---|---|---|
| 1 | 6 | -3.709337 | -0.997219 | -0.021262 |
| 2 | 7 | -2.812222 | -0.502115 | -1.081772 |
| 3 | 8 | -2.047025 | -2.489592 | 0.873576 |
| 4 | 6 | -3.414737 | -2.393225 | 0.491223 |
| 5 | 6 | -2.869417 | -1.326194 | -2.307274 |
| 6 | 6 | -1.842536 | -2.441992 | -2.373916 |
| 7 | 8 | -0.541269 | -1.854843 | -2.406469 |
| 8 | 6 | 0.484270 | -2.793942 | -2.715811 |
| 9 | 6 | 1.794515 | -2.039755 | -2.788910 |
| 10 | 8 | 2.175230 | -1.444047 | -1.545247 |
| 11 | 8 | 0.365955 | -2.602198 | 2.180424 |
| 12 | 6 | -0.335102 | -3.809027 | 1.906051 |
| 13 | 6 | -1.814571 | -3.518973 | 1.830739 |
| 14 | 7 | 2.624864 | -0.802679 | 1.341885 |
| 15 | 6 | 2.440624 | -1.499799 | 2.629653 |
| 16 | 6 | 1.720892 | -2.828867 | 2.549024 |
| 17 | 6 | 2.825870 | -2.346182 | -0.645124 |
| 18 | 6 | 3.529175 | -1.537816 | 0.430274 |
| 19 | 6 | -3.180598 | 0.882747 | -1.408537 |
| 20 | 6 | 3.151133 | 0.540034 | 1.630710 |
| 21 | 6 | 3.126730 | 1.465473 | 0.440832 |
| 22 | 6 | -2.981575 | 1.850128 | -0.267070 |
| 23 | 6 | 1.953950 | 2.314039 | -1.366622 |
| 24 | 6 | 2.979737 | 3.200290 | -1.679409 |
| 25 | 6 | 4.122616 | 3.199072 | -0.891392 |
| 26 | 6 | 4.200733 | 2.314490 | 0.176829 |
| 27 | 6 | -3.823680 | 2.957176 | -0.130941 |
| 28 | 6 | -3.594713 | 3.860596 | 0.893453 |
| 29 | 6 | -2.555019 | 3.619792 | 1.785455 |
| 30 | 6 | -1.775038 | 2.484421 | 1.597991 |
| 31 | 82 | -0.001250 | -0.155572 | -0.076325 |
| 32 | 1 | -2.689622 | -0.669325 | -3.159600 |
| 33 | 1 | -3.870787 | -1.759295 | -2.439440 |
| 34 | 1 | -2.010134 | -3.006758 | -3.299362 |
| 35 | 1 | -1.909601 | -3.131840 | -1.529424 |
| 36 | 1 | 0.284621 | -3.257829 | -3.690183 |
| 37 | 1 | 0.514351 | -3.589372 | -1.961428 |
| 38 | 1 | 1.697573 | -1.214053 | -3.495058 |
| 39 | 1 | 2.587109 | -2.710845 | -3.136640 |
| 40 | 1 | -0.164851 | -4.542412 | 2.704669 |
| 41 | 1 | 0.021986 | -4.241438 | 0.962889 |
| 42 | 1 | -2.333976 | -4.440246 | 1.540094 |
| 43 | 1 | -2.196778 | -3.199878 | 2.808213 |
| 44 | 1 | 1.864946 | -0.837790 | 3.278864 |
| 45 | 1 | 3.416032 | -1.675524 | 3.110838 |
| 46 | 1 | 2.202899 | -3.511339 | 1.838960 |
| 47 | 1 | 1.761554 | -3.306532 | 3.536600 |
| 48 | 1 | -4.225776 | 0.955077 | -1.743989 |
| 49 | 1 | -2.538595 | 1.208733 | -2.229484 |

| | | | | |
|---|---|---|---|---|
| 50 | 1 | 4.176393 | 0.497066 | 2.025267 |
| 51 | 1 | 2.517418 | 0.973885 | 2.406983 |
| 52 | 1 | 2.864890 | 3.857038 | -2.529959 |
| 53 | 1 | 4.945850 | 3.867925 | -1.110575 |
| 54 | 1 | 5.082539 | 2.273662 | 0.803667 |
| 55 | 1 | -4.642258 | 3.098443 | -0.825051 |
| 56 | 1 | -4.222430 | 4.734780 | 1.009092 |
| 57 | 1 | -2.368289 | 4.314506 | 2.592653 |
| 58 | 7 | -1.970067 | 1.638316 | 0.577428 |
| 59 | 7 | 2.027210 | 1.478288 | -0.323524 |
| 60 | 6 | 0.701318 | 2.248731 | -2.224467 |
| 61 | 8 | -0.159297 | 1.354088 | -1.912850 |
| 62 | 8 | 0.597073 | 3.047779 | -3.165513 |
| 63 | 6 | -0.653033 | 2.081946 | 2.514577 |
| 64 | 8 | 0.112139 | 1.158115 | 2.205239 |
| 65 | 1 | -3.634144 | -0.304832 | 0.817678 |
| 66 | 1 | -4.754973 | -0.985982 | -0.370026 |
| 67 | 1 | -4.064615 | -2.568227 | 1.356565 |
| 68 | 1 | -3.647108 | -3.164090 | -0.253974 |
| 69 | 1 | 4.184408 | -2.210305 | 0.999717 |
| 70 | 1 | 4.174410 | -0.816985 | -0.075391 |
| 71 | 1 | 3.576943 | -2.924205 | -1.196265 |
| 72 | 1 | 2.101470 | -3.046891 | -0.221141 |
| 73 | 7 | -0.519931 | 2.746180 | 3.668647 |
| 74 | 1 | -1.177849 | 3.440283 | 3.979855 |
| 75 | 1 | 0.214192 | 2.475457 | 4.304667 |

---------------------------------------------------------------------

E(RTPSSh) = -2006.31868292 Hartree
Zero-point correction= 0.617304 (Hartree/Particle)
Thermal correction to Energy= 0.655637
Thermal correction to Enthalpy= 0.656582
Thermal correction to Gibbs Free Energy= 0.545946
Sum of electronic and zero-point Energies= -2005.701379
Sum of electronic and thermal Energies= -2005.663046
Sum of electronic and thermal Enthalpies= -2005.662101
Sum of electronic and thermal Free Energies= -2005.772737

**Table S29**: Cartesian coordinates (Å) of the Δ(λδδ)(λδδ) conformer of **[Pb(macropapam)]$^{+}$** from geometry optimizations (0 imaginary frequencies).

| Center Number | Atomic Number | Atomic Type | Coordinates (Angstroms) X | Y | Z |
|---|---|---|---|---|---|
| 1 | 6 | | -3.730642 | -1.551056 | -0.000770 |
| 2 | 7 | | -2.791604 | -1.051894 | -1.034662 |
| 3 | 8 | | -2.053465 | -1.945132 | 1.647355 |
| 4 | 6 | | -3.308695 | -1.305744 | 1.437369 |
| 5 | 6 | | -2.768569 | -1.983597 | -2.185643 |
| 6 | 6 | | -1.514113 | -1.908767 | -3.030170 |
| 7 | 8 | | -0.421800 | -2.395709 | -2.238877 |
| 8 | 6 | | 0.726738 | -2.816382 | -2.984518 |
| 9 | 6 | | 1.863054 | -1.822260 | -2.897280 |
| 10 | 8 | | 2.203885 | -1.689092 | -1.514449 |
| 11 | 8 | | 0.678339 | -2.473061 | 2.399479 |
| 12 | 6 | | -0.466383 | -2.899548 | 3.136973 |
| 13 | 6 | | -1.646550 | -1.958581 | 3.014651 |
| 14 | 7 | | 2.870101 | -0.805936 | 1.158882 |
| 15 | 6 | | 2.911487 | -1.643434 | 2.379577 |
| 16 | 6 | | 1.608345 | -1.689464 | 3.151611 |
| 17 | 6 | | 3.475066 | -1.078999 | -1.286723 |
| 18 | 6 | | 3.851455 | -1.298709 | 0.168889 |
| 19 | 6 | | -3.166063 | 0.286460 | -1.512233 |
| 20 | 6 | | 3.115151 | 0.591663 | 1.539815 |
| 21 | 6 | | 2.851069 | 1.600853 | 0.450265 |
| 22 | 6 | | -3.012134 | 1.383431 | -0.488962 |
| 23 | 6 | | 1.530687 | 2.289486 | -1.325948 |
| 24 | 6 | | 2.302385 | 3.429211 | -1.523209 |
| 25 | 6 | | 3.393689 | 3.643813 | -0.691316 |
| 26 | 6 | | 3.681669 | 2.710442 | 0.296153 |
| 27 | 6 | | -3.966269 | 2.400456 | -0.401296 |
| 28 | 6 | | -3.791437 | 3.418317 | 0.522146 |
| 29 | 6 | | -2.677846 | 3.388616 | 1.353761 |
| 30 | 6 | | -1.775298 | 2.340149 | 1.211393 |
| 31 | 82 | | 0.035411 | -0.507139 | -0.085259 |
| 32 | 1 | | -3.816432 | -2.630658 | -0.126458 |
| 33 | 1 | | -4.733795 | -1.128391 | -0.149076 |
| 34 | 1 | | -4.066590 | -1.755335 | 2.092260 |
| 35 | 1 | | -3.239002 | -0.243817 | 1.685457 |
| 36 | 1 | | 0.459293 | -2.980796 | -4.032051 |
| 37 | 1 | | 1.047145 | -3.767139 | -2.552204 |
| 38 | 1 | | 1.582431 | -0.848910 | -3.311534 |
| 39 | 1 | | 2.722465 | -2.213676 | -3.454279 |
| 40 | 1 | | -0.209210 | -3.027920 | 4.194342 |
| 41 | 1 | | -0.746256 | -3.872765 | 2.727700 |
| 42 | 1 | | -2.464769 | -2.334271 | 3.642864 |
| 43 | 1 | | -1.392062 | -0.944661 | 3.343660 |
| 44 | 1 | | 3.446147 | -0.024690 | -1.570515 |
| 45 | 1 | | 4.231115 | -1.574518 | -1.907564 |
| 46 | 1 | | 3.943883 | -2.375369 | 0.312354 |
| 47 | 1 | | 4.845527 | -0.860391 | 0.341271 |
| 48 | 1 | | -4.203319 | 0.292767 | -1.877581 |
| 49 | 1 | | -2.511795 | 0.534080 | -2.351281 |

| | | | | |
|---|---|---|---|---|
| 50 | 1 | 4.148298 | 0.723426 | 1.894839 |
| 51 | 1 | 2.449324 | 0.825813 | 2.373945 |
| 52 | 1 | 2.045857 | 4.106383 | -2.325421 |
| 53 | 1 | 4.024385 | 4.514610 | -0.821829 |
| 54 | 1 | 4.543720 | 2.828572 | 0.940310 |
| 55 | 1 | -4.836071 | 2.377514 | -1.045109 |
| 56 | 1 | -4.520116 | 4.213631 | 0.611937 |
| 57 | 1 | -2.548482 | 4.152112 | 2.108385 |
| 58 | 7 | -1.934528 | 1.370511 | 0.300722 |
| 59 | 7 | 1.789325 | 1.416174 | -0.343901 |
| 60 | 6 | 0.383647 | 1.951378 | -2.263486 |
| 61 | 8 | -0.166147 | 0.808066 | -2.091849 |
| 62 | 8 | 0.080395 | 2.774681 | -3.137665 |
| 63 | 6 | -0.579793 | 2.147627 | 2.102572 |
| 64 | 8 | 0.015242 | 1.061724 | 2.117793 |
| 65 | 1 | 1.794614 | -2.176514 | 4.116610 |
| 66 | 1 | 1.206625 | -0.689489 | 3.342160 |
| 67 | 1 | 3.712287 | -1.306701 | 3.054675 |
| 68 | 1 | 3.147368 | -2.664912 | 2.081225 |
| 69 | 1 | -1.648727 | -2.561597 | -3.899160 |
| 70 | 1 | -1.301066 | -0.895218 | -3.379883 |
| 71 | 1 | -2.836497 | -2.997894 | -1.792709 |
| 72 | 1 | -3.641034 | -1.821966 | -2.836542 |
| 73 | 7 | -0.207007 | 3.170886 | 2.880352 |
| 74 | 1 | -0.642336 | 4.076697 | 2.839733 |
| 75 | 1 | 0.583914 | 3.055172 | 3.494922 |

---------------------------------------------------------------------

E(RTPSSh) = -2006.31129381 Hartree
Zero-point correction= 0.617663 (Hartree/Particle)
Thermal correction to Energy= 0.655839
Thermal correction to Enthalpy= 0.656783
Thermal correction to Gibbs Free Energy= 0.546493
Sum of electronic and zero-point Energies= -2005.693631
Sum of electronic and thermal Energies= -2005.655455
Sum of electronic and thermal Enthalpies= -2005.654511
Sum of electronic and thermal Free Energies= -2005.764801

**Table S30**: Cartesian coordinates (Å) of the Δ(δλδ)(δλδ) conformer of **[Pb(macropapam)]$^+$** from geometry optimizations (0 imaginary frequencies).

| Center Number | Atomic Number | Atomic Type | Coordinates (Angstroms) X | Y | Z |
|---|---|---|---|---|---|
| 1 | 6 | | -3.651770 | -1.409721 | 0.384584 |
| 2 | 7 | | -2.831200 | -1.011947 | -0.777216 |
| 3 | 8 | | -1.864814 | -1.962815 | 1.873289 |
| 4 | 6 | | -3.023379 | -2.491699 | 1.237042 |
| 5 | 6 | | -2.946573 | -2.029309 | -1.839083 |
| 6 | 6 | | -1.850051 | -1.971629 | -2.879102 |
| 7 | 8 | | -0.614158 | -2.336352 | -2.263384 |
| 8 | 6 | | 0.434531 | -2.453106 | -3.222446 |
| 9 | 6 | | 1.694666 | -2.880358 | -2.519185 |
| 10 | 8 | | 2.134181 | -1.823972 | -1.667596 |
| 11 | 8 | | 0.941415 | -2.075751 | 2.487186 |
| 12 | 6 | | -0.093211 | -2.255135 | 3.448346 |
| 13 | 6 | | -1.283790 | -2.893396 | 2.781860 |
| 14 | 7 | | 2.964100 | -0.561919 | 0.883273 |
| 15 | 6 | | 3.205365 | -1.451899 | 2.033217 |
| 16 | 6 | | 2.094883 | -1.468078 | 3.061659 |
| 17 | 6 | | 3.350553 | -2.156949 | -0.999979 |
| 18 | 6 | | 3.841517 | -0.945016 | -0.238592 |
| 19 | 6 | | -3.278645 | 0.289659 | -1.286157 |
| 20 | 6 | | 3.209390 | 0.833770 | 1.264905 |
| 21 | 6 | | 2.817558 | 1.811866 | 0.184287 |
| 22 | 6 | | -3.044770 | 1.418390 | -0.313244 |
| 23 | 6 | | 1.370834 | 2.367746 | -1.538841 |
| 24 | 6 | | 2.058421 | 3.547066 | -1.802693 |
| 25 | 6 | | 3.168478 | 3.856034 | -1.026944 |
| 26 | 6 | | 3.562718 | 2.970432 | -0.032396 |
| 27 | 6 | | -3.939675 | 2.489967 | -0.251507 |
| 28 | 6 | | -3.695216 | 3.526605 | 0.633402 |
| 29 | 6 | | -2.583482 | 3.454483 | 1.465854 |
| 30 | 6 | | -1.747757 | 2.348683 | 1.357973 |
| 31 | 82 | | 0.040565 | -0.405194 | -0.075575 |
| 32 | 1 | | -4.346437 | 0.269765 | -1.550525 |
| 33 | 1 | | -2.713751 | 0.510451 | -2.194085 |
| 34 | 1 | | 4.265771 | 0.999833 | 1.524999 |
| 35 | 1 | | 2.615488 | 1.049978 | 2.154933 |
| 36 | 1 | | 1.721223 | 4.184013 | -2.608009 |
| 37 | 1 | | 3.731801 | 4.763578 | -1.206384 |
| 38 | 1 | | 4.442662 | 3.162779 | 0.568388 |
| 39 | 1 | | -4.814145 | 2.497412 | -0.889549 |
| 40 | 1 | | -4.367405 | 4.372883 | 0.691994 |
| 41 | 1 | | -2.394174 | 4.249939 | 2.172999 |
| 42 | 7 | | -1.962748 | 1.369968 | 0.467719 |
| 43 | 7 | | 1.734477 | 1.537101 | -0.552993 |
| 44 | 6 | | 0.186785 | 1.946288 | -2.393925 |
| 45 | 8 | | -0.350748 | 0.821836 | -2.103004 |
| 46 | 8 | | -0.162538 | 2.695395 | -3.317028 |
| 47 | 6 | | -0.548152 | 2.115920 | 2.234977 |
| 48 | 8 | | 0.179197 | 1.129605 | 2.053902 |
| 49 | 1 | | -4.645646 | -1.749492 | 0.056413 |

| | | | | |
|---|---|---|---|---|
| 50 | 1 | -3.751604 | -2.805891 | 1.995838 |
| 51 | 1 | -2.752939 | -3.378330 | 0.650490 |
| 52 | 1 | 4.865942 | -1.139086 | 0.113931 |
| 53 | 1 | 3.891751 | -0.108558 | -0.936715 |
| 54 | 1 | 4.107986 | -2.438688 | -1.741707 |
| 55 | 1 | 3.186361 | -3.021227 | -0.345371 |
| 56 | 1 | 1.843525 | -0.462488 | 3.417007 |
| 57 | 1 | 2.431117 | -2.057990 | 3.924286 |
| 58 | 1 | 3.324500 | -2.467819 | 1.655436 |
| 59 | 1 | 4.146698 | -1.181246 | 2.536584 |
| 60 | 1 | -2.085667 | -2.689796 | -3.674137 |
| 61 | 1 | -1.753374 | -0.978501 | -3.329230 |
| 62 | 1 | -3.919403 | -1.944346 | -2.347281 |
| 63 | 1 | -2.913021 | -3.013050 | -1.370500 |
| 64 | 1 | -0.980058 | -3.803020 | 2.248434 |
| 65 | 1 | -2.017716 | -3.172125 | 3.548747 |
| 66 | 1 | -0.375447 | -1.288406 | 3.882922 |
| 67 | 1 | 0.258687 | -2.908817 | 4.257015 |
| 68 | 1 | -3.800259 | -0.532485 | 1.015067 |
| 69 | 1 | 2.468095 | -3.098808 | -3.265617 |
| 70 | 1 | 1.514859 | -3.787850 | -1.929752 |
| 71 | 1 | 0.167611 | -3.208475 | -3.971783 |
| 72 | 1 | 0.585157 | -1.493604 | -3.730314 |
| 73 | 7 | -0.305047 | 2.992049 | 3.216584 |
| 74 | 1 | -0.903053 | 3.774927 | 3.417698 |
| 75 | 1 | 0.485762 | 2.836464 | 3.822416 |

---------------------------------------------------------------------

E(RTPSSh) = -2006.32862660 Hartree

Zero-point correction= 0.617044 (Hartree/Particle)

Thermal correction to Energy= 0.655301

Thermal correction to Enthalpy= 0.656245

Thermal correction to Gibbs Free Energy= 0.546270

Sum of electronic and zero-point Energies= -2005.711582

Sum of electronic and thermal Energies= -2005.673326

Sum of electronic and thermal Enthalpies= -2005.672382

Sum of electronic and thermal Free Energies= -2005.782356

**Table S31**: Cartesian coordinates (Å) of the Δ(λλλ)(λλλ) conformer of **[Pb(macropam)]$^{2+}$** from geometry optimizations (0 imaginary frequencies).

| Center Number | Atomic Number | Coordinates (Angstroms) X | Y | Z |
|---|---|---|---|---|
| 1 | 6 | -3.538302 | -1.547970 | -0.585826 |
| 2 | 7 | -2.529115 | -0.821451 | -1.384951 |
| 3 | 8 | -2.350231 | -1.311308 | 1.548526 |
| 4 | 6 | -3.603600 | -1.143018 | 0.879815 |
| 5 | 6 | -2.233365 | -1.564400 | -2.632248 |
| 6 | 6 | -1.466915 | -2.859140 | -2.412212 |
| 7 | 8 | -0.154256 | -2.642658 | -1.881419 |
| 8 | 6 | 0.873362 | -2.629093 | -2.876869 |
| 9 | 6 | 2.210830 | -2.581433 | -2.185977 |
| 10 | 8 | 2.351559 | -1.310395 | -1.548918 |
| 11 | 8 | 0.156061 | -2.641933 | 1.881733 |
| 12 | 6 | -0.870953 | -2.627427 | 2.877792 |
| 13 | 6 | -2.208828 | -2.581284 | 2.187587 |
| 14 | 7 | 2.530144 | -0.819580 | 1.384713 |
| 15 | 6 | 2.235016 | -1.562769 | 2.631998 |
| 16 | 6 | 1.469095 | -2.857805 | 2.411909 |
| 17 | 6 | 3.604641 | -1.140540 | -0.880154 |
| 18 | 6 | 3.539892 | -1.545292 | 0.585595 |
| 19 | 6 | -2.986720 | 0.531452 | -1.741812 |
| 20 | 6 | 2.986676 | 0.533719 | 1.741538 |
| 21 | 6 | 2.924375 | 1.542796 | 0.619268 |
| 22 | 6 | -2.924822 | 1.540752 | -0.619725 |
| 23 | 6 | 1.825100 | 2.366559 | -1.239534 |
| 24 | 6 | 2.766809 | 3.373374 | -1.418053 |
| 25 | 6 | 3.821623 | 3.459091 | -0.515589 |
| 26 | 6 | 3.907452 | 2.530098 | 0.507853 |
| 27 | 6 | -3.907893 | 2.528169 | -0.509202 |
| 28 | 6 | -3.822729 | 3.457321 | 0.514124 |
| 29 | 6 | -2.768577 | 3.371697 | 1.417358 |
| 30 | 6 | -1.826687 | 2.364928 | 1.239645 |
| 31 | 82 | -0.000290 | -0.414474 | -0.000089 |
| 32 | 1 | -3.304162 | -2.610427 | -0.634126 |
| 33 | 1 | -4.538467 | -1.420027 | -1.027118 |
| 34 | 1 | -4.372406 | -1.746973 | 1.372405 |
| 35 | 1 | -3.882052 | -0.097837 | 1.001193 |
| 36 | 1 | 3.881764 | -0.095032 | -1.001697 |
| 37 | 1 | 4.374215 | -1.743618 | -1.372658 |
| 38 | 1 | 3.306818 | -2.607983 | 0.634144 |
| 39 | 1 | 4.539996 | -1.416324 | 1.026720 |
| 40 | 1 | -4.010479 | 0.510741 | -2.139508 |
| 41 | 1 | -2.332676 | 0.893782 | -2.537340 |
| 42 | 1 | 4.010324 | 0.513809 | 2.139582 |
| 43 | 1 | 2.332076 | 0.895692 | 2.536762 |
| 44 | 1 | 2.712748 | 4.062066 | -2.249661 |
| 45 | 1 | 4.576033 | 4.226815 | -0.628824 |
| 46 | 1 | 4.732459 | 2.548830 | 1.208195 |
| 47 | 1 | -4.732391 | 2.546864 | -1.210144 |
| 48 | 1 | -4.577151 | 4.225129 | 0.626696 |
| 49 | 1 | -2.715207 | 4.060580 | 2.248849 |

| | | | | |
|---|---|---|---|---|
| 50 | 7 | -1.899326 | 1.482635 | 0.233627 |
| 51 | 7 | 1.898382 | 1.484440 | -0.233446 |
| 52 | 6 | 0.681164 | 2.126860 | -2.185809 |
| 53 | 8 | 0.047227 | 1.061098 | -2.148762 |
| 54 | 6 | -0.683211 | 2.125457 | 2.186534 |
| 55 | 8 | -0.047491 | 1.060811 | 2.148328 |
| 56 | 1 | 0.757460 | -1.765332 | -3.540355 |
| 57 | 1 | 0.813830 | -3.544695 | -3.475197 |
| 58 | 1 | 3.004501 | -2.708617 | -2.930966 |
| 59 | 1 | 2.282469 | -3.393407 | -1.454034 |
| 60 | 1 | -0.755041 | -1.762693 | 3.540000 |
| 61 | 1 | -0.810698 | -3.542180 | 3.477337 |
| 62 | 1 | -2.280707 | -3.394420 | 1.456958 |
| 63 | 1 | -3.002033 | -2.707423 | 2.933241 |
| 64 | 1 | 1.977096 | -3.517808 | 1.708063 |
| 65 | 1 | 1.399647 | -3.387652 | 3.366462 |
| 66 | 1 | 3.163561 | -1.798835 | 3.173745 |
| 67 | 1 | 1.642040 | -0.905585 | 3.269946 |
| 68 | 1 | -1.396825 | -3.388557 | -3.366949 |
| 69 | 1 | -1.974932 | -3.519682 | -1.708872 |
| 70 | 1 | -3.161708 | -1.800771 | -3.174197 |
| 71 | 1 | -1.640519 | -0.906869 | -3.269965 |
| 72 | 7 | 0.393200 | 3.085022 | -3.071755 |
| 73 | 1 | -0.360872 | 2.938272 | -3.725030 |
| 74 | 1 | 0.850910 | 3.980808 | -3.075997 |
| 75 | 7 | -0.397390 | 3.082585 | 3.074290 |
| 76 | 1 | -0.856863 | 3.977448 | 3.080300 |
| 77 | 1 | 0.356240 | 2.935668 | 3.728037 |

---------------------------------------------------------------------

E(RTPSSh) = -1986.89251202 Hartree
Zero-point correction= 0.643053 (Hartree/Particle)
Thermal correction to Energy= 0.681801
Thermal correction to Enthalpy= 0.682745
Thermal correction to Gibbs Free Energy= 0.571570
Sum of electronic and zero-point Energies= -1986.249459
Sum of electronic and thermal Energies= -1986.210711
Sum of electronic and thermal Enthalpies= -1986.209767
Sum of electronic and thermal Free Energies= -1986.320943

**Table S32**: Cartesian coordinates (Å) of the Δ(δδδ)(δδδ) conformer of **[Pb(macropam)]$^{2+}$** from geometry optimizations (0 imaginary frequencies).

| Center Number | Atomic Number | Coordinates (Angstroms) X | Y | Z |
|---|---|---|---|---|
| 1 | 6 | 3.789231 | -0.744819 | -0.286797 |
| 2 | 7 | 2.875750 | -0.545285 | 0.859557 |
| 3 | 8 | 2.123076 | -1.239422 | -1.912876 |
| 4 | 6 | 3.281697 | -1.777942 | -1.269847 |
| 5 | 6 | 3.108648 | -1.589558 | 1.879731 |
| 6 | 6 | 1.884622 | -1.919654 | 2.706959 |
| 7 | 8 | 0.932700 | -2.546735 | 1.838301 |
| 8 | 6 | -0.111900 | -3.251125 | 2.516610 |
| 9 | 6 | -1.223617 | -2.327449 | 2.981700 |
| 10 | 8 | -1.841713 | -1.637592 | 1.892185 |
| 11 | 8 | -0.427496 | -2.659284 | -1.868334 |
| 12 | 6 | 0.732477 | -3.148014 | -2.548673 |
| 13 | 6 | 1.647088 | -2.026593 | -3.008088 |
| 14 | 7 | -2.716731 | -1.074017 | -0.880253 |
| 15 | 6 | -2.745726 | -2.130176 | -1.914092 |
| 16 | 6 | -1.477651 | -2.214424 | -2.736541 |
| 17 | 6 | -2.867350 | -2.386619 | 1.233971 |
| 18 | 6 | -3.570367 | -1.460575 | 0.264278 |
| 19 | 6 | 3.061386 | 0.790639 | 1.442759 |
| 20 | 6 | -3.156697 | 0.207772 | -1.445775 |
| 21 | 6 | -2.972881 | 1.358440 | -0.483705 |
| 22 | 6 | 2.673185 | 1.894241 | 0.486771 |
| 23 | 6 | -1.807300 | 2.257728 | 1.301340 |
| 24 | 6 | -2.619453 | 3.385307 | 1.333334 |
| 25 | 6 | -3.646426 | 3.485112 | 0.399863 |
| 26 | 6 | -3.842613 | 2.451709 | -0.501847 |
| 27 | 6 | 3.362257 | 3.109274 | 0.480197 |
| 28 | 6 | 2.991876 | 4.090650 | -0.424928 |
| 29 | 6 | 1.967807 | 3.827652 | -1.328884 |
| 30 | 6 | 1.331876 | 2.592894 | -1.264570 |
| 31 | 82 | 0.047735 | -0.459701 | -0.003543 |
| 32 | 1 | 0.300788 | -3.794285 | 3.374311 |
| 33 | 1 | -0.489584 | -3.980107 | 1.798364 |
| 34 | 1 | -0.829957 | -1.551219 | 3.639260 |
| 35 | 1 | -1.973667 | -2.902307 | 3.536460 |
| 36 | 1 | 0.431356 | -3.756367 | -3.409035 |
| 37 | 1 | 1.242913 | -3.794142 | -1.833156 |
| 38 | 1 | 2.492626 | -2.443998 | -3.566261 |
| 39 | 1 | 1.112274 | -1.335400 | -3.660795 |
| 40 | 1 | 4.102321 | 0.949471 | 1.758601 |
| 41 | 1 | 2.434677 | 0.863621 | 2.334260 |
| 42 | 1 | -4.212453 | 0.171212 | -1.750656 |
| 43 | 1 | -2.565585 | 0.405885 | -2.342706 |
| 44 | 1 | -2.472318 | 4.177539 | 2.054196 |
| 45 | 1 | -4.296549 | 4.350368 | 0.396070 |
| 46 | 1 | -4.659944 | 2.479864 | -1.211020 |
| 47 | 1 | 4.182525 | 3.267839 | 1.168408 |
| 48 | 1 | 3.507671 | 5.041859 | -0.447965 |
| 49 | 1 | 1.699215 | 4.575801 | -2.061745 |

| | | | | |
|---|---|---|---|---|
| 50 | 7 | 1.664468 | 1.665789 | -0.356793 |
| 51 | 7 | -1.963605 | 1.291711 | 0.386547 |
| 52 | 6 | -0.710719 | 1.978245 | 2.292429 |
| 53 | 8 | 0.038141 | 0.998884 | 2.146531 |
| 54 | 6 | 0.256931 | 2.145722 | -2.218026 |
| 55 | 8 | -0.227198 | 1.005551 | -2.129837 |
| 56 | 1 | 4.789422 | -1.036419 | 0.061199 |
| 57 | 1 | 4.055417 | -1.970359 | -2.021555 |
| 58 | 1 | 3.045742 | -2.726171 | -0.774345 |
| 59 | 1 | -4.495234 | -1.938106 | -0.086630 |
| 60 | 1 | -3.854198 | -0.559639 | 0.807964 |
| 61 | 1 | -3.587420 | -2.744840 | 1.978261 |
| 62 | 1 | -2.443310 | -3.257765 | 0.722671 |
| 63 | 1 | -1.222075 | -1.251891 | -3.191334 |
| 64 | 1 | -1.620253 | -2.947570 | -3.537825 |
| 65 | 1 | -2.879140 | -3.089427 | -1.413160 |
| 66 | 1 | -3.604658 | -1.990157 | -2.584802 |
| 67 | 1 | 2.168130 | -2.619994 | 3.500043 |
| 68 | 1 | 1.454024 | -1.027106 | 3.172191 |
| 69 | 1 | 3.931098 | -1.298952 | 2.547870 |
| 70 | 1 | 3.415534 | -2.500748 | 1.365442 |
| 71 | 1 | 3.891597 | 0.200646 | -0.819311 |
| 72 | 7 | -0.143038 | 2.998834 | -3.163898 |
| 73 | 1 | -0.846067 | 2.702219 | -3.823332 |
| 74 | 1 | 0.234800 | 3.925088 | -3.265765 |
| 75 | 7 | -0.583635 | 2.800996 | 3.337578 |
| 76 | 1 | -1.217230 | 3.561004 | 3.518271 |
| 77 | 1 | 0.132388 | 2.612002 | 4.022171 |

---------------------------------------------------------------------

E(RTPSSh) = -1986.89526774 Hartree
Zero-point correction= 0.642756 (Hartree/Particle)
Thermal correction to Energy= 0.681523
Thermal correction to Enthalpy= 0.682467
Thermal correction to Gibbs Free Energy= 0.569643
Sum of electronic and zero-point Energies= -1986.252512
Sum of electronic and thermal Energies= -1986.213745
Sum of electronic and thermal Enthalpies= -1986.212801
Sum of electronic and thermal Free Energies= -1986.325625

**Table S33**: Cartesian coordinates (Å) of the Δ(δλλ)(δλλ) conformer of **[Pb(macropam)]$^{2+}$** from geometry optimizations (0 imaginary frequencies).

| Center Number | Atomic Number | Coordinates (Angstroms) X | Y | Z |
|---|---|---|---|---|
| 1 | 6 | -3.667839 | -1.107082 | -0.123997 |
| 2 | 7 | -2.703718 | -0.566987 | -1.109834 |
| 3 | 8 | -1.992931 | -1.890501 | 1.426883 |
| 4 | 6 | -3.189246 | -2.258295 | 0.740526 |
| 5 | 6 | -2.676728 | -1.310625 | -2.386662 |
| 6 | 6 | -1.979447 | -2.661692 | -2.341693 |
| 7 | 8 | -0.613068 | -2.578890 | -1.924957 |
| 8 | 6 | 0.320337 | -2.421192 | -2.999344 |
| 9 | 6 | 1.697388 | -2.721418 | -2.469081 |
| 10 | 8 | 2.066632 | -1.733121 | -1.502696 |
| 11 | 8 | 0.723977 | -2.646208 | 1.830794 |
| 12 | 6 | -0.227914 | -2.582765 | 2.898442 |
| 13 | 6 | -1.586456 | -2.913770 | 2.339757 |
| 14 | 7 | 2.716393 | -0.507650 | 1.100995 |
| 15 | 6 | 2.709406 | -1.283951 | 2.358483 |
| 16 | 6 | 2.086973 | -2.668322 | 2.266833 |
| 17 | 6 | 3.254974 | -2.113077 | -0.808490 |
| 18 | 6 | 3.703874 | -0.990754 | 0.108507 |
| 19 | 6 | -3.075542 | 0.826726 | -1.408520 |
| 20 | 6 | 3.036271 | 0.890304 | 1.439964 |
| 21 | 6 | 2.804404 | 1.844255 | 0.295247 |
| 22 | 6 | -2.857372 | 1.763367 | -0.246314 |
| 23 | 6 | 1.565393 | 2.417535 | -1.567735 |
| 24 | 6 | 2.346766 | 3.541898 | -1.807064 |
| 25 | 6 | 3.395792 | 3.817106 | -0.936530 |
| 26 | 6 | 3.638485 | 2.951041 | 0.116063 |
| 27 | 6 | -3.688742 | 2.872600 | -0.067267 |
| 28 | 6 | -3.454864 | 3.727494 | 0.996218 |
| 29 | 6 | -2.423434 | 3.434963 | 1.882629 |
| 30 | 6 | -1.649991 | 2.304597 | 1.647162 |
| 31 | 82 | 0.007565 | -0.418816 | -0.005213 |
| 32 | 1 | -4.124230 | 0.903002 | -1.728193 |
| 33 | 1 | -2.456223 | 1.162457 | -2.241195 |
| 34 | 1 | 4.076530 | 0.993197 | 1.778543 |
| 35 | 1 | 2.391875 | 1.183193 | 2.269915 |
| 36 | 1 | 2.173025 | 4.191846 | -2.653206 |
| 37 | 1 | 4.025547 | 4.682572 | -1.096957 |
| 38 | 1 | 4.468432 | 3.113838 | 0.791480 |
| 39 | 1 | -4.506887 | 3.048415 | -0.753826 |
| 40 | 1 | -4.077585 | 4.598863 | 1.152137 |
| 41 | 1 | -2.251427 | 4.081974 | 2.731629 |
| 42 | 7 | -1.846855 | 1.508017 | 0.586531 |
| 43 | 7 | 1.778454 | 1.603591 | -0.523837 |
| 44 | 6 | 0.452367 | 1.967708 | -2.471299 |
| 45 | 8 | -0.137994 | 0.897197 | -2.253948 |
| 46 | 6 | -0.559677 | 1.830121 | 2.565860 |
| 47 | 8 | 0.094218 | 0.810980 | 2.291290 |
| 48 | 1 | -4.593600 | -1.421972 | -0.626736 |
| 49 | 1 | -3.979114 | -2.470578 | 1.471845 |

| | | | | |
|---|---|---|---|---|
| 50 | 1 | -3.013314 | -3.177457 | 0.175830 |
| 51 | 1 | 4.627356 | -1.311231 | 0.611504 |
| 52 | 1 | 3.963268 | -0.143496 | -0.527338 |
| 53 | 1 | 4.060625 | -2.288556 | -1.532170 |
| 54 | 1 | 3.077765 | -3.054719 | -0.282720 |
| 55 | 1 | -1.551054 | -3.877309 | 1.818426 |
| 56 | 1 | -2.311720 | -2.984627 | 3.158852 |
| 57 | 1 | -0.228327 | -1.589022 | 3.357437 |
| 58 | 1 | 0.026497 | -3.325346 | 3.662338 |
| 59 | 1 | -3.930281 | -0.290194 | 0.549046 |
| 60 | 1 | 2.419146 | -2.716315 | -3.294171 |
| 61 | 1 | 1.706509 | -3.712264 | -2.000681 |
| 62 | 1 | 0.088718 | -3.135980 | -3.796208 |
| 63 | 1 | 0.272051 | -1.407018 | -3.407945 |
| 64 | 1 | -3.699185 | -1.470530 | -2.762668 |
| 65 | 1 | -2.157490 | -0.678275 | -3.108029 |
| 66 | 1 | -2.033604 | -3.105224 | -3.341239 |
| 67 | 1 | -2.466622 | -3.350044 | -1.654343 |
| 68 | 1 | 2.622121 | -3.310114 | 1.569797 |
| 69 | 1 | 2.150226 | -3.135577 | 3.254892 |
| 70 | 1 | 2.147180 | -0.697981 | 3.086756 |
| 71 | 1 | 3.733732 | -1.399534 | 2.745733 |
| 72 | 7 | -0.332440 | 2.520094 | 3.686547 |
| 73 | 1 | -0.866446 | 3.329510 | 3.952424 |
| 74 | 1 | 0.377277 | 2.195604 | 4.325281 |
| 75 | 7 | 0.136727 | 2.736120 | -3.517055 |
| 76 | 1 | -0.588750 | 2.431203 | -4.147663 |
| 77 | 1 | 0.589990 | 3.611976 | -3.713637 |

---------------------------------------------------------------------

E(RTPSSh) = -1986.89275912 Hartree
Zero-point correction= 0.643033 (Hartree/Particle)
Thermal correction to Energy= 0.682042
Thermal correction to Enthalpy= 0.682986
Thermal correction to Gibbs Free Energy= 0.569721
Sum of electronic and zero-point Energies= -1986.249726
Sum of electronic and thermal Energies= -1986.210717
Sum of electronic and thermal Enthalpies= -1986.209773
Sum of electronic and thermal Free Energies= -1986.323038

**Table S34**: Cartesian coordinates (Å) of the Δ(λδλ)(λδλ) conformer of **[Pb(macropam)]$^{2+}$** from geometry optimizations (0 imaginary frequencies).

| Center Number | Atomic Number | Atomic Type | Coordinates (Angstroms) X | Y | Z |
|---|---|---|---|---|---|
| 1 | 6 | | 3.083454 | 1.794810 | 1.716957 |
| 2 | 7 | | 2.138553 | 2.098899 | 0.624804 |
| 3 | 8 | | 2.421183 | -0.476392 | 2.038601 |
| 4 | 6 | | 3.547653 | 0.356919 | 1.783492 |
| 5 | 6 | | 1.497356 | 3.409062 | 0.880109 |
| 6 | 6 | | 0.505028 | 3.415547 | 2.021293 |
| 7 | 8 | | -0.607977 | 2.590478 | 1.687560 |
| 8 | 6 | | -1.593419 | 2.604642 | 2.717364 |
| 9 | 6 | | -2.799625 | 1.834492 | 2.252782 |
| 10 | 8 | | -2.421297 | 0.476686 | 2.038670 |
| 11 | 8 | | 0.607866 | -2.590180 | 1.688074 |
| 12 | 6 | | 1.593363 | -2.604190 | 2.717829 |
| 13 | 6 | | 2.799556 | -1.834144 | 2.253024 |
| 14 | 7 | | -2.138659 | -2.098795 | 0.625122 |
| 15 | 6 | | -1.497493 | -3.408897 | 0.880833 |
| 16 | 6 | | -0.505236 | -3.415016 | 2.022079 |
| 17 | 6 | | -3.547793 | -0.356526 | 1.783374 |
| 18 | 6 | | -3.083767 | -1.794482 | 1.717038 |
| 19 | 6 | | 2.828856 | 2.180485 | -0.669808 |
| 20 | 6 | | -2.828752 | -2.180634 | -0.669582 |
| 21 | 6 | | -3.149428 | -0.862490 | -1.330425 |
| 22 | 6 | | 3.149506 | 0.862222 | -1.330437 |
| 23 | 6 | | -2.438805 | 1.222463 | -2.025520 |
| 24 | 6 | | -3.620674 | 1.455139 | -2.719081 |
| 25 | 6 | | -4.602904 | 0.471447 | -2.701316 |
| 26 | 6 | | -4.363407 | -0.700465 | -2.004865 |
| 27 | 6 | | 4.363510 | 0.700088 | -2.004819 |
| 28 | 6 | | 4.603095 | -0.471997 | -2.700940 |
| 29 | 6 | | 3.620879 | -1.455707 | -2.718536 |
| 30 | 6 | | 2.438922 | -1.222841 | -2.025207 |
| 31 | 82 | | 0.000047 | -0.000001 | 0.219754 |
| 32 | 1 | | 2.590394 | 2.022023 | 2.661385 |
| 33 | 1 | | 3.969848 | 2.443384 | 1.643904 |
| 34 | 1 | | 4.265520 | 0.266175 | 2.608170 |
| 35 | 1 | | 4.055400 | 0.034879 | 0.868130 |
| 36 | 1 | | 0.969769 | 3.695692 | -0.029211 |
| 37 | 1 | | 2.257512 | 4.178039 | 1.085142 |
| 38 | 1 | | 0.163168 | 4.447492 | 2.169927 |
| 39 | 1 | | 0.944968 | 3.077062 | 2.966484 |
| 40 | 1 | | -1.890469 | 3.637843 | 2.935306 |
| 41 | 1 | | -1.183630 | 2.160505 | 3.632744 |
| 42 | 1 | | -3.200962 | 2.263739 | 1.326884 |
| 43 | 1 | | -3.576027 | 1.887563 | 3.025527 |
| 44 | 1 | | 1.890386 | -3.637363 | 2.935937 |
| 45 | 1 | | 1.183643 | -2.159869 | 3.633150 |
| 46 | 1 | | 3.575984 | -1.886996 | 3.025754 |
| 47 | 1 | | 3.200873 | -2.263611 | 1.327222 |
| 48 | 1 | | -0.969840 | -3.695805 | -0.028362 |
| 49 | 1 | | -2.257671 | -4.177812 | 1.086027 |

| | | | | |
|---|---|---|---|---|
| 50 | 1 | -0.945198 | -3.076078 | 2.967097 |
| 51 | 1 | -0.163474 | -4.446926 | 2.171165 |
| 52 | 1 | -4.055308 | -0.034483 | 0.867885 |
| 53 | 1 | -4.265827 | -0.265651 | 2.607897 |
| 54 | 1 | -2.590983 | -2.021727 | 2.661603 |
| 55 | 1 | -3.970233 | -2.442941 | 1.643804 |
| 56 | 1 | 3.754026 | 2.767773 | -0.588586 |
| 57 | 1 | 2.164232 | 2.711098 | -1.355288 |
| 58 | 1 | -3.753873 | -2.768014 | -0.588420 |
| 59 | 1 | -2.163959 | -2.711246 | -1.354897 |
| 60 | 1 | -3.790825 | 2.367845 | -3.272681 |
| 61 | 1 | -5.536446 | 0.621235 | -3.227935 |
| 62 | 1 | -5.102166 | -1.490738 | -1.974246 |
| 63 | 1 | 5.102223 | 1.490411 | -1.974377 |
| 64 | 1 | 5.536688 | -0.621921 | -3.227431 |
| 65 | 1 | 3.791166 | -2.368635 | -3.271727 |
| 66 | 7 | 2.214185 | -0.091633 | -1.340214 |
| 67 | 7 | -2.214109 | 0.091372 | -1.340324 |
| 68 | 6 | -1.282370 | 2.179965 | -2.012472 |
| 69 | 8 | -0.217624 | 1.870019 | -1.452240 |
| 70 | 6 | 1.282435 | -2.180275 | -2.012150 |
| 71 | 8 | 0.217910 | -1.870550 | -1.451358 |
| 72 | 7 | -1.421080 | 3.354330 | -2.629959 |
| 73 | 1 | -2.268664 | 3.637491 | -3.090921 |
| 74 | 1 | -0.643331 | 3.996257 | -2.639919 |
| 75 | 7 | 1.420829 | -3.354365 | -2.630206 |
| 76 | 1 | 0.643047 | -3.996256 | -2.640103 |
| 77 | 1 | 2.268058 | -3.637214 | -3.092014 |

---------------------------------------------------------------------

E(RTPSSh) = -1986.89884254
Zero-point correction= 0.643160 (Hartree/Particle)
Thermal correction to Energy= 0.681885
Thermal correction to Enthalpy= 0.682829
Thermal correction to Gibbs Free Energy= 0.572335
Sum of electronic and zero-point Energies= -1986.255683
Sum of electronic and thermal Energies= -1986.216958
Sum of electronic and thermal Enthalpies= -1986.216013
Sum of electronic and thermal Free Energies= -1986.326508

**Table S35**: Cartesian coordinates (Å) of the Δ(λλδ)(λλδ) conformer of **[Pb(macropam)]$^{2+}$** from geometry optimizations (0 imaginary frequencies).

| Center Number | Atomic Number | Coordinates (Angstroms) X | Y | Z |
|---|---|---|---|---|
| 1 | 6 | -1.025837 | 3.685028 | 0.488811 |
| 2 | 7 | -0.720365 | 2.847397 | -0.702637 |
| 3 | 8 | -2.518565 | 1.995476 | 1.287683 |
| 4 | 6 | -1.469307 | 2.875378 | 1.693973 |
| 5 | 6 | -1.779430 | 3.018537 | -1.720169 |
| 6 | 6 | -1.825244 | 1.933242 | -2.773389 |
| 7 | 8 | -2.245492 | 0.715204 | -2.151118 |
| 8 | 6 | -2.529942 | -0.320260 | -3.094119 |
| 9 | 6 | -3.265086 | -1.428066 | -2.368707 |
| 10 | 8 | -2.522615 | -1.991504 | -1.287151 |
| 11 | 8 | -2.246431 | -0.711591 | 2.151778 |
| 12 | 6 | -2.528957 | 0.324385 | 3.094738 |
| 13 | 6 | -3.262113 | 1.433473 | 2.369286 |
| 14 | 7 | -0.725269 | -2.846374 | 0.702982 |
| 15 | 6 | -1.784252 | -3.015692 | 1.720850 |
| 16 | 6 | -1.827933 | -1.930229 | 2.774002 |
| 17 | 6 | -1.474703 | -2.872987 | -1.693487 |
| 18 | 6 | -1.032504 | -3.683415 | -0.488384 |
| 19 | 6 | 0.598070 | 3.178086 | -1.253938 |
| 20 | 6 | 0.592760 | -3.179168 | 1.253899 |
| 21 | 6 | 1.706367 | -2.803629 | 0.306135 |
| 22 | 6 | 1.711212 | 2.800788 | -0.306333 |
| 23 | 6 | 2.442439 | -1.420170 | -1.395728 |
| 24 | 6 | 3.652356 | -2.095483 | -1.495772 |
| 25 | 6 | 3.883240 | -3.164889 | -0.635363 |
| 26 | 6 | 2.895430 | -3.535978 | 0.261998 |
| 27 | 6 | 2.901206 | 3.531598 | -0.261980 |
| 28 | 6 | 3.888488 | 3.159069 | 0.635369 |
| 29 | 6 | 3.656194 | 2.089722 | 1.495462 |
| 30 | 6 | 2.445463 | 1.415911 | 1.395144 |
| 31 | 82 | -0.527716 | 0.000677 | -0.000058 |
| 32 | 1 | -1.799684 | 4.413261 | 0.230120 |
| 33 | 1 | -0.139142 | 4.253126 | 0.778140 |
| 34 | 1 | -1.830922 | 3.555546 | 2.474081 |
| 35 | 1 | -0.628998 | 2.303707 | 2.103614 |
| 36 | 1 | -0.633523 | -2.302565 | -2.103063 |
| 37 | 1 | -1.837350 | -3.552544 | -2.473656 |
| 38 | 1 | -1.807706 | -4.410177 | -0.229586 |
| 39 | 1 | -0.146956 | -4.253236 | -0.777865 |
| 40 | 1 | 0.681956 | 4.247813 | -1.491247 |
| 41 | 1 | 0.731902 | 2.625715 | -2.186161 |
| 42 | 1 | 0.675019 | -4.249037 | 1.491179 |
| 43 | 1 | 0.727701 | -2.627048 | 2.186117 |
| 44 | 1 | 4.403471 | -1.825181 | -2.224826 |
| 45 | 1 | 4.814453 | -3.713942 | -0.687232 |
| 46 | 1 | 3.028525 | -4.388329 | 0.915822 |
| 47 | 1 | 3.035385 | 4.383948 | -0.915582 |
| 48 | 1 | 4.820359 | 3.706980 | 0.687502 |
| 49 | 1 | 4.406807 | 1.818403 | 2.224654 |

| | | | | |
|---|---|---|---|---|
| 50 | 7 | 1.512354 | 1.751995 | 0.493941 |
| 51 | 7 | 1.508918 | -1.754784 | -0.494399 |
| 52 | 6 | 2.014836 | -0.300613 | -2.303018 |
| 53 | 8 | 0.876326 | 0.187120 | -2.195764 |
| 54 | 6 | 2.016529 | 0.296657 | 2.302200 |
| 55 | 8 | 0.876734 | -0.188270 | 2.196262 |
| 56 | 1 | -2.564390 | -2.215782 | 3.533622 |
| 57 | 1 | -0.864397 | -1.780480 | 3.270859 |
| 58 | 1 | -1.681905 | -3.987963 | 2.224869 |
| 59 | 1 | -2.738349 | -3.012630 | 1.195099 |
| 60 | 1 | -2.561543 | 2.220039 | -3.532690 |
| 61 | 1 | -0.862146 | 1.781969 | -3.270622 |
| 62 | 1 | -2.733347 | 3.017020 | -1.194086 |
| 63 | 1 | -1.675593 | 3.990673 | -2.224119 |
| 64 | 1 | -1.600822 | -0.672173 | -3.556205 |
| 65 | 1 | -3.179512 | 0.070506 | -3.886141 |
| 66 | 1 | -3.537783 | -2.211214 | -3.085372 |
| 67 | 1 | -4.174776 | -1.027492 | -1.920095 |
| 68 | 1 | -1.599192 | 0.674685 | 3.556766 |
| 69 | 1 | -3.179190 | -0.065153 | 3.886831 |
| 70 | 1 | -4.172525 | 1.034545 | 1.920673 |
| 71 | 1 | -3.533368 | 2.217186 | 3.085868 |
| 72 | 7 | 2.876921 | -0.136908 | 3.224854 |
| 73 | 1 | 3.802726 | 0.241023 | 3.333314 |
| 74 | 1 | 2.591624 | -0.869883 | 3.856171 |
| 75 | 7 | 2.874772 | 0.130280 | -3.227297 |
| 76 | 1 | 2.590092 | 0.863348 | -3.858784 |
| 77 | 1 | 3.799110 | -0.250656 | -3.337693 |

---------------------------------------------------------------------

E(RTPSSh) = -1986.88765603 Hartree
Zero-point correction= 0.643004 (Hartree/Particle)
Thermal correction to Energy= 0.681837
Thermal correction to Enthalpy= 0.682781
Thermal correction to Gibbs Free Energy= 0.571943
Sum of electronic and zero-point Energies= -1986.244652
Sum of electronic and thermal Energies= -1986.205819
Sum of electronic and thermal Enthalpies= -1986.204875
Sum of electronic and thermal Free Energies= -1986.315713

**Table S36**: Cartesian coordinates (Å) of the Δ(δδλ)(δδλ) conformer of **[Pb(macropam)]$^{2+}$** from geometry optimizations (0 imaginary frequencies).

| Center Number | Atomic Number | Coordinates (Angstroms) X | Y | Z |
|---|---|---|---|---|
| 1 | 6 | 3.814030 | -0.254254 | -0.050143 |
| 2 | 7 | 2.858955 | 0.085908 | 1.022074 |
| 3 | 8 | 2.437717 | -2.060868 | -0.832487 |
| 4 | 6 | 3.777886 | -1.695511 | -0.513349 |
| 5 | 6 | 3.111339 | -0.668113 | 2.269989 |
| 6 | 6 | 2.320307 | -1.956756 | 2.403507 |
| 7 | 8 | 0.931096 | -1.625170 | 2.419621 |
| 8 | 6 | 0.096916 | -2.708264 | 2.824227 |
| 9 | 6 | -1.320282 | -2.184075 | 2.904812 |
| 10 | 8 | -1.821054 | -1.724490 | 1.644728 |
| 11 | 8 | 0.050594 | -2.681437 | -1.976742 |
| 12 | 6 | 0.974908 | -3.734452 | -1.716545 |
| 13 | 6 | 2.372888 | -3.168071 | -1.730036 |
| 14 | 7 | -2.442008 | -1.269883 | -1.219765 |
| 15 | 6 | -2.169277 | -1.978373 | -2.487762 |
| 16 | 6 | -1.233936 | -3.159218 | -2.363722 |
| 17 | 6 | -2.360935 | -2.758565 | 0.816432 |
| 18 | 6 | -3.204275 | -2.109749 | -0.265186 |
| 19 | 6 | 2.950061 | 1.527555 | 1.292248 |
| 20 | 6 | -3.190966 | -0.046928 | -1.549121 |
| 21 | 6 | -3.297142 | 0.920425 | -0.398924 |
| 22 | 6 | 2.490042 | 2.390469 | 0.141717 |
| 23 | 6 | -2.262440 | 2.038483 | 1.335054 |
| 24 | 6 | -3.425513 | 2.728745 | 1.657838 |
| 25 | 6 | -4.566959 | 2.490899 | 0.901829 |
| 26 | 6 | -4.503110 | 1.574114 | -0.134478 |
| 27 | 6 | 3.029961 | 3.667795 | -0.033351 |
| 28 | 6 | 2.569336 | 4.460960 | -1.070235 |
| 29 | 6 | 1.612932 | 3.946949 | -1.940291 |
| 30 | 6 | 1.143078 | 2.658703 | -1.717169 |
| 31 | 82 | 0.081583 | -0.276365 | 0.005665 |
| 32 | 1 | 2.831737 | -0.028225 | 3.107858 |
| 33 | 1 | 4.180433 | -0.894967 | 2.378694 |
| 34 | 1 | 2.593265 | -2.428982 | 3.354719 |
| 35 | 1 | 2.517694 | -2.665093 | 1.595689 |
| 36 | 1 | 0.403762 | -3.065190 | 3.815115 |
| 37 | 1 | 0.184636 | -3.543080 | 2.118891 |
| 38 | 1 | -1.347886 | -1.317776 | 3.566756 |
| 39 | 1 | -1.981013 | -2.958899 | 3.306733 |
| 40 | 1 | 0.901765 | -4.505716 | -2.492252 |
| 41 | 1 | 0.749425 | -4.196376 | -0.748095 |
| 42 | 1 | 3.073022 | -3.952806 | -1.421091 |
| 43 | 1 | 2.646360 | -2.832838 | -2.737323 |
| 44 | 1 | -1.722489 | -1.256447 | -3.172607 |
| 45 | 1 | -3.109679 | -2.334252 | -2.935282 |
| 46 | 1 | -1.600405 | -3.901420 | -1.645269 |
| 47 | 1 | -1.164564 | -3.654924 | -3.339714 |
| 48 | 1 | 3.975483 | 1.825032 | 1.555910 |
| 49 | 1 | 2.312240 | 1.746559 | 2.150117 |

| | | | | |
|---|---|---|---|---|
| 50 | 1 | -4.201667 | -0.279231 | -1.911131 |
| 51 | 1 | -2.656396 | 0.451630 | -2.360048 |
| 52 | 1 | -3.466203 | 3.417719 | 2.489906 |
| 53 | 1 | -5.492280 | 3.003073 | 1.131481 |
| 54 | 1 | -5.375876 | 1.350288 | -0.734021 |
| 55 | 1 | 3.799497 | 4.023274 | 0.639869 |
| 56 | 1 | 2.958660 | 5.460263 | -1.215558 |
| 57 | 1 | 1.254613 | 4.552973 | -2.760738 |
| 58 | 7 | 1.551030 | 1.915040 | -0.678313 |
| 59 | 7 | -2.200727 | 1.157996 | 0.327017 |
| 60 | 6 | -0.982266 | 2.175921 | 2.105764 |
| 61 | 8 | -0.056328 | 1.366509 | 1.933592 |
| 62 | 6 | 0.165604 | 1.956400 | -2.616880 |
| 63 | 8 | -0.301392 | 0.851721 | -2.297814 |
| 64 | 1 | 3.586477 | 0.384678 | -0.903783 |
| 65 | 1 | 4.843868 | -0.030108 | 0.269873 |
| 66 | 1 | 4.413320 | -1.775069 | -1.402442 |
| 67 | 1 | 4.177972 | -2.383736 | 0.240705 |
| 68 | 1 | -3.754432 | -2.893275 | -0.801444 |
| 69 | 1 | -3.945263 | -1.483098 | 0.233942 |
| 70 | 1 | -3.005005 | -3.407114 | 1.420882 |
| 71 | 1 | -1.556311 | -3.371396 | 0.400545 |
| 72 | 7 | -0.877208 | 3.175008 | 2.984653 |
| 73 | 1 | -1.586373 | 3.878720 | 3.101587 |
| 74 | 1 | -0.025324 | 3.272259 | 3.515728 |
| 75 | 7 | -0.168701 | 2.547327 | -3.767488 |
| 76 | 1 | -0.804057 | 2.073508 | -4.391172 |
| 77 | 1 | 0.253621 | 3.401555 | -4.089364 |

---------------------------------------------------------------------

E(RTPSSh) = -1986.89402460 Hartree
Zero-point correction= 0.642679 (Hartree/Particle)
Thermal correction to Energy= 0.681672
Thermal correction to Enthalpy= 0.682616
Thermal correction to Gibbs Free Energy= 0.570738
Sum of electronic and zero-point Energies= -1986.251346
Sum of electronic and thermal Energies= -1986.212353
Sum of electronic and thermal Enthalpies= -1986.211409
Sum of electronic and thermal Free Energies= -1986.323287

**Table S37**: Cartesian coordinates (Å) of the Δ(λδδ)(λδδ) conformer of **[Pb(macropam)]$^{2+}$** from geometry optimizations (0 imaginary frequencies).

| Center Number | Atomic Number | Coordinates (Angstroms) X | Y | Z |
|---|---|---|---|---|
| 1 | 6 | 3.783868 | -1.429875 | 0.089630 |
| 2 | 7 | 2.819444 | -0.928839 | 1.097208 |
| 3 | 8 | 2.119370 | -1.735259 | -1.584853 |
| 4 | 6 | 3.407430 | -1.165319 | -1.356885 |
| 5 | 6 | 2.840252 | -1.813027 | 2.286470 |
| 6 | 6 | 1.567515 | -1.784129 | 3.104121 |
| 7 | 8 | 0.526584 | -2.361791 | 2.307509 |
| 8 | 6 | -0.610178 | -2.824733 | 3.044426 |
| 9 | 6 | -1.774392 | -1.863648 | 2.964813 |
| 10 | 8 | -2.119236 | -1.736797 | 1.583313 |
| 11 | 8 | -0.526386 | -2.360193 | -2.309412 |
| 12 | 6 | 0.610451 | -2.822347 | -3.046698 |
| 13 | 6 | 1.774505 | -1.861116 | -2.966411 |
| 14 | 7 | -2.819417 | -0.928083 | -1.098055 |
| 15 | 6 | -2.840025 | -1.811357 | -2.288021 |
| 16 | 6 | -1.567219 | -1.781673 | -3.105531 |
| 17 | 6 | -3.407387 | -1.166776 | 1.355851 |
| 18 | 6 | -3.783809 | -1.430044 | -0.090897 |
| 19 | 6 | 3.135447 | 0.441634 | 1.523235 |
| 20 | 6 | -3.135626 | 0.442646 | -1.523010 |
| 21 | 6 | -2.919856 | 1.494672 | -0.464632 |
| 22 | 6 | 2.919800 | 1.494397 | 0.465586 |
| 23 | 6 | -1.644614 | 2.319149 | 1.276009 |
| 24 | 6 | -2.497059 | 3.401324 | 1.462373 |
| 25 | 6 | -3.603354 | 3.521367 | 0.628743 |
| 26 | 6 | -3.823412 | 2.552840 | -0.336397 |
| 27 | 6 | 3.823654 | 2.552337 | 0.337798 |
| 28 | 6 | 3.603669 | 3.521538 | -0.626700 |
| 29 | 6 | 2.497184 | 3.402335 | -1.460169 |
| 30 | 6 | 1.644423 | 2.320304 | -1.274290 |
| 31 | 82 | -0.000045 | -0.591094 | 0.000047 |
| 32 | 1 | 3.843325 | -2.511612 | 0.207779 |
| 33 | 1 | 4.789237 | -1.025585 | 0.272878 |
| 34 | 1 | 4.146285 | -1.666820 | -1.993079 |
| 35 | 1 | 3.405898 | -0.104677 | -1.617908 |
| 36 | 1 | -0.339701 | -2.996755 | 4.090148 |
| 37 | 1 | -0.901053 | -3.777491 | 2.596850 |
| 38 | 1 | -1.523982 | -0.883790 | 3.383983 |
| 39 | 1 | -2.621690 | -2.281142 | 3.521314 |
| 40 | 1 | 0.339977 | -2.993644 | -4.092543 |
| 41 | 1 | 0.901518 | -3.775378 | -2.599831 |
| 42 | 1 | 2.621833 | -2.278066 | -3.523284 |
| 43 | 1 | 1.523914 | -0.880985 | -3.384844 |
| 44 | 1 | -3.405941 | -0.106394 | 1.617924 |
| 45 | 1 | -4.146182 | -1.668985 | 1.991559 |
| 46 | 1 | -3.843241 | -2.511679 | -0.209995 |
| 47 | 1 | -4.789190 | -1.025625 | -0.273766 |
| 48 | 1 | 4.175354 | 0.509051 | 1.873126 |
| 49 | 1 | 2.487963 | 0.684370 | 2.368469 |

| | | | | |
|---|---|---|---|---|
| 50 | 1 | -4.175628 | 0.510261 | -1.872591 |
| 51 | 1 | -2.488416 | 0.686088 | -2.368257 |
| 52 | 1 | -2.337168 | 4.123940 | 2.250502 |
| 53 | 1 | -4.293231 | 4.346275 | 0.750523 |
| 54 | 1 | -4.691838 | 2.596867 | -0.980761 |
| 55 | 1 | 4.692270 | 2.595673 | 0.981953 |
| 56 | 1 | 4.293782 | 4.346300 | -0.748134 |
| 57 | 1 | 2.337379 | 4.125454 | -2.247852 |
| 58 | 7 | 1.844174 | 1.398933 | -0.321123 |
| 59 | 7 | -1.844465 | 1.398404 | 0.322237 |
| 60 | 6 | -0.467142 | 2.029370 | 2.161256 |
| 61 | 8 | 0.085390 | 0.918192 | 2.117399 |
| 62 | 6 | 0.466778 | 2.031422 | -2.159672 |
| 63 | 8 | -0.085745 | 0.920255 | -2.116893 |
| 64 | 1 | -1.720740 | -2.393205 | -4.001011 |
| 65 | 1 | -1.290301 | -0.771144 | -3.418654 |
| 66 | 1 | -3.689613 | -1.565936 | -2.941894 |
| 67 | 1 | -2.978918 | -2.834701 | -1.940394 |
| 68 | 1 | 1.721102 | -2.396570 | 3.998967 |
| 69 | 1 | 1.290657 | -0.773914 | 3.418316 |
| 70 | 1 | 2.979210 | -2.836084 | 1.938025 |
| 71 | 1 | 3.689868 | -1.568050 | 2.940480 |
| 72 | 7 | -0.058569 | 2.983718 | 3.000277 |
| 73 | 1 | -0.458179 | 3.906773 | 3.014899 |
| 74 | 1 | 0.717511 | 2.797320 | 3.616769 |
| 75 | 7 | 0.058346 | 2.986671 | -2.997799 |
| 76 | 1 | -0.717866 | 2.801064 | -3.614357 |
| 77 | 1 | 0.457605 | 3.909900 | -3.010897 |

---------------------------------------------------------------------

E(RTPSSh) = -1986.88629864 Hartree

Zero-point correction= 0.643471 (Hartree/Particle)

Thermal correction to Energy= 0.682137

Thermal correction to Enthalpy= 0.683082

Thermal correction to Gibbs Free Energy= 0.572442

Sum of electronic and zero-point Energies= -1986.242828

Sum of electronic and thermal Energies= -1986.204161

Sum of electronic and thermal Enthalpies= -1986.203217

Sum of electronic and thermal Free Energies= -1986.313856

**Table S38**: Cartesian coordinates (Å) of the Δ(δλδ)(δλδ) conformer of **[Pb(macropam)]$^{2+}$** from geometry optimizations (0 imaginary frequencies).

| Center Number | Atomic Number | X | Y | Z |
|---|---|---|---|---|
| 1 | 6 | 3.715410 | -1.122884 | -0.478165 |
| 2 | 7 | 2.913242 | -0.761543 | 0.708852 |
| 3 | 8 | 1.894826 | -1.887319 | -1.825242 |
| 4 | 6 | 3.144369 | -2.283181 | -1.262095 |
| 5 | 6 | 3.168250 | -1.731053 | 1.791918 |
| 6 | 6 | 2.118856 | -1.735488 | 2.879911 |
| 7 | 8 | 0.885853 | -2.190777 | 2.323842 |
| 8 | 6 | -0.107921 | -2.368135 | 3.331152 |
| 9 | 6 | -1.364608 | -2.890901 | 2.690443 |
| 10 | 8 | -1.894760 | -1.887724 | 1.824985 |
| 11 | 8 | -0.885718 | -2.190389 | -2.324249 |
| 12 | 6 | 0.108069 | -2.367264 | -3.331633 |
| 13 | 6 | 1.364799 | -2.890186 | -2.691130 |
| 14 | 7 | -2.913167 | -0.761482 | -0.708922 |
| 15 | 6 | -3.168112 | -1.730863 | -1.792119 |
| 16 | 6 | -2.118765 | -1.735044 | -2.880156 |
| 17 | 6 | -3.144311 | -2.283464 | 1.261759 |
| 18 | 6 | -3.715332 | -1.123025 | 0.478039 |
| 19 | 6 | 3.267148 | 0.590637 | 1.156141 |
| 20 | 6 | -3.267175 | 0.590742 | -1.156012 |
| 21 | 6 | -2.878432 | 1.658183 | -0.163163 |
| 22 | 6 | 2.878380 | 1.658189 | 0.163409 |
| 23 | 6 | -1.443795 | 2.382971 | 1.497638 |
| 24 | 6 | -2.160826 | 3.559152 | 1.679526 |
| 25 | 6 | -3.281837 | 3.779360 | 0.886212 |
| 26 | 6 | -3.655797 | 2.811829 | -0.030702 |
| 27 | 6 | 3.655716 | 2.811870 | 0.031106 |
| 28 | 6 | 3.281778 | 3.779469 | -0.885748 |
| 29 | 6 | 2.160827 | 3.559295 | -1.679150 |
| 30 | 6 | 1.443769 | 2.383100 | -1.497376 |
| 31 | 82 | -0.000073 | -0.501149 | 0.000067 |
| 32 | 1 | 4.345223 | 0.675879 | 1.356472 |
| 33 | 1 | 2.745209 | 0.788113 | 2.094110 |
| 34 | 1 | -4.345267 | 0.675946 | -1.356279 |
| 35 | 1 | -2.745292 | 0.788404 | -2.093969 |
| 36 | 1 | -1.876773 | 4.296076 | 2.417477 |
| 37 | 1 | -3.863438 | 4.684651 | 1.002305 |
| 38 | 1 | -4.543981 | 2.933255 | -0.637324 |
| 39 | 1 | 4.543846 | 2.933264 | 0.637814 |
| 40 | 1 | 3.863361 | 4.684786 | -1.001733 |
| 41 | 1 | 1.876884 | 4.296245 | -2.417116 |
| 42 | 7 | 1.782512 | 1.469576 | -0.575834 |
| 43 | 7 | -1.782563 | 1.469504 | 0.576048 |
| 44 | 6 | -0.254651 | 1.989893 | 2.326737 |
| 45 | 8 | 0.336161 | 0.919776 | 2.106744 |
| 46 | 6 | 0.254633 | 1.990128 | -2.326563 |
| 47 | 8 | -0.336380 | 0.920131 | -2.106588 |
| 48 | 1 | 4.748326 | -1.364342 | -0.187748 |
| 49 | 1 | 3.842833 | -2.540461 | -2.067430 |

| | | | | |
|---|---|---|---|---|
| 50 | 1 | 3.004179 | -3.177378 | -0.643704 |
| 51 | 1 | -4.748256 | -1.364398 | 0.187584 |
| 52 | 1 | -3.756664 | -0.258268 | 1.140808 |
| 53 | 1 | -3.842782 | -2.540878 | 2.067045 |
| 54 | 1 | -3.004131 | -3.177559 | 0.643218 |
| 55 | 1 | -1.976227 | -0.746226 | -3.328658 |
| 56 | 1 | -2.439693 | -2.424512 | -3.670412 |
| 57 | 1 | -3.199521 | -2.727677 | -1.352499 |
| 58 | 1 | -4.151785 | -1.545568 | -2.249040 |
| 59 | 1 | 2.439766 | -2.425090 | 3.670056 |
| 60 | 1 | 1.976248 | -0.746765 | 3.328604 |
| 61 | 1 | 4.151889 | -1.545727 | 2.248895 |
| 62 | 1 | 3.199762 | -2.727805 | 1.352164 |
| 63 | 1 | 1.156277 | -3.804661 | -2.122875 |
| 64 | 1 | 2.095757 | -3.125867 | -3.473801 |
| 65 | 1 | 0.302937 | -1.413656 | -3.835842 |
| 66 | 1 | -0.244367 | -3.091498 | -4.076078 |
| 67 | 1 | 3.756770 | -0.258016 | -1.140789 |
| 68 | 1 | -2.095528 | -3.126998 | 3.473025 |
| 69 | 1 | -1.156005 | -3.805108 | 2.121787 |
| 70 | 1 | 0.244574 | -3.092643 | 4.075304 |
| 71 | 1 | -0.302864 | -1.414744 | 3.835744 |
| 72 | 7 | -0.133107 | 2.806614 | -3.308457 |
| 73 | 1 | -0.917526 | 2.539984 | -3.883525 |
| 74 | 1 | 0.340371 | 3.665592 | -3.530509 |
| 75 | 7 | 0.133345 | 2.806386 | 3.308502 |
| 76 | 1 | -0.340229 | 3.665262 | 3.530746 |
| 77 | 1 | 0.917772 | 2.539672 | 3.883524 |

---------------------------------------------------------------------

E(RTPSSh) = -1986.90386642 Hartree
Zero-point correction= 0.642552 (Hartree/Particle)
Thermal correction to Energy= 0.681447
Thermal correction to Enthalpy= 0.682391
Thermal correction to Gibbs Free Energy= 0.570914
Sum of electronic and zero-point Energies= -1986.261314
Sum of electronic and thermal Energies= -1986.222419
Sum of electronic and thermal Enthalpies= -1986.221475
Sum of electronic and thermal Free Energies= -1986.332953

**Table S39**: Cartesian coordinates (Å) of the Δ(λλλ)(λλλ) conformer of **[Bi(macropa)]$^+$** from geometry optimizations (0 imaginary frequencies).

| Center Number | Atomic Number | Coordinates (Angstroms) X | Y | Z |
|---|---|---|---|---|
| 1 | 6 | -3.511388 | 1.485409 | 0.570810 |
| 2 | 7 | -2.479132 | 0.792227 | 1.375093 |
| 3 | 8 | -2.352096 | 1.165319 | -1.549454 |
| 4 | 6 | -3.602925 | 1.015009 | -0.870179 |
| 5 | 6 | -2.195791 | 1.578867 | 2.600337 |
| 6 | 6 | -1.408809 | 2.848246 | 2.323980 |
| 7 | 8 | -0.086049 | 2.575741 | 1.846884 |
| 8 | 6 | 0.880201 | 2.465514 | 2.898061 |
| 9 | 6 | 2.247056 | 2.395483 | 2.275022 |
| 10 | 8 | 2.350503 | 1.167327 | 1.548845 |
| 11 | 8 | 0.083408 | 2.575635 | -1.847026 |
| 12 | 6 | -0.883063 | 2.465375 | -2.898025 |
| 13 | 6 | -2.249713 | 2.394049 | -2.274671 |
| 14 | 7 | 2.478121 | 0.794296 | -1.375312 |
| 15 | 6 | 2.193879 | 1.580526 | -2.600626 |
| 16 | 6 | 1.405865 | 2.849275 | -2.324321 |
| 17 | 6 | 3.601733 | 1.018505 | 0.869823 |
| 18 | 6 | 3.509661 | 1.488724 | -0.571176 |
| 19 | 6 | -2.922131 | -0.560261 | 1.761651 |
| 20 | 6 | 2.922481 | -0.557769 | -1.761804 |
| 21 | 6 | 2.755970 | -1.579848 | -0.666541 |
| 22 | 6 | -2.754719 | -1.582123 | 0.666340 |
| 23 | 6 | 1.627273 | -2.251693 | 1.252528 |
| 24 | 6 | 2.449603 | -3.356154 | 1.414018 |
| 25 | 6 | 3.451094 | -3.577317 | 0.475125 |
| 26 | 6 | 3.621399 | -2.667479 | -0.558997 |
| 27 | 6 | -3.619380 | -2.670339 | 0.558538 |
| 28 | 6 | -3.448184 | -3.580059 | -0.475542 |
| 29 | 6 | -2.446544 | -3.358220 | -1.414115 |
| 30 | 6 | -1.625034 | -2.253170 | -1.252390 |
| 31 | 83 | 0.000075 | 0.303538 | 0.000121 |
| 32 | 1 | -3.277004 | 2.548815 | 0.568334 |
| 33 | 1 | -4.496107 | 1.371973 | 1.045932 |
| 34 | 1 | -4.376944 | 1.594544 | -1.381486 |
| 35 | 1 | -3.879887 | -0.035435 | -0.943400 |
| 36 | 1 | 3.880011 | -0.031574 | 0.943157 |
| 37 | 1 | 4.374899 | 1.599047 | 1.381245 |
| 38 | 1 | 3.273989 | 2.551845 | -0.568683 |
| 39 | 1 | 4.494457 | 1.376438 | -1.046387 |
| 40 | 1 | -3.971340 | -0.555440 | 2.082341 |
| 41 | 1 | -2.315049 | -0.876293 | 2.609965 |
| 42 | 1 | 3.971723 | -0.551897 | -2.082372 |
| 43 | 1 | 2.315835 | -0.874371 | -2.610216 |
| 44 | 1 | 2.304444 | -4.001159 | 2.268643 |
| 45 | 1 | 4.112418 | -4.429567 | 0.566336 |
| 46 | 1 | 4.426318 | -2.780052 | -1.273673 |
| 47 | 1 | -4.424398 | -2.783460 | 1.273018 |
| 48 | 1 | -4.108924 | -4.432743 | -0.566949 |
| 49 | 1 | -2.300654 | -4.003161 | -2.268660 |

| | | | | |
|---|---|---|---|---|
| 50 | 7 | -1.756066 | -1.419902 | -0.210203 |
| 51 | 7 | 1.757447 | -1.418381 | 0.210265 |
| 52 | 6 | 0.591156 | -1.870938 | 2.284268 |
| 53 | 8 | -0.053545 | -0.779379 | 2.043294 |
| 54 | 8 | 0.446131 | -2.573438 | 3.283522 |
| 55 | 6 | -0.588829 | -1.871855 | -2.283796 |
| 56 | 8 | 0.054795 | -0.779616 | -2.042869 |
| 57 | 8 | -0.442550 | -2.574564 | -3.282715 |
| 58 | 1 | 0.690074 | 1.573310 | 3.502773 |
| 59 | 1 | 0.822616 | 3.351381 | 3.539191 |
| 60 | 1 | 3.007529 | 2.418978 | 3.063000 |
| 61 | 1 | 2.401441 | 3.250899 | 1.608794 |
| 62 | 1 | -0.692540 | 1.573605 | -3.503259 |
| 63 | 1 | -0.826157 | 3.351606 | -3.538698 |
| 64 | 1 | -2.404452 | 3.248902 | -1.607791 |
| 65 | 1 | -3.010405 | 2.417715 | -3.062454 |
| 66 | 1 | 1.877847 | 3.468694 | -1.561785 |
| 67 | 1 | 1.349716 | 3.439998 | -3.243350 |
| 68 | 1 | 3.129613 | 1.847962 | -3.111195 |
| 69 | 1 | 1.618756 | 0.942272 | -3.271388 |
| 70 | 1 | -1.353290 | 3.439149 | 3.242934 |
| 71 | 1 | -1.881222 | 3.467161 | 1.561302 |
| 72 | 1 | -3.131825 | 1.845570 | 3.110754 |
| 73 | 1 | -1.620248 | 0.941200 | 3.271308 |

---------------------------------------------------------------------

E(RTPSSh) = -2047.33553583 Hartree
Zero-point correction= 0.596306 (Hartree/Particle)
Thermal correction to Energy= 0.632488
Thermal correction to Enthalpy= 0.633432
Thermal correction to Gibbs Free Energy= 0.529473
Sum of electronic and zero-point Energies= -2046.739230
Sum of electronic and thermal Energies= -2046.703048
Sum of electronic and thermal Enthalpies= -2046.702103
Sum of electronic and thermal Free Energies= -2046.806063

**Table S40**: Cartesian coordinates (Å) of the Δ(δδδ)(δδδ) conformer of **[Bi(macropa)]**$^{+}$ from geometry optimizations (0 imaginary frequencies).

| Center Number | Atomic Number | Coordinates (Angstroms) X | Y | Z |
|---|---|---|---|---|
| 1 | 83 | 0.000360 | 0.000073 | -0.029210 |
| 2 | 8 | 0.882922 | -2.659200 | 0.863793 |
| 3 | 8 | 1.548276 | -0.550625 | 2.491207 |
| 4 | 8 | 0.091289 | -1.584363 | -1.633980 |
| 5 | 8 | 1.334562 | -3.056545 | -2.763399 |
| 6 | 7 | 2.031067 | 1.946864 | 0.882241 |
| 7 | 7 | 2.203422 | -0.021082 | -1.177230 |
| 8 | 6 | 4.628093 | -0.406800 | -2.429433 |
| 9 | 1 | 5.587669 | -0.558904 | -2.907039 |
| 10 | 6 | 2.798425 | 2.189843 | -0.347500 |
| 11 | 1 | 3.711723 | 2.762272 | -0.142624 |
| 12 | 1 | 2.179610 | 2.783850 | -1.023668 |
| 13 | 6 | -0.167314 | -3.611250 | 0.644837 |
| 14 | 1 | 0.153214 | -4.598952 | 0.990364 |
| 15 | 1 | -0.311182 | -3.653680 | -0.433121 |
| 16 | 6 | 1.441387 | 3.213807 | 1.372084 |
| 17 | 1 | 2.172037 | 4.030086 | 1.286085 |
| 18 | 1 | 1.229069 | 3.093752 | 2.434476 |
| 19 | 6 | 3.614336 | -1.347355 | -2.567712 |
| 20 | 1 | 3.733176 | -2.242839 | -3.160714 |
| 21 | 6 | 1.213023 | -2.015501 | -2.125454 |
| 22 | 6 | 2.404950 | -1.109394 | -1.930804 |
| 23 | 6 | 2.927804 | 1.383247 | 1.918082 |
| 24 | 1 | 3.527385 | 2.181177 | 2.376808 |
| 25 | 1 | 3.627624 | 0.702426 | 1.430370 |
| 26 | 6 | 3.153900 | 0.915988 | -1.069101 |
| 27 | 6 | 1.586010 | -2.878016 | 2.090081 |
| 28 | 1 | 2.219309 | -3.767063 | 1.997939 |
| 29 | 1 | 0.881997 | -3.031630 | 2.913354 |
| 30 | 6 | 2.433201 | -1.665781 | 2.368753 |
| 31 | 1 | 2.983042 | -1.812320 | 3.304689 |
| 32 | 1 | 3.155717 | -1.507535 | 1.561027 |
| 33 | 6 | 4.390736 | 0.742932 | -1.686656 |
| 34 | 1 | 5.150353 | 1.506635 | -1.583307 |
| 35 | 6 | 2.170720 | 0.631464 | 2.997384 |
| 36 | 1 | 2.854726 | 0.371140 | 3.811913 |
| 37 | 1 | 1.361184 | 1.232713 | 3.411400 |
| 38 | 8 | -0.884633 | 2.657391 | 0.866834 |
| 39 | 8 | -1.551406 | 0.547193 | 2.491391 |
| 40 | 8 | -0.089310 | 1.586413 | -1.632141 |
| 41 | 8 | -1.331435 | 3.059930 | -2.761067 |
| 42 | 7 | -2.032217 | -1.948094 | 0.878520 |
| 43 | 7 | -2.201931 | 0.022855 | -1.178836 |
| 44 | 6 | -4.625541 | 0.410449 | -2.432576 |
| 45 | 1 | -5.584704 | 0.563262 | -2.910785 |
| 46 | 6 | -2.798034 | -2.189185 | -0.352537 |
| 47 | 1 | -3.711609 | -2.761851 | -0.149579 |
| 48 | 1 | -2.178445 | -2.782268 | -1.028810 |
| 49 | 6 | 0.165694 | 3.609690 | 0.649354 |

| | | | | |
|---|---|---|---|---|
| 50 | 1 | -0.155139 | 4.597032 | 0.995625 |
| 51 | 1 | 0.310142 | 3.653158 | -0.428488 |
| 52 | 6 | -1.443338 | -3.215857 | 1.367216 |
| 53 | 1 | -2.174185 | -4.031794 | 1.279685 |
| 54 | 1 | -1.231725 | -3.097184 | 2.429903 |
| 55 | 6 | -3.611610 | 1.351115 | -2.568719 |
| 56 | 1 | -3.729894 | 2.247424 | -3.160586 |
| 57 | 6 | -1.210708 | 2.018608 | -2.123424 |
| 58 | 6 | -2.402784 | 1.112206 | -1.931108 |
| 59 | 6 | -2.930203 | -1.385908 | 1.914074 |
| 60 | 1 | -3.530312 | -2.184475 | 2.370996 |
| 61 | 1 | -3.629458 | -0.704439 | 1.426458 |
| 62 | 6 | -3.152609 | -0.914272 | -1.072729 |
| 63 | 6 | -1.588542 | 2.875033 | 2.092864 |
| 64 | 1 | -2.221672 | 3.764252 | 2.001215 |
| 65 | 1 | -0.885105 | 3.027678 | 2.916810 |
| 66 | 6 | -2.436105 | 1.662610 | 2.369582 |
| 67 | 1 | -2.986629 | 1.808141 | 3.305271 |
| 68 | 1 | -3.158031 | 1.505386 | 1.561131 |
| 69 | 6 | -4.388891 | -0.740283 | -1.691135 |
| 70 | 1 | -5.148661 | -1.504059 | -1.589455 |
| 71 | 6 | -2.174421 | -0.635573 | 2.995291 |
| 72 | 1 | -2.859368 | -0.376350 | 3.809379 |
| 73 | 1 | -1.365349 | -1.237366 | 3.409423 |

---------------------------------------------------------------------

E(RTPSSh) = -2047.33915097 Hartree
Zero-point correction= 0.596319 (Hartree/Particle)
Thermal correction to Energy= 0.632384
Thermal correction to Enthalpy= 0.633328
Thermal correction to Gibbs Free Energy= 0.529979
Sum of electronic and zero-point Energies= -2046.742832
Sum of electronic and thermal Energies= -2046.706767
Sum of electronic and thermal Enthalpies= -2046.705823
Sum of electronic and thermal Free Energies= -2046.809172

**Table S41**: Cartesian coordinates (Å) of the $\Delta(\delta\lambda\lambda)(\delta\lambda\lambda)$ conformer of **[Bi(macropa)]$^+$** from geometry optimizations (0 imaginary frequencies).

| Center Number | Atomic Number | Coordinates (Angstroms) X | Y | Z |
|---|---|---|---|---|
| 1 | 6 | 0.880170 | -3.649466 | 0.372962 |
| 2 | 7 | 0.535387 | -2.773512 | -0.773327 |
| 3 | 8 | 1.734404 | -1.833095 | 1.693187 |
| 4 | 6 | 2.002569 | -3.169329 | 1.268595 |
| 5 | 6 | 1.434299 | -2.936484 | -1.936991 |
| 6 | 6 | 2.761476 | -2.203672 | -1.820644 |
| 7 | 8 | 2.600861 | -0.791075 | -1.659834 |
| 8 | 6 | 2.508775 | -0.069654 | -2.895043 |
| 9 | 6 | 2.736521 | 1.385366 | -2.589162 |
| 10 | 8 | 1.716139 | 1.846358 | -1.696049 |
| 11 | 8 | 2.598449 | 0.811252 | 1.656415 |
| 12 | 6 | 2.515081 | 0.088235 | 2.891255 |
| 13 | 6 | 2.753523 | -1.364713 | 2.583827 |
| 14 | 7 | 0.515576 | 2.776987 | 0.773688 |
| 15 | 6 | 1.414090 | 2.946251 | 1.936747 |
| 16 | 6 | 2.747310 | 2.224991 | 1.818284 |
| 17 | 6 | 1.976674 | 3.183826 | -1.270493 |
| 18 | 6 | 0.852877 | 3.656089 | -0.372414 |
| 19 | 6 | -0.842873 | -3.103974 | -1.188178 |
| 20 | 6 | -0.864777 | 3.097051 | 1.189721 |
| 21 | 6 | -1.861855 | 2.620272 | 0.167179 |
| 22 | 6 | -1.842841 | -2.633894 | -0.165310 |
| 23 | 6 | -2.329003 | 1.130172 | -1.554639 |
| 24 | 6 | -3.536602 | 1.750220 | -1.835119 |
| 25 | 6 | -3.916509 | 2.839035 | -1.057897 |
| 26 | 6 | -3.058265 | 3.295331 | -0.066743 |
| 27 | 6 | -3.034773 | -3.316763 | 0.068822 |
| 28 | 6 | -3.895739 | -2.866176 | 1.060244 |
| 29 | 6 | -3.522717 | -1.775035 | 1.837563 |
| 30 | 6 | -2.319208 | -1.147169 | 1.556914 |
| 31 | 83 | 0.345970 | 0.001081 | 0.000006 |
| 32 | 1 | -0.968004 | -4.184203 | -1.334672 |
| 33 | 1 | -1.039209 | -2.611239 | -2.139700 |
| 34 | 1 | -0.997741 | 4.176277 | 1.336720 |
| 35 | 1 | -1.056965 | 2.602468 | 2.141139 |
| 36 | 1 | -4.137762 | 1.388323 | -2.656942 |
| 37 | 1 | -4.852768 | 3.348885 | -1.246230 |
| 38 | 1 | -3.295448 | 4.176627 | 0.514921 |
| 39 | 1 | -3.266438 | -4.199430 | -0.512982 |
| 40 | 1 | -4.828640 | -3.382097 | 1.248717 |
| 41 | 1 | -4.126018 | -1.417168 | 2.659587 |
| 42 | 7 | -1.530523 | -1.545344 | 0.548376 |
| 43 | 7 | -1.542628 | 1.533651 | -0.546403 |
| 44 | 6 | -1.773631 | 0.024407 | -2.420426 |
| 45 | 8 | -0.592481 | -0.383063 | -2.095707 |
| 46 | 8 | -2.429986 | -0.389389 | -3.375020 |
| 47 | 6 | -1.770600 | -0.038083 | 2.422795 |
| 48 | 8 | -0.592483 | 0.377370 | 2.097220 |
| 49 | 8 | -2.428759 | 0.370646 | 3.378330 |

| | | | | |
|---|---|---|---|---|
| 50 | 1 | 1.127753 | -4.658620 | 0.017605 |
| 51 | 1 | 2.039955 | -3.829818 | 2.142917 |
| 52 | 1 | 2.983278 | -3.210538 | 0.787524 |
| 53 | 1 | 1.094659 | 4.666493 | -0.016614 |
| 54 | 1 | -0.043182 | 3.736217 | -0.988093 |
| 55 | 1 | 2.008459 | 3.845420 | -2.144194 |
| 56 | 1 | 2.957930 | 3.230562 | -0.791067 |
| 57 | 1 | 3.733248 | -1.495529 | 2.111229 |
| 58 | 1 | 2.727263 | -1.948012 | 3.510914 |
| 59 | 1 | 1.536380 | 0.235756 | 3.354924 |
| 60 | 1 | 3.295751 | 0.437101 | 3.574844 |
| 61 | 1 | -0.014279 | -3.734715 | 0.990291 |
| 62 | 1 | 2.703282 | 1.967942 | -3.516483 |
| 63 | 1 | 3.716405 | 1.524095 | -2.119176 |
| 64 | 1 | 3.290498 | -0.412797 | -3.580321 |
| 65 | 1 | 1.530122 | -0.225280 | -3.356120 |
| 66 | 1 | 1.637771 | -4.000965 | -2.119977 |
| 67 | 1 | 0.901798 | -2.548028 | -2.805345 |
| 68 | 1 | 3.347927 | -2.405832 | -2.722501 |
| 69 | 1 | 3.345705 | -2.537319 | -0.966891 |
| 70 | 1 | 3.327696 | 2.564397 | 0.964164 |
| 71 | 1 | 3.332984 | 2.431349 | 2.719703 |
| 72 | 1 | 0.885638 | 2.552261 | 2.805078 |
| 73 | 1 | 1.608781 | 4.012178 | 2.120796 |

---------------------------------------------------------------------

E(RTPSSh) = -2047.33395176 Hartree
Zero-point correction= 0.596515 (Hartree/Particle)
Thermal correction to Energy= 0.632788
Thermal correction to Enthalpy= 0.633733
Thermal correction to Gibbs Free Energy= 0.529484
Sum of electronic and zero-point Energies= -2046.737437
Sum of electronic and thermal Energies= -2046.701163
Sum of electronic and thermal Enthalpies= -2046.700219
Sum of electronic and thermal Free Energies= -2046.804468

**Table S42**: Cartesian coordinates (Å) of the Δ(λδλ)(λδλ) conformer of **[Bi(macropa)]$^+$** from geometry optimizations (0 imaginary frequencies).

| Center Number | Atomic Number | Coordinates (Angstroms) X | Y | Z |
|---|---|---|---|---|
| 1 | 6 | 2.327927 | -2.208900 | -1.954846 |
| 2 | 7 | 1.684023 | -2.321621 | -0.629186 |
| 3 | 8 | 2.330602 | 0.171028 | -2.050125 |
| 4 | 6 | 3.175185 | -0.972260 | -2.127555 |
| 5 | 6 | 0.885708 | -3.571278 | -0.564667 |
| 6 | 6 | -0.245707 | -3.649070 | -1.561410 |
| 7 | 8 | -1.136371 | -2.552823 | -1.367702 |
| 8 | 6 | -2.112340 | -2.510663 | -2.407192 |
| 9 | 6 | -3.072975 | -1.386941 | -2.135827 |
| 10 | 8 | -2.330767 | -0.171252 | -2.049918 |
| 11 | 8 | 1.136374 | 2.552863 | -1.368528 |
| 12 | 6 | 2.112376 | 2.510421 | -2.407949 |
| 13 | 6 | 3.072886 | 1.386650 | -2.136287 |
| 14 | 7 | -1.683844 | 2.321737 | -0.629352 |
| 15 | 6 | -0.885567 | 3.571438 | -0.565593 |
| 16 | 6 | 0.245514 | 3.648842 | -1.562728 |
| 17 | 6 | -3.175466 | 0.971971 | -2.127007 |
| 18 | 6 | -2.328286 | 2.208730 | -1.954712 |
| 19 | 6 | 2.688520 | -2.391332 | 0.443562 |
| 20 | 6 | -2.687790 | 2.391749 | 0.443865 |
| 21 | 6 | -3.120323 | 1.069761 | 1.022544 |
| 22 | 6 | 3.120657 | -1.069287 | 1.022415 |
| 23 | 6 | -2.505513 | -1.021757 | 1.822267 |
| 24 | 6 | -3.756132 | -1.242658 | 2.376629 |
| 25 | 6 | -4.726339 | -0.258461 | 2.224259 |
| 26 | 6 | -4.400846 | 0.912249 | 1.555200 |
| 27 | 6 | 4.401099 | -0.911626 | 1.555206 |
| 28 | 6 | 4.726274 | 0.258972 | 2.224627 |
| 29 | 6 | 3.755800 | 1.242853 | 2.377271 |
| 30 | 6 | 2.505290 | 1.021868 | 1.822669 |
| 31 | 83 | 0.000110 | -0.000046 | 0.024916 |
| 32 | 1 | 1.545629 | -2.190074 | -2.712946 |
| 33 | 1 | 2.959838 | -3.089387 | -2.142494 |
| 34 | 1 | 3.656883 | -1.009660 | -3.111741 |
| 35 | 1 | 3.970033 | -0.907065 | -1.375210 |
| 36 | 1 | 0.478286 | -3.636762 | 0.442663 |
| 37 | 1 | 1.534909 | -4.443224 | -0.730622 |
| 38 | 1 | -0.779997 | -4.593259 | -1.400176 |
| 39 | 1 | 0.113673 | -3.650067 | -2.596331 |
| 40 | 1 | -2.664035 | -3.457637 | -2.444415 |
| 41 | 1 | -1.611648 | -2.360407 | -3.371120 |
| 42 | 1 | -3.625178 | -1.553727 | -1.203848 |
| 43 | 1 | -3.793007 | -1.330375 | -2.960099 |
| 44 | 1 | 2.664175 | 3.457333 | -2.445309 |
| 45 | 1 | 1.611735 | 2.360066 | -3.371892 |
| 46 | 1 | 3.792945 | 1.329812 | -2.960517 |
| 47 | 1 | 3.625072 | 1.553604 | -1.204326 |
| 48 | 1 | -0.477744 | 3.637287 | 0.441555 |
| 49 | 1 | -1.534838 | 4.443317 | -0.731613 |

| | | | | |
|---|---|---|---|---|
| 50 | 1 | -0.114126 | 3.649125 | -2.597565 |
| 51 | 1 | 0.779650 | 4.593246 | -1.402215 |
| 52 | 1 | -3.970005 | 0.906707 | -1.374338 |
| 53 | 1 | -3.657571 | 1.009307 | -3.110998 |
| 54 | 1 | -1.546291 | 2.189988 | -2.713123 |
| 55 | 1 | -2.960427 | 3.089096 | -2.142182 |
| 56 | 1 | 3.572823 | -2.957344 | 0.125833 |
| 57 | 1 | 2.239824 | -2.943819 | 1.273093 |
| 58 | 1 | -3.571951 | 2.958324 | 0.126725 |
| 59 | 1 | -2.238278 | 2.943696 | 1.273314 |
| 60 | 1 | -3.940816 | -2.159891 | 2.917040 |
| 61 | 1 | -5.718261 | -0.394872 | 2.635701 |
| 62 | 1 | -5.124160 | 1.709734 | 1.445441 |
| 63 | 1 | 5.124611 | -1.708913 | 1.445303 |
| 64 | 1 | 5.718129 | 0.395487 | 2.636195 |
| 65 | 1 | 3.940171 | 2.159936 | 2.918040 |
| 66 | 7 | 2.211931 | -0.094855 | 1.139031 |
| 67 | 7 | -2.211801 | 0.095135 | 1.139043 |
| 68 | 6 | -1.369001 | -1.988359 | 2.027930 |
| 69 | 8 | -0.217961 | -1.609639 | 1.555781 |
| 70 | 8 | -1.548756 | -3.030511 | 2.647207 |
| 71 | 6 | 1.368579 | 1.988184 | 2.028625 |
| 72 | 8 | 0.217850 | 1.610023 | 1.555360 |
| 73 | 8 | 1.547967 | 3.029681 | 2.649133 |

---------------------------------------------------------------------

E(RTPSSh) = -2047.33893116 Hartree
Zero-point correction= 0.595564 (Hartree/Particle)
Thermal correction to Energy= 0.632151
Thermal correction to Enthalpy= 0.633096
Thermal correction to Gibbs Free Energy= 0.527572
Sum of electronic and zero-point Energies= -2046.743367
Sum of electronic and thermal Energies= -2046.706780
Sum of electronic and thermal Enthalpies= -2046.705836
Sum of electronic and thermal Free Energies= -2046.811359

**Table S43**: Cartesian coordinates (Å) of the Δ(λλδ)(λλδ) conformer of **[Bi(macropa)]**$^{+}$ from geometry optimizations (0 imaginary frequencies).

| Center Number | Atomic Number | Coordinates (Angstroms) X | Y | Z |
|---|---|---|---|---|
| 1 | 6 | 0.898474 | -3.609010 | 0.598685 |
| 2 | 7 | 0.658405 | -2.785366 | -0.623257 |
| 3 | 8 | 2.476934 | -1.960919 | 1.287956 |
| 4 | 6 | 1.424218 | -2.795773 | 1.767141 |
| 5 | 6 | 1.762021 | -3.017432 | -1.585509 |
| 6 | 6 | 1.878972 | -1.974575 | -2.670828 |
| 7 | 8 | 2.276694 | -0.742082 | -2.064237 |
| 8 | 6 | 2.595683 | 0.257457 | -3.033979 |
| 9 | 6 | 3.275104 | 1.402651 | -2.316460 |
| 10 | 8 | 2.465844 | 1.974070 | -1.288384 |
| 11 | 8 | 2.272772 | 0.753926 | 2.063958 |
| 12 | 6 | 2.597977 | -0.243747 | 3.033563 |
| 13 | 6 | 3.283493 | -1.385076 | 2.315670 |
| 14 | 7 | 0.642966 | 2.788711 | 0.623362 |
| 15 | 6 | 1.745455 | 3.026481 | 1.585521 |
| 16 | 6 | 1.868121 | 1.984093 | 2.670685 |
| 17 | 6 | 1.408469 | 2.803232 | -1.767175 |
| 18 | 6 | 0.878638 | 3.613613 | -0.598579 |
| 19 | 6 | -0.646329 | -3.123298 | -1.216663 |
| 20 | 6 | -0.663477 | 3.119910 | 1.216806 |
| 21 | 6 | -1.767008 | 2.656377 | 0.301466 |
| 22 | 6 | -1.752349 | -2.665673 | -0.301332 |
| 23 | 6 | -2.371207 | 1.182008 | -1.396872 |
| 24 | 6 | -3.594867 | 1.806885 | -1.578511 |
| 25 | 6 | -3.909235 | 2.886603 | -0.759167 |
| 26 | 6 | -2.976555 | 3.333965 | 0.168436 |
| 27 | 6 | -2.958223 | -3.349799 | -0.168383 |
| 28 | 6 | -3.893384 | -2.907495 | 0.759137 |
| 29 | 6 | -3.584935 | -1.826085 | 1.578493 |
| 30 | 6 | -2.364679 | -1.194566 | 1.396921 |
| 31 | 83 | 0.354551 | 0.001027 | 0.000129 |
| 32 | 1 | 1.600401 | -4.410510 | 0.353393 |
| 33 | 1 | -0.033205 | -4.083075 | 0.910758 |
| 34 | 1 | 1.797896 | -3.472017 | 2.544238 |
| 35 | 1 | 0.624576 | -2.187804 | 2.206915 |
| 36 | 1 | 0.611993 | 2.191014 | -2.206793 |
| 37 | 1 | 1.778298 | 3.481569 | -2.544294 |
| 38 | 1 | 1.576374 | 4.418775 | -0.353315 |
| 39 | 1 | -0.055536 | 4.082825 | -0.910545 |
| 40 | 1 | -0.743737 | -4.202555 | -1.386876 |
| 41 | 1 | -0.734695 | -2.619650 | -2.179496 |
| 42 | 1 | -0.766538 | 4.198657 | 1.386934 |
| 43 | 1 | -0.749211 | 2.615850 | 2.179657 |
| 44 | 1 | -4.259440 | 1.459203 | -2.356533 |
| 45 | 1 | -4.856070 | 3.399787 | -0.868878 |
| 46 | 1 | -3.167389 | 4.211207 | 0.772716 |
| 47 | 1 | -3.144244 | -4.228068 | -0.772671 |
| 48 | 1 | -4.837435 | -3.425796 | 0.868778 |
| 49 | 1 | -4.251451 | -1.482021 | 2.356464 |

| | | | | |
|---|---|---|---|---|
| 50 | 7 | -1.504753 | -1.583954 | 0.447119 |
| 51 | 7 | -1.513484 | 1.576064 | -0.447024 |
| 52 | 6 | -1.861664 | 0.091716 | -2.308470 |
| 53 | 8 | -0.650031 | -0.298565 | -2.062762 |
| 54 | 8 | -2.564784 | -0.321724 | -3.225490 |
| 55 | 6 | -1.861176 | -0.101509 | 2.308547 |
| 56 | 8 | -0.651717 | 0.295433 | 2.062816 |
| 57 | 8 | -2.566527 | 0.308007 | 3.225612 |
| 58 | 1 | 2.642375 | 2.309186 | 3.374797 |
| 59 | 1 | 0.937204 | 1.834084 | 3.224712 |
| 60 | 1 | 1.628621 | 4.014088 | 2.051523 |
| 61 | 1 | 2.670953 | 3.031958 | 1.012121 |
| 62 | 1 | 2.655053 | -2.295473 | -3.374853 |
| 63 | 1 | 0.947252 | -1.829831 | -3.224903 |
| 64 | 1 | 2.687602 | -3.017925 | -1.012225 |
| 65 | 1 | 1.650391 | -4.005690 | -2.051399 |
| 66 | 1 | 1.685698 | 0.575144 | -3.554959 |
| 67 | 1 | 3.288796 | -0.155985 | -3.775845 |
| 68 | 1 | 3.560801 | 2.172408 | -3.042116 |
| 69 | 1 | 4.171542 | 1.040081 | -1.812718 |
| 70 | 1 | 1.690102 | -0.566617 | 3.555041 |
| 71 | 1 | 3.289143 | 0.173649 | 3.775028 |
| 72 | 1 | 4.177641 | -1.017467 | 1.811516 |
| 73 | 1 | 3.573831 | -2.153282 | 3.041127 |

---------------------------------------------------------------------

E(RTPSSh) = -2047.32319283 Hartree
Zero-point correction= 0.595744 (Hartree/Particle)
Thermal correction to Energy= 0.632204
Thermal correction to Enthalpy= 0.633148
Thermal correction to Gibbs Free Energy= 0.528170
Sum of electronic and zero-point Energies= -2046.727449
Sum of electronic and thermal Energies= -2046.690989
Sum of electronic and thermal Enthalpies= -2046.690045
Sum of electronic and thermal Free Energies= -2046.795023

**Table S44**: Cartesian coordinates (Å) of the Δ(δδλ)(δδλ) conformer of **[Bi(macropa)]**$^{+}$ from geometry optimizations (0 imaginary frequencies).

| Center Number | Atomic Number | Coordinates (Angstroms) X | Y | Z |
|---|---|---|---|---|
| 1 | 6 | 3.194789 | 1.971567 | -0.375491 |
| 2 | 7 | 2.327135 | 1.688081 | 0.788193 |
| 3 | 8 | 3.051728 | -0.308398 | -1.074239 |
| 4 | 6 | 3.958176 | 0.774629 | -0.897509 |
| 5 | 6 | 3.097146 | 1.233952 | 1.970128 |
| 6 | 6 | 3.227412 | -0.273551 | 2.091797 |
| 7 | 8 | 1.905465 | -0.808135 | 2.132445 |
| 8 | 6 | 1.790554 | -2.098246 | 2.725307 |
| 9 | 6 | 0.313899 | -2.325178 | 2.958951 |
| 10 | 8 | -0.443089 | -2.271096 | 1.739810 |
| 11 | 8 | 1.418465 | -2.297673 | -1.811809 |
| 12 | 6 | 2.802066 | -2.606742 | -1.660581 |
| 13 | 6 | 3.591979 | -1.343331 | -1.895893 |
| 14 | 7 | -1.386876 | -2.394166 | -1.035417 |
| 15 | 6 | -0.809376 | -2.948805 | -2.280572 |
| 16 | 6 | 0.618059 | -3.423825 | -2.151454 |
| 17 | 6 | -0.475413 | -3.514619 | 1.027482 |
| 18 | 6 | -1.586096 | -3.436588 | 0.001502 |
| 19 | 6 | 1.588630 | 2.918143 | 1.125586 |
| 20 | 6 | -2.679366 | -1.781771 | -1.386714 |
| 21 | 6 | -3.198343 | -0.881850 | -0.301170 |
| 22 | 6 | 0.604208 | 3.287196 | 0.048062 |
| 23 | 6 | -2.738647 | 0.619680 | 1.408794 |
| 24 | 6 | -4.083241 | 0.773430 | 1.709058 |
| 25 | 6 | -5.014234 | 0.065392 | 0.959381 |
| 26 | 6 | -4.564103 | -0.784634 | -0.040476 |
| 27 | 6 | 0.307973 | 4.616273 | -0.246325 |
| 28 | 6 | -0.583084 | 4.907714 | -1.270460 |
| 29 | 6 | -1.118179 | 3.865519 | -2.018746 |
| 30 | 6 | -0.770361 | 2.566526 | -1.680787 |
| 31 | 83 | 0.136535 | -0.126213 | 0.010084 |
| 32 | 1 | 2.571499 | 1.585263 | 2.858017 |
| 33 | 1 | 4.096465 | 1.687274 | 1.978057 |
| 34 | 1 | 3.741907 | -0.496612 | 3.032779 |
| 35 | 1 | 3.786846 | -0.729101 | 1.272944 |
| 36 | 1 | 2.314867 | -2.117026 | 3.687537 |
| 37 | 1 | 2.231787 | -2.863828 | 2.077193 |
| 38 | 1 | -0.081041 | -1.526055 | 3.585376 |
| 39 | 1 | 0.146162 | -3.286792 | 3.451947 |
| 40 | 1 | 3.107502 | -3.358841 | -2.396053 |
| 41 | 1 | 2.987330 | -3.005806 | -0.657168 |
| 42 | 1 | 4.641824 | -1.524374 | -1.639495 |
| 43 | 1 | 3.534549 | -1.036844 | -2.946370 |
| 44 | 1 | -0.837889 | -2.153293 | -3.024744 |
| 45 | 1 | -1.425254 | -3.784382 | -2.641640 |
| 46 | 1 | 0.730224 | -4.220247 | -1.407455 |
| 47 | 1 | 0.939417 | -3.829223 | -3.118208 |
| 48 | 1 | 2.268803 | 3.765293 | 1.284565 |
| 49 | 1 | 1.044775 | 2.738708 | 2.052599 |

| | | | | |
|---|---|---|---|---|
| 50 | 1 | -3.436600 | -2.543340 | -1.610225 |
| 51 | 1 | -2.528303 | -1.185434 | -2.287205 |
| 52 | 1 | -4.367638 | 1.422467 | 2.524718 |
| 53 | 1 | -6.072859 | 0.154530 | 1.167271 |
| 54 | 1 | -5.256479 | -1.383021 | -0.618027 |
| 55 | 1 | 0.787789 | 5.405656 | 0.317519 |
| 56 | 1 | -0.829704 | 5.935744 | -1.503762 |
| 57 | 1 | -1.774053 | 4.034535 | -2.860740 |
| 58 | 7 | 0.034419 | 2.293128 | -0.644498 |
| 59 | 7 | -2.314877 | -0.165015 | 0.406815 |
| 60 | 6 | -1.668477 | 1.280639 | 2.239120 |
| 61 | 8 | -0.454260 | 0.981064 | 1.913926 |
| 62 | 8 | -1.985254 | 2.016906 | 3.170378 |
| 63 | 6 | -1.198629 | 1.378580 | -2.508074 |
| 64 | 8 | -0.720902 | 0.244105 | -2.118999 |
| 65 | 8 | -1.915867 | 1.547414 | -3.492932 |
| 66 | 1 | 2.558155 | 2.351728 | -1.174729 |
| 67 | 1 | 3.920125 | 2.759143 | -0.124334 |
| 68 | 1 | 4.412881 | 1.052201 | -1.855091 |
| 69 | 1 | 4.766728 | 0.472881 | -0.221064 |
| 70 | 1 | -1.711212 | -4.420299 | -0.466897 |
| 71 | 1 | -2.509505 | -3.213858 | 0.538129 |
| 72 | 1 | -0.703205 | -4.322622 | 1.730137 |
| 73 | 1 | 0.498763 | -3.717028 | 0.577684 |

---------------------------------------------------------------------

E(RTPSSh) = -2047.33195754 Hartree
Zero-point correction= 0.595854 (Hartree/Particle)
Thermal correction to Energy= 0.632447
Thermal correction to Enthalpy= 0.633391
Thermal correction to Gibbs Free Energy= 0.528000
Sum of electronic and zero-point Energies= -2046.736103
Sum of electronic and thermal Energies= -2046.699511
Sum of electronic and thermal Enthalpies= -2046.698567
Sum of electronic and thermal Free Energies= -2046.803957

**Table S45**: Cartesian coordinates (Å) of the Δ(λδδ)(λδδ) conformer of **[Bi(macropa)]**$^{+}$ from geometry optimizations (0 imaginary frequencies).

| Center Number | Atomic Number | Coordinates (Angstroms) X | Y | Z |
|---|---|---|---|---|
| 1 | 6 | 3.756643 | 1.287106 | 0.188785 |
| 2 | 7 | 2.849134 | 0.860159 | -0.906544 |
| 3 | 8 | 1.933047 | 1.575104 | 1.681128 |
| 4 | 6 | 3.244215 | 1.013915 | 1.590588 |
| 5 | 6 | 2.982717 | 1.798359 | -2.049158 |
| 6 | 6 | 1.754429 | 1.877387 | -2.924379 |
| 7 | 8 | 0.700621 | 2.429394 | -2.125214 |
| 8 | 6 | -0.409150 | 2.924336 | -2.880002 |
| 9 | 6 | -1.508616 | 1.896549 | -3.012576 |
| 10 | 8 | -1.932206 | 1.575059 | -1.681555 |
| 11 | 8 | -0.699535 | 2.430442 | 2.124661 |
| 12 | 6 | 0.410424 | 2.925075 | 2.879346 |
| 13 | 6 | 1.509497 | 1.896898 | 3.012091 |
| 14 | 7 | -2.848620 | 0.861462 | 0.906459 |
| 15 | 6 | -2.981791 | 1.799954 | 2.048868 |
| 16 | 6 | -1.753401 | 1.878772 | 2.923962 |
| 17 | 6 | -3.243740 | 1.014750 | -1.590737 |
| 18 | 6 | -3.755887 | 1.288648 | -0.188953 |
| 19 | 6 | 3.158334 | -0.503319 | -1.363116 |
| 20 | 6 | -3.158441 | -0.501766 | 1.363354 |
| 21 | 6 | -2.778233 | -1.558012 | 0.360828 |
| 22 | 6 | 2.777673 | -1.559162 | -0.360320 |
| 23 | 6 | -1.353968 | -2.241315 | -1.344276 |
| 24 | 6 | -2.072117 | -3.409143 | -1.551522 |
| 25 | 6 | -3.182738 | -3.651416 | -0.752074 |
| 26 | 6 | -3.552453 | -2.704751 | 0.193535 |
| 27 | 6 | 3.551352 | -2.706232 | -0.192810 |
| 28 | 6 | 3.181120 | -3.652607 | 0.752891 |
| 29 | 6 | 2.070493 | -3.409771 | 1.552155 |
| 30 | 6 | 1.352920 | -2.241617 | 1.344732 |
| 31 | 83 | 0.000073 | 0.435001 | -0.000041 |
| 32 | 1 | 3.880099 | 2.365769 | 0.103575 |
| 33 | 1 | 4.749065 | 0.832172 | 0.073612 |
| 34 | 1 | 3.907691 | 1.524701 | 2.295783 |
| 35 | 1 | 3.225926 | -0.045148 | 1.854874 |
| 36 | 1 | -0.077187 | 3.251090 | -3.869988 |
| 37 | 1 | -0.788978 | 3.788856 | -2.332710 |
| 38 | 1 | -1.169479 | 0.994025 | -3.528497 |
| 39 | 1 | -2.346309 | 2.332320 | -3.566730 |
| 40 | 1 | 0.078608 | 3.252120 | 3.869288 |
| 41 | 1 | 0.790589 | 3.789358 | 2.331911 |
| 42 | 1 | 2.347328 | 2.332421 | 3.566236 |
| 43 | 1 | 1.170007 | 0.994548 | 3.528080 |
| 44 | 1 | -3.226186 | -0.044385 | -1.854781 |
| 45 | 1 | -3.906934 | 1.525802 | -2.296002 |
| 46 | 1 | -3.878622 | 2.367420 | -0.104060 |
| 47 | 1 | -4.748617 | 0.834421 | -0.073582 |
| 48 | 1 | 4.227761 | -0.605926 | -1.590293 |
| 49 | 1 | 2.600318 | -0.686405 | -2.282422 |

| | | | | |
|---|---|---|---|---|
| 50 | 1 | -4.227914 | -0.603867 | 1.590556 |
| 51 | 1 | -2.600494 | -0.684879 | 2.282700 |
| 52 | 1 | -1.764430 | -4.084652 | -2.336758 |
| 53 | 1 | -3.771438 | -4.550153 | -0.884875 |
| 54 | 1 | -4.441992 | -2.836503 | 0.795620 |
| 55 | 1 | 4.440860 | -2.838502 | -0.794827 |
| 56 | 1 | 3.769398 | -4.551597 | 0.885852 |
| 57 | 1 | 1.762359 | -4.085103 | 2.337368 |
| 58 | 7 | 1.679934 | -1.365780 | 0.383563 |
| 59 | 7 | -1.680435 | -1.365251 | -0.383124 |
| 60 | 6 | -0.214739 | -1.836549 | -2.247182 |
| 61 | 8 | 0.289915 | -0.671876 | -2.000356 |
| 62 | 8 | 0.134983 | -2.579163 | -3.161129 |
| 63 | 6 | 0.213630 | -1.836351 | 2.247346 |
| 64 | 8 | -0.290219 | -0.671320 | 2.000591 |
| 65 | 8 | -0.136705 | -2.578872 | 3.161143 |
| 66 | 1 | -1.966374 | 2.556356 | 3.757748 |
| 67 | 1 | -1.458575 | 0.906459 | 3.325354 |
| 68 | 1 | -3.851320 | 1.537608 | 2.666595 |
| 69 | 1 | -3.155815 | 2.794932 | 1.641184 |
| 70 | 1 | 1.967641 | 2.554833 | -3.758213 |
| 71 | 1 | 1.459372 | 0.905111 | -3.325684 |
| 72 | 1 | 3.157013 | 2.793371 | -1.641673 |
| 73 | 1 | 3.852218 | 1.535595 | -2.666742 |

---------------------------------------------------------------------

E(RTPSSh) = -2047.32399897 Hartree
Zero-point correction= 0.596164 (Hartree/Particle)
Thermal correction to Energy= 0.632493
Thermal correction to Enthalpy= 0.633437
Thermal correction to Gibbs Free Energy= 0.528338
Sum of electronic and zero-point Energies= -2046.727835
Sum of electronic and thermal Energies= -2046.691506
Sum of electronic and thermal Enthalpies= -2046.690562
Sum of electronic and thermal Free Energies= -2046.795661

**Table S46**: Cartesian coordinates (Å) of the Δ(δλδ)(δλδ) conformer of **[Bi(macropa)]**$^{+}$ from geometry optimizations (0 imaginary frequencies).

| Center Number | Atomic Number | Coordinates (Angstroms) X | Y | Z |
|---|---|---|---|---|
| 1 | 6 | 3.638360 | -0.999198 | -0.692896 |
| 2 | 7 | 2.894374 | -0.700314 | 0.551619 |
| 3 | 8 | 1.755265 | -1.842233 | -1.890259 |
| 4 | 6 | 3.073863 | -2.172191 | -1.456711 |
| 5 | 6 | 3.254184 | -1.699250 | 1.581019 |
| 6 | 6 | 2.283057 | -1.770868 | 2.732828 |
| 7 | 8 | 1.028964 | -2.230564 | 2.233692 |
| 8 | 6 | 0.092832 | -2.404103 | 3.293586 |
| 9 | 6 | -1.209574 | -2.877040 | 2.715152 |
| 10 | 8 | -1.753436 | -1.843394 | 1.890702 |
| 11 | 8 | -1.026696 | -2.232050 | -2.233255 |
| 12 | 6 | -0.090409 | -2.404872 | -3.293130 |
| 13 | 6 | 1.212443 | -2.876503 | -2.714636 |
| 14 | 7 | -2.893636 | -0.703043 | -0.551487 |
| 15 | 6 | -3.252384 | -1.702542 | -1.580690 |
| 16 | 6 | -2.281165 | -1.773487 | -2.732457 |
| 17 | 6 | -3.071674 | -2.174742 | 1.457126 |
| 18 | 6 | -3.637318 | -1.002455 | 0.693074 |
| 19 | 6 | 3.251881 | 0.649201 | 1.016392 |
| 20 | 6 | -3.252481 | 0.646009 | -1.016554 |
| 21 | 6 | -2.740205 | 1.699554 | -0.071836 |
| 22 | 6 | 2.738496 | 1.702010 | 0.071465 |
| 23 | 6 | -1.161600 | 2.302204 | 1.525831 |
| 24 | 6 | -1.814305 | 3.493381 | 1.801419 |
| 25 | 6 | -2.967058 | 3.794612 | 1.085453 |
| 26 | 6 | -3.447506 | 2.876928 | 0.161189 |
| 27 | 6 | 3.444524 | 2.880112 | -0.161776 |
| 28 | 6 | 2.963150 | 3.797019 | -1.086326 |
| 29 | 6 | 1.810780 | 3.494357 | -1.802301 |
| 30 | 6 | 1.159314 | 2.302567 | -1.526435 |
| 31 | 83 | 0.000137 | -0.349730 | 0.000124 |
| 32 | 1 | 4.340324 | 0.760840 | 1.110458 |
| 33 | 1 | 2.811742 | 0.804060 | 2.001299 |
| 34 | 1 | -4.341026 | 0.756586 | -1.110678 |
| 35 | 1 | -2.812465 | 0.801061 | -2.001494 |
| 36 | 1 | -1.423246 | 4.143491 | 2.570852 |
| 37 | 1 | -3.504007 | 4.716384 | 1.269433 |
| 38 | 1 | -4.374041 | 3.052939 | -0.369492 |
| 39 | 1 | 4.370811 | 3.057305 | 0.368946 |
| 40 | 1 | 3.499110 | 4.719326 | -1.270513 |
| 41 | 1 | 1.419107 | 4.143854 | -2.571942 |
| 42 | 7 | 1.594439 | 1.455795 | -0.582158 |
| 43 | 7 | -1.595836 | 1.454733 | 0.581783 |
| 44 | 6 | 0.032106 | 1.839775 | 2.323023 |
| 45 | 8 | 0.497027 | 0.680440 | 1.987406 |
| 46 | 8 | 0.463521 | 2.534769 | 3.239027 |
| 47 | 6 | -0.033900 | 1.838718 | -2.323527 |
| 48 | 8 | -0.497480 | 0.678912 | -1.987706 |
| 49 | 8 | -0.466141 | 2.533101 | -3.239603 |

| | | | | |
|---|---|---|---|---|
| 50 | 1 | 4.695797 | -1.192993 | -0.466866 |
| 51 | 1 | 3.708070 | -2.360820 | -2.330690 |
| 52 | 1 | 3.046968 | -3.090216 | -0.859351 |
| 53 | 1 | -4.694561 | -1.197346 | 0.467092 |
| 54 | 1 | -3.597359 | -0.124974 | 1.338745 |
| 55 | 1 | -3.705755 | -2.363797 | 2.331110 |
| 56 | 1 | -3.043850 | -3.092876 | 0.859975 |
| 57 | 1 | -2.146488 | -0.806455 | -3.226793 |
| 58 | 1 | -2.665422 | -2.487549 | -3.470914 |
| 59 | 1 | -3.282232 | -2.681367 | -1.103577 |
| 60 | 1 | -4.259362 | -1.496988 | -1.969705 |
| 61 | 1 | 2.667901 | -2.484515 | 3.471375 |
| 62 | 1 | 2.147560 | -0.803886 | 3.227025 |
| 63 | 1 | 4.260990 | -1.492593 | 1.969898 |
| 64 | 1 | 3.284987 | -2.678153 | 1.104135 |
| 65 | 1 | 1.069248 | -3.787459 | -2.121915 |
| 66 | 1 | 1.910296 | -3.095994 | -3.530620 |
| 67 | 1 | 0.047950 | -1.456802 | -3.824968 |
| 68 | 1 | -0.464257 | -3.154433 | -4.001281 |
| 69 | 1 | 3.597534 | -0.121876 | -1.338729 |
| 70 | 1 | -1.907176 | -3.097144 | 3.531190 |
| 71 | 1 | -1.065494 | -3.787920 | 2.122527 |
| 72 | 1 | 0.467355 | -3.153208 | 4.001860 |
| 73 | 1 | -0.046393 | -1.456070 | 3.825270 |

---------------------------------------------------------------------

E(RTPSSh) = -2047.34019306 Hartree
Zero-point correction= 0.596139 (Hartree/Particle)
Thermal correction to Energy= 0.632446
Thermal correction to Enthalpy= 0.633390
Thermal correction to Gibbs Free Energy= 0.529080
Sum of electronic and zero-point Energies= -2046.744054
Sum of electronic and thermal Energies= -2046.707747
Sum of electronic and thermal Enthalpies= -2046.706803
Sum of electronic and thermal Free Energies= -2046.811113

**Table S47**: Cartesian coordinates (Å) of the Δ(λλλ)(λλλ) conformer of **[Bi(macropapam)]$^{2+}$**from geometry optimizations (0 imaginary frequencies).

| Center Number | Atomic Number | Coordinates (Angstroms) X | Y | Z |
|---|---|---|---|---|
| 1 | 6 | 3.643315 | 1.126054 | -0.524509 |
| 2 | 7 | 2.562746 | 0.526922 | -1.341941 |
| 3 | 8 | 2.364300 | 1.007238 | 1.542156 |
| 4 | 6 | 3.624454 | 0.696243 | 0.929687 |
| 5 | 6 | 2.396687 | 1.314117 | -2.589202 |
| 6 | 6 | 1.725672 | 2.656451 | -2.353546 |
| 7 | 8 | 0.371587 | 2.512065 | -1.907773 |
| 8 | 6 | -0.569552 | 2.458014 | -2.986874 |
| 9 | 6 | -1.954893 | 2.488667 | -2.405020 |
| 10 | 8 | -2.155746 | 1.286273 | -1.653505 |
| 11 | 8 | 0.083650 | 2.593737 | 1.733046 |
| 12 | 6 | 1.009048 | 2.476632 | 2.825677 |
| 13 | 6 | 2.377102 | 2.255365 | 2.246808 |
| 14 | 7 | -2.414842 | 1.019958 | 1.272893 |
| 15 | 6 | -2.127330 | 1.855092 | 2.467289 |
| 16 | 6 | -1.222667 | 3.028564 | 2.144816 |
| 17 | 6 | -3.440909 | 1.225741 | -1.025282 |
| 18 | 6 | -3.380097 | 1.737121 | 0.402193 |
| 19 | 6 | 2.872629 | -0.874495 | -1.681163 |
| 20 | 6 | -2.954424 | -0.280494 | 1.712036 |
| 21 | 6 | -2.813928 | -1.373674 | 0.684534 |
| 22 | 6 | 2.548071 | -1.847737 | -0.575125 |
| 23 | 6 | -1.750910 | -2.192107 | -1.207243 |
| 24 | 6 | -2.595380 | -3.288857 | -1.274580 |
| 25 | 6 | -3.576640 | -3.428675 | -0.296886 |
| 26 | 6 | -3.708429 | -2.447010 | 0.668933 |
| 27 | 6 | 3.260723 | -3.037737 | -0.433785 |
| 28 | 6 | 2.958750 | -3.896059 | 0.613907 |
| 29 | 6 | 1.987026 | -3.524392 | 1.536854 |
| 30 | 6 | 1.320557 | -2.325317 | 1.342680 |
| 31 | 83 | 0.050034 | 0.387968 | 0.061535 |
| 32 | 1 | 3.533720 | 2.208521 | -0.563993 |
| 33 | 1 | 4.622512 | 0.882428 | -0.958696 |
| 34 | 1 | 4.428530 | 1.208303 | 1.463847 |
| 35 | 1 | 3.780921 | -0.374023 | 1.049702 |
| 36 | 1 | -3.775008 | 0.191332 | -1.081412 |
| 37 | 1 | -4.158618 | 1.832014 | -1.584743 |
| 38 | 1 | -3.089864 | 2.786109 | 0.377405 |
| 39 | 1 | -4.384031 | 1.689468 | 0.844231 |
| 40 | 1 | 3.930195 | -0.989902 | -1.947986 |
| 41 | 1 | 2.286724 | -1.144041 | -2.559193 |
| 42 | 1 | -4.013315 | -0.193679 | 1.983026 |
| 43 | 1 | -2.408801 | -0.585948 | 2.604638 |
| 44 | 1 | -2.512473 | -4.023800 | -2.062442 |
| 45 | 1 | -4.248404 | -4.276447 | -0.316871 |
| 46 | 1 | -4.500394 | -2.491852 | 1.404802 |
| 47 | 1 | 4.053369 | -3.269624 | -1.133056 |
| 48 | 1 | 3.500079 | -4.826190 | 0.730381 |
| 49 | 1 | 1.751710 | -4.124918 | 2.403704 |

| | | | | |
|---|---|---|---|---|
| 50 | 7 | 1.567438 | -1.540498 | 0.282849 |
| 51 | 7 | -1.827111 | -1.292622 | -0.211679 |
| 52 | 6 | -0.741046 | -1.832865 | -2.249472 |
| 53 | 8 | -0.000313 | -0.843727 | -2.073490 |
| 54 | 6 | 0.346249 | -1.783381 | 2.359229 |
| 55 | 8 | -0.142304 | -0.611874 | 2.092864 |
| 56 | 8 | 0.097095 | -2.428949 | 3.371794 |
| 57 | 1 | -0.423656 | 1.549618 | -3.579461 |
| 58 | 1 | -0.431830 | 3.331042 | -3.632933 |
| 59 | 1 | -2.689077 | 2.538380 | -3.215830 |
| 60 | 1 | -2.074994 | 3.367912 | -1.763803 |
| 61 | 1 | 0.724805 | 1.645612 | 3.476909 |
| 62 | 1 | 1.004853 | 3.406563 | 3.401234 |
| 63 | 1 | 2.641194 | 3.073303 | 1.569460 |
| 64 | 1 | 3.112851 | 2.208386 | 3.055448 |
| 65 | 1 | -1.606956 | 3.637509 | 1.327357 |
| 66 | 1 | -1.130625 | 3.669300 | 3.025051 |
| 67 | 1 | -3.060655 | 2.229403 | 2.907333 |
| 68 | 1 | -1.639919 | 1.218554 | 3.205142 |
| 69 | 1 | 1.747292 | 3.234171 | -3.281878 |
| 70 | 1 | 2.233399 | 3.242429 | -1.587702 |
| 71 | 1 | 3.370098 | 1.481169 | -3.069820 |
| 72 | 1 | 1.786782 | 0.724122 | -3.273247 |
| 73 | 7 | -0.679359 | -2.552679 | -3.362320 |
| 74 | 1 | -1.297711 | -3.324688 | -3.548170 |
| 75 | 1 | -0.028307 | -2.289111 | -4.086904 |

---------------------------------------------------------------------

E(RTPSSh) = -2027.90236630 Hartree
Zero-point correction= 0.622270 (Hartree/Particle)
Thermal correction to Energy= 0.658813
Thermal correction to Enthalpy= 0.659757
Thermal correction to Gibbs Free Energy= 0.555905
Sum of electronic and zero-point Energies= -2027.280097
Sum of electronic and thermal Energies= -2027.243553
Sum of electronic and thermal Enthalpies= -2027.242609
Sum of electronic and thermal Free Energies= -2027.346461

**Table S48**: Cartesian coordinates (Å) of the Δ(δδδ)(δδδ) conformer of **[Bi(macropapam)]$^{2+}$** from geometry optimizations (0 imaginary frequencies).

| Center Number | Atomic Number | Coordinates (Angstroms) X | Y | Z |
|---|---|---|---|---|
| 1 | 83 | -0.015324 | 0.073100 | 0.051498 |
| 2 | 8 | 0.893289 | -2.602352 | 1.037698 |
| 3 | 8 | 1.457331 | -0.374099 | 2.532325 |
| 4 | 8 | 0.172312 | -1.700406 | -1.532029 |
| 5 | 7 | 1.939468 | 2.013777 | 0.792070 |
| 6 | 7 | 2.235846 | -0.094097 | -1.109011 |
| 7 | 6 | 4.694342 | -0.470639 | -2.309483 |
| 8 | 1 | 5.662882 | -0.623796 | -2.766439 |
| 9 | 6 | 2.758202 | 2.181052 | -0.418924 |
| 10 | 1 | 3.654342 | 2.775861 | -0.208990 |
| 11 | 1 | 2.167169 | 2.721359 | -1.161587 |
| 12 | 6 | -0.147021 | -3.577914 | 0.893545 |
| 13 | 1 | 0.179554 | -4.532727 | 1.315826 |
| 14 | 1 | -0.291109 | -3.713720 | -0.176248 |
| 15 | 6 | 1.320105 | 3.311667 | 1.158487 |
| 16 | 1 | 2.050609 | 4.122201 | 1.041071 |
| 17 | 1 | 1.060591 | 3.268718 | 2.216047 |
| 18 | 6 | 3.705880 | -1.443269 | -2.421214 |
| 19 | 1 | 3.899460 | -2.342705 | -2.988066 |
| 20 | 6 | 1.286125 | -2.092681 | -1.954233 |
| 21 | 6 | 2.482851 | -1.210728 | -1.809552 |
| 22 | 6 | 2.808474 | 1.553735 | 1.906371 |
| 23 | 1 | 3.356664 | 2.405183 | 2.327883 |
| 24 | 1 | 3.552334 | 0.867648 | 1.498757 |
| 25 | 6 | 3.156995 | 0.871206 | -1.044437 |
| 26 | 6 | 1.590982 | -2.722208 | 2.281932 |
| 27 | 1 | 2.259148 | -3.589077 | 2.247884 |
| 28 | 1 | 0.883487 | -2.854805 | 3.105314 |
| 29 | 6 | 2.386141 | -1.463981 | 2.494946 |
| 30 | 1 | 2.922493 | -1.526531 | 3.446970 |
| 31 | 1 | 3.115505 | -1.327641 | 1.690232 |
| 32 | 6 | 4.412515 | 0.703655 | -1.631699 |
| 33 | 1 | 5.147556 | 1.493685 | -1.555881 |
| 34 | 6 | 2.028063 | 0.851577 | 3.000016 |
| 35 | 1 | 2.685983 | 0.652549 | 3.851456 |
| 36 | 1 | 1.188836 | 1.451860 | 3.351106 |
| 37 | 8 | -0.958383 | 2.663695 | 0.597513 |
| 38 | 8 | -1.606710 | 0.708181 | 2.354991 |
| 39 | 8 | -0.032705 | 1.443839 | -1.723229 |
| 40 | 8 | -1.232343 | 2.786820 | -3.042868 |
| 41 | 7 | -2.021190 | -1.911715 | 0.991522 |
| 42 | 7 | -2.153950 | -0.087580 | -1.204697 |
| 43 | 6 | -4.534411 | 0.170541 | -2.572699 |
| 44 | 1 | -5.477432 | 0.274444 | -3.093894 |
| 45 | 6 | -2.768500 | -2.238134 | -0.230400 |
| 46 | 1 | -3.688456 | -2.789525 | -0.001339 |
| 47 | 1 | -2.146300 | -2.886460 | -0.851606 |
| 48 | 6 | 0.078860 | 3.628821 | 0.347847 |
| 49 | 1 | -0.284175 | 4.626823 | 0.605972 |

| | | | | |
|---|---|---|---|---|
| 50 | 1 | 0.270145 | 3.596692 | -0.722529 |
| 51 | 6 | -1.429815 | -3.138176 | 1.574110 |
| 52 | 1 | -2.155855 | -3.961922 | 1.539309 |
| 53 | 1 | -1.227541 | -2.941454 | 2.626996 |
| 54 | 6 | -3.518550 | 1.100523 | -2.759204 |
| 55 | 1 | -3.617456 | 1.938170 | -3.434578 |
| 56 | 6 | -1.138775 | 1.817906 | -2.299577 |
| 57 | 6 | -2.333011 | 0.927885 | -2.060931 |
| 58 | 6 | -2.936449 | -1.297161 | 1.981484 |
| 59 | 1 | -3.507052 | -2.073418 | 2.507621 |
| 60 | 1 | -3.660142 | -0.679881 | 1.446674 |
| 61 | 6 | -3.102909 | -1.021292 | -1.053861 |
| 62 | 6 | -1.733359 | 2.986568 | 1.761266 |
| 63 | 1 | -2.394934 | 3.827886 | 1.534320 |
| 64 | 1 | -1.074480 | 3.264556 | 2.587998 |
| 65 | 6 | -2.537978 | 1.773546 | 2.134237 |
| 66 | 1 | -3.098365 | 1.973934 | 3.052489 |
| 67 | 1 | -3.245575 | 1.515852 | 1.340453 |
| 68 | 6 | -4.317999 | -0.909145 | -1.726415 |
| 69 | 1 | -5.076928 | -1.667295 | -1.585269 |
| 70 | 6 | -2.188028 | -0.440981 | 2.982945 |
| 71 | 1 | -2.862832 | -0.119095 | 3.781467 |
| 72 | 1 | -1.354856 | -0.980333 | 3.432532 |
| 73 | 7 | 1.404591 | -3.254403 | -2.577162 |
| 74 | 1 | 0.584011 | -3.820776 | -2.735074 |
| 75 | 1 | 2.282584 | -3.595122 | -2.932838 |

---------------------------------------------------------------------

E(RTPSSh) = -2027.90372627 Hartree
Zero-point correction= 0.622059 (Hartree/Particle)
Thermal correction to Energy= 0.658643
Thermal correction to Enthalpy= 0.659587
Thermal correction to Gibbs Free Energy= 0.555461
Sum of electronic and zero-point Energies= -2027.281668
Sum of electronic and thermal Energies= -2027.245083
Sum of electronic and thermal Enthalpies= -2027.244139
Sum of electronic and thermal Free Energies= -2027.348266

**Table S49**: Cartesian coordinates (Å) of the Δ(δλλ)(δλλ) conformer of **[Bi(macropapam)]$^{2+}$** from geometry optimizations (0 imaginary frequencies).

| Center Number | Atomic Number | Coordinates (Angstroms) X | Y | Z |
|---|---|---|---|---|
| 1 | 6 | 1.461795 | 3.445652 | -0.380965 |
| 2 | 7 | 0.977033 | 2.643533 | 0.769408 |
| 3 | 8 | 2.001410 | 1.493032 | -1.678731 |
| 4 | 6 | 2.484733 | 2.777372 | -1.271589 |
| 5 | 6 | 1.885775 | 2.675985 | 1.936789 |
| 6 | 6 | 3.084000 | 1.747135 | 1.825131 |
| 7 | 8 | 2.702298 | 0.377009 | 1.659532 |
| 8 | 6 | 2.520336 | -0.325279 | 2.896121 |
| 9 | 6 | 2.486376 | -1.796543 | 2.588511 |
| 10 | 8 | 1.387167 | -2.067942 | 1.709949 |
| 11 | 8 | 2.367410 | -1.211448 | -1.585231 |
| 12 | 6 | 2.478778 | -0.537151 | -2.850385 |
| 13 | 6 | 2.942812 | 0.861976 | -2.559945 |
| 14 | 7 | 0.022498 | -2.799680 | -0.717155 |
| 15 | 6 | 0.922735 | -3.173091 | -1.832523 |
| 16 | 6 | 2.337176 | -2.643257 | -1.679756 |
| 17 | 6 | 1.340497 | -3.450971 | 1.357859 |
| 18 | 6 | 0.145887 | -3.695797 | 0.462250 |
| 19 | 6 | -0.335033 | 3.192159 | 1.164448 |
| 20 | 6 | -1.375841 | -2.872688 | -1.184545 |
| 21 | 6 | -2.309013 | -2.194241 | -0.216917 |
| 22 | 6 | -1.385560 | 2.867442 | 0.134497 |
| 23 | 6 | -2.550882 | -0.702445 | 1.540165 |
| 24 | 6 | -3.884442 | -1.037335 | 1.713007 |
| 25 | 6 | -4.445736 | -1.978642 | 0.853725 |
| 26 | 6 | -3.643174 | -2.588303 | -0.094741 |
| 27 | 6 | -2.461499 | 3.717313 | -0.114577 |
| 28 | 6 | -3.361740 | 3.400976 | -1.122792 |
| 29 | 6 | -3.137461 | 2.271419 | -1.902605 |
| 30 | 6 | -2.044668 | 1.473671 | -1.603690 |
| 31 | 83 | 0.406215 | -0.075773 | -0.061384 |
| 32 | 1 | -0.291761 | 4.280880 | 1.291945 |
| 33 | 1 | -0.610176 | 2.763768 | 2.126276 |
| 34 | 1 | -1.698841 | -3.913592 | -1.307478 |
| 35 | 1 | -1.442194 | -2.388289 | -2.157838 |
| 36 | 1 | -4.486370 | -0.592832 | 2.492787 |
| 37 | 1 | -5.486428 | -2.256114 | 0.955445 |
| 38 | 1 | -4.029301 | -3.372420 | -0.732561 |
| 39 | 1 | -2.571379 | 4.623110 | 0.467354 |
| 40 | 1 | -4.205921 | 4.048129 | -1.323446 |
| 41 | 1 | -3.770529 | 2.010610 | -2.738565 |
| 42 | 7 | -1.224589 | 1.746200 | -0.578444 |
| 43 | 7 | -1.804893 | -1.228658 | 0.555110 |
| 44 | 6 | -1.766427 | 0.185487 | 2.451365 |
| 45 | 8 | -0.583566 | 0.465251 | 2.164385 |
| 46 | 6 | -1.649168 | 0.301304 | -2.463997 |
| 47 | 8 | -0.550193 | -0.295396 | -2.112749 |
| 48 | 8 | -2.328291 | -0.008778 | -3.436511 |
| 49 | 1 | 1.877662 | 4.398613 | -0.029610 |

| | | | | |
|---|---|---|---|---|
| 50 | 1 | 2.623330 | 3.405553 | -2.158317 |
| 51 | 1 | 3.461138 | 2.660969 | -0.795898 |
| 52 | 1 | 0.163571 | -4.742919 | 0.135214 |
| 53 | 1 | -0.750960 | -3.558389 | 1.065146 |
| 54 | 1 | 1.213115 | -4.054757 | 2.263410 |
| 55 | 1 | 2.287620 | -3.747452 | 0.899222 |
| 56 | 1 | 3.924989 | 0.843924 | -2.076716 |
| 57 | 1 | 3.011870 | 1.434377 | -3.490300 |
| 58 | 1 | 1.517070 | -0.541906 | -3.366977 |
| 59 | 1 | 3.228127 | -1.041208 | -3.466258 |
| 60 | 1 | 0.594509 | 3.679486 | -0.997975 |
| 61 | 1 | 2.360574 | -2.362767 | 3.517559 |
| 62 | 1 | 3.419122 | -2.107955 | 2.106330 |
| 63 | 1 | 3.366349 | -0.123300 | 3.560393 |
| 64 | 1 | 1.596640 | -0.006501 | 3.386383 |
| 65 | 1 | 2.249649 | 3.697465 | 2.113721 |
| 66 | 1 | 1.298782 | 2.380834 | 2.806998 |
| 67 | 1 | 3.690031 | 1.852615 | 2.729741 |
| 68 | 1 | 3.717771 | 1.988132 | 0.975744 |
| 69 | 1 | 2.827996 | -3.010057 | -0.782922 |
| 70 | 1 | 2.930291 | -2.961892 | -2.540632 |
| 71 | 1 | 0.494464 | -2.757552 | -2.744489 |
| 72 | 1 | 0.960655 | -4.263924 | -1.948262 |
| 73 | 7 | -2.332924 | 0.627304 | 3.567033 |
| 74 | 1 | -3.266194 | 0.370189 | 3.843552 |
| 75 | 1 | -1.792410 | 1.186700 | 4.210040 |

---------------------------------------------------------------------

E(RTPSSh) = -2027.90010630 Hartree
Zero-point correction= 0.622200 (Hartree/Particle)
Thermal correction to Energy= 0.658955
Thermal correction to Enthalpy= 0.659899
Thermal correction to Gibbs Free Energy= 0.555217
Sum of electronic and zero-point Energies= -2027.277906
Sum of electronic and thermal Energies= -2027.241152
Sum of electronic and thermal Enthalpies= -2027.240207
Sum of electronic and thermal Free Energies= -2027.344889

**Table S50**: Cartesian coordinates (Å) of the Δ(λδλ)(λδλ) conformer of **[Bi(macropapam)]$^{2+}$** from geometry optimizations (0 imaginary frequencies).

| Center Number | Atomic Number | Coordinates (Angstroms) X | Y | Z |
|---|---|---|---|---|
| 1 | 6 | 2.219286 | 2.246755 | 1.949798 |
| 2 | 7 | 1.616948 | 2.325248 | 0.597366 |
| 3 | 8 | 2.292875 | -0.128209 | 2.040621 |
| 4 | 6 | 3.098501 | 1.039930 | 2.157972 |
| 5 | 6 | 0.820574 | 3.576411 | 0.480601 |
| 6 | 6 | -0.334230 | 3.662431 | 1.444919 |
| 7 | 8 | -1.182831 | 2.525325 | 1.276044 |
| 8 | 6 | -2.191669 | 2.513951 | 2.290674 |
| 9 | 6 | -3.126371 | 1.367555 | 2.037454 |
| 10 | 8 | -2.351489 | 0.169053 | 1.991863 |
| 11 | 8 | 1.168957 | -2.549686 | 1.408776 |
| 12 | 6 | 2.132103 | -2.464298 | 2.457165 |
| 13 | 6 | 3.066787 | -1.323381 | 2.171414 |
| 14 | 7 | -1.644723 | -2.340216 | 0.670464 |
| 15 | 6 | -0.850455 | -3.592926 | 0.638532 |
| 16 | 6 | 0.284071 | -3.644196 | 1.632600 |
| 17 | 6 | -3.169677 | -0.990539 | 2.123802 |
| 18 | 6 | -2.295371 | -2.210430 | 1.991542 |
| 19 | 6 | 2.659275 | 2.388147 | -0.441371 |
| 20 | 6 | -2.644223 | -2.429611 | -0.404628 |
| 21 | 6 | -3.074792 | -1.122386 | -1.017868 |
| 22 | 6 | 3.108019 | 1.067740 | -1.004958 |
| 23 | 6 | -2.473756 | 0.951896 | -1.870513 |
| 24 | 6 | -3.713911 | 1.137333 | -2.458642 |
| 25 | 6 | -4.674969 | 0.146577 | -2.292106 |
| 26 | 6 | -4.346360 | -0.997354 | -1.580577 |
| 27 | 6 | 4.397533 | 0.932476 | -1.529316 |
| 28 | 6 | 4.745421 | -0.227973 | -2.196196 |
| 29 | 6 | 3.789868 | -1.226887 | -2.357810 |
| 30 | 6 | 2.534754 | -1.028438 | -1.806031 |
| 31 | 83 | -0.014077 | 0.087781 | 0.049425 |
| 32 | 1 | 1.411025 | 2.210908 | 2.679277 |
| 33 | 1 | 2.813731 | 3.149603 | 2.143572 |
| 34 | 1 | 3.534275 | 1.089949 | 3.162030 |
| 35 | 1 | 3.927750 | 1.002475 | 1.441728 |
| 36 | 1 | 0.444351 | 3.620426 | -0.539239 |
| 37 | 1 | 1.468087 | 4.446947 | 0.649405 |
| 38 | 1 | -0.898763 | 4.576330 | 1.229389 |
| 39 | 1 | -0.002855 | 3.716370 | 2.487020 |
| 40 | 1 | -2.750968 | 3.455496 | 2.266445 |
| 41 | 1 | -1.714461 | 2.411634 | 3.271522 |
| 42 | 1 | -3.671665 | 1.495667 | 1.095990 |
| 43 | 1 | -3.852333 | 1.314453 | 2.855769 |
| 44 | 1 | 2.706368 | -3.396231 | 2.520923 |
| 45 | 1 | 1.617321 | -2.305066 | 3.412001 |
| 46 | 1 | 3.768481 | -1.222802 | 3.006241 |
| 47 | 1 | 3.640312 | -1.497551 | 1.254408 |
| 48 | 1 | -0.449660 | -3.695367 | -0.367477 |
| 49 | 1 | -1.501274 | -4.458080 | 0.830305 |

| | | | | |
|---|---|---|---|---|
| 50 | 1 | -0.074380 | -3.619912 | 2.667284 |
| 51 | 1 | 0.820185 | -4.590800 | 1.494348 |
| 52 | 1 | -3.971622 | -0.972027 | 1.376858 |
| 53 | 1 | -3.640409 | -0.996129 | 3.113226 |
| 54 | 1 | -1.516639 | -2.155228 | 2.751502 |
| 55 | 1 | -2.908983 | -3.099234 | 2.198489 |
| 56 | 1 | 3.529932 | 2.953009 | -0.090882 |
| 57 | 1 | 2.244288 | 2.939458 | -1.288994 |
| 58 | 1 | -3.531023 | -2.987717 | -0.080557 |
| 59 | 1 | -2.195627 | -3.001671 | -1.221137 |
| 60 | 1 | -3.897678 | 2.033017 | -3.034268 |
| 61 | 1 | -5.660206 | 0.256183 | -2.726622 |
| 62 | 1 | -5.060162 | -1.801946 | -1.461359 |
| 63 | 1 | 5.106259 | 1.741758 | -1.414384 |
| 64 | 1 | 5.739331 | -0.353021 | -2.604350 |
| 65 | 1 | 4.036171 | -2.123095 | -2.908892 |
| 66 | 7 | 2.215951 | 0.082299 | -1.119826 |
| 67 | 7 | -2.177551 | -0.137247 | -1.142442 |
| 68 | 6 | -1.349084 | 1.925955 | -2.088467 |
| 69 | 8 | -0.202639 | 1.586353 | -1.564246 |
| 70 | 8 | -1.517738 | 2.934572 | -2.758599 |
| 71 | 6 | 1.382022 | -1.955377 | -1.979301 |
| 72 | 8 | 0.260192 | -1.637222 | -1.511598 |
| 73 | 7 | 1.533870 | -3.079691 | -2.656308 |
| 74 | 1 | 2.413079 | -3.359970 | -3.058705 |
| 75 | 1 | 0.735995 | -3.678055 | -2.812952 |

---------------------------------------------------------------------

E(RTPSSh) = -2027.90170824 Hartree
Zero-point correction= 0.621568 (Hartree/Particle)
Thermal correction to Energy= 0.658570
Thermal correction to Enthalpy= 0.659514
Thermal correction to Gibbs Free Energy= 0.553923
Sum of electronic and zero-point Energies= -2027.280141
Sum of electronic and thermal Energies= -2027.243139
Sum of electronic and thermal Enthalpies= -2027.242194
Sum of electronic and thermal Free Energies= -2027.347785

**Table S51**: Cartesian coordinates (Å) of the Δ(λλδ)(λλδ) conformer of **[Bi(macropapam)]$^{2+}$** from geometry optimizations (0 imaginary frequencies).

| Center Number | Atomic Number | Coordinates (Angstroms) X | Y | Z |
|---|---|---|---|---|
| 1 | 6 | -0.967199 | -3.575121 | -0.853342 |
| 2 | 7 | -0.700252 | -2.859971 | 0.428580 |
| 3 | 8 | -2.244021 | -1.630166 | -1.366373 |
| 4 | 6 | -1.403232 | -2.630536 | -1.964689 |
| 5 | 6 | -1.778669 | -3.168415 | 1.394836 |
| 6 | 6 | -1.877307 | -2.199042 | 2.547864 |
| 7 | 8 | -2.255484 | -0.923927 | 2.022628 |
| 8 | 6 | -2.594064 | 0.009925 | 3.049530 |
| 9 | 6 | -3.184560 | 1.228013 | 2.378008 |
| 10 | 8 | -2.282311 | 1.827545 | 1.442876 |
| 11 | 8 | -2.272060 | 1.072434 | -1.897053 |
| 12 | 6 | -2.682301 | 0.169332 | -2.929426 |
| 13 | 6 | -3.230648 | -1.048780 | -2.232300 |
| 14 | 7 | -0.523984 | 2.831170 | -0.356886 |
| 15 | 6 | -1.636517 | 3.258151 | -1.241248 |
| 16 | 6 | -1.864951 | 2.343662 | -2.416727 |
| 17 | 6 | -1.240953 | 2.607403 | 2.037215 |
| 18 | 6 | -0.673209 | 3.519683 | 0.962111 |
| 19 | 6 | 0.620593 | -3.225710 | 0.954465 |
| 20 | 6 | 0.778930 | 3.155117 | -0.959805 |
| 21 | 6 | 1.888042 | 2.544203 | -0.144498 |
| 22 | 6 | 1.694726 | -2.669641 | 0.054393 |
| 23 | 6 | 2.468392 | 0.958349 | 1.445545 |
| 24 | 6 | 3.751482 | 1.467976 | 1.566093 |
| 25 | 6 | 4.108327 | 2.549288 | 0.763372 |
| 26 | 6 | 3.161021 | 3.113280 | -0.074560 |
| 27 | 6 | 2.911998 | -3.316214 | -0.149928 |
| 28 | 6 | 3.818467 | -2.792348 | -1.062409 |
| 29 | 6 | 3.470583 | -1.662975 | -1.796981 |
| 30 | 6 | 2.242835 | -1.072551 | -1.544893 |
| 31 | 83 | -0.482604 | 0.027857 | -0.068706 |
| 32 | 1 | -1.740396 | -4.326699 | -0.682169 |
| 33 | 1 | -0.071559 | -4.106329 | -1.179443 |
| 34 | 1 | -1.966226 | -3.179751 | -2.722575 |
| 35 | 1 | -0.548864 | -2.152261 | -2.450224 |
| 36 | 1 | -0.464485 | 1.951676 | 2.444861 |
| 37 | 1 | -1.641196 | 3.210071 | 2.858102 |
| 38 | 1 | -1.316450 | 4.392600 | 0.828996 |
| 39 | 1 | 0.297005 | 3.884380 | 1.300212 |
| 40 | 1 | 0.744022 | -4.313698 | 1.025544 |
| 41 | 1 | 0.722408 | -2.817540 | 1.959769 |
| 42 | 1 | 0.934527 | 4.239307 | -1.014539 |
| 43 | 1 | 0.806883 | 2.762356 | -1.976721 |
| 44 | 1 | 4.465452 | 1.058751 | 2.266801 |
| 45 | 1 | 5.104322 | 2.966943 | 0.826429 |
| 46 | 1 | 3.387441 | 3.994708 | -0.659832 |
| 47 | 1 | 3.127199 | -4.230209 | 0.387999 |
| 48 | 1 | 4.769593 | -3.281959 | -1.227416 |
| 49 | 1 | 4.112703 | -1.250657 | -2.562124 |

| | | | | |
|---|---|---|---|---|
| 50 | 7 | 1.406920 | -1.543202 | -0.609240 |
| 51 | 7 | 1.584541 | 1.456714 | 0.567577 |
| 52 | 6 | 1.871648 | -0.104952 | 2.311263 |
| 53 | 8 | 0.691944 | -0.464534 | 2.104849 |
| 54 | 6 | 1.709169 | 0.073475 | -2.363989 |
| 55 | 8 | 0.493366 | 0.440638 | -2.069155 |
| 56 | 8 | 2.381909 | 0.565729 | -3.259952 |
| 57 | 1 | -2.673675 | 2.753665 | -3.029844 |
| 58 | 1 | -0.979042 | 2.216224 | -3.044010 |
| 59 | 1 | -1.458103 | 4.278521 | -1.602813 |
| 60 | 1 | -2.539638 | 3.265412 | -0.633950 |
| 61 | 1 | -2.656918 | -2.553261 | 3.231101 |
| 62 | 1 | -0.943801 | -2.106344 | 3.110450 |
| 63 | 1 | -2.718219 | -3.133597 | 0.845156 |
| 64 | 1 | -1.656510 | -4.185161 | 1.791661 |
| 65 | 1 | -1.705038 | 0.254110 | 3.641501 |
| 66 | 1 | -3.343146 | -0.429615 | 3.717848 |
| 67 | 1 | -3.483418 | 1.963274 | 3.131749 |
| 68 | 1 | -4.058696 | 0.940723 | 1.793549 |
| 69 | 1 | -1.830074 | -0.070442 | -3.573366 |
| 70 | 1 | -3.465701 | 0.636718 | -3.534772 |
| 71 | 1 | -4.061392 | -0.765283 | -1.585665 |
| 72 | 1 | -3.574405 | -1.790895 | -2.955974 |
| 73 | 7 | 2.589467 | -0.610366 | 3.304857 |
| 74 | 1 | 3.529083 | -0.309629 | 3.505817 |
| 75 | 1 | 2.173272 | -1.289879 | 3.924294 |

---------------------------------------------------------------------

E(RTPSSh) = -2027.88815540 Hartree
Zero-point correction= 0.621550 (Hartree/Particle)
Thermal correction to Energy= 0.658558
Thermal correction to Enthalpy= 0.659502
Thermal correction to Gibbs Free Energy= 0.554180
Sum of electronic and zero-point Energies= -2027.266605
Sum of electronic and thermal Energies= -2027.229597
Sum of electronic and thermal Enthalpies= -2027.228653
Sum of electronic and thermal Free Energies= -2027.333975

**Table S52**: Cartesian coordinates (Å) of the Δ(δδλ)(δδλ) conformer of **[Bi(macropapam)]$^{2+}$** from geometry optimizations (0 imaginary frequencies).

| Center Number | Atomic Number | Coordinates (Angstroms) X | Y | Z |
|---|---|---|---|---|
| 1 | 6 | -1.613842 | 3.290587 | 0.530380 |
| 2 | 7 | -1.089399 | 2.608984 | -0.678051 |
| 3 | 8 | -2.680400 | 1.266568 | 1.205399 |
| 4 | 6 | -2.869194 | 2.674123 | 1.105181 |
| 5 | 6 | -2.038984 | 2.661972 | -1.817516 |
| 6 | 6 | -2.995987 | 1.485968 | -1.885301 |
| 7 | 8 | -2.203361 | 0.294169 | -1.893212 |
| 8 | 6 | -2.730606 | -0.811806 | -2.630428 |
| 9 | 6 | -1.547392 | -1.699478 | -2.937949 |
| 10 | 8 | -0.848681 | -2.074116 | -1.735555 |
| 11 | 8 | -2.332641 | -1.312743 | 1.788060 |
| 12 | 6 | -3.675998 | -0.832193 | 1.739599 |
| 13 | 6 | -3.649563 | 0.643007 | 2.051493 |
| 14 | 7 | -0.000020 | -2.784293 | 0.958964 |
| 15 | 6 | -0.754060 | -3.022150 | 2.211061 |
| 16 | 6 | -2.224978 | -2.692886 | 2.119707 |
| 17 | 6 | -1.400609 | -3.231441 | -1.082548 |
| 18 | 6 | -0.366267 | -3.749291 | -0.107592 |
| 19 | 6 | 0.167052 | 3.277349 | -1.067943 |
| 20 | 6 | 1.433276 | -2.911245 | 1.269301 |
| 21 | 6 | 2.306270 | -2.326924 | 0.193189 |
| 22 | 6 | 1.260944 | 3.043188 | -0.061015 |
| 23 | 6 | 2.582693 | -0.760454 | -1.496682 |
| 24 | 6 | 3.839475 | -1.246981 | -1.818931 |
| 25 | 6 | 4.341323 | -2.317232 | -1.088018 |
| 26 | 6 | 3.556209 | -2.875446 | -0.090109 |
| 27 | 6 | 2.270492 | 3.986615 | 0.139499 |
| 28 | 6 | 3.234187 | 3.754223 | 1.105455 |
| 29 | 6 | 3.135237 | 2.615967 | 1.901113 |
| 30 | 6 | 2.098746 | 1.729175 | 1.653681 |
| 31 | 83 | -0.283520 | -0.053665 | -0.077188 |
| 32 | 1 | -1.446918 | 2.651793 | -2.732001 |
| 33 | 1 | -2.610130 | 3.598000 | -1.800109 |
| 34 | 1 | -3.553355 | 1.554708 | -2.823331 |
| 35 | 1 | -3.705807 | 1.446124 | -1.059970 |
| 36 | 1 | -3.171567 | -0.456852 | -3.566369 |
| 37 | 1 | -3.503684 | -1.323899 | -2.049556 |
| 38 | 1 | -0.822838 | -1.152175 | -3.538166 |
| 39 | 1 | -1.860563 | -2.599310 | -3.471829 |
| 40 | 1 | -4.290689 | -1.344081 | 2.486836 |
| 41 | 1 | -4.101247 | -1.019541 | 0.748269 |
| 42 | 1 | -4.641791 | 1.069148 | 1.868557 |
| 43 | 1 | -3.376976 | 0.817560 | 3.097764 |
| 44 | 1 | -0.310238 | -2.386508 | 2.976783 |
| 45 | 1 | -0.645118 | -4.068875 | 2.526165 |
| 46 | 1 | -2.751562 | -3.313121 | 1.386464 |
| 47 | 1 | -2.684326 | -2.876044 | 3.097684 |
| 48 | 1 | 0.022584 | 4.359140 | -1.179011 |
| 49 | 1 | 0.480156 | 2.877960 | -2.031894 |

| | | | | |
|---|---|---|---|---|
| 50 | 1 | 1.718692 | -3.958528 | 1.426727 |
| 51 | 1 | 1.620545 | -2.382075 | 2.203212 |
| 52 | 1 | 4.383128 | -0.798066 | -2.637666 |
| 53 | 1 | 5.316792 | -2.729229 | -1.312238 |
| 54 | 1 | 3.893274 | -3.739436 | 0.467468 |
| 55 | 1 | 2.280402 | 4.892273 | -0.452485 |
| 56 | 1 | 4.033487 | 4.464924 | 1.268197 |
| 57 | 1 | 3.850938 | 2.450763 | 2.693978 |
| 58 | 7 | 1.219303 | 1.918314 | 0.656776 |
| 59 | 7 | 1.854620 | -1.266491 | -0.489197 |
| 60 | 6 | 1.926711 | 0.317773 | -2.316660 |
| 61 | 8 | 0.700622 | 0.597952 | -1.989397 |
| 62 | 8 | 2.530167 | 0.841331 | -3.245603 |
| 63 | 6 | 1.772108 | 0.537264 | 2.495302 |
| 64 | 8 | 0.800088 | -0.180569 | 2.183736 |
| 65 | 1 | -0.832079 | 3.257539 | 1.288646 |
| 66 | 1 | -1.816923 | 4.346078 | 0.305014 |
| 67 | 1 | -3.039291 | 3.108316 | 2.096223 |
| 68 | 1 | -3.753286 | 2.885851 | 0.493153 |
| 69 | 1 | -0.726427 | -4.686726 | 0.332362 |
| 70 | 1 | 0.532239 | -3.983768 | -0.679829 |
| 71 | 1 | -1.589926 | -4.000613 | -1.836310 |
| 72 | 1 | -2.347993 | -2.975994 | -0.605230 |
| 73 | 7 | 2.500612 | 0.290987 | 3.577502 |
| 74 | 1 | 3.263780 | 0.879655 | 3.867111 |
| 75 | 1 | 2.253232 | -0.482149 | 4.176990 |

---------------------------------------------------------------------

E(RTPSSh) = -2027.89921385 Hartree
Zero-point correction= 0.621852 (Hartree/Particle)
Thermal correction to Energy= 0.658840
Thermal correction to Enthalpy= 0.659785
Thermal correction to Gibbs Free Energy= 0.554479
Sum of electronic and zero-point Energies= -2027.277362
Sum of electronic and thermal Energies= -2027.240373
Sum of electronic and thermal Enthalpies= -2027.239429
Sum of electronic and thermal Free Energies= -2027.344735

**Table S53**: Cartesian coordinates (Å) of the Δ(λδδ)(λδδ) conformer of **[Bi(macropapam)]$^{2+}$** from geometry optimizations (0 imaginary frequencies).

| Center Number | Atomic Number | Coordinates (Angstroms) X | Y | Z |
|---|---|---|---|---|
| 1 | 6 | -3.607052 | -1.469022 | 0.438782 |
| 2 | 7 | -2.759768 | -1.075534 | -0.725074 |
| 3 | 8 | -1.667423 | -1.750960 | 1.772684 |
| 4 | 6 | -2.987481 | -1.206499 | 1.798808 |
| 5 | 6 | -2.862093 | -2.118402 | -1.778483 |
| 6 | 6 | -1.673327 | -2.162247 | -2.705605 |
| 7 | 8 | -0.527791 | -2.497506 | -1.901291 |
| 8 | 6 | 0.589374 | -3.004341 | -2.649565 |
| 9 | 6 | 1.580030 | -1.908979 | -2.954479 |
| 10 | 8 | 1.991639 | -1.386173 | -1.682180 |
| 11 | 8 | 1.049501 | -2.360773 | 2.040432 |
| 12 | 6 | 0.032156 | -3.023159 | 2.797316 |
| 13 | 6 | -1.151247 | -2.122046 | 3.056500 |
| 14 | 7 | 2.946378 | -0.535870 | 0.814083 |
| 15 | 6 | 3.230138 | -1.445018 | 1.953072 |
| 16 | 6 | 2.034340 | -1.694299 | 2.840444 |
| 17 | 6 | 3.239807 | -0.680066 | -1.688942 |
| 18 | 6 | 3.858066 | -0.852329 | -0.314527 |
| 19 | 6 | -3.186140 | 0.221867 | -1.273460 |
| 20 | 6 | 3.096546 | 0.858397 | 1.254504 |
| 21 | 6 | 2.544774 | 1.847844 | 0.262343 |
| 22 | 6 | -2.854254 | 1.365596 | -0.353110 |
| 23 | 6 | 1.008044 | 2.310690 | -1.419703 |
| 24 | 6 | 1.558437 | 3.560205 | -1.656867 |
| 25 | 6 | 2.640499 | 3.961424 | -0.881906 |
| 26 | 6 | 3.153964 | 3.085857 | 0.064728 |
| 27 | 6 | -3.683888 | 2.486099 | -0.272475 |
| 28 | 6 | -3.369404 | 3.504834 | 0.610014 |
| 29 | 6 | -2.264071 | 3.360920 | 1.443477 |
| 30 | 6 | -1.490897 | 2.216875 | 1.318535 |
| 31 | 83 | 0.042355 | -0.510373 | -0.072240 |
| 32 | 1 | -3.778591 | -2.541677 | 0.368392 |
| 33 | 1 | -4.584936 | -0.978205 | 0.385016 |
| 34 | 1 | -3.586393 | -1.732073 | 2.549032 |
| 35 | 1 | -2.957580 | -0.150683 | 2.073263 |
| 36 | 1 | 0.237962 | -3.474502 | -3.570668 |
| 37 | 1 | 1.059722 | -3.759667 | -2.019109 |
| 38 | 1 | 1.145898 | -1.113626 | -3.565513 |
| 39 | 1 | 2.447511 | -2.330169 | -3.470009 |
| 40 | 1 | 0.443503 | -3.390702 | 3.741996 |
| 41 | 1 | -0.286583 | -3.875035 | 2.194878 |
| 42 | 1 | -1.916950 | -2.673992 | 3.611215 |
| 43 | 1 | -0.874781 | -1.229495 | 3.625616 |
| 44 | 1 | 3.083666 | 0.359514 | -1.978873 |
| 45 | 1 | 3.906205 | -1.139811 | -2.423347 |
| 46 | 1 | 4.137339 | -1.900450 | -0.221434 |
| 47 | 1 | 4.778865 | -0.258823 | -0.254421 |
| 48 | 1 | -4.266468 | 0.228647 | -1.463821 |
| 49 | 1 | -2.678018 | 0.380121 | -2.225260 |

| | | | | |
|---|---|---|---|---|
| 50 | 1 | 4.151386 | 1.101251 | 1.436971 |
| 51 | 1 | 2.566691 | 0.971676 | 2.200700 |
| 52 | 1 | 1.150897 | 4.175591 | -2.446054 |
| 53 | 1 | 3.100452 | 4.928738 | -1.037686 |
| 54 | 1 | 4.031907 | 3.342906 | 0.642751 |
| 55 | 1 | -4.569689 | 2.538310 | -0.891541 |
| 56 | 1 | -3.993179 | 4.385966 | 0.679237 |
| 57 | 1 | -2.039566 | 4.124972 | 2.174046 |
| 58 | 7 | -1.757231 | 1.269248 | 0.404106 |
| 59 | 7 | 1.464810 | 1.501388 | -0.451666 |
| 60 | 6 | -0.072261 | 1.739959 | -2.301618 |
| 61 | 8 | -0.403291 | 0.509707 | -2.041131 |
| 62 | 8 | -0.542257 | 2.406736 | -3.214874 |
| 63 | 6 | -0.361505 | 1.848433 | 2.225573 |
| 64 | 8 | 0.226968 | 0.759396 | 2.053169 |
| 65 | 1 | 2.341259 | -2.353077 | 3.659376 |
| 66 | 1 | 1.625251 | -0.775312 | 3.267422 |
| 67 | 1 | 4.062232 | -1.062850 | 2.558603 |
| 68 | 1 | 3.535528 | -2.405745 | 1.541502 |
| 69 | 1 | -1.834951 | -2.957145 | -3.438826 |
| 70 | 1 | -1.502251 | -1.221434 | -3.231672 |
| 71 | 1 | -2.921734 | -3.083864 | -1.278988 |
| 72 | 1 | -3.779063 | -1.987509 | -2.366933 |
| 73 | 7 | -0.033377 | 2.662668 | 3.219005 |
| 74 | 1 | -0.508658 | 3.532905 | 3.391755 |
| 75 | 1 | 0.697914 | 2.393943 | 3.860547 |

---------------------------------------------------------------------

E(RTPSSh) = -2027.88931478 Hartree
Zero-point correction= 0.622075 (Hartree/Particle)
Thermal correction to Energy= 0.658885
Thermal correction to Enthalpy= 0.659829
Thermal correction to Gibbs Free Energy= 0.554243
Sum of electronic and zero-point Energies= -2027.267239
Sum of electronic and thermal Energies= -2027.230430
Sum of electronic and thermal Enthalpies= -2027.229486
Sum of electronic and thermal Free Energies= -2027.335071

**Table S54**: Cartesian coordinates (Å) of the Δ(δλδ)(δλδ) conformer of **[Bi(macropapam)]$^{2+}$** from geometry optimizations (0 imaginary frequencies).

| Center Number | Atomic Number | Coordinates (Angstroms) X | Y | Z |
|---|---|---|---|---|
| 1 | 6 | -3.382947 | -1.378499 | 0.945507 |
| 2 | 7 | -2.750333 | -1.071184 | -0.361546 |
| 3 | 8 | -1.343091 | -1.976176 | 2.023280 |
| 4 | 6 | -2.655460 | -2.447801 | 1.721739 |
| 5 | 6 | -3.042727 | -2.174441 | -1.305481 |
| 6 | 6 | -2.127732 | -2.199213 | -2.500655 |
| 7 | 8 | -0.795052 | -2.422172 | -2.033566 |
| 8 | 6 | 0.099569 | -2.573214 | -3.136811 |
| 9 | 6 | 1.478101 | -2.814751 | -2.600962 |
| 10 | 8 | 1.890965 | -1.656860 | -1.864561 |
| 11 | 8 | 1.462985 | -1.970585 | 2.234314 |
| 12 | 6 | 0.621775 | -2.276412 | 3.342535 |
| 13 | 6 | -0.632463 | -2.921875 | 2.829719 |
| 14 | 7 | 2.979800 | -0.245674 | 0.430327 |
| 15 | 6 | 3.551391 | -1.137896 | 1.462879 |
| 16 | 6 | 2.661587 | -1.331369 | 2.664798 |
| 17 | 6 | 3.280400 | -1.741976 | -1.533456 |
| 18 | 6 | 3.692372 | -0.464951 | -0.847779 |
| 19 | 6 | -3.312421 | 0.186548 | -0.880864 |
| 20 | 6 | 3.142064 | 1.157999 | 0.834726 |
| 21 | 6 | 2.406083 | 2.082531 | -0.098194 |
| 22 | 6 | -2.885415 | 1.359118 | -0.041059 |
| 23 | 6 | 0.691441 | 2.358521 | -1.643392 |
| 24 | 6 | 1.114144 | 3.636535 | -1.969886 |
| 25 | 6 | 2.219054 | 4.154843 | -1.304177 |
| 26 | 6 | 2.886073 | 3.359392 | -0.383110 |
| 27 | 6 | -3.701783 | 2.482248 | 0.104434 |
| 28 | 6 | -3.307225 | 3.506672 | 0.947315 |
| 29 | 6 | -2.135898 | 3.362899 | 1.685830 |
| 30 | 6 | -1.377883 | 2.218697 | 1.497654 |
| 31 | 83 | 0.040386 | -0.424534 | -0.071757 |
| 32 | 1 | -4.409001 | 0.148912 | -0.893927 |
| 33 | 1 | -2.971317 | 0.328108 | -1.906028 |
| 34 | 1 | 4.201359 | 1.446906 | 0.847601 |
| 35 | 1 | 2.760874 | 1.273520 | 1.848392 |
| 36 | 1 | 0.590338 | 4.185268 | -2.739296 |
| 37 | 1 | 2.578655 | 5.151045 | -1.527231 |
| 38 | 1 | 3.786933 | 3.707868 | 0.104732 |
| 39 | 1 | -4.640511 | 2.531086 | -0.431203 |
| 40 | 1 | -3.918744 | 4.391572 | 1.062689 |
| 41 | 1 | -1.842471 | 4.130918 | 2.387094 |
| 42 | 7 | -1.721168 | 1.270966 | 0.609769 |
| 43 | 7 | 1.297225 | 1.625152 | -0.696950 |
| 44 | 6 | -0.420910 | 1.673541 | -2.390681 |
| 45 | 8 | -0.663742 | 0.450977 | -2.017680 |
| 46 | 8 | -1.001349 | 2.246931 | -3.303090 |
| 47 | 6 | -0.167511 | 1.853740 | 2.290503 |
| 48 | 8 | 0.461561 | 0.814305 | 1.995123 |
| 49 | 1 | -4.424148 | -1.690016 | 0.792404 |

| | | | | |
|---|---|---|---|---|
| 50 | 1 | -3.199423 | -2.634264 | 2.654436 |
| 51 | 1 | -2.591777 | -3.396147 | 1.177707 |
| 52 | 1 | 4.778237 | -0.478375 | -0.684052 |
| 53 | 1 | 3.472044 | 0.368431 | -1.514840 |
| 54 | 1 | 3.861186 | -1.853586 | -2.455115 |
| 55 | 1 | 3.454301 | -2.629201 | -0.916238 |
| 56 | 1 | 2.417150 | -0.387968 | 3.163802 |
| 57 | 1 | 3.183280 | -1.971917 | 3.385873 |
| 58 | 1 | 3.720120 | -2.113152 | 1.008742 |
| 59 | 1 | 4.527247 | -0.755875 | 1.792185 |
| 60 | 1 | -2.424129 | -3.025985 | -3.155290 |
| 61 | 1 | -2.159988 | -1.270849 | -3.077873 |
| 62 | 1 | -4.084769 | -2.112093 | -1.644534 |
| 63 | 1 | -2.927709 | -3.114999 | -0.769721 |
| 64 | 1 | -0.401736 | -3.813737 | 2.236518 |
| 65 | 1 | -1.257788 | -3.216627 | 3.679351 |
| 66 | 1 | 0.382476 | -1.359027 | 3.892383 |
| 67 | 1 | 1.134555 | -2.969026 | 4.020731 |
| 68 | 1 | -3.396121 | -0.469276 | 1.545725 |
| 69 | 1 | 2.166888 | -2.976105 | -3.436495 |
| 70 | 1 | 1.501238 | -3.693979 | -1.948267 |
| 71 | 1 | -0.210984 | -3.428837 | -3.746437 |
| 72 | 1 | 0.077554 | -1.669276 | -3.754533 |
| 73 | 7 | 0.193532 | 2.613951 | 3.313419 |
| 74 | 1 | -0.326783 | 3.427664 | 3.597438 |
| 75 | 1 | 0.987215 | 2.346198 | 3.876659 |

---------------------------------------------------------------------

E(RTPSSh) = -2027.90475140 Hartree
Zero-point correction= 0.621626 (Hartree/Particle)
Thermal correction to Energy= 0.658520
Thermal correction to Enthalpy= 0.659464
Thermal correction to Gibbs Free Energy= 0.554227
Sum of electronic and zero-point Energies= -2027.283125
Sum of electronic and thermal Energies= -2027.246232
Sum of electronic and thermal Enthalpies= -2027.245288
Sum of electronic and thermal Free Energies= -2027.350524

**Table S55**: Cartesian coordinates obtained for the transition state of the **[Bi(macropa)]**$^{+}$ Δ(λδλ)(λδλ) ⇆ Δ(λδλ)(λδδ) equilibrum from geometry optimizations (1 imaginary frequency)

| Center Number | Atomic Number | Coordinates (Angstroms) X | Y | Z |
|---|---|---|---|---|
| 1 | 6 | 2.096409 | 2.503512 | 1.818457 |
| 2 | 7 | 1.612870 | 2.419835 | 0.425738 |
| 3 | 8 | 2.046902 | 0.165026 | 2.244006 |
| 4 | 6 | 2.899305 | 1.305315 | 2.261381 |
| 5 | 6 | 0.800534 | 3.616795 | 0.095252 |
| 6 | 6 | -0.400450 | 3.821163 | 0.990001 |
| 7 | 8 | -1.207551 | 2.645333 | 0.995047 |
| 8 | 6 | -2.149109 | 2.679978 | 2.064975 |
| 9 | 6 | -3.007434 | 1.447706 | 2.004152 |
| 10 | 8 | -2.163106 | 0.294681 | 2.036056 |
| 11 | 8 | 1.156435 | -2.239914 | 1.333186 |
| 12 | 6 | 1.745140 | -2.152770 | 2.636465 |
| 13 | 6 | 2.737627 | -1.022576 | 2.623194 |
| 14 | 7 | -1.583730 | -2.360886 | 0.775199 |
| 15 | 6 | -0.835197 | -3.646299 | 0.780208 |
| 16 | 6 | 0.699010 | -3.537751 | 0.942786 |
| 17 | 6 | -2.909215 | -0.881672 | 2.334451 |
| 18 | 6 | -2.038390 | -2.100665 | 2.162538 |
| 19 | 6 | 2.735270 | 2.368913 | -0.523115 |
| 20 | 6 | -2.725501 | -2.461516 | -0.148881 |
| 21 | 6 | -3.160500 | -1.172195 | -0.801723 |
| 22 | 6 | 3.186351 | 0.994818 | -0.949317 |
| 23 | 6 | -2.550109 | 0.809723 | -1.846439 |
| 24 | 6 | -3.833860 | 1.021054 | -2.324308 |
| 25 | 6 | -4.818414 | 0.094981 | -1.998251 |
| 26 | 6 | -4.475872 | -1.017145 | -1.242850 |
| 27 | 6 | 4.506780 | 0.778910 | -1.346942 |
| 28 | 6 | 4.860590 | -0.433190 | -1.921212 |
| 29 | 6 | 3.879557 | -1.397563 | -2.122661 |
| 30 | 6 | 2.591844 | -1.123070 | -1.690415 |
| 31 | 83 | 0.018456 | -0.039959 | -0.049953 |
| 32 | 1 | 1.230557 | 2.584415 | 2.474130 |
| 33 | 1 | 2.711279 | 3.405302 | 1.955089 |
| 34 | 1 | 3.264506 | 1.480795 | 3.280019 |
| 35 | 1 | 3.772818 | 1.129740 | 1.621958 |
| 36 | 1 | 0.470575 | 3.500077 | -0.935471 |
| 37 | 1 | 1.415731 | 4.526177 | 0.159397 |
| 38 | 1 | -0.978885 | 4.670184 | 0.606820 |
| 39 | 1 | -0.105714 | 4.063874 | 2.016706 |
| 40 | 1 | -2.788377 | 3.567042 | 1.981788 |
| 41 | 1 | -1.612738 | 2.726188 | 3.020212 |
| 42 | 1 | -3.616189 | 1.432871 | 1.093205 |
| 43 | 1 | -3.679265 | 1.443052 | 2.869506 |
| 44 | 1 | 2.265100 | -3.084201 | 2.873948 |
| 45 | 1 | 0.971445 | -1.978421 | 3.390993 |
| 46 | 1 | 3.172669 | -0.904574 | 3.622711 |
| 47 | 1 | 3.544094 | -1.227238 | 1.909507 |
| 48 | 1 | -1.016023 | -4.148767 | -0.167638 |
| 49 | 1 | -1.245042 | -4.289635 | 1.566093 |

| | | | | |
|---|---|---|---|---|
| 50 | 1 | 1.039810 | -4.273643 | 1.672646 |
| 51 | 1 | 1.175837 | -3.764412 | -0.009467 |
| 52 | 1 | -3.799382 | -0.931185 | 1.696290 |
| 53 | 1 | -3.254400 | -0.843141 | 3.374195 |
| 54 | 1 | -1.153527 | -2.005955 | 2.791842 |
| 55 | 1 | -2.612177 | -2.964727 | 2.527316 |
| 56 | 1 | 3.597287 | 2.936365 | -0.152604 |
| 57 | 1 | 2.409846 | 2.863124 | -1.442819 |
| 58 | 1 | -3.592137 | -2.937211 | 0.326299 |
| 59 | 1 | -2.425418 | -3.110809 | -0.973944 |
| 60 | 1 | -4.033935 | 1.887726 | -2.937790 |
| 61 | 1 | -5.836164 | 0.229310 | -2.341864 |
| 62 | 1 | -5.212274 | -1.772057 | -0.999425 |
| 63 | 1 | 5.238062 | 1.565411 | -1.212878 |
| 64 | 1 | 5.882800 | -0.614956 | -2.227755 |
| 65 | 1 | 4.085408 | -2.341571 | -2.606264 |
| 66 | 7 | 2.269180 | 0.030957 | -1.086773 |
| 67 | 7 | -2.236139 | -0.247604 | -1.081676 |
| 68 | 6 | -1.407007 | 1.717706 | -2.225274 |
| 69 | 8 | -0.239484 | 1.374061 | -1.772079 |
| 70 | 8 | -1.607724 | 2.683049 | -2.955202 |
| 71 | 6 | 1.454852 | -2.077176 | -1.950993 |
| 72 | 8 | 0.288923 | -1.712132 | -1.508078 |
| 73 | 8 | 1.657287 | -3.112677 | -2.577057 |

---------------------------------------------------------------------

E(RTPSSh) = -2047.32577667 Hartree
Zero-point correction= 0.595626 (Hartree/Particle)
Thermal correction to Energy= 0.631545
Thermal correction to Enthalpy= 0.632490
Thermal correction to Gibbs Free Energy= 0.528603
Sum of electronic and zero-point Energies= -2046.730150
Sum of electronic and thermal Energies= -2046.694231
Sum of electronic and thermal Enthalpies= -2046.693287
Sum of electronic and thermal Free Energies= -2046.797174

**Table S56**: Cartesian coordinates (Å) of the Δ(λδλ)(λδδ) conformer of **[Bi(macropa)]⁺** from geometry optimizations (0 imaginary frequencies).

| Center Number | Atomic Number | Coordinates (Angstroms) X | Y | Z |
|---|---|---|---|---|
| 1 | 6 | -2.236028 | 2.436139 | -1.702895 |
| 2 | 7 | -1.739973 | 2.367097 | -0.310790 |
| 3 | 8 | -2.160182 | 0.086681 | -2.108986 |
| 4 | 6 | -3.020936 | 1.222484 | -2.138210 |
| 5 | 6 | -1.030245 | 3.626356 | 0.038298 |
| 6 | 6 | 0.139336 | 3.970962 | -0.851727 |
| 7 | 8 | 1.092139 | 2.916628 | -0.811013 |
| 8 | 6 | 2.178201 | 3.172843 | -1.696790 |
| 9 | 6 | 3.183010 | 2.062283 | -1.562812 |
| 10 | 8 | 2.530980 | 0.834353 | -1.872211 |
| 11 | 8 | -1.188242 | -2.359701 | -1.281381 |
| 12 | 6 | -1.846282 | -2.227007 | -2.543682 |
| 13 | 6 | -2.855797 | -1.118245 | -2.433504 |
| 14 | 7 | 1.787254 | -1.993502 | -1.368662 |
| 15 | 6 | 1.037304 | -3.127533 | -1.961081 |
| 16 | 6 | -0.258482 | -3.450889 | -1.243284 |
| 17 | 6 | 3.455088 | -0.224895 | -2.075754 |
| 18 | 6 | 2.685057 | -1.474272 | -2.426318 |
| 19 | 6 | -2.851297 | 2.228293 | 0.645277 |
| 20 | 6 | 2.548947 | -2.470078 | -0.205115 |
| 21 | 6 | 2.992316 | -1.402814 | 0.765407 |
| 22 | 6 | -3.235034 | 0.818067 | 1.007237 |
| 23 | 6 | 2.452224 | 0.470697 | 2.028631 |
| 24 | 6 | 3.651875 | 0.420679 | 2.722321 |
| 25 | 6 | 4.551466 | -0.591729 | 2.409105 |
| 26 | 6 | 4.213114 | -1.517125 | 1.432484 |
| 27 | 6 | -4.533820 | 0.519771 | 1.420844 |
| 28 | 6 | -4.822604 | -0.745889 | 1.909669 |
| 29 | 6 | -3.801498 | -1.685051 | 2.002246 |
| 30 | 6 | -2.536935 | -1.325404 | 1.565336 |
| 31 | 83 | -0.056740 | 0.030440 | 0.001187 |
| 32 | 1 | -1.375913 | 2.540881 | -2.363574 |
| 33 | 1 | -2.873876 | 3.322119 | -1.833086 |
| 34 | 1 | -3.383445 | 1.384563 | -3.159622 |
| 35 | 1 | -3.895466 | 1.045137 | -1.501246 |
| 36 | 1 | -0.676369 | 3.514007 | 1.062092 |
| 37 | 1 | -1.732842 | 4.471138 | 0.004244 |
| 38 | 1 | 0.589561 | 4.897462 | -0.473733 |
| 39 | 1 | -0.166461 | 4.156660 | -1.887828 |
| 40 | 1 | 2.654131 | 4.128269 | -1.443991 |
| 41 | 1 | 1.807042 | 3.230107 | -2.727371 |
| 42 | 1 | 3.593734 | 2.024209 | -0.546864 |
| 43 | 1 | 4.007513 | 2.240386 | -2.263412 |
| 44 | 1 | -2.355611 | -3.163878 | -2.794133 |
| 45 | 1 | -1.124580 | -1.995771 | -3.332881 |
| 46 | 1 | -3.373474 | -1.004081 | -3.392356 |
| 47 | 1 | -3.597664 | -1.338574 | -1.657750 |
| 48 | 1 | 1.661561 | -4.032044 | -1.989849 |

| | | | | |
|---|---|---|---|---|
| 49 | 1 | 0.818872 | -2.867362 | -2.996763 |
| 50 | 1 | -0.714325 | -4.331575 | -1.707741 |
| 51 | 1 | -0.096641 | -3.664147 | -0.188408 |
| 52 | 1 | 4.088947 | -0.356150 | -1.191450 |
| 53 | 1 | 4.116422 | 0.016385 | -2.917574 |
| 54 | 1 | 2.063838 | -1.255295 | -3.295976 |
| 55 | 1 | 3.407871 | -2.250746 | -2.718158 |
| 56 | 1 | -3.738421 | 2.775058 | 0.305131 |
| 57 | 1 | -2.536080 | 2.696771 | 1.581615 |
| 58 | 1 | 3.425592 | -3.051967 | -0.519374 |
| 59 | 1 | 1.900558 | -3.143029 | 0.361586 |
| 60 | 1 | 3.849331 | 1.153625 | 3.491279 |
| 61 | 1 | 5.498882 | -0.666199 | 2.927593 |
| 62 | 1 | 4.882605 | -2.328842 | 1.179428 |
| 63 | 1 | -5.299304 | 1.282650 | 1.363601 |
| 64 | 1 | -5.827327 | -0.991772 | 2.228956 |
| 65 | 1 | -3.959845 | -2.675119 | 2.404801 |
| 66 | 7 | -2.274965 | -0.111254 | 1.057567 |
| 67 | 7 | 2.150174 | -0.405175 | 1.059634 |
| 68 | 6 | 1.381251 | 1.470330 | 2.389494 |
| 69 | 8 | 0.235553 | 1.321487 | 1.790253 |
| 70 | 8 | 1.603767 | 2.328562 | 3.235559 |
| 71 | 6 | -1.359761 | -2.254153 | 1.700283 |
| 72 | 8 | -0.213620 | -1.769696 | 1.324532 |
| 73 | 8 | -1.509106 | -3.372376 | 2.180408 |

---------------------------------------------------------------------

E(RTPSSh) = -2047.33738273 Hartree
Zero-point correction= 0.595952 (Hartree/Particle)
Thermal correction to Energy= 0.632269
Thermal correction to Enthalpy= 0.633213
Thermal correction to Gibbs Free Energy= 0.528994
Sum of electronic and zero-point Energies= -2046.741430
Sum of electronic and thermal Energies= -2046.705114
Sum of electronic and thermal Enthalpies= -2046.704170
Sum of electronic and thermal Free Energies= -2046.808388

**Table S57**: Cartesian coordinates obtained for the transition state of the **[Bi(macropa)]**$^+$ Δ(λδλ)(λδδ) ⇆ Δ(δδλ)(λδδ) equilibrum from geometry optimizations (1 imaginary frequency).

| Center Number | Atomic Number | Coordinates (Angstroms) X | Y | Z |
|---|---|---|---|---|
| 1 | 6 | -2.219776 | 2.301392 | -1.917465 |
| 2 | 7 | -1.697455 | 2.347673 | -0.533274 |
| 3 | 8 | -2.150989 | -0.069564 | -2.146696 |
| 4 | 6 | -3.013829 | 1.059575 | -2.242516 |
| 5 | 6 | -0.948669 | 3.614479 | -0.324993 |
| 6 | 6 | 0.254576 | 3.799377 | -1.219325 |
| 7 | 8 | 1.177658 | 2.745305 | -0.976738 |
| 8 | 6 | 2.366162 | 2.886214 | -1.754097 |
| 9 | 6 | 3.379526 | 1.903918 | -1.216102 |
| 10 | 8 | 2.893425 | 0.564924 | -1.287741 |
| 11 | 8 | -1.148917 | -2.425051 | -1.159605 |
| 12 | 6 | -1.830628 | -2.404070 | -2.415380 |
| 13 | 6 | -2.846124 | -1.295600 | -2.376695 |
| 14 | 7 | 1.814788 | -1.961878 | -1.380130 |
| 15 | 6 | 1.079550 | -3.169796 | -1.849091 |
| 16 | 6 | -0.177626 | -3.474547 | -1.058696 |
| 17 | 6 | 3.215986 | -0.083390 | -2.505933 |
| 18 | 6 | 2.651220 | -1.517944 | -2.528091 |
| 19 | 6 | -2.794387 | 2.325933 | 0.449513 |
| 20 | 6 | 2.630003 | -2.358369 | -0.214215 |
| 21 | 6 | 3.000996 | -1.294090 | 0.789560 |
| 22 | 6 | -3.232771 | 0.958629 | 0.905037 |
| 23 | 6 | 2.344608 | 0.474625 | 2.146304 |
| 24 | 6 | 3.549033 | 0.469930 | 2.836103 |
| 25 | 6 | 4.514612 | -0.455861 | 2.462076 |
| 26 | 6 | 4.236748 | -1.345916 | 1.432961 |
| 27 | 6 | -4.540939 | 0.733816 | 1.335016 |
| 28 | 6 | -4.875732 | -0.491129 | 1.893885 |
| 29 | 6 | -3.890880 | -1.461883 | 2.039275 |
| 30 | 6 | -2.612791 | -1.173499 | 1.587988 |
| 31 | 83 | -0.056586 | 0.035474 | 0.013038 |
| 32 | 1 | -1.372507 | 2.349683 | -2.600430 |
| 33 | 1 | -2.856921 | 3.176970 | -2.106708 |
| 34 | 1 | -3.398324 | 1.145967 | -3.265254 |
| 35 | 1 | -3.874647 | 0.931329 | -1.576038 |
| 36 | 1 | -0.615818 | 3.618941 | 0.712591 |
| 37 | 1 | -1.617507 | 4.473452 | -0.476688 |
| 38 | 1 | 0.715706 | 4.761961 | -0.966262 |
| 39 | 1 | -0.009927 | 3.832912 | -2.282143 |
| 40 | 1 | 2.759576 | 3.905176 | -1.654322 |
| 41 | 1 | 2.143203 | 2.705234 | -2.812397 |
| 42 | 1 | 3.559923 | 2.109049 | -0.159818 |
| 43 | 1 | 4.326394 | 2.000320 | -1.757421 |
| 44 | 1 | -2.335267 | -3.362690 | -2.578228 |
| 45 | 1 | -1.126953 | -2.232709 | -3.235381 |
| 46 | 1 | -3.373317 | -1.253436 | -3.336392 |
| 47 | 1 | -3.580384 | -1.461833 | -1.580517 |
| 48 | 1 | 1.733203 | -4.053157 | -1.828741 |
| 49 | 1 | 0.809532 | -3.008701 | -2.891423 |

| | | | | |
|---|---|---|---|---|
| 50 | 1 | -0.611931 | -4.409273 | -1.429074 |
| 51 | 1 | 0.022347 | -3.585085 | 0.005410 |
| 52 | 1 | 4.305603 | -0.108678 | -2.620113 |
| 53 | 1 | 2.805921 | 0.478115 | -3.352401 |
| 54 | 1 | 2.022273 | -1.602848 | -3.411823 |
| 55 | 1 | 3.479748 | -2.226845 | -2.656257 |
| 56 | 1 | -3.662547 | 2.889538 | 0.087696 |
| 57 | 1 | -2.437255 | 2.838529 | 1.346690 |
| 58 | 1 | 3.546228 | -2.865640 | -0.541417 |
| 59 | 1 | 2.048583 | -3.093383 | 0.349308 |
| 60 | 1 | 3.705640 | 1.181725 | 3.633742 |
| 61 | 1 | 5.472178 | -0.486508 | 2.966379 |
| 62 | 1 | 4.966520 | -2.083576 | 1.125469 |
| 63 | 1 | -5.278138 | 1.519549 | 1.233431 |
| 64 | 1 | -5.888507 | -0.680755 | 2.225842 |
| 65 | 1 | -4.086509 | -2.421424 | 2.495889 |
| 66 | 7 | -2.307818 | -0.000774 | 1.012685 |
| 67 | 7 | 2.090083 | -0.384438 | 1.152786 |
| 68 | 6 | 1.246164 | 1.449091 | 2.494097 |
| 69 | 8 | 0.164271 | 1.379804 | 1.773507 |
| 70 | 8 | 1.399562 | 2.239965 | 3.418086 |
| 71 | 6 | -1.466860 | -2.130919 | 1.788417 |
| 72 | 8 | -0.299049 | -1.696340 | 1.420440 |
| 73 | 8 | -1.661673 | -3.220308 | 2.316799 |

---------------------------------------------------------------------

E(RTPSSh) = -2047.32238412
Zero-point correction= 0.595514 (Hartree/Particle)
Thermal correction to Energy= 0.631435
Thermal correction to Enthalpy= 0.632379
Thermal correction to Gibbs Free Energy= 0.528120
Sum of electronic and zero-point Energies= -2046.726870
Sum of electronic and thermal Energies= -2046.690949
Sum of electronic and thermal Enthalpies= -2046.690005
Sum of electronic and thermal Free Energies= -2046.794264

**Table S58**: Cartesian coordinates (Å) of the Δ(δδλ)(λδδ) conformer of **[Bi(macropa)]$^+$** from geometry optimizations (0 imaginary frequencies).

| Center Number | Atomic Number | Coordinates (Angstroms) X | Y | Z |
|---|---|---|---|---|
| 1 | 6 | 2.226958 | 2.287056 | 1.776032 |
| 2 | 7 | 1.769057 | 2.278462 | 0.369485 |
| 3 | 8 | 2.020219 | -0.064628 | 2.104081 |
| 4 | 6 | 2.938957 | 1.022646 | 2.194172 |
| 5 | 6 | 1.074721 | 3.552115 | 0.042970 |
| 6 | 6 | -0.102491 | 3.871844 | 0.934984 |
| 7 | 8 | -0.995811 | 2.762949 | 0.970921 |
| 8 | 6 | -2.011776 | 2.950828 | 1.956102 |
| 9 | 6 | -2.758111 | 1.657167 | 2.133565 |
| 10 | 8 | -1.816962 | 0.666891 | 2.546275 |
| 11 | 8 | 0.930290 | -2.425768 | 1.206439 |
| 12 | 6 | 1.545803 | -2.358734 | 2.494971 |
| 13 | 6 | 2.626617 | -1.313886 | 2.442625 |
| 14 | 7 | -2.026286 | -1.955019 | 1.080965 |
| 15 | 6 | -1.372677 | -3.117912 | 1.715024 |
| 16 | 6 | -0.036602 | -3.479341 | 1.090902 |
| 17 | 6 | -2.425328 | -0.506990 | 3.078504 |
| 18 | 6 | -3.033926 | -1.389158 | 2.003057 |
| 19 | 6 | 2.903093 | 2.151545 | -0.561805 |
| 20 | 6 | -2.683947 | -2.356530 | -0.168492 |
| 21 | 6 | -3.085559 | -1.179636 | -1.020495 |
| 22 | 6 | 3.256517 | 0.745310 | -0.969302 |
| 23 | 6 | -2.429273 | 0.778078 | -2.083448 |
| 24 | 6 | -3.636617 | 0.883227 | -2.759771 |
| 25 | 6 | -4.602869 | -0.089119 | -2.534910 |
| 26 | 6 | -4.317856 | -1.138688 | -1.670621 |
| 27 | 6 | 4.552973 | 0.421726 | -1.370339 |
| 28 | 6 | 4.812321 | -0.836676 | -1.893520 |
| 29 | 6 | 3.765965 | -1.741942 | -2.034165 |
| 30 | 6 | 2.504036 | -1.357944 | -1.611064 |
| 31 | 83 | 0.045991 | 0.042271 | -0.060476 |
| 32 | 1 | 1.352886 | 2.411373 | 2.414270 |
| 33 | 1 | 2.900562 | 3.137692 | 1.950358 |
| 34 | 1 | 3.277170 | 1.133750 | 3.230351 |
| 35 | 1 | 3.821464 | 0.818783 | 1.576729 |
| 36 | 1 | 0.739378 | 3.468369 | -0.988874 |
| 37 | 1 | 1.778418 | 4.393337 | 0.112903 |
| 38 | 1 | -0.610026 | 4.755456 | 0.529894 |
| 39 | 1 | 0.213974 | 4.114875 | 1.955553 |
| 40 | 1 | -2.703202 | 3.741568 | 1.642053 |
| 41 | 1 | -1.546734 | 3.247111 | 2.903444 |
| 42 | 1 | -3.248877 | 1.367037 | 1.198389 |
| 43 | 1 | -3.529327 | 1.789732 | 2.901149 |
| 44 | 1 | 1.986810 | -3.329790 | 2.744084 |
| 45 | 1 | 0.810558 | -2.098590 | 3.262399 |
| 46 | 1 | 3.107888 | -1.240621 | 3.423903 |
| 47 | 1 | 3.385869 | -1.572348 | 1.696152 |
| 48 | 1 | -2.029177 | -3.999538 | 1.674033 |
| 49 | 1 | -1.227478 | -2.887115 | 2.770289 |

| | | | | |
|---|---|---|---|---|
| 50 | 1 | 0.353164 | -4.382000 | 1.572256 |
| 51 | 1 | -0.131764 | -3.673287 | 0.024213 |
| 52 | 1 | -3.204962 | -0.228632 | 3.797327 |
| 53 | 1 | -1.636478 | -1.025777 | 3.624059 |
| 54 | 1 | -3.612897 | -2.193180 | 2.479837 |
| 55 | 1 | -3.740875 | -0.801101 | 1.415235 |
| 56 | 1 | 3.793706 | 2.658628 | -0.173507 |
| 57 | 1 | 2.626917 | 2.666853 | -1.485696 |
| 58 | 1 | -3.565039 | -2.982744 | 0.023910 |
| 59 | 1 | -1.977256 | -2.949346 | -0.752878 |
| 60 | 1 | -3.788590 | 1.702430 | -3.447848 |
| 61 | 1 | -5.559958 | -0.040661 | -3.038502 |
| 62 | 1 | -5.037707 | -1.927826 | -1.497150 |
| 63 | 1 | 5.339843 | 1.158633 | -1.275782 |
| 64 | 1 | 5.815032 | -1.102794 | -2.202727 |
| 65 | 1 | 3.903328 | -2.722975 | -2.465534 |
| 66 | 7 | 2.271313 | -0.152751 | -1.068683 |
| 67 | 7 | -2.182069 | -0.208604 | -1.212678 |
| 68 | 6 | -1.287083 | 1.722640 | -2.365219 |
| 69 | 8 | -0.152499 | 1.427121 | -1.800860 |
| 70 | 8 | -1.446755 | 2.669549 | -3.127112 |
| 71 | 6 | 1.297511 | -2.239964 | -1.803265 |
| 72 | 8 | 0.160893 | -1.719095 | -1.451070 |
| 73 | 8 | 1.422363 | -3.351566 | -2.305842 |

---------------------------------------------------------------------

E(RTPSSh) = -2047.33869631 Hartree
Zero-point correction= 0.596062 (Hartree/Particle)
Thermal correction to Energy= 0.632346
Thermal correction to Enthalpy= 0.633290
Thermal correction to Gibbs Free Energy= 0.529038
Sum of electronic and zero-point Energies= -2046.742635
Sum of electronic and thermal Energies= -2046.706350
Sum of electronic and thermal Enthalpies= -2046.705406
Sum of electronic and thermal Free Energies= -2046.809659

**Table S59**: Cartesian coordinates obtained for the transition state of the **[Bi(macropa)]**$^{+}$ Δ(δδλ)(λδδ)⇆ Δ(δλλ)(λδδ) equilibrum from geometry optimizations (1 imaginary frequency).

| Center Number | Atomic Number | Coordinates (Angstroms) X | Y | Z |
|---|---|---|---|---|
| 1 | 6 | 2.086117 | 2.220412 | 2.014080 |
| 2 | 7 | 1.563047 | 2.327603 | 0.631794 |
| 3 | 8 | 2.131399 | -0.161311 | 2.093135 |
| 4 | 6 | 2.938889 | 0.999937 | 2.262006 |
| 5 | 6 | 0.745367 | 3.563032 | 0.509018 |
| 6 | 6 | -0.527935 | 3.577180 | 1.320357 |
| 7 | 8 | -1.430636 | 2.597924 | 0.803510 |
| 8 | 6 | -2.702963 | 2.634936 | 1.452981 |
| 9 | 6 | -2.983339 | 1.366496 | 2.287075 |
| 10 | 8 | -1.807250 | 0.563024 | 2.373168 |
| 11 | 8 | 1.227478 | -2.498548 | 0.976123 |
| 12 | 6 | 1.933820 | -2.525550 | 2.216752 |
| 13 | 6 | 2.889424 | -1.364109 | 2.233634 |
| 14 | 7 | -1.761416 | -2.145943 | 1.028502 |
| 15 | 6 | -1.010366 | -3.303506 | 1.561177 |
| 16 | 6 | 0.293684 | -3.575435 | 0.833829 |
| 17 | 6 | -2.074424 | -0.686250 | 3.019473 |
| 18 | 6 | -2.713474 | -1.674726 | 2.059970 |
| 19 | 6 | 2.666976 | 2.424917 | -0.341862 |
| 20 | 6 | -2.488088 | -2.523430 | -0.190730 |
| 21 | 6 | -2.997262 | -1.335129 | -0.967681 |
| 22 | 6 | 3.145936 | 1.113938 | -0.910471 |
| 23 | 6 | -2.500884 | 0.707806 | -1.963445 |
| 24 | 6 | -3.736052 | 0.763012 | -2.593767 |
| 25 | 6 | -4.631552 | -0.277488 | -2.378859 |
| 26 | 6 | -4.252929 | -1.343490 | -1.572103 |
| 27 | 6 | 4.452185 | 0.966388 | -1.377590 |
| 28 | 6 | 4.812956 | -0.201306 | -2.035012 |
| 29 | 6 | 3.857414 | -1.191497 | -2.237045 |
| 30 | 6 | 2.579661 | -0.981617 | -1.743353 |
| 31 | 83 | 0.029464 | 0.043691 | -0.037175 |
| 32 | 1 | 1.238140 | 2.189616 | 2.697019 |
| 33 | 1 | 2.681287 | 3.112342 | 2.254804 |
| 34 | 1 | 3.320700 | 1.039757 | 3.288584 |
| 35 | 1 | 3.803510 | 0.956511 | 1.589348 |
| 36 | 1 | 0.481377 | 3.671312 | -0.542085 |
| 37 | 1 | 1.345220 | 4.434366 | 0.806093 |
| 38 | 1 | -0.972765 | 4.572918 | 1.214767 |
| 39 | 1 | -0.353654 | 3.402810 | 2.386627 |
| 40 | 1 | -3.463195 | 2.735471 | 0.676458 |
| 41 | 1 | -2.755797 | 3.519255 | 2.090154 |
| 42 | 1 | -3.794597 | 0.797131 | 1.825090 |
| 43 | 1 | -3.302115 | 1.639653 | 3.298826 |
| 44 | 1 | 2.493733 | -3.463251 | 2.304619 |
| 45 | 1 | 1.240670 | -2.452229 | 3.060552 |
| 46 | 1 | 3.432938 | -1.354934 | 3.184885 |
| 47 | 1 | 3.614797 | -1.442316 | 1.415951 |
| 48 | 1 | -1.632961 | -4.209319 | 1.529589 |
| 49 | 1 | -0.801929 | -3.105061 | 2.613229 |

| | | | | |
|---|---|---|---|---|
| 50 | 1 | 0.737215 | -4.498283 | 1.221556 |
| 51 | 1 | 0.139899 | -3.692822 | -0.237661 |
| 52 | 1 | -2.725844 | -0.527286 | 3.885273 |
| 53 | 1 | -1.111562 | -1.048555 | 3.381746 |
| 54 | 1 | -3.125045 | -2.526614 | 2.617101 |
| 55 | 1 | -3.554207 | -1.193407 | 1.558349 |
| 56 | 1 | 3.515035 | 2.978864 | 0.076660 |
| 57 | 1 | 2.302150 | 3.003881 | -1.193986 |
| 58 | 1 | -3.324885 | -3.197603 | 0.031798 |
| 59 | 1 | -1.797768 | -3.060243 | -0.845372 |
| 60 | 1 | -3.964413 | 1.598679 | -3.239580 |
| 61 | 1 | -5.607587 | -0.267882 | -2.847126 |
| 62 | 1 | -4.917239 | -2.182285 | -1.410425 |
| 63 | 1 | 5.166948 | 1.765298 | -1.229221 |
| 64 | 1 | 5.824373 | -0.331789 | -2.398223 |
| 65 | 1 | 4.076262 | -2.106757 | -2.767994 |
| 66 | 7 | 2.251297 | 0.135374 | -1.077805 |
| 67 | 7 | -2.165729 | -0.300017 | -1.148197 |
| 68 | 6 | -1.427380 | 1.736192 | -2.235385 |
| 69 | 8 | -0.261822 | 1.495762 | -1.712969 |
| 70 | 8 | -1.672786 | 2.695053 | -2.959344 |
| 71 | 6 | 1.459170 | -1.961948 | -1.987064 |
| 72 | 8 | 0.288205 | -1.596070 | -1.563328 |
| 73 | 8 | 1.681126 | -3.005244 | -2.593781 |

---------------------------------------------------------------------

E(RTPSSh) = -2047.32934178 Hartree
Zero-point correction= 0.595891 (Hartree/Particle)
Thermal correction to Energy= 0.631610
Thermal correction to Enthalpy= 0.632554
Thermal correction to Gibbs Free Energy= 0.528525
Sum of electronic and zero-point Energies= -2046.733450
Sum of electronic and thermal Energies= -2046.697732
Sum of electronic and thermal Enthalpies= -2046.696788
Sum of electronic and thermal Free Energies= -2046.800817

**Table S60**: Cartesian coordinates (Å) of the Δ(δλλ)(λδδ) conformer of **[Bi(macropa)]$^+$** from geometry optimizations (0 imaginary frequencies). This conformation of the complex corresponds to the previously published crystal structure.

| Center Number | Atomic Number | Coordinates (Angstroms) X | Y | Z |
|---|---|---|---|---|
| 1 | 6 | -2.093093 | -1.849445 | 2.310362 |
| 2 | 7 | -1.606935 | -2.146811 | 0.943159 |
| 3 | 8 | -1.986565 | 0.516338 | 2.082472 |
| 4 | 6 | -2.863969 | -0.554525 | 2.418791 |
| 5 | 6 | -0.783405 | -3.384320 | 0.951023 |
| 6 | 6 | 0.517278 | -3.264880 | 1.712663 |
| 7 | 8 | 1.318169 | -2.235097 | 1.122193 |
| 8 | 6 | 2.548519 | -2.008758 | 1.824069 |
| 9 | 6 | 2.338034 | -1.196613 | 3.089360 |
| 10 | 8 | 1.623315 | 0.008204 | 2.807444 |
| 11 | 8 | -0.960319 | 2.627884 | 0.667937 |
| 12 | 6 | -1.577158 | 2.849301 | 1.937184 |
| 13 | 6 | -2.630061 | 1.791140 | 2.131090 |
| 14 | 7 | 1.987043 | 2.102581 | 0.639711 |
| 15 | 6 | 1.351678 | 3.386731 | 1.007503 |
| 16 | 6 | 0.016429 | 3.617966 | 0.320348 |
| 17 | 6 | 2.358438 | 1.220534 | 2.965963 |
| 18 | 6 | 2.977627 | 1.713962 | 1.667900 |
| 19 | 6 | -2.737086 | -2.347446 | 0.016159 |
| 20 | 6 | 2.669655 | 2.243773 | -0.654447 |
| 21 | 6 | 3.052625 | 0.925582 | -1.276282 |
| 22 | 6 | -3.207730 | -1.105175 | -0.697545 |
| 23 | 6 | 2.353518 | -1.180400 | -1.968845 |
| 24 | 6 | 3.556759 | -1.427601 | -2.614837 |
| 25 | 6 | 4.542769 | -0.449745 | -2.566882 |
| 26 | 6 | 4.283113 | 0.743686 | -1.904554 |
| 27 | 6 | -4.524145 | -0.977098 | -1.140060 |
| 28 | 6 | -4.874369 | 0.111884 | -1.926505 |
| 29 | 6 | -3.899442 | 1.038050 | -2.282852 |
| 30 | 6 | -2.610933 | 0.850685 | -1.808068 |
| 31 | 83 | -0.047953 | -0.051961 | -0.062340 |
| 32 | 1 | -1.231281 | -1.787332 | 2.973060 |
| 33 | 1 | -2.733505 | -2.668470 | 2.664734 |
| 34 | 1 | -3.213171 | -0.438032 | 3.450867 |
| 35 | 1 | -3.744509 | -0.539632 | 1.766053 |
| 36 | 1 | -0.559808 | -3.622913 | -0.087201 |
| 37 | 1 | -1.353349 | -4.217494 | 1.383638 |
| 38 | 1 | 1.047721 | -4.220127 | 1.636467 |
| 39 | 1 | 0.354964 | -3.058824 | 2.773544 |
| 40 | 1 | 3.192004 | -1.476351 | 1.124702 |
| 41 | 1 | 3.017583 | -2.968900 | 2.065549 |
| 42 | 1 | 3.310740 | -0.979159 | 3.540269 |
| 43 | 1 | 1.751073 | -1.758664 | 3.820252 |
| 44 | 1 | -2.044012 | 3.840092 | 1.954550 |
| 45 | 1 | -0.839282 | 2.794565 | 2.743418 |
| 46 | 1 | -3.110891 | 1.930575 | 3.105401 |
| 47 | 1 | -3.394611 | 1.851678 | 1.348659 |
| 48 | 1 | 2.019947 | 4.227748 | 0.772627 |

| | | | | |
|---|---|---|---|---|
| 49 | 1 | 1.206683 | 3.397741 | 2.086651 |
| 50 | 1 | -0.359546 | 4.610866 | 0.587674 |
| 51 | 1 | 0.109201 | 3.564732 | -0.763017 |
| 52 | 1 | 3.155466 | 1.085463 | 3.703378 |
| 53 | 1 | 1.657761 | 1.950402 | 3.374713 |
| 54 | 1 | 3.639317 | 2.563823 | 1.894821 |
| 55 | 1 | 3.605709 | 0.925123 | 1.249468 |
| 56 | 1 | -3.582284 | -2.826995 | 0.521871 |
| 57 | 1 | -2.402640 | -3.033291 | -0.765960 |
| 58 | 1 | 3.562262 | 2.876990 | -0.566782 |
| 59 | 1 | 1.983797 | 2.736199 | -1.347687 |
| 60 | 1 | 3.692488 | -2.360456 | -3.143023 |
| 61 | 1 | 5.497641 | -0.607675 | -3.052017 |
| 62 | 1 | 5.020363 | 1.535186 | -1.873252 |
| 63 | 1 | -5.254808 | -1.729107 | -0.872386 |
| 64 | 1 | -5.893797 | 0.228215 | -2.271759 |
| 65 | 1 | -4.111528 | 1.884499 | -2.920119 |
| 66 | 7 | -2.295305 | -0.181976 | -1.013646 |
| 67 | 7 | 2.129899 | -0.045366 | -1.294664 |
| 68 | 6 | 1.194667 | -2.144500 | -2.059474 |
| 69 | 8 | 0.075861 | -1.739879 | -1.539154 |
| 70 | 8 | 1.336216 | -3.213626 | -2.644761 |
| 71 | 6 | -1.464344 | 1.743380 | -2.220075 |
| 72 | 8 | -0.291460 | 1.377775 | -1.804345 |
| 73 | 8 | -1.676562 | 2.711687 | -2.944136 |

---------------------------------------------------------------------

E(RTPSSh) = -2047.33847435 Hartree
Zero-point correction= 0.596163 (Hartree/Particle)
Thermal correction to Energy= 0.632346
Thermal correction to Enthalpy= 0.633291
Thermal correction to Gibbs Free Energy= 0.529341
Sum of electronic and zero-point Energies= -2046.742311
Sum of electronic and thermal Energies= -2046.706128
Sum of electronic and thermal Enthalpies= -2046.705184
Sum of electronic and thermal Free Energies= -2046.809134

**Table S61**: Cartesian coordinates obtained for the transition state of the **[Bi(macropa)]$^+$** Δ(δδλ)(λδδ)⇆ Δ(δδδ)(λδδ) equilibrum from geometry optimizations (1 imaginary frequency).

| Center Number | Atomic Number | Coordinates (Angstroms) X | Y | Z |
|---|---|---|---|---|
| 1 | 6 | 2.053182 | 2.082109 | 2.031997 |
| 2 | 7 | 1.705505 | 2.273077 | 0.601013 |
| 3 | 8 | 1.958019 | -0.310563 | 2.047005 |
| 4 | 6 | 2.796008 | 0.804668 | 2.339571 |
| 5 | 6 | 1.057330 | 3.606539 | 0.460305 |
| 6 | 6 | -0.480519 | 3.637257 | 0.617288 |
| 7 | 8 | -1.033955 | 2.428879 | 1.150022 |
| 8 | 6 | -1.668291 | 2.593850 | 2.424280 |
| 9 | 6 | -2.451089 | 1.348421 | 2.731653 |
| 10 | 8 | -1.544108 | 0.250042 | 2.771791 |
| 11 | 8 | 0.988562 | -2.557731 | 0.820636 |
| 12 | 6 | 1.637023 | -2.664878 | 2.088746 |
| 13 | 6 | 2.647247 | -1.555085 | 2.192585 |
| 14 | 7 | -1.954141 | -2.082137 | 0.890627 |
| 15 | 6 | -1.287448 | -3.331515 | 1.314141 |
| 16 | 6 | 0.021026 | -3.590104 | 0.588046 |
| 17 | 6 | -2.180388 | -0.977443 | 3.124397 |
| 18 | 6 | -2.884814 | -1.627125 | 1.945197 |
| 19 | 6 | 2.914110 | 2.229229 | -0.243010 |
| 20 | 6 | -2.701320 | -2.316580 | -0.351384 |
| 21 | 6 | -3.130352 | -1.049499 | -1.045290 |
| 22 | 6 | 3.254493 | 0.879945 | -0.825331 |
| 23 | 6 | -2.496000 | 0.992705 | -1.948545 |
| 24 | 6 | -3.730913 | 1.171870 | -2.556360 |
| 25 | 6 | -4.702119 | 0.195826 | -2.372554 |
| 26 | 6 | -4.393000 | -0.932855 | -1.624228 |
| 27 | 6 | 4.560628 | 0.593021 | -1.223717 |
| 28 | 6 | 4.820904 | -0.579552 | -1.917702 |
| 29 | 6 | 3.768121 | -1.435774 | -2.222179 |
| 30 | 6 | 2.498104 | -1.097468 | -1.783822 |
| 31 | 83 | 0.021004 | 0.069972 | -0.067369 |
| 32 | 1 | 1.126915 | 2.106355 | 2.605034 |
| 33 | 1 | 2.679052 | 2.919289 | 2.370103 |
| 34 | 1 | 3.057243 | 0.799259 | 3.403705 |
| 35 | 1 | 3.729827 | 0.728615 | 1.770153 |
| 36 | 1 | 1.278060 | 3.981787 | -0.536234 |
| 37 | 1 | 1.518546 | 4.296352 | 1.175494 |
| 38 | 1 | -0.937279 | 3.793882 | -0.358332 |
| 39 | 1 | -0.760230 | 4.473865 | 1.259462 |
| 40 | 1 | -2.353507 | 3.445982 | 2.387222 |
| 41 | 1 | -0.916380 | 2.780474 | 3.198601 |
| 42 | 1 | -3.220185 | 1.194031 | 1.967196 |
| 43 | 1 | -2.945443 | 1.464012 | 3.703738 |
| 44 | 1 | 2.145046 | -3.632377 | 2.165325 |
| 45 | 1 | 0.911348 | -2.584890 | 2.903624 |
| 46 | 1 | 3.133260 | -1.599567 | 3.173041 |
| 47 | 1 | 3.413973 | -1.645411 | 1.415311 |
| 48 | 1 | -1.952249 | -4.194331 | 1.163620 |
| 49 | 1 | -1.100294 | -3.269590 | 2.385387 |

| | | | | |
|---|---|---|---|---|
| 50 | 1 | 0.430456 | -4.552949 | 0.910749 |
| 51 | 1 | -0.119416 | -3.620406 | -0.490976 |
| 52 | 1 | -2.907762 | -0.803760 | 3.925274 |
| 53 | 1 | -1.393357 | -1.616785 | 3.524848 |
| 54 | 1 | -3.492713 | -2.468615 | 2.308696 |
| 55 | 1 | -3.575331 | -0.908973 | 1.499105 |
| 56 | 1 | 3.787300 | 2.631591 | 0.283357 |
| 57 | 1 | 2.744599 | 2.879328 | -1.103844 |
| 58 | 1 | -3.581933 | -2.948195 | -0.174801 |
| 59 | 1 | -2.047266 | -2.851246 | -1.044088 |
| 60 | 1 | -3.901363 | 2.051879 | -3.159777 |
| 61 | 1 | -5.681720 | 0.302759 | -2.820809 |
| 62 | 1 | -5.116096 | -1.726577 | -1.489604 |
| 63 | 1 | 5.353822 | 1.293523 | -0.996937 |
| 64 | 1 | 5.830069 | -0.815682 | -2.230387 |
| 65 | 1 | 3.907632 | -2.346423 | -2.786676 |
| 66 | 7 | 2.261887 | 0.022921 | -1.083316 |
| 67 | 7 | -2.223936 | -0.073481 | -1.186198 |
| 68 | 6 | -1.357900 | 1.954111 | -2.185989 |
| 69 | 8 | -0.216699 | 1.638932 | -1.649190 |
| 70 | 8 | -1.535102 | 2.949242 | -2.881852 |
| 71 | 6 | 1.291352 | -1.936612 | -2.122722 |
| 72 | 8 | 0.151328 | -1.480323 | -1.704075 |
| 73 | 8 | 1.426279 | -2.958215 | -2.789597 |

---------------------------------------------------------------------

E(RTPSSh) = -2047.32832150 Hartree

Zero-point correction= 0.596153 (Hartree/Particle)

Thermal correction to Energy= 0.631694

Thermal correction to Enthalpy= 0.632638

Thermal correction to Gibbs Free Energy= 0.530699

Sum of electronic and zero-point Energies= -2046.732169

Sum of electronic and thermal Energies= -2046.696628

Sum of electronic and thermal Enthalpies= -2046.695683

Sum of electronic and thermal Free Energies= -2046.797622

**Table S62**: Cartesian coordinates (Å) of the Δ(δδδ)(λδδ) conformer of **[Bi(macropa)]**$^{+}$ from geometry optimizations (0 imaginary frequencies).

| Center Number | Atomic Number | Coordinates (Angstroms) X | Y | Z |
|---|---|---|---|---|
| 1 | 6 | -2.628066 | -1.436160 | 2.349102 |
| 2 | 7 | -1.845199 | -1.973651 | 1.210012 |
| 3 | 8 | -2.242883 | 0.873547 | 1.912728 |
| 4 | 6 | -3.269330 | -0.089116 | 2.124429 |
| 5 | 6 | -1.176241 | -3.211643 | 1.689812 |
| 6 | 6 | 0.090016 | -3.557974 | 0.930862 |
| 7 | 8 | 1.084764 | -2.533780 | 1.074409 |
| 8 | 6 | 1.788966 | -2.615312 | 2.317046 |
| 9 | 6 | 2.554901 | -1.335703 | 2.511887 |
| 10 | 8 | 1.606876 | -0.266328 | 2.563909 |
| 11 | 8 | -0.870675 | 2.823408 | 0.553856 |
| 12 | 6 | -1.608594 | 3.152520 | 1.729700 |
| 13 | 6 | -2.767046 | 2.197734 | 1.820388 |
| 14 | 7 | 2.045497 | 2.090273 | 0.737311 |
| 15 | 6 | 1.448526 | 3.398923 | 1.083694 |
| 16 | 6 | 0.203434 | 3.726626 | 0.279429 |
| 17 | 6 | 2.180753 | 0.980088 | 2.965848 |
| 18 | 6 | 2.937574 | 1.650384 | 1.833798 |
| 19 | 6 | -2.711603 | -2.307313 | 0.067821 |
| 20 | 6 | 2.816778 | 2.205208 | -0.506833 |
| 21 | 6 | 3.181335 | 0.867670 | -1.095551 |
| 22 | 6 | -3.098939 | -1.146236 | -0.814382 |
| 23 | 6 | 2.452034 | -1.229377 | -1.778576 |
| 24 | 6 | 3.668667 | -1.517358 | -2.380004 |
| 25 | 6 | 4.677893 | -0.564631 | -2.314873 |
| 26 | 6 | 4.426271 | 0.645232 | -1.680919 |
| 27 | 6 | -4.343716 | -1.108707 | -1.443367 |
| 28 | 6 | -4.623187 | -0.092424 | -2.346258 |
| 29 | 6 | -3.645361 | 0.855414 | -2.627135 |
| 30 | 6 | -2.426352 | 0.755415 | -1.974470 |
| 31 | 83 | 0.013409 | -0.006585 | 0.006808 |
| 32 | 1 | -1.943191 | -1.346092 | 3.193078 |
| 33 | 1 | -3.416390 | -2.149086 | 2.630381 |
| 34 | 1 | -3.838273 | 0.170354 | 3.025349 |
| 35 | 1 | -3.970144 | -0.087896 | 1.282176 |
| 36 | 1 | -1.868342 | -4.063048 | 1.644979 |
| 37 | 1 | -0.933343 | -3.062187 | 2.741436 |
| 38 | 1 | -0.089820 | -3.653580 | -0.137813 |
| 39 | 1 | 0.485051 | -4.508651 | 1.302958 |
| 40 | 1 | 2.479581 | -3.465246 | 2.293356 |
| 41 | 1 | 1.089487 | -2.753751 | 3.146230 |
| 42 | 1 | 3.266269 | -1.187986 | 1.692918 |
| 43 | 1 | 3.112890 | -1.385514 | 3.453278 |
| 44 | 1 | -1.983420 | 4.179699 | 1.658248 |
| 45 | 1 | -0.980639 | 3.070423 | 2.623051 |
| 46 | 1 | -3.362410 | 2.426322 | 2.711791 |
| 47 | 1 | -3.410023 | 2.284830 | 0.936917 |
| 48 | 1 | 2.187640 | 4.201002 | 0.945303 |
| 49 | 1 | 1.197667 | 3.382440 | 2.144494 |

| | | | | |
|---|---|---|---|---|
| 50 | 1 | -0.110961 | 4.751507 | 0.502185 |
| 51 | 1 | 0.385231 | 3.648514 | -0.791587 |
| 52 | 1 | 2.854500 | 0.819597 | 3.814263 |
| 53 | 1 | 1.345821 | 1.592286 | 3.307685 |
| 54 | 1 | 3.508414 | 2.502578 | 2.227111 |
| 55 | 1 | 3.664821 | 0.947772 | 1.423629 |
| 56 | 1 | -3.619702 | -2.824489 | 0.402590 |
| 57 | 1 | -2.159298 | -3.002687 | -0.569716 |
| 58 | 1 | 3.726952 | 2.800689 | -0.360932 |
| 59 | 1 | 2.200519 | 2.723759 | -1.244835 |
| 60 | 1 | 3.796061 | -2.461406 | -2.890000 |
| 61 | 1 | 5.643524 | -0.754140 | -2.766119 |
| 62 | 1 | 5.180755 | 1.419779 | -1.638497 |
| 63 | 1 | -5.076782 | -1.873139 | -1.221380 |
| 64 | 1 | -5.588015 | -0.047728 | -2.835175 |
| 65 | 1 | -3.799086 | 1.651158 | -3.341757 |
| 66 | 7 | -2.181554 | -0.208034 | -1.074934 |
| 67 | 7 | 2.234685 | -0.079740 | -1.126522 |
| 68 | 6 | 1.271315 | -2.158659 | -1.905955 |
| 69 | 8 | 0.141195 | -1.705219 | -1.451589 |
| 70 | 8 | 1.401804 | -3.243907 | -2.462274 |
| 71 | 6 | -1.273693 | 1.673701 | -2.305655 |
| 72 | 8 | -0.135135 | 1.373246 | -1.757494 |
| 73 | 8 | -1.438484 | 2.600121 | -3.092391 |

---------------------------------------------------------------------

E(RTPSSh) = -2047.33742910
Zero-point correction= 0.596171 (Hartree/Particle)
Thermal correction to Energy= 0.632277
Thermal correction to Enthalpy= 0.633222
Thermal correction to Gibbs Free Energy= 0.529848
Sum of electronic and zero-point Energies= -2046.741258
Sum of electronic and thermal Energies= -2046.705152
Sum of electronic and thermal Enthalpies= -2046.704208
Sum of electronic and thermal Free Energies= -2046.807582

**Table S63**: Cartesian coordinates obtained for the transition state of the **[Bi(macropa)]**$^+$ Δ(δδδ)(λδδ)⇆ Δ(δδδ)(δδδ) equilibrum from geometry optimizations (1 imaginary frequency).

| Center Number | Atomic Number | Coordinates (Angstroms) X | Y | Z |
|---|---|---|---|---|
| 1 | 6 | 3.344964 | 1.480371 | 1.871255 |
| 2 | 7 | 2.245054 | 1.919821 | 0.972975 |
| 3 | 8 | 2.232393 | -0.744726 | 1.874886 |
| 4 | 6 | 3.300818 | 0.034310 | 2.410744 |
| 5 | 6 | 1.647483 | 3.164662 | 1.507868 |
| 6 | 6 | 0.313804 | 3.540821 | 0.888896 |
| 7 | 8 | -0.706637 | 2.565397 | 1.150118 |
| 8 | 6 | -1.273596 | 2.706583 | 2.456391 |
| 9 | 6 | -2.241224 | 1.579933 | 2.687441 |
| 10 | 8 | -1.501909 | 0.357049 | 2.664328 |
| 11 | 8 | 0.631571 | -2.715665 | 0.748489 |
| 12 | 6 | 1.210466 | -2.906499 | 2.041114 |
| 13 | 6 | 2.494669 | -2.130553 | 2.113844 |
| 14 | 7 | -2.224348 | -1.896093 | 0.821086 |
| 15 | 6 | -1.719683 | -3.229348 | 1.214080 |
| 16 | 6 | -0.450419 | -3.617190 | 0.478197 |
| 17 | 6 | -2.280475 | -0.777120 | 3.049698 |
| 18 | 6 | -3.082562 | -1.340930 | 1.890894 |
| 19 | 6 | 2.781946 | 2.146710 | -0.377875 |
| 20 | 6 | -2.999898 | -2.009566 | -0.420734 |
| 21 | 6 | -3.283628 | -0.672257 | -1.049395 |
| 22 | 6 | 3.101285 | 0.866976 | -1.105498 |
| 23 | 6 | -2.435870 | 1.360231 | -1.784066 |
| 24 | 6 | -3.643559 | 1.716737 | -2.366133 |
| 25 | 6 | -4.708787 | 0.830192 | -2.269958 |
| 26 | 6 | -4.522104 | -0.381555 | -1.617716 |
| 27 | 6 | 4.334800 | 0.644998 | -1.712526 |
| 28 | 6 | 4.531321 | -0.506400 | -2.466863 |
| 29 | 6 | 3.478254 | -1.396432 | -2.633338 |
| 30 | 6 | 2.272919 | -1.111945 | -2.005963 |
| 31 | 83 | -0.037570 | -0.010922 | -0.035277 |
| 32 | 1 | 3.372882 | 2.150891 | 2.729998 |
| 33 | 1 | 4.294843 | 1.617876 | 1.349521 |
| 34 | 1 | 3.201437 | 0.046068 | 3.501717 |
| 35 | 1 | 4.248998 | -0.457902 | 2.169976 |
| 36 | 1 | 2.336274 | 4.013730 | 1.382940 |
| 37 | 1 | 1.514284 | 3.019052 | 2.579761 |
| 38 | 1 | 0.373776 | 3.626727 | -0.193798 |
| 39 | 1 | -0.000506 | 4.509336 | 1.291677 |
| 40 | 1 | -1.799480 | 3.665384 | 2.522961 |
| 41 | 1 | -0.490266 | 2.680353 | 3.219656 |
| 42 | 1 | -3.022283 | 1.581028 | 1.919669 |
| 43 | 1 | -2.715466 | 1.708304 | 3.666821 |
| 44 | 1 | 1.423072 | -3.970063 | 2.194759 |
| 45 | 1 | 0.526144 | -2.570358 | 2.825807 |
| 46 | 1 | 2.918172 | -2.266305 | 3.115163 |
| 47 | 1 | 3.218830 | -2.494208 | 1.376128 |
| 48 | 1 | -2.485216 | -3.997471 | 1.037231 |
| 49 | 1 | -1.530624 | -3.220070 | 2.286904 |

| | | | | |
|---|---|---|---|---|
| 50 | 1 | -0.156492 | -4.630962 | 0.768852 |
| 51 | 1 | -0.587964 | -3.590768 | -0.600890 |
| 52 | 1 | -2.959809 | -0.503153 | 3.863494 |
| 53 | 1 | -1.572274 | -1.508104 | 3.440751 |
| 54 | 1 | -3.773650 | -2.109206 | 2.264800 |
| 55 | 1 | -3.693874 | -0.546869 | 1.458408 |
| 56 | 1 | 3.678822 | 2.778528 | -0.348973 |
| 57 | 1 | 2.023544 | 2.663283 | -0.967597 |
| 58 | 1 | -3.944416 | -2.542786 | -0.254281 |
| 59 | 1 | -2.413669 | -2.590074 | -1.136693 |
| 60 | 1 | -3.720977 | 2.661071 | -2.885544 |
| 61 | 1 | -5.668554 | 1.073003 | -2.707898 |
| 62 | 1 | -5.323528 | -1.104906 | -1.544787 |
| 63 | 1 | 5.124509 | 1.375599 | -1.596631 |
| 64 | 1 | 5.489173 | -0.694707 | -2.934901 |
| 65 | 1 | 3.562818 | -2.286369 | -3.240392 |
| 66 | 7 | 2.113430 | -0.028925 | -1.237938 |
| 67 | 7 | -2.276531 | 0.208055 | -1.119709 |
| 68 | 6 | -1.203900 | 2.213259 | -1.941104 |
| 69 | 8 | -0.098470 | 1.685216 | -1.507909 |
| 70 | 8 | -1.276140 | 3.304990 | -2.494495 |
| 71 | 6 | 1.041391 | -1.955141 | -2.228726 |
| 72 | 8 | -0.059463 | -1.494826 | -1.707138 |
| 73 | 8 | 1.105660 | -2.970502 | -2.911370 |

---------------------------------------------------------------------

E(RTPSSh) = -2047.32161067 Hartree
Zero-point correction= 0.596240 (Hartree/Particle)
Thermal correction to Energy= 0.631886
Thermal correction to Enthalpy= 0.632830
Thermal correction to Gibbs Free Energy= 0.529171
Sum of electronic and zero-point Energies= -2046.725371
Sum of electronic and thermal Energies= -2046.689725
Sum of electronic and thermal Enthalpies= -2046.688780
Sum of electronic and thermal Free Energies= -2046.792440

**Table S64**: Cartesian coordinates obtained for the transition state of the **[Bi(macropa)]**$^{+}$ Δ(δδλ)(λδδ)⇆ Δ(δδλ)(δδδ) equilibrum from geometry optimizations (1 imaginary frequency).

| Center Number | Atomic Number | Coordinates (Angstroms) X | Y | Z |
|---|---|---|---|---|
| 1 | 6 | -1.850254 | 1.463733 | 2.738571 |
| 2 | 7 | -1.950641 | 0.146805 | 2.068222 |
| 3 | 8 | -2.024999 | 2.166057 | 0.424675 |
| 4 | 6 | -1.772873 | 2.648407 | 1.746543 |
| 5 | 6 | -1.657788 | -0.972404 | 2.997477 |
| 6 | 6 | -0.330070 | -0.844699 | 3.724362 |
| 7 | 8 | 0.695609 | -0.422298 | 2.826025 |
| 8 | 6 | 1.789150 | 0.186625 | 3.513798 |
| 9 | 6 | 2.700199 | 0.826772 | 2.500711 |
| 10 | 8 | 1.932589 | 1.769002 | 1.751478 |
| 11 | 8 | -0.494744 | 2.273833 | -1.858316 |
| 12 | 6 | -0.973912 | 3.502042 | -1.303945 |
| 13 | 6 | -2.250196 | 3.174290 | -0.565922 |
| 14 | 7 | 2.340615 | 1.538659 | -1.225206 |
| 15 | 6 | 1.919381 | 2.669820 | -2.074338 |
| 16 | 6 | 0.593560 | 2.431378 | -2.775658 |
| 17 | 6 | 2.730524 | 2.661804 | 0.974869 |
| 18 | 6 | 3.346226 | 1.994116 | -0.242534 |
| 19 | 6 | -3.316047 | -0.038478 | 1.533219 |
| 20 | 6 | 2.910812 | 0.465878 | -2.048527 |
| 21 | 6 | 3.065944 | -0.825248 | -1.286952 |
| 22 | 6 | -3.399531 | -0.752919 | 0.210973 |
| 23 | 6 | 2.059854 | -2.438458 | 0.042193 |
| 24 | 6 | 3.166127 | -3.273955 | -0.035961 |
| 25 | 6 | 4.264060 | -2.844614 | -0.770566 |
| 26 | 6 | 4.207071 | -1.615113 | -1.415008 |
| 27 | 6 | -4.584997 | -1.389231 | -0.161113 |
| 28 | 6 | -4.696486 | -1.945722 | -1.425516 |
| 29 | 6 | -3.617364 | -1.862274 | -2.299082 |
| 30 | 6 | -2.461107 | -1.240318 | -1.858611 |
| 31 | 83 | -0.118055 | 0.049096 | -0.045809 |
| 32 | 1 | -0.944037 | 1.467024 | 3.336870 |
| 33 | 1 | -2.696827 | 1.597923 | 3.421153 |
| 34 | 1 | -0.784347 | 3.113572 | 1.793365 |
| 35 | 1 | -2.520031 | 3.404860 | 2.000154 |
| 36 | 1 | -1.679379 | -1.884304 | 2.403852 |
| 37 | 1 | -2.440842 | -1.044092 | 3.764701 |
| 38 | 1 | -0.073923 | -1.813287 | 4.167513 |
| 39 | 1 | -0.404783 | -0.119449 | 4.541657 |
| 40 | 1 | 2.344497 | -0.562254 | 4.090031 |
| 41 | 1 | 1.403780 | 0.945025 | 4.205366 |
| 42 | 1 | 3.134487 | 0.066370 | 1.844056 |
| 43 | 1 | 3.516643 | 1.335957 | 3.025842 |
| 44 | 1 | -1.184822 | 4.214807 | -2.108959 |
| 45 | 1 | -0.231202 | 3.945097 | -0.633458 |
| 46 | 1 | -2.670189 | 4.073231 | -0.107137 |
| 47 | 1 | -2.979728 | 2.754920 | -1.260341 |
| 48 | 1 | 2.685947 | 2.893033 | -2.831053 |
| 49 | 1 | 1.838786 | 3.553853 | -1.441333 |

| | | | | |
|---|---|---|---|---|
| 50 | 1 | 0.384011 | 3.268728 | -3.449127 |
| 51 | 1 | 0.609031 | 1.513651 | -3.361618 |
| 52 | 1 | 3.530846 | 3.079021 | 1.597136 |
| 53 | 1 | 2.065705 | 3.478092 | 0.691315 |
| 54 | 1 | 4.058115 | 2.688100 | -0.712072 |
| 55 | 1 | 3.924274 | 1.126655 | 0.080377 |
| 56 | 1 | -3.763998 | 0.943405 | 1.381630 |
| 57 | 1 | -3.943375 | -0.564529 | 2.260705 |
| 58 | 1 | 3.879758 | 0.756128 | -2.475817 |
| 59 | 1 | 2.229548 | 0.271560 | -2.879784 |
| 60 | 1 | 3.141185 | -4.233620 | 0.460181 |
| 61 | 1 | 5.147699 | -3.464637 | -0.853254 |
| 62 | 1 | 5.034696 | -1.262871 | -2.016904 |
| 63 | 1 | -5.405371 | -1.437079 | 0.543172 |
| 64 | 1 | -5.610884 | -2.441075 | -1.726166 |
| 65 | 1 | -3.647114 | -2.273610 | -3.297682 |
| 66 | 7 | -2.365076 | -0.700726 | -0.633013 |
| 67 | 7 | 2.030585 | -1.234777 | -0.541889 |
| 68 | 6 | 0.787145 | -2.884788 | 0.718041 |
| 69 | 8 | -0.240798 | -2.103163 | 0.570006 |
| 70 | 8 | 0.753086 | -3.957362 | 1.312410 |
| 71 | 6 | -1.241307 | -1.138607 | -2.739921 |
| 72 | 8 | -0.183756 | -0.630145 | -2.188134 |
| 73 | 8 | -1.295320 | -1.528812 | -3.902099 |

---------------------------------------------------------------------

E(RTPSSh) = -2047.32670469 Hartree
Zero-point correction= 0.595900 (Hartree/Particle)
Thermal correction to Energy= 0.631636
Thermal correction to Enthalpy= 0.632580
Thermal correction to Gibbs Free Energy= 0.529216
Sum of electronic and zero-point Energies= -2046.730805
Sum of electronic and thermal Energies= -2046.695069
Sum of electronic and thermal Enthalpies= -2046.694125
Sum of electronic and thermal Free Energies= -2046.797489

**Table S65**: Cartesian coordinates (Å) of the Δ(δδλ)(δδδ) conformer of **[Bi(macropa)]**$^{+}$ from geometry optimizations (0 imaginary frequencies).

| Center Number | Atomic Number | Coordinates (Angstroms) X | Y | Z |
|---|---|---|---|---|
| 1 | 6 | -2.123821 | 2.230250 | 2.049477 |
| 2 | 7 | -2.106302 | 0.761799 | 1.820331 |
| 3 | 8 | -2.310583 | 2.455086 | -0.320740 |
| 4 | 6 | -1.663789 | 3.005996 | 0.824421 |
| 5 | 6 | -1.838000 | -0.010007 | 3.056706 |
| 6 | 6 | -0.544179 | 0.372998 | 3.753628 |
| 7 | 8 | 0.515245 | 0.517582 | 2.807100 |
| 8 | 6 | 1.596759 | 1.283924 | 3.340485 |
| 9 | 6 | 2.588739 | 1.556778 | 2.241762 |
| 10 | 8 | 1.905712 | 2.243402 | 1.192799 |
| 11 | 8 | -0.445416 | 1.765682 | -2.322011 |
| 12 | 6 | -0.787738 | 3.142218 | -2.118762 |
| 13 | 6 | -2.177665 | 3.209121 | -1.521839 |
| 14 | 7 | 2.378280 | 1.097413 | -1.536138 |
| 15 | 6 | 1.983619 | 1.943238 | -2.681105 |
| 16 | 6 | 0.627167 | 1.574564 | -3.256308 |
| 17 | 6 | 2.780972 | 2.824813 | 0.227884 |
| 18 | 6 | 3.386135 | 1.795324 | -0.709459 |
| 19 | 6 | -3.415820 | 0.345008 | 1.278636 |
| 20 | 6 | 2.940905 | -0.172992 | -2.011084 |
| 21 | 6 | 3.070418 | -1.188176 | -0.905371 |
| 22 | 6 | -3.386975 | -0.781314 | 0.282932 |
| 23 | 6 | 2.030212 | -2.327642 | 0.829785 |
| 24 | 6 | 3.124649 | -3.160734 | 1.014842 |
| 25 | 6 | 4.233817 | -2.980006 | 0.198022 |
| 26 | 6 | 4.199649 | -1.995152 | -0.781121 |
| 27 | 6 | -4.515203 | -1.584812 | 0.107237 |
| 28 | 6 | -4.537057 | -2.523231 | -0.912408 |
| 29 | 6 | -3.427664 | -2.645881 | -1.742250 |
| 30 | 6 | -2.329953 | -1.839064 | -1.494564 |
| 31 | 83 | -0.097342 | 0.010468 | -0.062990 |
| 32 | 1 | -1.448037 | 2.478882 | 2.864664 |
| 33 | 1 | -3.128347 | 2.545005 | 2.352296 |
| 34 | 1 | -0.574311 | 2.932820 | 0.726174 |
| 35 | 1 | -1.922518 | 4.064813 | 0.939343 |
| 36 | 1 | -1.814189 | -1.060492 | 2.771993 |
| 37 | 1 | -2.652732 | 0.128407 | 3.780945 |
| 38 | 1 | -0.294735 | -0.401096 | 4.487776 |
| 39 | 1 | -0.657878 | 1.315879 | 4.299428 |
| 40 | 1 | 2.088043 | 0.736139 | 4.152778 |
| 41 | 1 | 1.206710 | 2.227426 | 3.739099 |
| 42 | 1 | 3.024947 | 0.621096 | 1.877174 |
| 43 | 1 | 3.397111 | 2.181561 | 2.638638 |
| 44 | 1 | -0.793025 | 3.665120 | -3.081239 |
| 45 | 1 | -0.053056 | 3.630707 | -1.471677 |
| 46 | 1 | -2.442263 | 4.258299 | -1.345403 |
| 47 | 1 | -2.898257 | 2.773848 | -2.214503 |
| 48 | 1 | 2.734925 | 1.885861 | -3.481697 |
| 49 | 1 | 1.964744 | 2.980549 | -2.346540 |

| | | | | |
|---|---|---|---|---|
| 50 | 1 | 0.437196 | 2.178073 | -4.149756 |
| 51 | 1 | 0.582751 | 0.522314 | -3.531458 |
| 52 | 1 | 3.588560 | 3.364685 | 0.735557 |
| 53 | 1 | 2.179887 | 3.556517 | -0.312976 |
| 54 | 1 | 4.135433 | 2.285336 | -1.347320 |
| 55 | 1 | 3.918630 | 1.046404 | -0.120732 |
| 56 | 1 | -3.845049 | 1.197824 | 0.752468 |
| 57 | 1 | -4.103251 | 0.085909 | 2.091726 |
| 58 | 1 | 3.918950 | -0.027024 | -2.487732 |
| 59 | 1 | 2.265320 | -0.590225 | -2.760847 |
| 60 | 1 | 3.082518 | -3.930795 | 1.771728 |
| 61 | 1 | 5.108876 | -3.607124 | 0.311751 |
| 62 | 1 | 5.036645 | -1.845750 | -1.450516 |
| 63 | 1 | -5.363459 | -1.459571 | 0.767475 |
| 64 | 1 | -5.406372 | -3.151393 | -1.059312 |
| 65 | 1 | -3.389774 | -3.352355 | -2.558819 |
| 66 | 7 | -2.317617 | -0.933022 | -0.503909 |
| 67 | 7 | 2.024600 | -1.349308 | -0.083620 |
| 68 | 6 | 0.743762 | -2.538549 | 1.586593 |
| 69 | 8 | -0.272553 | -1.832325 | 1.182255 |
| 70 | 8 | 0.684190 | -3.373768 | 2.480907 |
| 71 | 6 | -1.083174 | -1.934660 | -2.334364 |
| 72 | 8 | -0.077531 | -1.213767 | -1.937059 |
| 73 | 8 | -1.063051 | -2.656484 | -3.324707 |

---------------------------------------------------------------------

E(RTPSSh) = -2047.32835340 Hartree
Zero-point correction= 0.596236 (Hartree/Particle)
Thermal correction to Energy= 0.632548
Thermal correction to Enthalpy= 0.633492
Thermal correction to Gibbs Free Energy= 0.528844
Sum of electronic and zero-point Energies= -2046.732118
Sum of electronic and thermal Energies= -2046.695805
Sum of electronic and thermal Enthalpies= -2046.694861
Sum of electronic and thermal Free Energies= -2046.799509

**Table S66**: Cartesian coordinates obtained for the transition state of the **[Bi(macropa)]**$^{+}$ Δ(δδλ)(δδδ) ⇆ Δ(δδδ)(δδδ) equilibrum from geometry optimizations (1 imaginary frequency).

| Center Number | Atomic Number | Coordinates (Angstroms) X | Y | Z |
|---|---|---|---|---|
| 1 | 6 | -2.051835 | -2.081782 | 2.033187 |
| 2 | 7 | -1.704807 | -2.273124 | 0.602095 |
| 3 | 8 | -1.957500 | 0.310996 | 2.047037 |
| 4 | 6 | -2.794852 | -0.804417 | 2.340675 |
| 5 | 6 | -1.056333 | -3.606398 | 0.461332 |
| 6 | 6 | 0.481466 | -3.636976 | 0.619119 |
| 7 | 8 | 1.034480 | -2.428134 | 1.151182 |
| 8 | 6 | 1.670088 | -2.592532 | 2.424869 |
| 9 | 6 | 2.452201 | -1.346447 | 2.731382 |
| 10 | 8 | 1.544516 | -0.248662 | 2.771480 |
| 11 | 8 | -0.988991 | 2.557814 | 0.819803 |
| 12 | 6 | -1.637389 | 2.665494 | 2.087917 |
| 13 | 6 | -2.647107 | 1.555300 | 2.192618 |
| 14 | 7 | 1.953850 | 2.082985 | 0.889573 |
| 15 | 6 | 1.286919 | 3.332425 | 1.312518 |
| 16 | 6 | -0.021746 | 3.590303 | 0.586525 |
| 17 | 6 | 2.179995 | 0.979324 | 3.123682 |
| 18 | 6 | 2.884547 | 1.628823 | 1.944465 |
| 19 | 6 | -2.913827 | -2.229760 | -0.241374 |
| 20 | 6 | 2.700936 | 2.316766 | -0.352640 |
| 21 | 6 | 3.130133 | 1.049301 | -1.045738 |
| 22 | 6 | -3.254560 | -0.880833 | -0.824335 |
| 23 | 6 | 2.495855 | -0.993250 | -1.948315 |
| 24 | 6 | 3.730950 | -1.172796 | -2.555651 |
| 25 | 6 | 4.702223 | -0.196811 | -2.371888 |
| 26 | 6 | 4.392958 | 0.932260 | -1.624201 |
| 27 | 6 | -4.560900 | -0.594277 | -1.222399 |
| 28 | 6 | -4.821553 | 0.577915 | -1.916854 |
| 29 | 6 | -3.768967 | 1.434132 | -2.222120 |
| 30 | 6 | -2.498766 | 1.096192 | -1.784073 |
| 31 | 83 | -0.021071 | -0.069876 | -0.067516 |
| 32 | 1 | -1.125285 | -2.105608 | 2.605783 |
| 33 | 1 | -2.677357 | -2.919002 | 2.371842 |
| 34 | 1 | -3.055331 | -0.798621 | 3.404993 |
| 35 | 1 | -3.729109 | -0.728969 | 1.771881 |
| 36 | 1 | -1.276559 | -3.981420 | -0.535400 |
| 37 | 1 | -1.517736 | -4.296471 | 1.176166 |
| 38 | 1 | 0.938766 | -3.794344 | -0.356156 |
| 39 | 1 | 0.760887 | -4.473083 | 1.262069 |
| 40 | 1 | 2.355991 | -3.444092 | 2.387271 |
| 41 | 1 | 0.918975 | -2.779717 | 3.199829 |
| 42 | 1 | 3.220841 | -1.191894 | 1.966502 |
| 43 | 1 | 2.947084 | -1.461321 | 3.703282 |
| 44 | 1 | -2.145940 | 3.632767 | 2.163787 |
| 45 | 1 | -0.911609 | 2.586547 | 2.902807 |
| 46 | 1 | -3.132581 | 1.599822 | 3.173335 |
| 47 | 1 | -3.414313 | 1.645145 | 1.415760 |
| 48 | 1 | 1.951493 | 4.195322 | 1.161456 |
| 49 | 1 | 1.099983 | 3.270966 | 2.383844 |

| | | | | |
|---|---|---|---|---|
| 50 | 1 | -0.431365 | 4.553188 | 0.908856 |
| 51 | 1 | 0.118527 | 3.620178 | -0.492533 |
| 52 | 1 | 2.907102 | 0.806480 | 3.924988 |
| 53 | 1 | 1.392419 | 1.618484 | 3.523383 |
| 54 | 1 | 3.491911 | 2.470741 | 2.307846 |
| 55 | 1 | 3.575545 | 0.910890 | 1.498774 |
| 56 | 1 | -3.786718 | -2.631870 | 0.285670 |
| 57 | 1 | -2.744781 | -2.880351 | -1.101932 |
| 58 | 1 | 3.581494 | 2.948584 | -0.176514 |
| 59 | 1 | 2.046792 | 2.850930 | -1.045650 |
| 60 | 1 | 3.901474 | -2.053048 | -3.158692 |
| 61 | 1 | 5.681976 | -0.304069 | -2.819734 |
| 62 | 1 | 5.116075 | 1.725988 | -1.489724 |
| 63 | 1 | -5.353913 | -1.294781 | -0.994999 |
| 64 | 1 | -5.830848 | 0.813800 | -2.229305 |
| 65 | 1 | -3.908819 | 2.344506 | -2.786976 |
| 66 | 7 | -2.262171 | -0.023862 | -1.083116 |
| 67 | 7 | 2.223689 | 0.073270 | -1.186464 |
| 68 | 6 | 1.357654 | -1.954561 | -2.185828 |
| 69 | 8 | 0.216611 | -1.639655 | -1.648578 |
| 70 | 8 | 1.534673 | -2.949329 | -2.882259 |
| 71 | 6 | -1.292236 | 1.935337 | -2.123677 |
| 72 | 8 | -0.152032 | 1.479499 | -1.704921 |
| 73 | 8 | -1.427409 | 2.956563 | -2.791054 |

---------------------------------------------------------------------

E(RTPSSh) = -2047.32832137 Hartree
Zero-point correction= 0.596146 (Hartree/Particle)
Thermal correction to Energy= 0.631691
Thermal correction to Enthalpy= 0.632635
Thermal correction to Gibbs Free Energy= 0.530681
Sum of electronic and zero-point Energies= -2046.732175
Sum of electronic and thermal Energies= -2046.696631
Sum of electronic and thermal Enthalpies= -2046.695687
Sum of electronic and thermal Free Energies= -2046.797640
